# Fluoride Exposure and Children’s IQ Scores: A Systematic Review and Meta-Analysis

**DOI:** 10.1001/jamapediatrics.2024.5542

**Published:** 2025-01-06

**Authors:** Kyla W. Taylor, Sorina E. Eftim, Christopher A. Sibrizzi, Robyn B. Blain, Kristen Magnuson, Pamela A. Hartman, Andrew A. Rooney, John R. Bucher

**Affiliations:** 1 Division of Translational Toxicology, National Institute of Environmental Health Sciences, National Institutes of Health, Research Triangle Park, North Carolina; 2 ICF, Reston, Virginia

## Abstract

**Importance:**

Previous meta-analyses suggest that fluoride exposure is adversely associated with children’s IQ scores. An individual’s total fluoride exposure comes primarily from fluoride in drinking water, food, and beverages.

**Objective:**

To perform a systematic review and meta-analysis of epidemiological studies investigating children’s IQ scores and prenatal or postnatal fluoride exposure.

**Data Sources:**

BIOSIS, Embase, PsycInfo, PubMed, Scopus, Web of Science, CNKI, and Wanfang, searched through October 2023.

**Study Selection:**

Studies reporting children’s IQ scores, fluoride exposure, and effect sizes.

**Data Extraction and Synthesis:**

Data were extracted into the Health Assessment Workplace Collaborative system. Study quality was evaluated using the OHAT risk-of-bias tool. Pooled standardized mean differences (SMDs) and regression coefficients were estimated with random-effects models.

**Main Outcomes and Measures:**

Children’s IQ scores.

**Results:**

Of 74 studies included (64 cross-sectional and 10 cohort studies), most were conducted in China (n = 45); other locations included Canada (n = 3), Denmark (n = 1), India (n = 12), Iran (n = 4), Mexico (n = 4), New Zealand (n = 1), Pakistan (n = 2), Spain (n = 1), and Taiwan (n = 1). Fifty-two studies were rated high risk of bias and 22 were rated low risk of bias. Sixty-four studies reported inverse associations between fluoride exposure measures and children’s IQ. Analysis of 59 studies with group-level measures of fluoride in drinking water, dental fluorosis, or other measures of fluoride exposure (47 high risk of bias, 12 low risk of bias; n = 20 932 children) showed an inverse association between fluoride exposure and IQ (pooled SMD, −0.45; 95% CI, −0.57 to −0.33; *P* < .001). In 31 studies reporting fluoride measured in drinking water, a dose-response association was found between exposed and reference groups (SMD, −0.15; 95% CI, −0.20 to −0.11; *P* < .001), and associations remained inverse when exposed groups were restricted to less than 4 mg/L and less than 2 mg/L; however, the association was null at less than 1.5 mg/L. In analyses restricted to low risk-of-bias studies, the association remained inverse when exposure was restricted to less than 4 mg/L, less than 2 mg/L, and less than 1.5 mg/L fluoride in drinking water. In 20 studies reporting fluoride measured in urine, there was an inverse dose-response association (SMD, −0.15; 95% CI, −0.23 to −0.07; *P* < .001). Associations remained inverse when exposed groups were restricted to less than 4 mg/L, less than 2 mg/L, and less than 1.5 mg/L fluoride in urine; the associations held in analyses restricted to the low risk-of-bias studies. Analysis of 13 studies with individual-level measures found an IQ score decrease of 1.63 points (95% CI, −2.33 to −0.93; *P* < .001) per 1-mg/L increase in urinary fluoride. Among low risk-of-bias studies, there was an IQ score decrease of 1.14 points (95% CI, –1.68 to –0.61; *P* < .001). Associations remained inverse when stratified by risk of bias, sex, age, outcome assessment type, country, exposure timing, and exposure matrix.

**Conclusions and Relevance:**

This systematic review and meta-analysis found inverse associations and a dose-response association between fluoride measurements in urine and drinking water and children’s IQ across the large multicountry epidemiological literature. There were limited data and uncertainty in the dose-response association between fluoride exposure and children’s IQ when fluoride exposure was estimated by drinking water alone at concentrations less than 1.5 mg/L. These findings may inform future comprehensive public health risk-benefit assessments of fluoride exposures.

## Key Points

**Question:**

Is fluoride exposure associated with children’s IQ scores?

**Findings:**

Despite differences in exposure and outcome measures and risk of bias across studies, and when using group-level and individual-level exposure estimates, this systematic review and meta-analysis of 74 cross-sectional and prospective cohort studies found significant inverse associations between fluoride exposure and children’s IQ scores. For fluoride measured in water, associations remained inverse when exposed groups were restricted to less than 4 mg/L or less than 2 mg/L but not when restricted to less than 1.5 mg/L; for fluoride measured in urine, associations remained inverse at less than 4 mg/L, less than 2 mg/L, and less than 1.5 mg/L; and among the subset of low risk-of-bias studies, there were inverse associations when exposed groups were restricted to less than 4 mg/L, less than 2 mg/L, and less than 1.5 mg/L for analyses of fluoride measured both in water and in urine.

**Meaning:**

This comprehensive meta-analysis may inform future risk-benefit assessments of the use of fluoride in children’s oral health.

## Teaser

This systematic review and meta-analysis investigates the association between fluoride exposure and children’s IQ scores.

## Introduction

Fluoride from natural sources occurs in some community water systems (CWSs), and in the United States and some other countries, fluoride is added to public drinking water systems or salt for the prevention of tooth decay. For CWSs that add fluoride, the US Public Health Service recommends a fluoride concentration of 0.7 mg/L, the US Environmental Protection Agency’s (EPA’s) enforceable and nonenforceable standards for fluoride in drinking water are 4.0 mg/L and 2.0 mg/L,^1^ and the World Health Organization’s (WHO’s) drinking water quality guideline for fluoride is 1.5 mg/L.^2^ Water and water-based beverages are the main source of systemic fluoride intake. In the United States, the Centers for Disease Control and Prevention (CDC) estimates that water and processed beverages (eg, soda and juices) provide approximately 75% of a person’s fluoride intake,^3^ and EPA estimates that 40% to 70% of a person’s fluoride intake comes from fluoridated drinking water.^4^ However, an individual’s total exposure also reflects contributions from fluoride in other sources, such as food, dental products, industrial emissions, and pharmaceuticals.^4^ Accumulating evidence suggests that fluoride exposure may affect brain development. A 2006 report from the National Research Council (NRC) concluded that high levels of naturally occurring fluoride in drinking water may be of concern for neurotoxic effects.^5^ This finding was largely based on studies from endemic fluorosis areas in China that had limitations in study design or methods. Following the NRC review, studies from an additional 10 countries have been published (eFigure 1A in Supplement 1). Previous meta-analyses^6,7,8^ found an inverse association between fluoride exposure and children’s IQ. Since the most recent meta-analysis,^8^ 4 new studies on exposure to fluoride and children’s IQ have been published, including 3 studies^9,10,11^ that measured individual-level maternal and children’s urinary fluoride concentrations.

To incorporate newer evidence and increase transparency, objectivity, and rigor in the analysis of fluoride research, we conducted a systematic review and meta-analysis of studies that provided estimates of group-level and individual-level fluoride exposure in relation to children’s IQ scores.

## Methods

The search, selection, extraction, and risk-of-bias evaluation of studies were part of a larger systematic review.^12^ Brief methods are outlined herein, with detailed methods available in the protocol^13^ and the “Detailed Methods” section of eAppendix 1 in Supplement 1. This study follows the Meta-Analysis of Observational Studies in Epidemiology (MOOSE) reporting guidelines. Data analysis was conducted from June 2020 to January 2024. The most recent analysis update was performed in January and February 2024.

### Systematic Literature Review, Study Selection, and Data Extraction

Literature searches were conducted in BIOSIS, Embase, PsycInfo, PubMed, Scopus, Web of Science, CNKI, and Wanfang. The searches were performed through October 2023 without language restrictions.^13^ Studies were independently screened by 2 reviewers against inclusion and exclusion criteria described in the “Detailed Methods” section of eAppendix 1 in Supplement 1 and the protocol.^13^ Data were extracted from included studies by 1 extractor and verified by a second extractor into the Health Assessment Workspace Collaborative (HAWC) system. Data are publicly available and downloadable (https://hawcproject.org/assessment/405/).

### Quality Assessment: Risk of Bias

Quality of individual studies, also called risk of bias, was independently evaluated by 2 trained assessors following criteria prespecified in the protocol,^13^ using the National Toxicology Program’s or Division of Translational Toxicology’s OHAT approach.^14^ Risk-of-bias questions concerning confounding, exposure characterization, and outcome assessment were considered key. If not addressed appropriately, these questions were thought to have the greatest potential impact on the results.^13^ The remaining risk-of-bias questions were used to identify other concerns that may indicate serious risk-of-bias issues (eg, selection bias, inappropriate statistical analysis). No study was excluded from the meta-analysis based on concerns for risk of bias; however, subgroup analyses were conducted with and without high risk-of-bias studies (ie, studies rated probably high risk of bias for ≥2 key risk-of-bias questions or definitely high risk of bias for any single question) to assess their potential impact, in terms of magnitude and direction of bias, on the results. Ratings and justification are available in HAWC (https://hawcproject.org/assessment/405/).

### Statistical Analysis

We conducted the following analyses, planned a priori in the protocol: (1) mean-effects meta-analysis, (2) dose-response mean-effects meta-analysis, and (3) regression slopes meta-analysis (detailed methods are provided in the “Detailed Methods” section of eAppendix 1 in Supplement 1).

The mean-effects meta-analysis included studies that reported mean IQ scores and group-level exposures for at least 1 exposed group and 1 reference group. The effect estimates were standardized mean differences (SMDs) for heteroscedastic population variances.^15,16,17^ SMDs were calculated from the difference in mean IQ scores between an exposed group and a reference group. If an individual study reported mean IQ scores for multiple exposure groups, the highest exposure group was considered the exposed group and the lowest exposure group was considered the reference group. A sensitivity analysis was performed to evaluate the impact of all exposure groups combined compared with a reference group. Pooled SMDs and 95% CIs were estimated using random-effects models. To determine whether the data support an exposure-response association, we conducted a dose-response mean-effects meta-analysis that included studies from the mean-effects meta-analysis and used a 1-step approach as described in the protocol.^13,18,19,20^ A pooled dose-response curve was estimated using a restricted maximum likelihood estimation method. Potential nonlinear associations were examined using quadratic terms and restricted cubic splines. Model comparison was based on the maximum likelihood Akaike information criterion (AIC).^21^ To examine associations at lower fluoride levels, subgroup analyses were restricted to 0 to less than 4 mg/L (comparable to EPA’s enforceable drinking water standard for fluoride of ≤4 mg/L), 0 to less than 2 mg/L (comparable to EPA’s nonenforceable standard for fluoride in drinking water of ≤2 mg/L), and 0 to less than 1.5 mg/L (comparable to WHO’s guideline for fluoride in drinking water of ≤1.5 mg/L).^4^

The regression slopes meta-analysis included studies that reported regression slopes to estimate associations between individual-level fluoride exposures and children’s IQ. Data from individual studies were pooled using a random-effects model.^22^

Heterogeneity was assessed by Cochran *Q* test^23^ and the *I*^2^ statistic.^24^ Subgroup analyses stratified studies by risk of bias (high or low), study location (country), outcome assessment, exposure matrix (eg, urine, water), sex, and age to further investigate sources of heterogeneity. An analysis stratified by prenatal or postnatal exposure was suggested post hoc. Potential publication bias was assessed with funnel plots and Egger tests.^25,26,27^ If publication bias was present, trim-and-fill methods^28,29^ were used to estimate the number of hypothetical “missing” studies and predict the impact of the missing studies on the pooled effect estimate.

Statistical analyses were performed using Stata version 17.0 statistical software (StataCorp LLC).^30^ The combine, meta esize, meta set, meta summarize, drmeta, meta funnel, meta bias, meta trimfill, and metareg packages were used.^31^

## Results

### Study Sample

A total of 74 publications (64 cross-sectional studies and 10 prospective cohort studies) met the inclusion criteria, with 65 included in the primary analyses and an additional 9 included in sensitivity analyses (eFigure 1B in Supplement 1; see eTable 2 in Supplement 1 for excluded publications). Characteristics of the 74 publications and the study-specific effect estimates used in the meta-analyses are shown in eTable 1 in Supplement 1. Most studies were conducted in China (n = 45); other locations included Canada (n = 3), Denmark (n = 1), India (n = 12), Iran (n = 4), Mexico (n = 4), New Zealand (n = 1), Pakistan (n = 2), Spain (n = 1), and Taiwan (n = 1). No studies were conducted in the United States. Of these, 59 publications reported mean IQ scores for group-level exposures^10,11,32,33,34,35,36,37,38,39,40,41,42,43,44,45,46,47,48,49,50,51,52,53,54,55,56,57,58,59,60,61,62,63,64,65,66,67,68,69,70,71,72,73,74,75,76,77,78,79,80,81,82,83,84,85,86,87,88,89,90,91,92,93,94,95^ and 19 reported regression slopes for individual-level exposures based on urinary or water fluoride concentrations and fluoride intake.^9,10,11,32,33,34,35,36,37,38,96,97,98,99,100,101,102,103,104^ Additional details on study characteristics are provided in the “Results” section of eAppendix 1 in Supplement 1. Sixty-four studies reported inverse associations between fluoride exposure measures and children’s IQ. Fifty-two studies were rated high risk of bias. Twenty-two studies were rated low risk of bias, with 13 rated low risk of bias across all 7 risk-of-bias domains and 9 rated low risk of bias in 6 domains and probably high risk of bias in no more than 1 domain. Results from risk-of-bias evaluations are presented in eFigure 2 in Supplement 1. Interactive versions of the figures and risk-of-bias evaluations are available in HAWC (links provided in the “Results” section of eAppendix 1 in Supplement 1). Further details and justification about low risk-of-bias studies are presented in eAppendix 2 in Supplement 1.

### Mean-Effects Meta-Analysis

**Figure 1.**
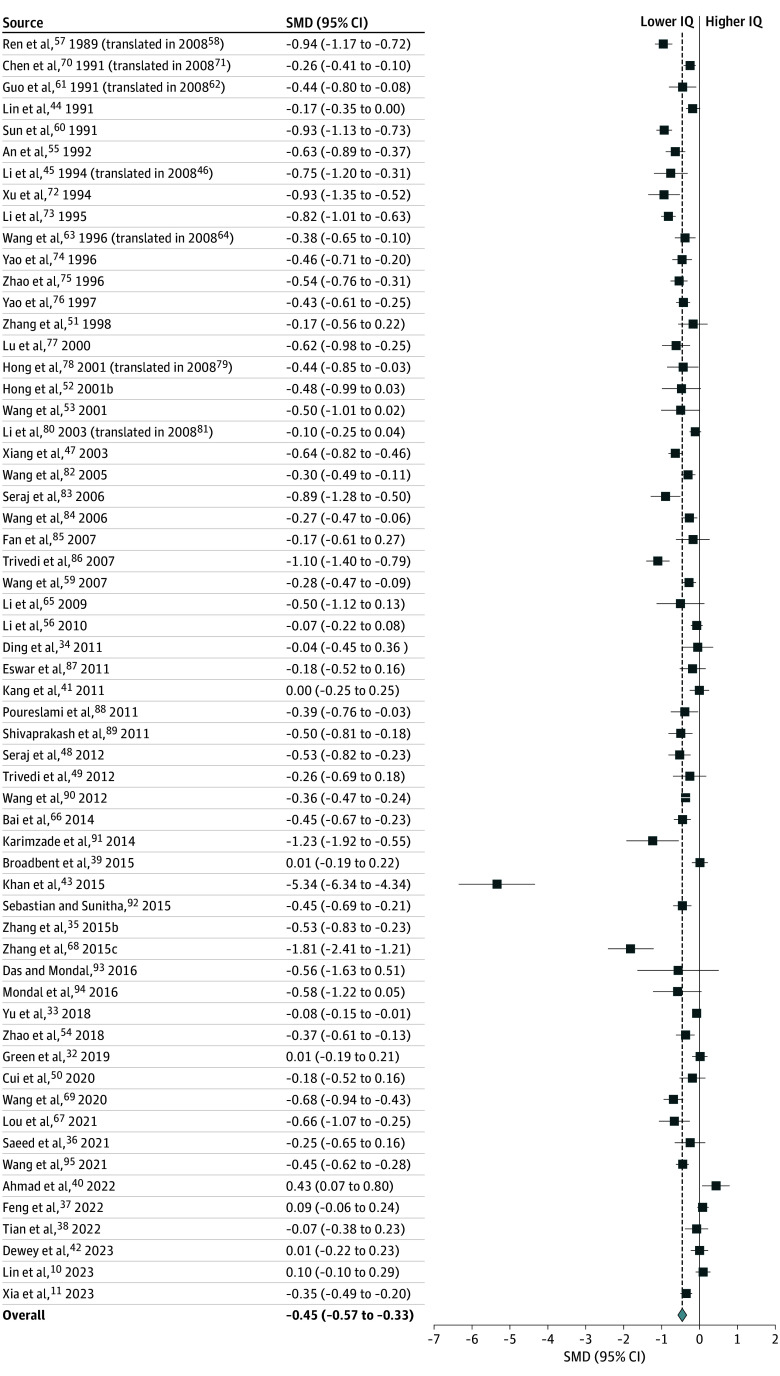
Forest Plot for Random-Effects Meta-Analysis of Standardized Mean Differences (SMDs) of the Association Between Group-Level Measures of Fluoride Exposure and IQ Scores in Children Effect size is expressed as the standardized weighted mean difference for heteroscedastic population variances (SMD). The random-effects pooled SMD is shown as a diamond. Error bars represent 95% CIs for the study-specific SMDs. Studies are presented in chronological order as found in eTable 1 in Supplement 1.

The meta-analysis of 59 studies (47 high risk of bias, 12 low risk of bias; n = 20 932 children) that provided mean IQ scores showed that, when compared with children exposed to lower fluoride levels, children exposed to higher fluoride levels had statistically significantly lower IQ scores (random-effects pooled SMD, −0.45; 95% CI, −0.57 to −0.33; *P* < .001) (Table 1 and Figure 1). There was evidence of high heterogeneity (*I*^2^ = 94%; *P* < .001; Table 1) and publication bias (funnel plot and Egger *P* < .001, Begg *P* = .03; eFigures 3 and 4 in Supplement 1). Adjusting for possible publication bias through trim-and-fill analysis supported the statistically significant inverse association after imputation of 2 additional studies to the right side (adjusted SMD, –0.39; 95% CI, −0.58 to −0.20) or 17 studies to the left side (adjusted SMD, –0.63; 95% CI, –0.76 to –0.50) (eFigures 5 and 6 in Supplement 1). Fifty-two of the 59 studies (88%) reported an inverse association with SMDs ranging from −5.34 (95% CI, −6.34 to −4.34) to −0.04 (95% CI, −0.45 to 0.36) (Figure 1). Seven studies that did not report inverse associations reported SMDs ranging from 0.00 (95% CI, −0.25 to 0.25) to 0.43 (95% CI, 0.07 to 0.80).^10,32,37,39,40,41,42^ Three studies^43,44,45^ lacked clear descriptions of their intelligence assessment methods; however, sensitivity analyses did not reveal substantial changes in the pooled SMD estimate when these studies were excluded or when a study^103^ that reported the cognitive subset of evaluations using Bayley and McCarthy tests was included (eTable 3 in Supplement 1).

**Table 1.** Pooled Standardized Mean Differences (SMDs) From Random-Effects Meta-Analyses of the Association Between Group-Level Measures of Fluoride Exposure and IQ Scores in Children

Analysis	Studies, No.	SMD (95% CI)	Heterogeneity	
			*P* value	*I*^2^, %
Overall association with IQ	59	−0.45 (−0.57 to −0.33)	<.001	94
Subgroup analysis				
Risk of bias				
Low	12	−0.19 (−0.35 to −0.04)	.01	87
High	47	−0.52 (−0.68 to −0.37)	<.001	94
Sex				
Female	15	−0.45 (−0.65 to −0.25)	<.001	78
Male	16	−0.53 (−0.77 to −0.29)	<.001	88
Age, y				
<10^a^	14	−0.38 (−0.57 to −0.19)	<.001	82
≥10	17	−0.52 (−0.67 to −0.37)	<.001	71
Country				
China	41	−0.42 (−0.51 to −0.33)	<.001	86
India	8	−1.09 (−2.23 to 0.06)	<.001	98
Iran	4	−0.68 (−0.99 to −0.38)	.08	57
Canada	2	0.01 (−0.14 to 0.16)	NA	0
Pakistan	2	0.10 (−0.57 to 0.77)	.01	83
New Zealand	1	0.01 (−0.19 to 0.22)	NA	NA
Taiwan	1	0.10 (−0.10 to 0.29)	NA	NA
Assessment type				
CRT-RC	31	−0.35 (−0.45 to −0.25)	<.001	85
Non–CRT-RC tests	28	−0.59 (−0.88 to −0.29)	<.001	96
Raven tests	12	−0.72 (−1.48 to 0.04)	<.001	99
Other tests	16	−0.52 (−0.72 to −0.32)	<.001	87
Exposure matrix				
Water fluoride	34	−0.35 (−0.45 to −0.24)	<.001	87
Dental fluorosis	9	−0.86 (−1.91 to 0.19)	.11	99
Other exposures^b^	16	−0.54 (−0.71 to −0.37)	<.001	81

Abbreviations: CRT-RC, Combined Raven Test–The Rural Edition in China; NA, not applicable.

^a^ Both An et al^55^ and Li et al^56^ included 10-year-old children in the group listed as younger than 10 years (ages 7-10 years reported).

^b^ Includes iodine,^44,52,53,54,57,58^ arsenic,^36,51,59^ aluminum,^60^ and non–drinking water fluoride (ie, fluoride from coal burning^61,62,63,64,65,66,67,68,69^).

Among the low risk-of-bias studies,^10,11,32,33,34,35,37,42,47,48,49,50^ the random-effects pooled SMD was −0.19 (95% CI, −0.35 to −0.04; *P* = .01) with high heterogeneity (*I*^2^ = 87%) (Table 1; eFigure 7 in Supplement 1) and no evidence of publication bias (funnel plot and Egger *P* = .56; eFigures 8 and 9 in Supplement 1). Among the high risk-of-bias studies, the random-effects pooled SMD was −0.52 (95% CI, −0.68 to −0.37; *P* < .001) with high heterogeneity (*I*^2^ = 94%) (Table 1; eFigure 7 in Supplement 1). There was evidence of publication bias (funnel plot and Egger *P* < .001; eFigures 8 and 9 in Supplement 1); the trim-and-fill analysis had an adjusted pooled SMD of −0.47 (95% CI, −0.72 to −0.23) (eFigures 10 and 11 in Supplement 1).

Subgroup analyses by sex, age, study location, outcome assessment type, and exposure assessment matrix found inverse associations between measures of fluoride exposure and children’s IQ (Table 1; eFigures 12-16 in Supplement 1). The subgroup analyses did not explain a large amount of the overall heterogeneity; however, the degree of heterogeneity was lower for studies restricted to Iran (*I*^2^ = 57%), children aged 10 years or older (*I*^2^ = 71%), and girls (*I*^2^ = 78%) (“Results” section of eAppendix 1 in Supplement 1). The results of the metaregression models indicate that year of publication and mean age of children did not explain a large degree of heterogeneity (“Results” section of eAppendix 1 in Supplement 1).

### Dose-Response Mean-Effects Meta-Analysis

The dose-response mean-effects meta-analysis included data from 38 studies (eTable 1 in Supplement 1). We excluded studies for which there was evidence that coexposures to arsenic or iodine might be differential.^36,41,44,51,52,53,54,105^ Results from both the analysis of 31 studies with group-level fluoride measurements in drinking water (24 high risk of bias, 7 low risk of bias; n = 12 487 children) and the analysis of 20 studies with group-level mean urinary fluoride levels (10 high risk of bias, 10 low risk of bias; n = 9756 children) found that lower children’s IQ scores were associated with increasing levels of fluoride exposure. Based on the linear models, the mean SMD between exposed and reference groups was −0.15 (95% CI, −0.20 to −0.11; *P* < .001) for water fluoride levels and −0.15 (95% CI, −0.23 to −0.07; *P* < .001) for urinary fluoride levels (Table 2; eTable 4 in Supplement 1). Based on the AIC, the best model fit was achieved when restricted cubic spline levels were added to the linear models for drinking water. Given the small difference in AICs between the different models, and considerations of parsimony and ease of interpretability, the linear model results were chosen for the purposes of discussion and are presented in Table 2, although results from all models are presented in eTable 4 in Supplement 1. For fluoride in water, the associations remained inverse when exposed groups were restricted to less than 4 mg/L (16 high risk-of-bias studies, 7 low risk-of-bias studies) or less than 2 mg/L (4 high risk-of-bias studies, 4 low risk-of-bias studies); however, the association was null at less than 1.5 mg/L (4 high risk-of-bias studies, 3 low risk-of-bias studies) (Table 2; eTable 4 in Supplement 1). When we included only studies with low risk of bias, the associations remained inverse at less than 4 mg/L, less than 2 mg/L, and less than 1.5 mg/L fluoride in water, and the linear model was the best fit (Table 2; eTable 4 in Supplement 1). For urinary fluoride, the associations remained inverse when exposed groups were restricted to less than 4 mg/L (4 high risk-of-bias studies, 10 low risk-of-bias studies), less than 2 mg/L (2 high risk-of-bias studies, 4 low risk-of-bias studies), and less than 1.5 mg/L (1 high risk-of bias study, 4 low risk-of-bias studies). When we included only the low risk-of-bias studies, the associations remained inverse at less than 4 mg/L, less than 2 mg/L, and less than 1.5 mg/L for urinary fluoride, and the linear model was the best fit (Table 2; eTable 4 in Supplement 1).

**Table 2.** Pooled Changes in Standardized Mean Differences (SMDs) From the Linear Model From the Dose-Response Mean-Effects Meta-Analyses Using Group-Level Measures of Fluoride Exposure

Fluoride exposure, mg/L	Studies, No.	Effect estimates, No.^a^	Children, No.	Parameter estimates^b^	
				β (95% CI)	*P* value
**Water fluoride, all studies**					
All data	31	41	12 487	−0.15 (−0.20 to −0.11)	<.001
<4	23	29	9554	−0.22 (−0.27 to −0.17)	<.001
<2	8	10	3682	−0.18 (−0.40 to 0.03)	.10
<1.5	7	7	2832	0.05 (−0.36 to 0.45)	.82
**Water fluoride, low risk-of-bias studies**					
All data	7	12	5066	−0.21 (−0.33 to −0.09)	.001
<4	7	10	4962	−0.23 (−0.34 to −0.11)	<.001
<2	4	5	1632	−0.33 (−0.53 to −0.13)	.001
<1.5	3	3	879	−0.32 (−0.91 to 0.26)	.28
**Urinary fluoride, all studies**					
All data	20	32	9756	−0.15 (−0.23 to −0.07)	<.001
<4	14	25	8019	−0.20 (−0.31 to −0.08)	.001
<2	6	10	4692	−0.08 (−0.15 to −0.005)	.04
<1.5	5	8	4219	−0.08 (−0.15 to −0.003)	.04
**Urinary fluoride, low risk-of-bias studies**					
All data	10	14	6847	−0.13 (−0.23 to −0.03)	.01
<4	10	14	6847	−0.13 (−0.23 to −0.03)	.01
<2	4	7	4179	−0.08 (−0.15 to −0.002)	.04
<1.5	4	7	4179	−0.08 (−0.15 to −0.002)	.04

^a^ This represents the number of effect estimates (SMDs) from all the studies included in the analysis. Studies with more than 2 exposure levels provided more than 1 SMD for inclusion in the dose-response meta-analysis.

^b^ Parameter estimates are changes in SMDs (β [95% CI]) for the linear model based on the restricted maximum likelihood models.

### Regression Slopes Meta-Analysis

Each of the 19 studies with individual-level fluoride measures (2 high risk-of-bias studies, 17 low risk-of-bias studies) (eTable 1 in Supplement 1) reported urinary fluoride levels,^9,10,11,32,33,34,35,36,37,38,96,97,98,99,100,101,102,103,104^ 2 reported fluoride intake,^32,97^ and 2 reported water fluoride levels.^32,33^ Thirteen studies were included in the primary regression slopes meta-analysis. The 6 remaining studies, including 3 studies^96,97,98^ with populations that overlapped with already-included studies^32,33,101^ and 3 that reported scores based on Bayley assessments,^102,103,104^ were included in sensitivity analyses (eTable 5 in Supplement 1).

**Figure 2.**
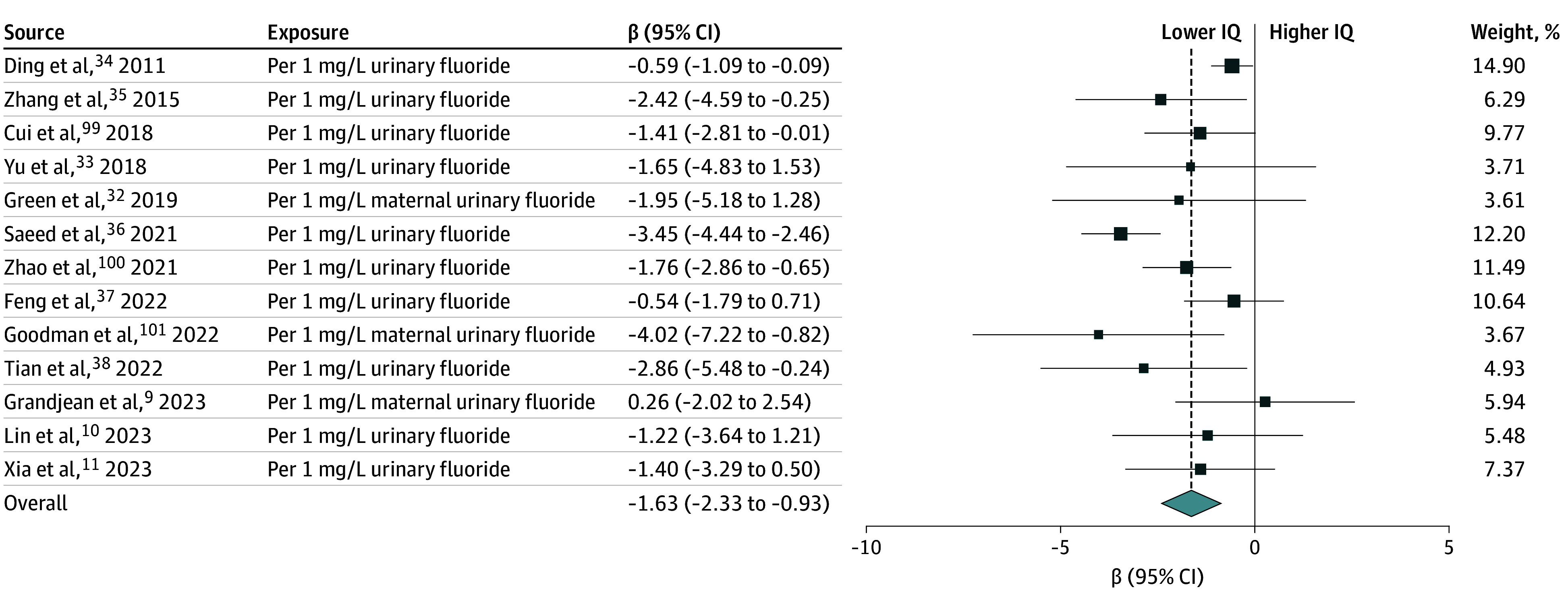
Forest Plot for Random-Effects Meta-Analysis of Regression Slopes of the Association Between Individual-Level Urinary Fluoride Measures and IQ Scores in Children The effect measures are regression slopes (β) per 1-mg/L increase in urinary fluoride. The βs for individual studies are shown with boxes representing the weight, and the pooled estimate is shown as a diamond. Error bars represent 95% CIs for the study-specific βs. Studies are presented in chronological order as found in eTable 1 in Supplement 1.

In the primary regression slopes meta-analysis, the pooled effect estimate from the 13 studies (2 high risk-of-bias studies, 11 low risk-of-bias studies; n = 4475 children) with individual-level data showed that a 1-mg/L increase in urinary fluoride was associated with a statistically significant decrease in IQ score of 1.63 points (95% CI, −2.33 to −0.93; *P* < .001) (Figure 2) with evidence of heterogeneity (*I*^2^ = 60%; *P* < .001; Table 3) and no indications of publication bias (eFigures 17 and 18 in Supplement 1). When restricted to low risk-of-bias studies, the decrease in IQ score was 1.14 points (95% CI, −1.68 to −0.61; *P* < .001) with evidence of low heterogeneity (*I*^2^ = 23%; *P* = .28; Table 3; eFigure 19 in Supplement 1) and a slight indication of publication bias (eFigure 20 in Supplement 1). The trim-and-fill analysis had an adjusted estimate of −0.78 (95% CI, −1.33 to −0.22) (eFigures 21 and 22 in Supplement 1).

**Table 3.** Pooled Changes in IQ Scores From Random-Effects Meta-Analyses of the Association Between Individual-Level Measures of Urinary Fluoride and IQ Scores in Children

Analysis	Studies, No.	β (95% CI)	Heterogeneity	
			*P* value	*I*^2^, %
Overall association with IQ	13	−1.63 (−2.33 to −0.93)	<.001	60
Subgroup analysis				
Risk of bias				
Low	11	−1.14 (−1.68 to −0.61)	.28	23
High	2	−3.38 (−4.30 to −2.45)	.68	0
Sex				
Female	3	−1.07 (−2.20 to 0.05)	.33	0
Male	3	−1.21 (−3.80 to 1.37)	.09	65
Country				
Canada	1	−1.95 (−5.19 to 1.28)	NA	NA
China	8	−1.20 (−1.79 to −0.61)	.25	31
Denmark	1	0.26 (−2.02 to 2.54)	NA	NA
Mexico	1	−4.02 (−7.22 to −0.82)	NA	NA
Pakistan	1	−3.45 (−4.44 to −2.46)	NA	NA
Taiwan	1	−1.22 (−3.64 to 1.21)	NA	NA
Assessment type				
CRT-RC	9	−1.19 (−1.75 to −0.63)	.34	27
Non-CRT-RC tests	4	−2.32 (−4.26 to −0.37)	.03	66
Exposure matrix				
Urinary fluoride	13	−1.63 (−2.33 to −0.93)	<.001	60
Intake	2	−3.87 (−7.15 to −0.59)	.74	0
Water fluoride	2	−4.77 (−9.09 to −0.45)	.71	0
Exposure timing				
Prenatal	3	−1.70 (−4.23 to 0.84)	.09	57
Postnatal	10	−1.65 (−2.39 to −0.90)	<.001	64

Abbreviations: CRT-RC, Combined Raven Test–The Rural Edition in China; NA, not applicable.

Subgroup analyses by risk of bias, sex, country, exposure matrix, outcome assessment type, and prenatal or postnatal exposure found inverse associations between measures of fluoride exposure and children’s IQ (Table 3; eFigures 23-27 in Supplement 1). The sensitivity analyses including reporting scores based on Bayley assessments^102,103,104^ showed no substantial changes in the pooled effect estimates (eTable 5 in Supplement 1).

## Discussion

This systematic review and meta-analysis found statistically significant inverse associations between measures of fluoride exposure and children’s IQ. These inverse associations were observed in all 3 sets of meta-analyses: the mean-effects meta-analysis (47 high risk-of-bias studies, 12 low risk-of-bias studies) and dose-response mean-effects meta-analysis (27 high risk-of-bias studies, 11 low risk-of-bias studies) of group-level fluoride exposure, and the regression slopes meta-analysis (2 high risk-of-bias studies, 11 low risk-of-bias studies) of individual-level urinary fluoride. Within each of these meta-analyses, we used prespecified criteria to assess study quality and classify studies into low and high risk of bias. Stratified analyses found similar inverse associations in both study quality strata. Further subgroup analyses by sex, age, timing of exposure, study location, outcome assessment type, and exposure assessment matrix also found inverse associations between fluoride exposure and children’s IQ.

Studies in these meta-analyses included cross-sectional and prospective cohort designs, each study having its own strengths and limitations. Although all studies contribute to our understanding of the overall association, well-designed studies that accurately measure exposure and outcome and adequately account for potential confounding variables are particularly informative. In these meta-analyses, we followed the OHAT approach^14^ to extract data from each of the published studies and to classify studies into high risk of bias and low risk of bias based on carefully predefined criteria.^13^ To make our process and decisions transparent, we provide full public access to the extracted data, risk-of-bias ratings, and rationale for those ratings for each individual study. These data can be used by other investigators to evaluate or extend our process and analysis (https://hawcproject.org/assessment/405/).

Studies using group-level exposures were assessed in the mean-effects meta-analysis. An advantage of such studies is that they can, for example, examine communities with different CWS fluoride levels. Although in the United States 40% to 70% of a person’s fluoride intake comes from fluoridated drinking water, there are other sources of fluoride exposure.^4^ Therefore, relying on CWS levels alone may underestimate an individual’s total fluoride exposure, which may vary considerably among members of a group depending on individual behaviors. Most of the studies in the mean-effects meta-analysis were cross-sectional; however, we have higher confidence in cross-sectional studies when there is evidence of temporality.^14^ Among the low risk-of-bias cross-sectional studies, most provided information to establish that exposure likely preceded the outcome (eg, only including children who had lived in a community since birth or children who had dental fluorosis).

Studies using individual-level exposures were assessed in the regression slopes meta-analysis, which included 13 studies with urinary fluoride measures, a more precise exposure assessment measure than group-level exposures. Unlike drinking water levels, individual-level urinary fluoride concentrations include all ingested fluoride and are considered a valid estimate of total fluoride exposure.^106,107^ Fluoride in urine is measured from both single or spot samples and multiple collections. When compared with 24-hour urine samples, spot samples are more prone to the influence of timing of exposure and can be affected by differences in dilution. However, correlations between urinary fluoride concentrations from 24-hour samples and spot samples adjusted for urinary dilution have been described.^108^ There were several recent North American prospective cohort studies conducted in Canada and Mexico^32,96,97,101^ that reported maternal urinary fluoride levels comparable to those in the United States.^109,110^ These studies combined multiple urinary measurements over the course of pregnancy to examine prenatal fluoride exposure during a critical period of brain development. Although the estimated decreases in IQ found in the regression slopes meta-analysis may seem small (1.63 IQ points per 1-mg/L increase in urinary fluoride), research on other neurotoxicants has shown that subtle shifts in IQ at the population level can affect people who fall within the high and low ranges of the population’s IQ distribution.^111,112,113,114,115^ For context, a 5-point decrease in a population’s IQ would nearly double the number of people classified as intellectually disabled.^116^

Finally, studies with group-level exposure measurements were used in the dose-response mean-effects meta-analysis of water or urinary fluoride levels. Although we examined 2 nonlinear models, a linear model almost always provided the best fit for both water and urinary data. There was a statistically significant dose-response association between group-level fluoride measures and children’s IQ. In stratified analyses of low risk-of-bias studies, the association remained inverse when exposure was restricted to less than 4 mg/L, less than 2 mg/L, and less than 1.5 mg/L fluoride in water or urine; except for fluoride concentrations less than 1.5 mg/L in water, these results were statistically significant. There was some inconsistency in the best-fit model and a lack of statistical significance at lower levels for water fluoride exposures, leading to uncertainty in the shape of the dose-response curve. This uncertainty is not surprising given the lower number of observations for fluoride concentrations in water (n = 879 from 3 studies) compared with urinary fluoride concentrations (n = 4218 from 5 studies). The ability to detect a true effect is reduced at lower exposure levels when exposure contrasts are diminished.^117^ Although the same cutoffs were used for the water and urine subgroup analyses, fluoride levels in water likely underestimate total fluoride exposures that are better estimated by levels in urine. Variable fluoride exposures from nonwater sources may also decrease the precision of the effect estimates at lower fluoride concentrations in water. In contrast, the best model fit for urinary fluoride concentrations was consistently linear.

Elevated naturally occurring fluoride levels in groundwater (>1.5 mg/L) are prevalent globally and include central Australia, eastern Brazil, sub-Saharan Africa, the southern Arabian Peninsula, south and east Asia, and western North America.^118^ Although to our knowledge no epidemiological studies addressing fluoride exposure and children’s IQ have been conducted in the United States, significant inequalities in CWS fluoride levels exist by county sociodemographic characteristics, including racial and ethnic composition, especially among Hispanic and Latino communities.^119^ Of note, there are regions of the United States where CWS and private wells contain natural fluoride concentrations greater than 1.5 mg/L,^120^ serving more than 2.9 million US residents.^119^ In addition, the US Geological Survey estimates that 172 000 US residents are served by domestic wells that exceed EPA’s enforceable standard of 4.0 mg/L fluoride in drinking water, and 522 000 are served by domestic wells that exceed EPA’s nonenforceable standard of 2.0 mg/L fluoride in drinking water.^1^ To reduce risk of moderate-to-severe dental fluorosis, the CDC recommends that parents use an alternative source of water for children aged 8 years or younger and for bottle-fed infants if their primary drinking water contains greater than 2 mg/L of fluoride.^121^ Currently, there are no recommendations or restrictions on fluoride levels in drinking water based on cognitive neurodevelopmental outcomes.^121^

To our knowledge, no studies of fluoride exposure and children’s IQ have been performed in the United States, and no nationally representative urinary fluoride levels are available, hindering application of these findings to the US population. Although this meta-analysis was not designed to address the broader public health implications of water fluoridation in the United States, these results may inform future public health risk-benefit assessments of fluoride.

### Strengths and Limitations

Strengths of this systematic review and meta-analysis include a large body of literature, a predefined systematic search and screening process, risk-of-bias assessment of individual studies, prespecified subgroup analyses, and use of both group-level and individual-level exposure data. The consistency of the inverse associations across the high and low risk-of-bias studies, different intelligence assessment methods, different exposure matrices, different study locations, different analytical approaches, and evidence of a dose-response association strengthen confidence in the conclusion of an overall inverse association between fluoride exposure and children’s IQ. It is notable that there is a diversity of study design factors across studies, which could be described as overall heterogeneity of the body of evidence. In this case, the heterogeneity supports the robustness of the conclusions and is different from heterogeneity in the results, which we did not find in this meta-analysis.

The body of existing literature has limitations in that many of the studies were classified as having high risk of bias. Most of the studies included in the mean-effects and dose-response mean-effects meta-analyses were cross-sectional and had study design and/or methodological limitations. However, the consistency in meta-analytic associations across the high and low risk-of-bias studies and the other subgroup analyses reduced the likelihood that specific biases or potential confounders in individual studies could explain the inverse association between fluoride exposure and children’s IQ.

While several recent studies conclude that fluoride exposures from community water fluoridation are not associated with children’s IQ or other neurodevelopmental outcomes,^122,123,124^ the results of the mean-effects meta-analysis were consistent with 6 previous meta-analyses^6,7,8,122,125,126^ that reported statistically significant inverse associations between fluoride exposure and children’s IQ scores (see the “Characteristics of Previous Meta-Analyses” section of eAppendix 1 and eTable 6 in Supplement 1).

## Conclusions

This meta-analysis found inverse associations and an inverse dose-response association between fluoride exposure and children’s IQ across the multicountry epidemiological literature. There were limited data and uncertainty in the dose-response association between fluoride exposure and children’s IQ when fluoride exposure was estimated by drinking water alone at concentrations less than 1.5 mg/L. Confidence in the associations at lower fluoride levels could be increased by additional prospective cohort studies with individual fluoride exposure measures. These results may inform future comprehensive public health risk-benefit assessments of fluoride.

## Notes

Supplement 1. **eAppendix 1.** Detailed Methods, Results, and Characteristics of Previous Meta-Analyses **eTable 1.** Characteristics of Studies Included in the Primary and Subgroup Analyses for the Mean-Effects, Dose-Response, and Regression-Slopes Meta-Analyses **eTable 2.** List of Excluded Studies From Mean-Effects Meta-Analysis **eTable 3.** Sensitivity Analyses for Mean-Effects Meta-Analysis: Pooled SMDs and 95% CIs for Children’s IQ Score and Exposures to Fluoride **eTable 4.** Dose-Response Meta-Analysis Using Mean Effects—Restricted Maximum Likelihood Models **eTable 5.** Regression Slopes and 95% CIs From Random Effects Meta-Analysis of the Association Between Individual-Level Measures of Urinary Fluoride and IQ Scores in Children: Sensitivity Analyses **eTable 6.** Previous Meta-Analyses on Exposures to Fluoride and Children’s IQ **eFigure 1.** Information on Study Location by Year and Reference Flow Diagram **eFigure 2.** Results from Risk-of-bias Evaluations for Studies Included in the Meta-Analyses and Sensitivity Analyses **eFigure 3.** Funnel Plot of Included Studies **eFigure 4.** Test for Publication Bias **eFigure 5.** Trim-and-Fill Analysis **eFigure 6.** Filled-In Funnel Plots to Eliminate Publication Bias **eFigure 7.** Association Between Fluoride Exposure and IQ Scores in Children: Effect by Risk of Bias **eFigure 8.** Funnel Plot by Risk-of-Bias Evaluation **eFigure 9.** Test for Publication Bias by Risk of Bias **eFigure 10.** Trim-and-Fill Analysis for High Risk-of-Bias Studies **eFigure 11.** Filled-In Funnel Plots for High Risk-of-Bias Studies **eFigure 12.** Association Between Fluoride Exposure and IQ Scores in Children: Effect by Sex **eFigure 13.** Association Between Fluoride Exposure and IQ Scores in Children: Effect by Age Group **eFigure 14.** Association Between Fluoride Exposure and IQ Scores in Children: Effect by Country **eFigure 15.** Association Between Fluoride Exposure and IQ Scores in Children: Effect by Outcome Assessment Type **eFigure 16.** Association Between Fluoride Exposure and IQ Scores in Children: Effect by Exposure Matrix **eFigure 17.** Funnel Plot for Studies With Individual-Level Exposures **eFigure 18.** Test for Publication Bias for Studies With Individual-Level Exposures **eFigure 19.** Association Between Individual-Level Fluoride Exposure and IQ Scores in Children: Effect by Risk of Bias **eFigure 20.** Test for Publication Bias for Low Risk-of-Bias Studies With Individual-Level Exposures **eFigure 21.** Trim-and-Fill Analysis for Low Risk-of-Bias Studies **eFigure 22.** Filled-In Funnel Plots for Low Risk-of-Bias Studies **eFigure 23.** Association Between Individual-Level Fluoride Exposure and IQ Scores in Children: Effect by Exposure Matrix **eFigure 24.** Association Between Individual-Level Fluoride Exposure and IQ Scores in Children: Effect by Country **eFigure 25.** Association Between Individual-Level Fluoride Exposure and IQ Scores in Children: Effect by Assessment Type **eFigure 26.** Association Between Individual-Level Fluoride Exposure and IQ Scores in Children: Effect by Sex **eFigure 27.** Association Between Individual-Level Fluoride Exposure and IQ Scores in Children: Effect by Prenatal vs. Postnatal Exposure **eAppendix 2.** Details for Low Risk-of-Bias Studies **eAppendix 3.** Contributors to Fluoride Exposure and Children’s IQ Scores: A Systematic Review and Meta-Analysis

Supplement 2. Data Sharing Statement
