# Supplementary material for: Fluoride Exposure and Children’s IQ Scores: A Systematic Review and Meta-Analysis
Source: JAMA Pediatr. 2025 Jan 6;179(3):282–92. doi: 10.1001/jamapediatrics.2024.5542 (PMC11877182; doi:10.1001/jamapediatrics.2024.5542)
Supplement: Supplement 1. — eAppendix 1. Detailed Methods, Results, and Characteristics of Previous Meta-Analyses eTable 1. Characteristics of Studies Included in the Primary and Subgroup Analyses for the Mean-Effects, Dose-Response, and Regression-Slopes Meta-Analyses eTable 2. List of Excluded Studies From Mean-Effects Meta-Analysis eTable 3. Sensitivity Analyses for Mean-Effects Meta-Analysis: Pooled SMDs and 95% CIs for Children’s IQ Score and Exposures to Fluoride eTable 4. Dose-Response Meta-Analysis Using Mean Effects—Restricted Maximum Likelihood Models eTable 5. Regression Slopes and 95% CIs From Random Effects Meta-Analysis of the Association Between Individual-Level Measures of Urinary Fluoride and IQ Scores in Children: Sensitivity Analyses eTable 6. Previous Meta-Analyses on Exposures to Fluoride and Children’s IQ eFigure 1. Information on Study Location by Year and Reference Flow Diagram eFigure 2. Results from Risk-of-bias Evaluations for Studies Included in the Meta-Analyses and Sensitivity Analyses eFigure 3. Funnel Plot of Included Studies eFigure 4. Test for Publication Bias eFigure 5. Trim-and-Fill Analysis eFigure 6. Filled-In Funnel Plots to Eliminate Publication Bias eFigure 7. Association Between Fluoride Exposure and IQ Scores in Children: Effect by Risk of Bias eFigure 8. Funnel Plot by Risk-of-Bias Evaluation eFigure 9. Test for Publication Bias by Risk of Bias eFigure 10. Trim-and-Fill Analysis for High Risk-of-Bias Studies eFigure 11. Filled-In Funnel Plots for High Risk-of-Bias Studies eFigure 12. Association Between Fluoride Exposure and IQ Scores in Children: Effect by Sex eFigure 13. Association Between Fluoride Exposure and IQ Scores in Children: Effect by Age Group eFigure 14. Association Between Fluoride Exposure and IQ Scores in Children: Effect by Country eFigure 15. Association Between Fluoride Exposure and IQ Scores in Children: Effect by Outcome Assessment Type eFigure 16. Association Between Fluoride Exposure and IQ Scores in Children: Effect by Exposu [file jamapediatr-e245542-s001.pdf]

Supplemental Online Content

Taylor KW, Eftim SE, Sibrizzi CA, et al. Fluoride exposure and children's IQ scores: a systematic review and meta-analysis. *JAMA Pediatr*. Published online January 6, 2025. doi:10.1001/jamapediatrics.2024.5542

eAppendix 1. Detailed Methods, Results, and Characteristics of Previous Meta-Analyses
eTable 1. Characteristics of Studies Included in the Primary and Subgroup Analyses for the Mean-Effects, Dose-Response, and Regression-Slopes Meta-Analyses
eTable 2. List of Excluded Studies From Mean-Effects Meta-Analysis
eTable 3. Sensitivity Analyses for Mean-Effects Meta-Analysis: Pooled SMDs and 95% CIs for Children's IQ Score and Exposures to Fluoride
eTable 4. Dose-Response Meta-Analysis Using Mean Effects—Restricted Maximum Likelihood Models
eTable 5. Regression Slopes and 95% CIs From Random Effects Meta-Analysis of the Association Between Individual-Level Measures of Urinary Fluoride and IQ Scores in Children: Sensitivity Analyses
eTable 6. Previous Meta-Analyses on Exposures to Fluoride and Children's IQ
eFigure 1. Information on Study Location by Year and Reference Flow Diagram
eFigure 2. Results From Risk-of-Bias Evaluations for Studies Included in the Meta-Analyses and Sensitivity Analyses
eFigure 3. Funnel Plot of Included Studies
eFigure 4. Test for Publication Bias
eFigure 5. Trim-and-Fill Analysis
eFigure 6. Filled-In Funnel Plots to Eliminate Publication Bias
eFigure 7. Association Between Fluoride Exposure and IQ Scores in Children: Effect by Risk of Bias
eFigure 8. Funnel Plot by Risk-of-Bias Evaluation
eFigure 9. Test for Publication Bias by Risk of Bias
eFigure 10. Trim-and-Fill Analysis for High Risk-of-Bias Studies
eFigure 11. Filled-In Funnel Plots for High Risk-of-Bias Studies
eFigure 12. Association Between Fluoride Exposure and IQ Scores in Children: Effect by Sex
eFigure 13. Association Between Fluoride Exposure and IQ Scores in Children: Effect by Age Group
eFigure 14. Association Between Fluoride Exposure and IQ Scores in Children: Effect by Country
eFigure 15. Association Between Fluoride Exposure and IQ Scores in Children: Effect by Outcome Assessment Type
eFigure 16. Association Between Fluoride Exposure and IQ Scores in Children: Effect by Exposure Matrix
eFigure 17. Funnel Plot for Studies With Individual-Level Exposures
eFigure 18. Test for Publication Bias for Studies With Individual-Level Exposures
eFigure 19. Association Between Individual-Level Fluoride Exposure and IQ Scores in Children: Effect by Risk of Bias
eFigure 20. Test for Publication Bias for Low Risk-of-Bias Studies With Individual-Level Exposures
eFigure 21. Trim-and-Fill Analysis for Low Risk-of-Bias Studies
eFigure 22. Filled-In Funnel Plots for Low Risk-of-Bias Studies
eFigure 23. Association Between Individual-Level Fluoride Exposure and IQ Scores in Children: Effect by Exposure Matrix
eFigure 24. Association Between Individual-Level Fluoride Exposure and IQ Scores in Children: Effect by Country
eFigure 25. Association Between Individual-Level Fluoride Exposure and IQ Scores in Children: Effect by Assessment Type
eFigure 26. Association Between Individual-Level Fluoride Exposure and IQ Scores in Children: Effect by Sex

© 2025 Taylor KW et al. *JAMA Pediatrics*.

eFigure 27. Association Between Individual-Level Fluoride Exposure and IQ Scores in Children: Effect by Prenatal vs. Postnatal Exposure
eAppendix 2. Details for Low Risk-of-Bias Studies
eAppendix 3. Contributors to Fluoride Exposure and Children's IQ Scores: A Systematic Review and Meta-Analysis

This supplemental material has been provided by the authors to give readers additional information about their work.

© 2025 Taylor KW et al. *JAMA Pediatrics*.

eAppendix 1. Detailed Methods, Results, and Characteristics of Previous Meta-Analyses

Detailed Methods

Systematic Literature Review

Literature searches were conducted in the following databases: BIOSIS, EMBASE, PsychINFO, PubMed, Scopus, Web of Science, CNKI, and Wanfang. Search strategies tailored for each database are available in the protocol (https://ntp.niehs.nih.gov/go/785076). The last search was performed in October 2023. The identification of studies for the meta-analysis was part of a larger systematic review.[1] The meta-analysis protocol was peer reviewed and posted to the NTP website prior to conducting the meta-analysis and review. Note that our meta-analysis protocol is not also registered in the PROSPERO database (international prospective register of systematic reviews) and at this point could not be added to PROSPERO because, starting October 2019, reviews are only accepted for registration if data extraction has not yet started (https://www.crd.york.ac.uk/prospero/#aboutregpage). The meta-analysis was part of a larger systematic review that evaluated the body of evidence on the association between fluoride exposure and children's IQ, and data extraction was already underway at the time of the meta-analysis protocol design.

Study Selection

In order to be eligible for inclusion in the systematic literature review, individual study publications (referred to in this paper as "studies") had to satisfy eligibility criteria outlined in the protocol (i.e., address PECO statement in Table 1 and specific exclusion criteria in Table 2; https://ntp.niehs.nih.gov/go/785076).[2]

These criteria included the following: (1) Outcomes were children's intelligence levels reflected by IQ scores. In the case of multiple IQ scores, the most comprehensive IQ score reported was used (e.g., Full Scale IQ score versus Performance or Verbal IQ scores for studies using the Wechsler Primary and Preschool Scale of Intelligence-III). (2) Exposures to fluoride were based on environmental measures or biomonitoring data, expressed as "exposed" versus "reference" fluoride exposure groups or as individual-level fluoride exposures. If there were more than two exposure groups, the highest level was considered the "exposed" group and the lowest level was considered the "reference" group. However, all the exposure groups reporting fluoride exposure ranges were used in the analysis. (3) Effect estimates were expressed either as a mean outcome measure for IQ levels with a measure of uncertainty (e.g., 95% confidence interval [CI], standard error [SE]) and numbers of participants for "exposed" versus "reference" fluoride exposure groups or as a regression coefficient with 95% CI or SE (or data were provided for their calculation).

The following exclusions were made:

(1) Case studies and case reports.

(2) Articles without original data (e.g., reviews, editorials, commentaries). Reference lists from these materials, however, were reviewed to identify potentially relevant studies not identified from the database searches. New studies identified were assessed for eligibility for inclusion.

(3) Conference abstracts or reports and dissertations.

References retrieved from the literature search were independently screened by two trained screeners at the title and abstract level to determine whether a reference met the evidence selection criteria. Studies

that were not excluded during the title and abstract screening were further screened for inclusion with a full-text review by two independent reviewers. Studies that estimated the association between exposure to fluoride (based on environmental measures or biomonitoring data, reported as either individual-level or group-level measurements) and a quantitative measure of children's intelligence were included. Studies that did not report quantitative effect estimates (mean outcome measures or regression coefficients), measures of variability (95% CIs, SEs, or standard deviations [SDs]), or numbers of participants were excluded. Studies with missing measures of variability but with reported p-values for differences were included, and SDs were calculated following the approach in the Cochrane Handbook for Systematic Reviews[3] To avoid sample overrepresentation, if the same cohort was followed at multiple timepoints resulting in multiple study publications,[4,5] only the study publication that included the largest number of participants was included in this meta-analysis (**eTable 2**).Translation assistance was obtained to assess the relevance of non-English studies. Following full-text review, the remaining studies were "included" and used for the evaluation. Results of the study identification process are provided in the reference flow diagram (**eFigure 1b**).

Data Extraction

Data were extracted from included studies by one extractor and verified by a second extractor. Extraction was conducted in Health Assessment Workspace Collaborative (HAWC), an open source, web-based application for data extraction elements listed in the protocol. Data extraction results for included studies are publicly available and downloadable (https://hawcproject.org/assessment/405/).

Quality Assessment: Risk-of-bias Evaluation

Quality of individual studies, also called "risk of bias," was assessed using the National Toxicology Program's OHAT approach.[6] Studies were independently evaluated by two trained assessors who answered risk-of-bias questions following prespecified criteria detailed in the protocol.[2] Risk-of-bias questions concerning confounding, exposure characterization, and outcome assessment were considered key. If not addressed appropriately, these questions were thought to have the greatest potential impact on the results.[2] The remaining risk-of-bias questions were used to identify other concerns that may indicate serious risk-of-bias issues (e.g., selection bias, inappropriate statistical analysis). No study was excluded from the meta-analysis based on concerns for risk of bias; however, subgroup analyses were conducted with and without high risk-of-bias studies (i.e., studies rated "probably high" risk of bias for at least two key risk-of-bias questions or "definitely high" for any single question) to assess their potential impact (in terms of magnitude and direction of bias) on the results.

Statistical Analysis

We conducted the following analyses, planned a priori in the protocol: (1) *mean-effects meta-analysis*, (2) *dose-response mean-effects meta-analysis*, and (3) *regression slopes meta-analysis.* We also conducted several subgroup and sensitivity analyses.

Mean-effects meta-analysis

The *mean-effects meta-analysis* included studies that reported mean IQ scores and group-level exposures for at least one exposed group and one reference group. The effect estimates in the primary *mean-effects meta-analysis* were the SMDs for heteroscedastic population variances.[7-9] SMDs were calculated from the difference in mean IQ scores between an exposed group and a reference group. If mean IQ scores were reported for multiple exposure groups within a single study, the highest exposure group was considered the exposed group and the lowest exposure group was considered the reference group. A sensitivity analysis was performed to evaluate the impact of all exposure groups combined compared to a reference group. Subgroup analyses were predefined in the protocol and were stratified by risk-of-bias (high or low), study location (country), outcome assessment type, exposure assessment matrix (e.g., urine, water),

© 2025 Taylor KW et al. *JAMA Pediatrics*.

sex, and age group. To further evaluate potential sources of heterogeneity, we conducted meta-regression analyses using mean age in years (from the age range reported in each study) and year of publication.

A sensitivity analysis was performed to evaluate the impact of using any exposed group compared to the reference group. This was accomplished by using the approach outlined in the Cochrane Handbook for Systematic Reviews[3] which combines the data from all available exposure groups (n, mean, and standard deviation [SD]). Subgroup analyses were stratified by risk of bias (high or low), outcome assessment type, exposure matrix (e.g., urine or water), pre- or post-natal exposures, outcome, gender, and age group. If results were not reported by gender or age-specific subgroups (<10, ≥10 years), they were calculated (if possible) by combining smaller subgroups. If SDs were not reported, but mean effects, sample sizes (n values), and p-values for differences between groups were available, SDs were calculated using the SE and t-statistic (assuming equal variances). To avoid sample overrepresentation, if the same cohort was followed at multiple timepoints resulting in multiple study publications (e.g., Yu et al. [2018][5] and Wang et al. [2020b][4]), only the study publication that included the largest number of participants was included in the meta-analysis (see **eTable 2** for list of excluded studies and rationales). For studies with overlapping populations (i.e., multiple study publications that used the same cohort), results from one study publication were selected considering the following factors: most appropriate exposure metric, exposure range, exposure period, number of subjects, and statistical adjustment for potential confounders (see **eTable 1** for study-specific effect estimates used in the meta-analysis).

Dose-response mean-effects meta-analysis
To determine whether the data support an exposure-response relationship, we conducted a *dose-response mean-effects meta-analysis*. This analysis included studies from the *mean-effects meta-analysis* that reported fluoride exposure levels; we excluded studies for which there was evidence that co-exposures to arsenic or iodine might be differential (**eTable 1**).

The *dose-response mean-effects meta-analysis* was conducted using a one-step approach detailed in the protocol (https://ntp.niehs.nih.gov/go/78500.76).[10-12] The approach uses linear mixed models to analyze all available mean effect estimates for the reference group and one or more of the non-reference exposure groups. For each study, the median or mean fluoride level for each exposure group was assigned to its corresponding effect estimate. If median or mean levels by exposure group were not provided, the midpoint of the upper and lower boundaries in every exposure category was assigned as the average level. If the upper boundary for the highest exposure group was not reported, the boundary was assumed to have the same amplitude as the nearest exposure category. For each study, the standardized weighted mean differences (SMDs) and corresponding standard errors (SEs) were used to compare the differences in mean IQ scores between the exposed and reference groups. The corresponding SMD for the reference group was set to zero for this analysis. The SMDs and corresponding variances were used to estimate a pooled dose-response curve using a restricted maximum likelihood estimation method (see **eTable 1** for study-specific effect estimates used in the meta-analysis). This approach avoids limitations of the traditional two-step approach that first estimates study-specific dose-response models and then pools the coefficients across studies. To examine a potential nonlinear relationship between exposure to fluoride and children's IQ levels, quadratic terms and restricted cubic splines with three knots at percentiles (10th, 50th, 90th) were created, and a potential departure from a linear trend was assessed by testing the coefficient of the quadratic term and a second spline equal to zero. Models were compared and the best model fit was determined based on the maximum likelihood Akaike information criterion (AIC).[13] The AIC is a goodness-of-fit measure that adjusts for the number of parameters in the model, and lower AIC values indicate better fitting models (**eTable 4**). To examine a potential nonlinear relationship between exposure to fluoride and children's IQ levels, quadratic terms and restricted cubic splines with three knots at percentiles (10th, 50th, 90th) were created, and a potential departure from a linear trend was assessed by testing the coefficient of the quadratic term and a second spline equal to zero. Model comparison was based on the maximum likelihood Akaike information criterion (AIC).[13] To examine whether there were

associations at lower fluoride levels, we conducted subgroup analyses for both drinking water and urinary fluoride measures. Analyses were restricted to 0 to <4 mg/L, the Environmental Protection Agency (EPA) current enforceable drinking water standard for fluoride; 0 to <2 mg/L, the EPA's non-enforceable secondary standard for fluoride in drinking water;[14] and 0 to <1.5 mg/L, the WHO guideline for fluoride in drinking water[15]. The studies included in the lower fluoride level subgroups overlap between analyses (i.e., the <4-mg/L analysis included studies from the <2-mg/L analysis and the <2-mg/L analysis included studies from the <1.5-mg/L analysis).

Regression slopes meta-analysis

The *regression slopes meta-analysis* included studies that reported regression slopes to estimate associations between individual-level fluoride exposures and children's IQ. If results from multiple models were reported within a single study, either the most adjusted results or the main model results as presented by the study authors were selected. The study outcomes were evaluated with respect to a 1-mg/L unit increase in water or urinary fluoride or a 1-mg/day increase in fluoride intake.

Data from individual studies were pooled using a random-effects model.[16] Heterogeneity was assessed by Cochran's Q test[17] and the I^2 statistic.[18] Forest plots were used to display results and to examine possible heterogeneity between studies. Potential publication bias was assessed by developing funnel plots and performing Egger regressions on the estimates of effect size.[19-21] If publication bias was present, trim-and-fill methods[22,23] were used to estimate the number of missing studies and to predict the impact of the hypothetical "missing" studies on the pooled effect estimate. Subgroup analyses were performed to investigate sources of heterogeneity. Subgroup analyses were predefined in the protocol and were stratified by risk-of-bias (high or low), study location (e.g., country), outcome assessment, exposure matrix (e.g., urine, water), sex, and age group. In addition, an analysis stratified by pre- or post-natal exposure was suggested post-hoc. The assessments of heterogeneity and publication bias were performed separately for the two different effect measures used in the analyses (i.e., SMDs for the *mean-effects meta-analysis* and *dose-response meta-analysis,* and regression slopes for the *regression slopes meta-analysis*).

Statistical analyses were performed using the software STATA version 17.0[24] with the *combine, meta esize*, *meta set*, *meta summarize*, *drmeta*, *meta funnel*, *meta bias*, *meta trimfill*, and *metareg* packages.[25]

Reporting

This manuscript follows the Meta-analysis of Observational Studies in Epidemiology (MOOSE) reporting guidelines.

Results

Study Sample

Results of the study identification process are provided in the reference flow diagram (**eFigure 1b**). Characteristics of the 59 studies that compared mean IQ scores between groups of children with different levels of fluoride exposure are shown in **eTable 1** (see **eTable 2** for list of excluded publications). Study-specific effect estimates used in the meta-analyses are also presented in **eTable 1**. One study per country was conducted in New Zealand and Taiwan; 2 studies were conducted in Pakistan and Canada; 4 studies were conducted in Iran; 8 studies were conducted in India; and the remaining 41 studies were performed in China (**eTable 1**). Nine study populations were exposed to fluoride from coal burning[26 [translated in Guo et al. 2008a], 28 [translated in Li et al. 2008b], 30-35, 36 [translated in Wang et al. 2008b]]; otherwise, it was assumed that study populations were exposed to fluoride primarily through drinking water. Measures of fluoride exposure included water fluoride (n = 34 studies), dental fluorosis (n = 9), and other non-drinking water sources of fluoride such as coal burning (n = 16). Sixteen studies presented results for boys and 15 studies reported results for girls; children <10 years old and children ≥10 years old were examined in 14 and 17 studies, respectively

(**Table 1**). The Combined Raven's Test for Rural China (CRT-RC) was used to measure children's IQ in 31 studies. Other measures of IQ included the Wechsler intelligence tests,[36 [translated in Wang et al. 2008b], 38 [translated in Ren et al. 2008], 40, 41] Binet IQ test,[26 [translated in Guo et al. 2008a], 42] Raven's Standard Progressive Matrices test,[43-52] Raymond B Cattell test,[53] Japan IQ test,[54, 55] Index of Mental Capacity,[28 [translated in Li et al. 2008b]] and other tests using a doctor-prepared questionnaire.[56, 57] There were 12 low risk-of-bias studies and 47 high risk-of-bias studies (**eFigure 2a**).

eFigure 1. Information on Study Location by Year and Reference Flow Diagram

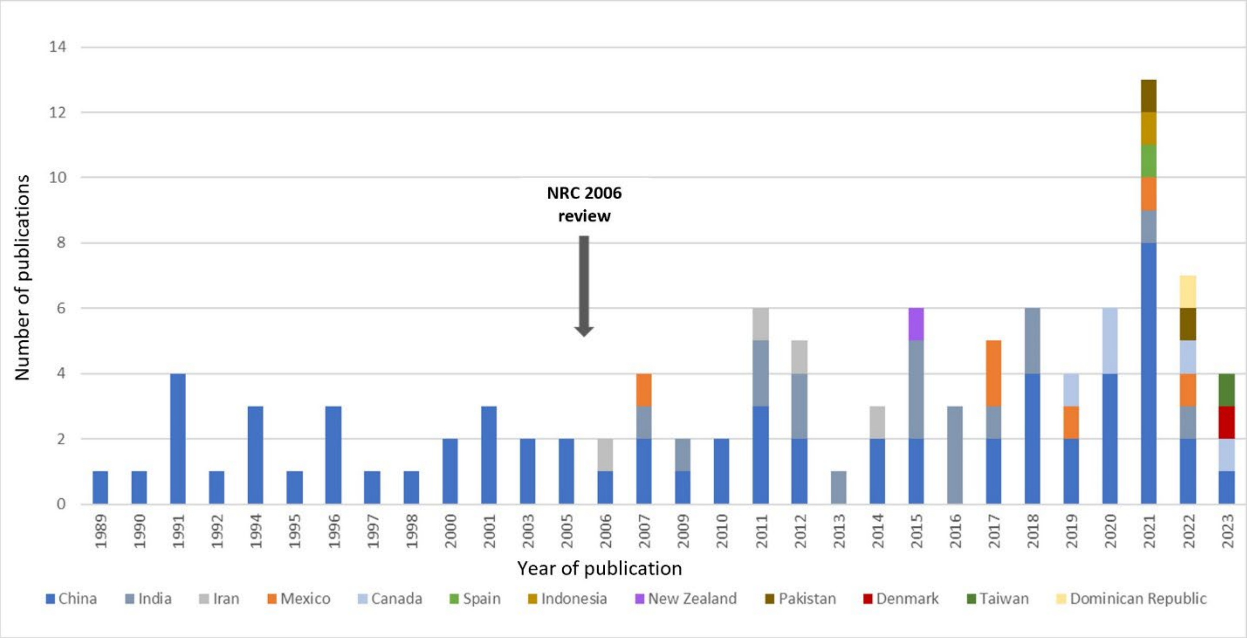

eFigure 1a. Number of Published Studies Evaluating the Association Between Estimates of Fluoride Exposure and IQ in Children, Identified by Country and Year of Publication

Note: Figure includes 104 epidemiological studies that were identified during the larger systematic review and the literature searches through October 2023 that evaluated the effects of fluoride exposure on children's IQ.

NRC: National Research Council

© 2025 Taylor KW et al. *JAMA Pediatrics*.

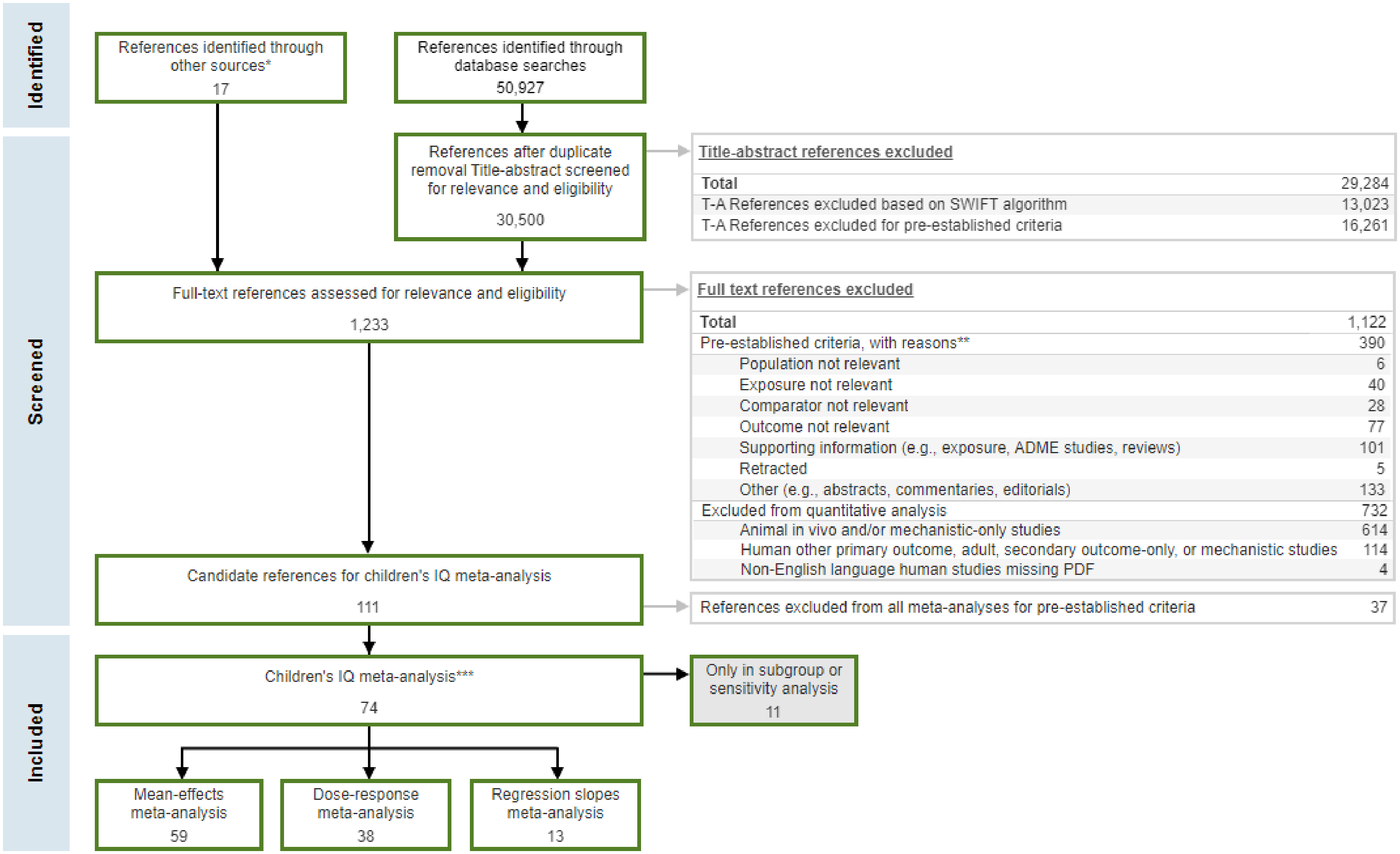

eFigure 1b. Reference Flow Diagram[a]

[a]An interactive version of **eFigure 1b** is available here: https://hawcproject.org/summary/visual/assessment/405/eFigure-1-Reference-Flow-Diagram/.

*This information was part of a larger systematic review effort resulting in many studies in the search strategy and reference flow diagram that were not considered for meta-analysis.

**Studies may have been excluded for more than one reason. The first one identified by the screener was recorded.

***Two studies that were included in sensitivity analyses evaluated cognitive function other than IQ. Multiple studies were included in more than one meta-analysis.

© 2025 Taylor KW et al. *JAMA Pediatrics*.

eTable 1. Characteristics of Studies Included in the Primary and Subgroup Analyses for the Mean-Effects, Dose-Response, and Regression-Slopes Meta-Analyses

Reference[a]<br>Study Design<br>Location<br>*Risk-of-bias Determination*	Fluoride Exposure		Mean-effects Meta-analysis<br><br>N, Mean (SD)<br>[Reference]<br>[Exposed]	Dose-response Mean-effects Meta-analysis<br><br>N, Mean (SD)<br>[Reference]<br>[Exposed]	Regression Slopes Meta-analysis<br><br>Slope (SE) or 95% CI per Unit Change Fluoride	Intelligence Assessment Method<br>Age Range (Years)<br>Confounders Considered	Source in Study
	Assessment (Metric, Exposure Groups)	Levels					
Ren et al. (1989)[38] [translated in Ren et al. 2008][me, o]<br>Cross-sectional<br>China<br>*High*	No fluoride measurement<br>Low iodine village/high fluoride and low iodine village	Not specified	169, 85.00 (22.30)<br>160, 64.80 (20.40)			Wechsler Intelligence Scale for Children<br>Ages: 8–14<br>Sex; iodine	Subjects, Methods, Results section
Chen et al. (1991)[59] [translated in Chen et al. 2008][me, w]<br>Cross-sectional<br>China<br>*High*	Drinking water<br>Nonendemic/endemic fluorosis village	0.89 mg/L (nonendemic)<br>4.55 mg/L (endemic)	320, 104.03 (14.96)<br>320, 100.24 (14.52)	320, 104.03 (14.96)<br>320, 100.24 (14.52)		Chinese Standardized Raven Test<br>Ages: 7–14<br>Age; sex	Results section, Table 1
Guo et al. (1991)[26] [translated in Guo et al. 2008a][me, o]<br>Cross-sectional<br>China<br>*High*	Serum<br>Reference area using wood/coal burning-related fluoride endemic area	0.1044 ± 0.0652 mg/L (reference)<br>0.1483 ± 0.0473 mg/L (endemic)	61, 81.39 (10.26)<br>60, 76.71 (10.85)			Chinese Binet Intelligence Test<br>Ages: 7–13<br>Age; sex; SES	Calculated by ICF

© 2025 Taylor KW et al. *JAMA Pediatrics*.

Reference[a]<br>Study Design<br>Location<br>*Risk-of-bias Determination*	Fluoride Exposure		Mean-effects Meta-analysis<br><br>N, Mean (SD)<br>[Reference]<br>[Exposed]	Dose-response Mean-effects Meta-analysis<br><br>N, Mean (SD)<br>[Reference]<br>[Exposed]	Regression Slopes Meta-analysis<br><br>Slope (SE) or 95% CI per Unit Change Fluoride	Intelligence Assessment Method<br>Age Range (Years)<br>Confounders Considered	Source in Study
	Assessment (Metric, Exposure Groups)	Levels					
Lin et al. (1991)[61, me, o]<br>Cross-sectional<br>China<br>*High*	Urine, drinking water<br>Reference area with iodine supplementation/high fluoride and low iodine village	Urine: 1.6 mg/L (reference area with iodine supplementation)<br>2.56 mg/L (high fluoride, low iodine village)<br>Water: 0.34 mg/L (low iodine village)<br>0.88 mg/L (high fluoride, low iodine village)	256, 78.00 (40.07)<br>250, 71.00 (40.07)			Combined Raven's Test for Rural China<br>Ages: 7–14<br>SES	Calculated by ICF
Sun et al. (1991)[54, me, o]<br>Cross-sectional<br>China<br>*High*	No fluoride measurement<br>Nonendemic area/endemic (aluminum-fluoride endemic toxicosis)	Fluorosis: 98.36% (endemic)	224, 82.68 (10.91)<br>196, 72.35 (11.36)			Japan's Shigeo Kobayashi's 50-point scoring method<br>Ages: 6.5–12<br>Age	Calculated by ICF
An et al. (1992)[40, me, w]<br>Cross-sectional<br>China<br>*High*	Drinking water<br>Nonhigh/high fluoride area	0.6−1.0 mg/L (nonhigh)<br>2.1−3.2 mg/L (secondary high)<br>5.2−7.6 mg/L (high)<br>2.1−7.6 mg/L (combined high)	121, 84.00 (12.10)<br>121, 75.90 (13.60)	121, 84.00 (12.10)<br>56, 76.10 (13.90)<br>65, 75.60 (13.30)		Wechsler Intelligence Scale for Children-Revised<br>Ages: 7–16<br>Age; race; SES	Table 1, Table 2
Li et al. (1994)[28] [translated in Li et al. 2008b][me, o]<br>Cross-sectional<br>China<br>*High*	Grain (cooked by burning high-fluoride coal)<br>Reference group (no dental fluorosis)/high fluoride group I (no dental fluorosis)/high fluoride group II (dental fluorosis present)/high fluoride group III (dental fluorosis present)	0.5 mg/kg (reference group)<br>4.7 mg/kg (group I)<br>5.2 mg/kg (group II)<br>31.6 mg/kg (group III)	49, 267.20 (39.50)<br>36, 240.00 (30.80)			Proofing test<br>Ages: 12–13<br>Age; sex; SES	Table 1

© 2025 Taylor KW et al. *JAMA Pediatrics*.

Reference[a] Study Design Location *Risk-of-bias Determination*	Fluoride Exposure		Mean-effects Meta-analysis N, Mean (SD) [Reference] [Exposed]	Dose-response Mean-effects Meta-analysis N, Mean (SD) [Reference] [Exposed]	Regression Slopes Meta-analysis Slope (SE) or 95% CI per Unit Change Fluoride	Intelligence Assessment Method Age Range (Years) Confounders Considered	Source in Study
	Assessment (Metric, Exposure Groups)	Levels					
Xu et al. (1994)[42, me, w*]<br>Cross-sectional<br>China<br>*High*	Drinking water<br>Reference region/low- and high-fluoride regions[b]	0.8 mg/L (reference region)<br>0.38 mg/L (low fluoride)<br>1.8 mg/L (high fluoride)	32, 83.83 (9.10)<br>97, 79.25 (2.25)	32, 83.83 (9.10)<br>21, 80.21 (8.27)<br>97, 79.25 (2.25)		Binet-Simon Scale<br>Ages: 8–14<br>–	Chart 1
Li et al. (1995)[30, me, o, u]<br>Cross-sectional<br>China<br>*High*	Urine, dental fluorosis index (DFI)<br>Nonfluorosis/fluorosis area due to soot from coal burning	1.02 mg/L; DFI: <0.4 (nonfluorosis)<br>1.81 mg/L; DFI: 0.8 (slight fluorosis)<br>2.01 mg/L; DFI: 2.5 (medium fluorosis)<br>2.69 mg/L; DFI: 3.2 (severe fluorosis)	226, 89.90 (10.40)<br>230, 80.30 (12.90)	226, 89.90 (10.40)<br>227, 89.70 (12.70)<br>224, 79.70 (12.70)<br>230, 80.30 (12.90)		China Rui Wen Scaler for Rural Areas<br>Ages: 8–13<br>Sex	Table 2
Wang et al. (1996)[36] [translated in Wang et al. 2008b][me, o, w]<br>Cross-sectional<br>China<br>*High*	Drinking water (well)<br>Low/high fluoride regions<br>Fluoride exposure from drinking water, contaminated food, and coal burning	0.58−1.0 mg/L (low)<br>>1.0−8.6 mg/L (high)	83, 101.23 (15.84)<br>147, 95.64 (14.34)	83, 101.23 (15.84)<br>147, 95.64 (14.34)		Wechsler Preschool and Primary Scale of Intelligence<br>Ages: 4–7<br>Age; sex	Table 1
Yao et al. (1996)[44, me, w]<br>Cross-sectional<br>China<br>*High*	Drinking water<br>Nonendemic/endemic fluorosis areas	1 mg/L (nonendemic)<br>2 mg/L (slightly endemic)<br>11 mg/L (severely endemic)	270, 98.46 (13.21)<br>78, 92.53 (12.34)	270, 98.46 (13.21)<br>188, 94.89 (11.15)<br>78, 92.53 (12.34)		Raven Test – Associative Atlas<br>Ages: 8–12<br>Iodine; SES	Table 2

© 2025 Taylor KW et al. *JAMA Pediatrics*.

Reference[a] Study Design Location *Risk-of-bias Determination*	Fluoride Exposure		Mean-effects Meta-analysis N, Mean (SD) [Reference] [Exposed]	Dose-response Mean-effects Meta-analysis N, Mean (SD) [Reference] [Exposed]	Regression Slopes Meta-analysis Slope (SE) or 95% CI per Unit Change Fluoride	Intelligence Assessment Method Age Range (Years) Confounders Considered	Source in Study
	Assessment (Metric, Exposure Groups)	Levels					
Zhao et al. (1996)[62, me, w]<br>Cross-sectional<br>China<br>*High*	Drinking water<br>Low fluoride village (Xinghua)/high fluoride village (Sima)	0.91 mg/L (low)<br>4.12 mg/L (high)	160, 105.21 (14.99)<br>160, 97.69 (13.00)	160, 105.21 (14.99)<br>160, 97.69 (13.00)		China Rui Wen Scaler for Rural Areas<br>Ages: 7–14<br>Age; SES	Table 1
Yao (1997)[43, me, w*]<br>Cross-sectional<br>China<br>*High*	Drinking water<br>Nonfluorosis/fluorosis area with water improvements/fluorosis area without water improvements	0.4 mg/L (nonfluorosis area)<br>0.33 mg/L (fluorosis area with water improvement)<br>2 mg/L (fluorosis area without water improvement)	314, 99.98 (12.21)<br>183, 94.89 (11.15)	314, 99.98 (12.21)<br>326, 97.83 (11.27)<br>183, 94.89 (11.15)		Raven's Standard Progressive Matrices (China's Rural Version)<br>Ages: 7–12<br>Iodine; SES	Section 2.1 Intelligence Tests, page 2
Zhang et al. (1998)[55, me, o]<br>Cross-sectional<br>China<br>*High*	Drinking water<br>Reference/high fluoride group<br>(all observation groups included arsenic exposure)	0.58 mg/L (reference)<br>0.8 mg/L (high fluoride)	52, 87.69 (11.04)<br>51, 85.62 (13.23)			Shigeo Kobayashi 50-pt. test<br>Ages: 4–10<br>Age; arsenic	Table 1
Lu et al. (2000)[63, me, w, u]<br>Cross-sectional<br>China<br>*High*	Urine, drinking water<br>Low/high fluoride area	Urine: 1.43 ± 0.64 mg/L (low)<br>4.99 ± 2.57 mg/L (high)<br>Water: 0.37 ± 0.04 mg/L (low)<br>3.15 ± 0.61 mg/L (high)	58, 103.05 (13.86)<br>60, 92.27 (20.45)	58, 103.05 (13.86)<br>60, 92.27 (20.45)		Chinese Combined Raven Test-C2<br>Ages: 10–12<br>SES	Table 1

© 2025 Taylor KW et al. *JAMA Pediatrics*.

Reference[a]<br>Study Design<br>Location<br>*Risk-of-bias Determination*	Fluoride Exposure		Mean-effects Meta-analysis<br><br>N, Mean (SD)<br>[Reference]<br>[Exposed]	Dose-response Mean-effects Meta-analysis<br><br>N, Mean (SD)<br>[Reference]<br>[Exposed]	Regression Slopes Meta-analysis<br><br>Slope (SE) or 95% CI per Unit Change Fluoride	Intelligence Assessment Method<br>Age Range (Years)<br>Confounders Considered	Source in Study
	Assessment (Metric, Exposure Groups)	Levels					
Hong et al. (2001)[64] [translated in Hong et al. 2008][me, w]<br>Cross-sectional<br>China<br>*High*	Drinking water<br>Reference/high fluoride[b]	0.75 mg/L (reference)<br>2.90 mg/L (high fluoride)	32, 82.79 (8.98)<br>85, 80.58 (2.28)	32, 82.79 (8.98)<br>85, 80.58 (2.28)		Chinese Standardized Raven Test<br>Ages: 8–14<br>Iodine; SES; demographics	Table 2
Hong et al. (2001b)[66, me, o]<br>Cross-sectional<br>China<br>*High*	Urine, drinking water<br>Nonendemic/endemic fluorosis areas (high fluoride, high iodine)	Urine: 0.796 ± 0.53 mg/L (nonendemic)<br>2.09 ± 1.03 mg/L (endemic)<br>Water: 0.48 mg/L (nonendemic)<br>2.81 mg/L (endemic)	30, 80.66 (11.93)<br>31, 75.89 (7.74)			Combined Raven's Test for Rural China<br>Ages: 8–14<br>–	Table 3, Table 4
Wang et al. (2001)[67, me, o]<br>Cross-sectional<br>China<br>*High*	Urine, drinking water<br>Reference point (low fluoride, low iodine)/investigative point (high fluoride, high iodine)	Urine: 0.82 mg/L (low fluoride, low iodine)<br>3.08 mg/L (high fluoride, high iodine)<br>Water: 0.5 mg/L (low fluoride, low iodine)<br>2.97 mg/L (high fluoride, high iodine)	30, 81.67 (11.97)<br>30, 76.67 (7.75)			Combined Raven's Test for Rural China<br>Ages: 8–12<br>–	Table 2
Li et al. (2003)[68] [translated in Li et al. 2008c][me]<br>Cross-sectional<br>China<br>*High*	No fluoride measurement<br>Reference/endemic fluorosis areas	Not specified	236, 93.78 (14.30)<br>720, 92.07 (17.12)			Chinese Standardized Raven Test<br>Ages: 6–13<br>–	Table 1

© 2025 Taylor KW et al. *JAMA Pediatrics*.

Reference[a] Study Design Location *Risk-of-bias Determination*	Fluoride Exposure: Assessment (Metric, Exposure Groups)	Fluoride Exposure: Levels	Mean-effects Meta-analysis N, Mean (SD) [Reference] [Exposed]	Dose-response Mean-effects Meta-analysis N, Mean (SD) [Reference] [Exposed]	Regression Slopes Meta-analysis Slope (SE) or 95% CI per Unit Change Fluoride	Intelligence Assessment Method Age Range (Years) Confounders Considered	Source in Study
Xiang et al. (2003a)[70, me, w*, u]<br>Cross-sectional<br>China<br>*Low*	Urine, drinking water<br>Nonendemic/endemic fluorosis areas	Urine: 1.11 ± 0.39 mg/L (reference)<br>3.47 ± 1.95 mg/L (high fluoride)<br>Water: 0.36 ± 0.15 mg/L (nonendemic)<br>0.75 ± 0.14 mg/L (endemic fluorosis area group A)<br>1.53 ± 0.27 mg/L (endemic fluorosis area group B)<br>2.46 ± 0.3 mg/L (endemic fluorosis area group C)<br>3.28 ± 0.25 mg/L (endemic fluorosis area group D)<br>4.16 ± 0.22 mg/L (endemic fluorosis area group E)<br>2.47 ± 0.79 mg/L (high fluoride)	290, 100.41 (13.21)<br>222, 92.02 (13.00)	290, 100.41 (13.21)<br>9, 99.56 (14.13)<br>42, 95.21 (12.22)<br>111, 92.19 (12.98)<br>52, 89.88 (11.98)<br>8, 78.38 (12.68)		Combined Raven's Test for Rural China<br>Ages: 8–13<br>Age; sex; iodine; lead; SES	Table 6, Table 8
Wang et al. (2005)[71, me, w, u]<br>Cross-sectional<br>China<br>*High*	Urine, drinking water<br>Reference/high fluoride group[c]	Urine: 1.51 mg/L(reference)<br>5.09 mg/L (high fluoride group)<br>Water: 0.48 mg/L (reference)<br>8.31 mg/L (high fluoride group)	196, 112.36 (14.87)<br>253, 107.83 (15.45)	196, 112.36 (14.87)<br>253, 107.83 (15.45)		Chinese Combined Raven Test-C2<br>Ages: 8–12<br>SES	Table 1

© 2025 Taylor KW et al. *JAMA Pediatrics*.

Reference[a] Study Design Location *Risk-of-bias Determination*	Fluoride Exposure		Mean-effects Meta-analysis N, Mean (SD) [Reference] [Exposed]	Dose-response Mean-effects Meta-analysis N, Mean (SD) [Reference] [Exposed]	Regression Slopes Meta-analysis Slope (SE) or 95% CI per Unit Change Fluoride	Intelligence Assessment Method Age Range (Years) Confounders Considered	Source in Study
	Assessment (Metric, Exposure Groups)	Levels					
Seraj et al. (2006)[45, me, w]<br>Cross-sectional<br>Iran<br>*High*	Drinking water<br>Low/high fluoride area	0.4 ppm (low)<br>2.5 ppm (high)	85, 98.90 (12.90)<br>41, 87.90 (11.00)	85, 98.90 (12.90)<br>41, 87.90 (11.00)		Raven Test<br>Ages: 7–11<br>Sex	Methodology, Findings section (Text under Table 2)
Wang et al. (2006)[72, me, w, u]<br>Cross-sectional<br>China<br>*High*	Urine, drinking water<br>Reference/high (area severely affected by fluorosis)	Urine: 1.51 ± 1.66 mg/L (reference)<br>5.50 ± 2.40 mg/L (high)<br>Water: 0.73 ± 0.28 mg/L (reference)<br>5.54 ± 3.88 mg/L (high)	166, 111.55 (15.19)<br>202, 107.46 (15.38)	166, 111.55 (15.19)<br>202, 107.46 (15.38)		Combined Raven's Test for Rural China<br>Ages: 8–12<br>–	Table 2
Fan et al. (2007)[73, me, w, u]<br>Cross-sectional<br>China<br>*High*	Urine, drinking water<br>Low/high fluoride area	Urine: 1.78 ± 0.46 mg/L (low)<br>2.89 ± 1.97 mg/L (high)<br>Water: 1.03 mg/L (low)<br>3.15 mg/L (high)	37, 98.41 (14.75)<br>42, 96.11 (12.00)	37, 98.41 (14.75)<br>42, 96.11 (12.00)		Chinese Combined Raven Test-C2<br>Ages: 7–14<br>–	Table 1
Trivedi et al. (2007)[57, me, w, u]<br>Cross-sectional<br>India<br>*High*	Urine, drinking water<br>Low/high fluoride area	Urine: 2.30 ± 0.28 mg/L (low)<br>6.13 ± 0.67 mg/L (high)<br>Water: 2.01 ± 0.009 mg/L (low)<br>5.55 ± 0.41 mg/L (high)	101, 104.44 (12.36)<br>89, 91.72 (10.66)	101, 104.44 (12.36)<br>89, 91.72 (10.66)		Questionnaire prepared by Professor JH Shah<br>Ages: 12–13<br>Age; sex	Table 2

© 2025 Taylor KW et al. *JAMA Pediatrics*.

Reference[a]<br>Study Design<br>Location<br>*Risk-of-bias Determination*	Fluoride Exposure		Mean-effects Meta-analysis<br><br>N, Mean (SD)<br>[Reference]<br>[Exposed]	Dose-response Mean-effects Meta-analysis<br><br>N, Mean (SD)<br>[Reference]<br>[Exposed]	Regression Slopes Meta-analysis<br><br>Slope (SE) or 95% CI per Unit Change Fluoride	Intelligence Assessment Method<br>Age Range (Years)<br>Confounders Considered	Source in Study
	Assessment (Metric, Exposure Groups)	Levels					
Wang et al. (2007)[74, me, o, u, w]<br><br>Cross-sectional<br>China<br>*High*	Urine, drinking water<br>Low fluoride, low arsenic/high fluoride, low arsenic area	Urine: 1.5 ± 1.6 mg/L (low fluoride, low arsenic)<br>5.1 ± 2.0 mg/L (high fluoride, low arsenic)<br><br>Water: 0.5 ± 0.2 mg/L (low fluoride, low arsenic)<br>8.3 ± 1.9 mg/L (high fluoride, low arsenic)	196, 104.80 (14.70)<br>253, 100.50 (15.80)	196, 104.80 (14.70)<br>253, 100.50 (15.80)		Combined Raven's Test for Rural China<br>Ages: 8–12<br>Age; sex; arsenic; SES	Table 2, Table 3
Li et al. (2009)[31, me, o, u*]<br><br>Cross-sectional<br>China<br>*High*	Urine<br><br>Endemic fluorosis region caused by coal burning (reference/mild/medium /severe)<br>Degree of dental fluorosis (normal/suspected/very mild/mild/medium/ severe)	0.962 ± 0.517 mg/L (reference)<br>1.235 ± 0.426 mg/L (mild)<br>1.670 ± 0.663 mg/L (medium)<br>2.336 ± 1.128 mg/L (severe)<br><br>0.867 ± 0.233 mg/L (normal)<br>1.094 ± 0.355 mg/L (suspected)<br>1.173 ± 0.480 mg/L (very mild)<br>1.637 ± 0.682 mg/L (mild)<br>2.005 ± 0.796 mg/L (medium)<br>2.662 ± 1.093 mg/L (severe)	20, 102.70 (17.61)<br>20, 93.85 (18.11)	20, 102.70 (17.61)<br>20, 97.30 (18.56)<br>20, 93.90 (17.60)<br>20, 93.85 (18.11)		Combined Raven's Test for Rural China<br>Ages: 8–12<br>Age; sex	Table 1

© 2025 Taylor KW et al. *JAMA Pediatrics*.

Reference[a]<br>Study Design<br>Location<br>*Risk-of-bias Determination*	Fluoride Exposure<br>Assessment (Metric, Exposure Groups)	 Levels	Mean-effects Meta-analysis<br>N, Mean (SD)<br>[Reference]<br>[Exposed]	Dose-response Mean-effects Meta-analysis<br>N, Mean (SD)<br>[Reference]<br>[Exposed]	Regression Slopes Meta-analysis<br>Slope (SE) or 95% CI per Unit Change Fluoride	Intelligence Assessment Method<br>Age Range (Years)<br>Confounders Considered	Source in Study
Li et al. (2010)[75, me]<br>Cross-sectional<br>China<br>*High*	No fluoride measurement<br>Nondental fluorosis children/dental fluorosis children	Not specified	329, 97.36 (18.24)<br>347, 98.73 (21.07)			Combined Raven's Test for Rural China<br>Ages: 7–10<br>Sex	Table 3
Ding et al. (2011)[76, me, u*, rs]<br>Cross-sectional<br>China<br>*Low*	Dental fluorosis (normal/questionable/very mild/mild/moderate)<br>Urine<br>Mean urinary fluoride levels (10 groups)	0.80 ± 0.55 mg/L (normal)<br>1.13 ± 0.73 mg/L (questionable)<br>1.11 ± 0.74 mg/L (very mild)<br>1.31 ± 0.78 mg/L (mild)<br>1.46 ± 0.79 mg/L (moderate)<br>0.26 mg/L (group 1)<br>0.45 mg/L (group 2)<br>0.56 mg/L (group 3)<br>0.66 mg/L (group 4)<br>0.75 mg/L (group 5)<br>0.89 mg/L (group 6)<br>1.08 mg/L (group 7)<br>1.33 mg/L (group 8)<br>1.74 mg/L (group 9)<br>2.96 mg/L (group 10)<br>Range: 0.10−3.55 mg/L	136, 104.07 (12.30)<br>28, 103.54 (13.59)	136, 104.07 (12.30)<br>54, 103.00 (16.10)<br>74, 102.11 (15.05)<br>39, 106.03 (12.33)<br>28, 103.54 (13.59)	−0.59 (−1.09, −0.08) per 1 mg/L urinary F	Combined Raven's Test for Rural China<br>Ages: 7–14<br>Age; arsenic; iodine; lead; SES; demographics	Table 2, Section 3 Results and Discussion (under Fig. 2)
Eswar et al. (2011)[47, me, w]<br>Cross-sectional<br>India<br>*High*	Drinking water<br>Low/high fluoride villages	0.29 mg/L (low)<br>2.45 mg/L (high)	65, 88.80 (15.30)<br>68, 86.30 (12.80)	65, 88.80 (15.30)<br>68, 86.30 (12.80)		Standard Progressive Matrices<br>Ages: 12–14<br>Age; sex	Table 1

© 2025 Taylor KW et al. *JAMA Pediatrics*.

Reference[a] Study Design Location *Risk-of-bias Determination*	Fluoride Exposure: Assessment (Metric, Exposure Groups)	Fluoride Exposure: Levels	Mean-effects Meta-analysis N, Mean (SD) [Reference] [Exposed]	Dose-response Mean-effects Meta-analysis N, Mean (SD) [Reference] [Exposed]	Regression Slopes Meta-analysis Slope (SE) or 95% CI per Unit Change Fluoride	Intelligence Assessment Method Age Range (Years) Confounders Considered	Source in Study
Kang et al. (2011)[77, me, o]<br>Cross-sectional<br>China<br>*High*	Drinking water<br>Reference/high fluoride areas<br>(both areas high arsenic exposure)	1.24 ± 0.74 mg/L (all children)<br><1.2 mg/L (reference)<br>≥1.2 mg/L (high fluoride)	90, 96.8 (12.7)<br>178, 96.8 (16.3)			Chinese Combined Raven Test-C2<br>Ages: 6–12<br>Age; sex	Table 1. Section 2.1
Poureslami et al. (2011)[48, me, w]<br>Cross-sectional<br>Iran<br>*High*	Drinking water<br>Reference/endemic dental fluorosis city	0.41 mg/L (reference)<br>2.38 mg/L (endemic)	60, 97.80 (15.95)<br>59, 91.37 (16.63)	60, 97.80 (15.95)<br>59, 91.37 (16.63)		Persian version of Raven's Matrices Test<br>Ages: 7–9<br>Sex	Table 3, Results section (under Table 3)
Shivaprakash et al. (2011)[49, me, w]<br>Cross-sectional<br>India<br>*High*	Drinking water<br>No fluorosis/fluorosis severity groups (mild/moderate/severe)/all fluorosis	<0.5 ppm (no fluorosis)<br>2.5–3.5 ppm (mild)<br>2.5–3.5 ppm (moderate)<br>2.5–3.5 ppm (severe)<br>2.5–3.5 ppm (all)	80, 76.36 (20.84)<br>80, 66.63 (18.09)	80, 76.36 (20.84)<br>80, 66.63 (18.09)		Raven's Colored Progressive Matrices<br>Ages: 7–11<br>Health factors; SES	Table 1
Seraj et al. (2012)[46, me, w]<br>Cross-sectional<br>Iran<br>*Low*	Drinking water<br>Normal/medium/high fluoride levels	0.8 ± 0.3 mg/L (normal)<br>3.1 ± 0.9 mg/L (medium)<br>5.2 ± 1.1 mg/L (high)	91, 97.77 (18.91)<br>96, 88.58 (16.01)	91, 97.77 (18.91)<br>106, 89.03 (12.99)<br>96, 88.58 (16.01)		Raven's Colored Progressive Matrices<br>Ages: 6–11<br>Age; sex; SES	Table 2
Trivedi et al. (2012)[56, me, w, u]<br>Cross-sectional<br>India<br>*Low*	Urine, ground water<br>Low/high fluoride area	Urine: 0.42 ± 0.23 mg/L (low)<br>2.69 ± 0.92 mg/L (high)<br>Water: 0.84 ± 0.38 mg/L (low)<br>2.3 ± 0.87 mg/L (high)	50, 97.17 (17.96)<br>34, 92.58 (18.25)	50, 97.17 (17.96)<br>34, 92.58 (18.25)		Questionnaire prepared by Professor JH Shah<br>Ages: 12–13<br>Sex; SES	Table 3, Results section (above Table 3)

© 2025 Taylor KW et al. *JAMA Pediatrics*.

Reference[a] Study Design Location *Risk-of-bias Determination*	Fluoride Exposure: Assessment (Metric, Exposure Groups)	Fluoride Exposure: Levels	Mean-effects Meta-analysis N, Mean (SD) [Reference] [Exposed]	Dose-response Mean-effects Meta-analysis N, Mean (SD) [Reference] [Exposed]	Regression Slopes Meta-analysis Slope (SE) or 95% CI per Unit Change Fluoride	Intelligence Assessment Method Age Range (Years) Confounders Considered	Source in Study
Wang et al. (2012b)[78, me]<br>Cross-sectional<br>China<br>*High*	No fluoride measurement<br>Reference/high fluoride areas	Not specified	455, 98.36 (14.56)<br>800, 92.21 (18.45)			Combined Raven's Test for Rural China<br>Ages: Primary school age<br>–	Table 1
Bai et al. (2014)[32, me, o]<br>Cross-sectional<br>China<br>*High*	Urine<br>Coal-burning-borne fluorosis areas (reference/lightly affected/seriously affected)	0.54 mg/L (reference)<br>0.81 mg/L (lightly affected area)<br>1.96 mg/L (seriously affected area)	164, 107.92 (13.62)<br>162, 101.22 (15.97)			Chinese Combined Raven Test-C2<br>Ages: 8–12<br>SES	Table 2
Karimzade et al. (2014)[53, me, w]<br>Cross-sectional<br>Iran<br>*High*	Drinking water<br>Low/high fluoride area	0.25 mg/L (low)<br>3.94 mg/L (high)	20, 104.25 (20.75)<br>19, 81.21 (16.17)	20, 104.25 (20.75)<br>19, 81.21 (16.17)		Iranian version of the Raymond B Cattell test<br>Ages: 9–12<br>Sex	Table 1

© 2025 Taylor KW et al. *JAMA Pediatrics*.

Reference[a] Study Design Location *Risk-of-bias Determination*	Fluoride Exposure		Mean-effects Meta-analysis N, Mean (SD) [Reference] [Exposed]	Dose-response Mean-effects Meta-analysis N, Mean (SD) [Reference] [Exposed]	Regression Slopes Meta-analysis Slope (SE) or 95% CI per Unit Change Fluoride	Intelligence Assessment Method Age Range (Years) Confounders Considered	Source in Study
	Assessment (Metric, Exposure Groups)	Levels					
Broadbent et al. (2015)[41, me, w*]<br>Prospective Cohort<br>New Zealand<br>*High*	Drinking water<br>Area without community water fluoridation (low)/area with community water fluoridation (high)<br>Fluoride tablet use (never/ever)<br>Fluoride toothpaste use (never/sometimes/always)	Water: 0.0–0.3 mg/L (low)<br>0.7–1.0 mg/L (high)<br>Tablet use: 0 mg (never used)<br>0.5 mg (ever used)<br>Range not specified for fluoride toothpaste use (always/sometimes/never)	99, 99.80 (14.50)<br>891, 100.00 (15.10)	99, 99.80 (14.50)<br>891, 100.00 (15.10)		Wechsler Intelligence Scale for Children-Revised<br>Ages: 7–13<br>Sex; SES; low birth weight; breastfeeding	Table 1
Khan et al. (2015)[50, me]<br>Cross-sectional<br>India<br>*High*	Drinking water<br>Low fluoride areas (Tiwariganj)/high fluoride areas (Unnao)<br>Fluorosis grades (normal/very mild/mild/moderate/severe)	0.19 mg/L (Tiwariganj)<br>2.41 mg/L (Unnao)<br>Ranges not specified by fluorosis grades	241, 110.10 (9.00)<br>5, 62.40 (2.40)			Raven's Colored Progressive Matrices<br>Ages: 6–11<br>Health factors; SES	Table/Fig-5
Kundu et al. (2015)[79, sa]<br>Cross-sectional<br>India<br>*High*	Drinking water<br>Low fluoride areas/high fluoride areas	Not specified	100, 85.80 (18.85)<br>100, 76.20 (19.10)			Raven's Standardized Progressive Matrices<br>Ages: 8−12<br>–	Table 2
Sebastian and Sunitha (2015)[51, me, w*]<br>Cross-sectional<br>India<br>*High*	Drinking water<br>Low/normal/high fluoride villages	0.40 mg/L (low)<br>1.2 mg/L (normal)<br>2.0 mg/L (high)	135, 86.37 (13.58)<br>135, 80.49 (12.67)	135, 86.37 (13.58)<br>135, 88.60 (14.01)<br>135, 80.49 (12.67)		Raven's Colored Progressive Matrices<br>Ages: 10–12<br>Age; sex; SES	Table 1, Table 2

© 2025 Taylor KW et al. *JAMA Pediatrics*.

Reference[a] Study Design Location *Risk-of-bias Determination*	Fluoride Exposure: Assessment (Metric, Exposure Groups)	Fluoride Exposure: Levels	Mean-effects Meta-analysis N, Mean (SD) [Reference] [Exposed]	Dose-response Mean-effects Meta-analysis N, Mean (SD) [Reference] [Exposed]	Regression Slopes Meta-analysis Slope (SE) or 95% CI per Unit Change Fluoride	Intelligence Assessment Method Age Range (Years) Confounders Considered	Source in Study
Zhang et al. (2015b)[80, me, w*, u, rs]<br>Cross-sectional<br>China<br>*Low*	Urine, drinking water, serum<br>Reference/high fluoride areas	Urine: 1.10 ± 0.67 mg/L (reference)<br>2.40 ± 1.01 mg/L (high)<br>Water: 0.63 (0.58–0.68) mg/L (reference)<br>1.40 (1.23–1.57) mg/L (high)<br>Serum: 0.06 ± 0.03 (reference)<br>0.18 ± 0.11 (high)	96, 109.42 (13.30)<br>84, 102.33 (13.46)	96, 109.42 (13.30)<br>84, 102.33 (13.46)	−2.42 (−4.59, −0.24) per 1 mg/L urinary F	Combined Raven's Test for Rural China<br>Ages: 10–12<br>Age; sex; arsenic; iodine; drinking water fluoride; SES; thyroid hormone levels; COMT genotype	Table 1, Table 3
Zhang et al. (2015c)[81, me, o]<br>Cross-sectional<br>China<br>*High*	Urine<br>Coal-burning endemic fluorosis area<br>Reference (no dental fluorosis)/mild dental fluorosis/middle dental fluorosis/critically ill dental fluorosis	0.83 ± 0.71 mg/L (reference)<br>1.54 ± 0.57 mg/L (mildly ill)<br>2.41 ± 0.76 mg/L (moderately ill)<br>3.32 ± 1.02 mg/L (critically ill)	30, 110.34 (11.52) (reference)<br>30, 90.52 (10.37) (critically ill)			Combined Raven's Test for Rural China<br>Ages: 7−13<br>–	Table 1, Table 3
Aravind et al. (2016)[82, sa]<br>Cross-sectional<br>India<br>*High*	Drinking water<br>Low/high fluoride levels	<1.2 ppm (low)<br>>2 ppm (high)	96, 41.03 (16.36)<br>96, 31.59 (16.81)			Raven's Standardized Progressive Matrices<br>Ages: 10−12<br>–	Table 1

© 2025 Taylor KW et al. *JAMA Pediatrics*.

Reference[a] Study Design Location *Risk-of-bias Determination*	Fluoride Exposure: Assessment (Metric, Exposure Groups)	Fluoride Exposure: Levels	Mean-effects Meta-analysis N, Mean (SD) [Reference] [Exposed]	Dose-response Mean-effects Meta-analysis N, Mean (SD) [Reference] [Exposed]	Regression Slopes Meta-analysis Slope (SE) or 95% CI per Unit Change Fluoride	Intelligence Assessment Method Age Range (Years) Confounders Considered	Source in Study
Das and Mondal (2016)[83, me, u]<br>Cross-sectional<br>India<br>*High*	Urine, drinking water intake<br>Dental fluorosis (normal/questionable/very mild/mild/moderate/severe)	Urine: 2.91 ± 1.76 mg/L (normal)<br>2.50 ± 2.39 mg/L (questionable)<br>2.58 ± 1.31 mg/L (very mild)<br>2.95 ± 1.44 mg/L (mild)<br>4.82 ± 3.57 mg/L (moderate)<br>3.81 ± 2.51 mg/L (severe)<br>Water: 0.069 ± 0.021 mg/kg-d (normal)<br>0.064 ± 0.004 mg/kg-d (questionable)<br>0.060 ± 0.036 mg/kg-d (very mild)<br>0.060 ± 0.030 mg/kg-d (mild)<br>0.099 ± 0.063 mg/kg-d (moderate)<br>0.093 ± 0.040 mg/kg-d (severe)	4, 108.30 (53.20)<br>23, 85.91 (37.68)	4, 108.30 (53.20)<br>17, 103.18 (33.35)<br>27, 107.70 (27.92)<br>35, 92.83 (26.90)<br>43, 84.51 (35.16)<br>23, 85.91 (37.68)		Combined Raven's Test for Rural China<br>Ages: 6–18<br>–	Table 3
Mondal et al. (2016)[52, me, w]<br>Cross-sectional<br>India<br>*High*	Drinking water<br>Low/high fluoride areas	Not reported (low)<br>0.33–18.08 mg/L (high)	22, 26.41(10.46)<br>18, 21.17 (6.77)	22, 26.41 (10.46)<br>18, 21.17 (6.77)		Raven Standard Theoretical Intelligence Test<br>Ages: 10–14<br>SES	Table 9

© 2025 Taylor KW et al. *JAMA Pediatrics*.

Reference[a]<br>Study Design<br>Location<br>*Risk-of-bias Determination*	Fluoride Exposure<br>Assessment (Metric, Exposure Groups)	Fluoride Exposure<br>Levels	Mean-effects Meta-analysis<br><br>N, Mean (SD)<br>[Reference]<br>[Exposed]	Dose-response Mean-effects Meta-analysis<br><br>N, Mean (SD)<br>[Reference]<br>[Exposed]	Regression Slopes Meta-analysis<br><br>Slope (SE) or 95% CI per Unit Change Fluoride	Intelligence Assessment Method<br>Age Range (Years)<br>Confounders Considered	Source in Study
Bashash et al. (2017)[84, sa]<br>Prospective Cohort<br>Mexico<br>*Low*	Urine<br>Reference/high fluoride levels (based on children's urinary fluoride)	Urine:<br><0.80 mg/L (reference)<br>≥0.80 mg/L (high)	77, 95.37 (10.31)<br>112, 96.80 (11.16)	77, 95.37 (10.31)<br>112, 96.80 (11.16)	−5.16 (−9.12, −1.19) per 1 mg/L maternal urinary F	Wechsler Abbreviated Scale of Intelligence<br>Ages: 6−12<br>Age; sex; weight at birth; parity; gestational age; maternal characteristics (smoking history, marital status, age at delivery, IQ, education, cohort)	Abstract, Table 3, author correspondence
Razdan et al. (2017)[85, sa]<br>Cross-sectional<br>India<br>*High*	Drinking water<br>Low/high fluoride levels	0.6 ppm (low)<br>4.99 ppm (high)	69, 38.61 (6.34)<br>75, 13.95 (5.14)			Raven's Standardized Progressive Matrices<br>Ages: 12–14<br>–	Table 2
Valdez Jiménez et al. (2017)[86, sa]<br>Prospective Cohort<br>Mexico<br>*Low*	Maternal urine, drinking water	Urine:<br>1.9 ± 1.0 mg/L (1st trimester)<br>2.0 ± 1.1 mg/L (2nd trimester)<br>2.7 ± 1.1 mg/L (3rd trimester)<br>Water:<br>2.6 ± 1.1 mg/L (1st trimester)<br>3.1 ± 1.1 mg/L (2nd trimester)<br>3.7 ± 1.0 mg/L (3rd trimester)			Bayley MDI:<br>−19.05 (8.9) per 1 log10 mg/L maternal urinary F (1st trimester)<br>−19.34 (7.46) per 1 log10 mg/L maternal urinary F (2nd trimester)	Bayley Scales of Infant Development II<br>Ages: Infancy<br>Age; gestational age; marginality index; type of drinking water	Table 2, Table 4

© 2025 Taylor KW et al. *JAMA Pediatrics*.

Reference[a] Study Design Location *Risk-of-bias Determination*	Fluoride Exposure: Assessment (Metric, Exposure Groups)	Fluoride Exposure: Levels	Mean-effects Meta-analysis N, Mean (SD) [Reference] [Exposed]	Dose-response Mean-effects Meta-analysis N, Mean (SD) [Reference] [Exposed]	Regression Slopes Meta-analysis Slope (SE) or 95% CI per Unit Change Fluoride	Intelligence Assessment Method Age Range (Years) Confounders Considered	Source in Study
Cui et al. (2018)[87, rs]<br>Cross-sectional<br>China<br>*Low*	Urine	Boys: 1.3 (0.9−1.7)[d] mg/L<br>Girls: 1.2 (0.9−1.6)[d] mg/L			−2.47 (−4.93, −0.01) per 1 log urinary F	Combined Raven's Test for Rural China<br>Ages: 7–12<br>Age; maternal education; smoking in family member; stress; anger; dopamine receptor-2 polymorphism	Table 2
Yu et al. (2018)[5, me, w, u*, rs]<br>Cross-sectional<br>China<br>*Low*	Maternal urine<br>Low/medium/high fluoride ranges<br>Drinking water<br>Normal/high fluoride	Urine: 0.01–1.60 mg/L (low)<br>1.60–2.50 mg/L (medium)<br>2.50–5.54 mg/L (high)<br>Water: ≤1 mg/L (normal)<br>>1 mg/L (high)<br>Overall: 0.01−5.54 mg/L (urine)<br>0.20−3.90 mg/L (water)	1636, 107.40 (13.00)<br>1250, 106.40 (12.30)	1636, 107.40 (13.00)<br>1250, 106.40 (12.30)	0.36 (−0.29, 1.01) per 0.5 mg/L maternal urinary F, 0.01–1.60 mg/L (low)<br>−2.67 (−4.67, −0.68) per 0.5 mg/L maternal urinary F, 1.60–2.50 mg/L (medium)<br>−0.84 (−2.18, 0.50) per 0.5 mg/L maternal urinary F, 2.50–5.54 mg/L (high)	Combined Raven's Test for Rural China<br>Ages: 7–13<br>Age; sex; health factors; SES	Table 1, Table 3
Zhao et al. (2018)[88, me, o]<br>Cross-sectional<br>China<br>*High*	Urine<br>Reference/exposed areas<br>All areas with iodine exposure	≤2.16 mg/L (reference)<br>>2.16 mg/L (exposed)	199, 114.52 (12.72)<br>100, 109.59 (14.24)			Combined Raven's Test for Rural China<br>Ages: 7–12<br>–	Table 4

© 2025 Taylor KW et al. *JAMA Pediatrics*.

Reference[a] Study Design Location *Risk-of-bias Determination*	Fluoride Exposure: Assessment (Metric, Exposure Groups)	Fluoride Exposure: Levels	Mean-effects Meta-analysis N, Mean (SD) [Reference] [Exposed]	Dose-response Mean-effects Meta-analysis N, Mean (SD) [Reference] [Exposed]	Regression Slopes Meta-analysis Slope (SE) or 95% CI per Unit Change Fluoride	Intelligence Assessment Method Age Range (Years) Confounders Considered	Source in Study
Green et al. (2019)[89, me, w*, u*, rs]<br>Prospective Cohort<br>Canada<br>*Low*	Maternal urine, drinking water, maternal fluoride intake<br>Nonfluoridated/fluoridated area	Urine: 0.40 ± 0.27 mg/L (nonfluoridated)<br>0.69 ± 0.42 mg/L (fluoridated)<br>Water: 0.13 ± 0.06 mg/L (nonfluoridated)<br>0.59 ± 0.08 mg/L (fluoridated)<br>Intake: 0.30 ± 0.26 mg/day (nonfluoridated)<br>0.93 ± 0.43 mg/day (fluoridated)<br>Overall: 0.51 ± 0.36 mg/L (urine)<br>0.54 ± 0.44 mg/day (intake)<br>0.31 ± 0.23 mg/L (water)	238, 108.07 (13.31)<br>162, 108.21 (13.72)	238, 108.07 (13.31)<br>162, 108.21 (13.72)	−1.95 (−5.19, 1.28) per 1 mg/L maternal urinary F<br>−5.29 (−10.39, −0.19) per 1 mg/L water F<br>−3.66 (−7.16, −0.15) per 1 mg/d maternal F intake	Wechsler Primary and Preschool Scale of Intelligence-III<br>Ages: 3−4<br>Sex; city; maternal education; race/ethnicity; HOME score; prenatal secondhand smoke exposure	Table 2, text page 945, eTable 4
Cui et al. (2020)[90, me, u]<br>Cross-sectional<br>China<br>*Low*	Urine<br>Low/medium/high fluoride levels	<1.6 mg/L (low)<br>1.6–2.5 mg/L (medium)<br>≥2.5 mg/L (high)	396, 112.16 (11.50)<br>36, 110.00 (14.92)	396, 112.16 (11.50)<br>66, 112.05 (12.01)<br>36, 110.00 (14.92)		Combined Raven's Test<br>Ages: 7–12<br>Sex; arsenic; iodine	Table 1

© 2025 Taylor KW et al. *JAMA Pediatrics*.

Reference[a] Study Design Location *Risk-of-bias Determination*	Fluoride Exposure: Assessment (Metric, Exposure Groups)	Fluoride Exposure: Levels	Mean-effects Meta-analysis N, Mean (SD) [Reference] [Exposed]	Dose-response Mean-effects Meta-analysis N, Mean (SD) [Reference] [Exposed]	Regression Slopes Meta-analysis Slope (SE) or 95% CI per Unit Change Fluoride	Intelligence Assessment Method Age Range (Years) Confounders Considered	Source in Study
Till et al. (2020)[91, rs]<br>(Subgroup analysis and sensitivity analysis)[e]<br>Prospective Cohort<br>Canada<br>*Low*	Residence, maternal urine, drinking water, infant fluoride intake from formula<br>Nonfluoridated areas/fluoridated	Urine: 0.38−0.42 mg/L (nonfluoridated) 0.64−0.70 mg/L (fluoridated)<br>Water: 0.13 mg/L (nonfluoridated) 0.58 mg/L (fluoridated)<br>Intake: 0.02−0.08 mg/day (nonfluoridated) 0.12−0.34 mg/day (fluoridated)			−2.69 (−7.38, 2.01) per 0.5 mg/day infant F intake (formula)	Wechsler Primary and Preschool Scale of Intelligence-III<br>Ages: 3−4<br>Age; sex; maternal education; maternal race; HOME total score; secondhand smoke status in the child's house	Table 2
Wang et al. (2020b)[4, sa]<br>Cross-sectional<br>China<br>*Low*	Urine, drinking water	Urine: 0.01−5.54 mg/L<br>Water: 0.20−3.90 mg/L			−1.214 (−1.987, −0.442) per 1 mg/L urinary F<br>−1.037 (−2.040, −0.035) per 1 mg/L urinary F (males)<br>−1.379 (−2.628, −0.129) per 1 mg/L urinary F (females);<br>−1.587 (−2.607, −0.568) per 1 mg/L water F<br>−1.422 (−2.792, −0.053) per 1 mg/L water F (males)<br>−1.649 (−3.201, −0.097) per 1 mg/L water F (females)	Combined Raven's Test for Rural China<br>Ages: 7−13<br>Age; sex; BMI; low birth weight; household income; parental education	Table 4

© 2025 Taylor KW et al. *JAMA Pediatrics*.

Reference[a] Study Design Location *Risk-of-bias Determination*	Fluoride Exposure		Mean-effects Meta-analysis N, Mean (SD) [Reference] [Exposed]	Dose-response Mean-effects Meta-analysis N, Mean (SD) [Reference] [Exposed]	Regression Slopes Meta-analysis Slope (SE) or 95% CI per Unit Change Fluoride	Intelligence Assessment Method Age Range (Years) Confounders Considered	Source in Study
	Assessment (Metric, Exposure Groups)	Levels					
Wang et al. (2020c)[34, me, o]<br>Cross-sectional<br>China<br>*High*	Urine<br>Coal-burning endemic fluorosis area<br>Nonendemic/endemic fluorosis regions	0.461 ± 0.210 mg/L (nonendemic)<br>0.689 ± 0.502 mg/L (endemic)	100, 97 (20.3)<br>170, 82.5 (21.7)			Combined Raven's Test for Rural China<br>Ages: 7−12<br>Age; sex	Section 2.1, Table 2
Cantoral et al. (2021)[92, sa]<br>Prospective Cohort<br>Mexico<br>*Low*	Maternal fluoride intake	1.12 ± 0.54 mg/day			Bayley III cognitive scores:<br>−1.14 (−3.26, 0.99) per 0.5 mg/L maternal F intake<br>0.07 (−2.37, 2.51) per 0.5 mg/L maternal F intake (females)<br>−3.50 (−6.58, −0.42) per 0.5 mg/L maternal F intake (males)	Bayley Scales of Infant and Toddler Development III<br>Ages: 1−2<br>Sex; gestational age; birth weight; breastfeeding; added salt; calcium intake (concurrent); maternal characteristics (age, education, SES)	Table 3, Table 4
Guo et al. (2021)[93, me]<br>(Subgroup analysis only)[f]<br>Cross-sectional<br>China<br>*High*	Urine<br>Reference/exposed areas<br>(all areas with iodine exposure)	1.16 mg/L (reference)<br>1.29 mg/L (iodine area 1)<br>2.01 mg/L (iodine area 2)	7−9 years:<br>71, 116.71 (12.16)<br>35, 118.11 (12.8)<br>22, 113.95 (12.26)<br>10−12 years:<br>79, 109.86 (12.05)<br>48, 110.83 (10.58)<br>44, 105.39 (13.6)			Combined Raven's Test for Rural China<br>Ages: 7−12<br>–	Table 2, Table 3

© 2025 Taylor KW et al. *JAMA Pediatrics*.

Reference[a] Study Design Location *Risk-of-bias Determination*	Fluoride Exposure		Mean-effects Meta-analysis<br>N, Mean (SD) [Reference] [Exposed]	Dose-response Mean-effects Meta-analysis<br>N, Mean (SD) [Reference] [Exposed]	Regression Slopes Meta-analysis<br>Slope (SE) or 95% CI per Unit Change Fluoride	Intelligence Assessment Method Age Range (Years) Confounders Considered	Source in Study
	Assessment (Metric, Exposure Groups)	Levels					
Ibarluzea et al. (2021)[94, sa]<br>Prospective Cohort<br>Spain<br>*Low*	Maternal urine<br>Nonfluorinated/fluoridated communities	Urine: 0.38 ± 0.27 mg/L (nonfluorinated)<br>0.70 ± 0.41 mg/L (fluoridated)<br>Water: <0.1 mg/L (nonfluorinated)<br>0.81 ± 0.15 mg/L (fluoridated)	Bayley MDI scores:<br>153, 97.696 (14.91)<br>160, 100.395 (15.411)<br>McCarthy GCI scores:<br>123, 98.666 (15.531)<br>124, 101.473 (15.423)	Bayley MDI scores:<br>153, 97.696 (14.91)<br>160, 100.395 (15.411)<br>McCarthy GCI scores:<br>123, 98.666 (15.531)<br>124, 101.473 (15.423)	Bayley MDI scores:<br>4.67 (−1.78, 11.13) per 1 mg/L maternal urinary F<br>7.86 (−1.68, 17.40) per 1 mg/L maternal urinary F (males)<br>1.77 (−7.32, 10.87) per 1 mg/L maternal urinary F (females)<br>McCarthy GCI scores:<br>−2.16 (−8.56, 4.23) per 1 mg/L maternal urinary F<br>−1.79 (−11.85, 8.27) per 1 mg/L maternal urinary F (males)<br>−3.60 (−12.07, 4.86) per 1 mg/L maternal urinary F (females)	Bayley Scales of Infant Development II, McCarthy Scales of Children's Abilities<br>Ages: 1, 4<br>Age at testing (McCarthy only); sex; order of child (between siblings); breastfeeding; nursery at 14 mo.; maternal characteristics (IQ, smoking, social class)	Section 2.2, author correspondence
Lou et al. (2021)[35, me, o]<br>Cross-sectional<br>China<br>*High*	Coal-burning endemic fluorosis area<br>No fluoride measurement<br>Nondental fluorosis children/dental fluorosis children	Not specified	44, 96.64 (11.70)<br>55, 88.51 (12.77)			Wechsler Intelligence Scale for Children-Revised in China (WISC-CR)<br>Ages: 8−12<br>–	Table 4
Prabhakar et al. (2021)[95, sa]<br>Cross-sectional<br>India<br>*High*	Reference (no dental fluorosis)/dental fluorosis	Urine: not specified (reference)<br>>1 mg/mL (dental fluorosis)	60, 26.15 (11.87)<br>60, 31.05 (11.06)			Raven's Standardized Progressive Matrices<br>Ages: 8−10<br>–	Table 3

© 2025 Taylor KW et al. *JAMA Pediatrics*.

Reference[a]<br>Study Design<br>Location<br>*Risk-of-bias Determination*	Fluoride Exposure		Mean-effects Meta-analysis<br><br>N, Mean (SD)<br>[Reference]<br>[Exposed]	Dose-response Mean-effects Meta-analysis<br><br>N, Mean (SD)<br>[Reference]<br>[Exposed]	Regression Slopes Meta-analysis<br><br>Slope (SE) or 95% CI per Unit Change Fluoride	Intelligence Assessment Method<br>Age Range (Years)<br>Confounders Considered	Source in Study
	Assessment (Metric, Exposure Groups)	Levels					
Saeed et al. (2021)[96, me, o, rs]<br>Cross-sectional<br>Pakistan<br>*High*	Urine, drinking water<br>Reference/high fluoride areas<br>Co-exposure with arsenic	Urine: 0.24 ± 0.15 mg/L (reference)<br>3.27 ± 2.60 mg/L (high fluoride)<br>Water: 0.15 ± 0.13 mg/L (reference)<br>5.64 ± 3.52 mg/L (high fluoride)	30, 100.93 (13.10)<br>118, 97.26 (15.39)		−3.54 (0.50) per 1 mg/L urinary F	Wechsler scale of intelligence (WISC-IV)<br>Ages: 5−16<br>Age; sex; parental education; dental fluorosis	Table 1, Table 3
Wang et al. (2021)[97, me, w]<br>Cross-sectional<br>China<br>*High*	Drinking water<br>Reference/high fluoride areas	1.0 ± 0.07 mg/L (reference)<br>2.8 ± 0.06 mg/L (high fluoride)	303, 109.0 (14.4)<br>275, 102.1 (16.3)	303, 109.0 (14.4)<br>275, 102.1 (16.3)		Combined Raven's Test<br>Ages: 9−11<br>Age; sex	Section 2.1, Table 2
Zhao et al. (2021)[98, rs]<br>Cross-sectional<br>China<br>*Low*	Urine<br>Nonendemic/endemic fluorosis areas	1.03 (0.72, 1.47) mg/L			−5.957 (−9.712, −2.202) per 1 log urinary F	Combined Raven's Test for Rural China<br>Ages: 6−11<br>Age; sex; BMI; paternal educational level; maternal educational level; household income; abnormal birth; maternal age at delivery	Section 3.1, Table 3

© 2025 Taylor KW et al. *JAMA Pediatrics*.

Reference[a] Study Design Location *Risk-of-bias Determination*	Fluoride Exposure		Mean-effects Meta-analysis N, Mean (SD) [Reference] [Exposed]	Dose-response Mean-effects Meta-analysis N, Mean (SD) [Reference] [Exposed]	Regression Slopes Meta-analysis Slope (SE) or 95% CI per Unit Change Fluoride	Intelligence Assessment Method Age Range (Years) Confounders Considered	Source in Study
	Assessment (Metric, Exposure Groups)	Levels					
Ahmad et al. (2022)[99, me, w, u]<br>Cross-sectional<br>Pakistan<br>*High*	Urine, drinking water<br>Reference/high fluoride areas	Urine: 3.53 ± 1.09 mg/L (reference)<br>5.99 ± 3.57 mg/L (high)<br>Water: 1.07 mg/L (reference)<br>2.04 mg/L (high)	60, 91.9 (14.96)<br>60, 98.55 (15.71)<br>Boys:<br>48, 92.3 (14.97)<br>38, 99.5 (15.50)<br>Girls:<br>12, 90.30 (15.49)<br>22, 96.90 (16.31)	60, 91.9 (14.96)<br>60, 98.55 (15.71)		Raven's Progressive Matrices Intelligence Test<br>Ages: 9−11<br>Sex	Calculated by ICF, Table 5.
Feng et al. (2022)[100, me, rs, u]<br>Cross-sectional<br>China<br>*Low*	Urine<br>Reference/high fluoride areas	0.83 ± 0.30 mg/L (reference)<br>2.15 ± 0.91 mg/L (high)	342, 121.50 (12.14)<br>341, 122.61 (11.61)	342, 121.50 (12.14)<br>341, 122.61 (11.61)	−0.54 (−1.79, 0.71) per 1 mg/L urinary F	Combined Raven's Test for Rural China<br>Ages: 8−12<br>Age; gender; BMI; age at which pregnancy occurred; gestational weeks; birth weight; birth modes; paternal and maternal education	Table 1, Figure 1, Calculated by ICF
Goodman et al. (2022a)[101, rs]<br>Prospective cohort<br>Mexico<br>*Low*	Maternal urine	0.89 (0.61, 1.10) mg/L[d]			−2.01 (−3.66, −0.46) per 0.5 mg/L urinary F	Wechsler Abbreviated Scale of Intelligence<br>Ages: 6−12<br>Age; sex; weight at birth; parity; gestational age; maternal characteristics (smoking history, marital status, age at delivery, education, cohort)	Supplemental Table 2
Tian et al. (2022)[102, me, o, rs]<br>Cross-sectional<br>China<br>*High*	Urine<br>Reference (no dental fluorosis)/dental fluorosis	1.53 ± 0.98 mg/L (reference)<br>2.14 ± 1.78 mg/L (dental fluorosis)	69, 100.38 (11.87)<br>104, 92.33 (11.68)		−2.58 (−5.48, −0.24) per 1 mg/L urinary F	Combined Raven's Test for Rural China<br>Ages: 10–13<br>–	Table 1, Abstract

© 2025 Taylor KW et al. *JAMA Pediatrics*.

Reference[a] Study Design Location *Risk-of-bias Determination*	Fluoride Exposure		Mean-effects Meta-analysis N, Mean (SD) [Reference] [Exposed]	Dose-response Mean-effects Meta-analysis N, Mean (SD) [Reference] [Exposed]	Regression Slopes Meta-analysis Slope (SE) or 95% CI per Unit Change Fluoride	Intelligence Assessment Method Age Range (Years) Confounders Considered	Source in Study
	Assessment (Metric, Exposure Groups)	Levels					
Dewey et al. (2023)[103, me]<br>Prospective ecological cohort<br>Canada<br>*Low*	Water<br>Not exposed/partially exposed/fully exposed to fluoridated drinking water during pregnancy	Not specified	101, 104.62 (11.41)<br>220, 103.92 (12.50)<br>295, 104.69 (14.02)<br>Boys:<br>45, 103.16 (13.73)<br>114, 103.29 (11.23)<br>157, 102.59 (14.97)<br>Girls:<br>56, 105.80 (9.1)<br>106, 104.59 (13.77)<br>138, 107.09 (12.48)			Wechsler Preschool and Primary Scale of Intelligence, 4th Edition: Canadian (WPPSI-IV CND)<br>Ages: 3−5<br>Sex	Author correspondence
Grandjean et al. (2023)[104, rs]<br>Prospective cohort<br>Denmark<br>*Low*	Maternal urine	0.58 ± 0.32 mg/L			0.18 (−1.39, 1.76) per 1 $\log_2$ mg/L urinary F<br>−0.40 (−2.52, 1.71) per 1 $\log_2$ mg/L urinary F (girls)<br>0.87 (−1.41, 3.15) per 1 $\log_2$ mg/L urinary F (boys)	Wechsler Intelligence Scales for Children, Danish version<br>Ages: 7<br>Parental education; preterm birth; age at the time of testing; examiner; breastfeeding duration; school grade; school type; smoking status; alcohol consumption	Table 2, Results section
Lin et al. (2023)[105, me, u*, rs]<br>Cross-sectional<br>Taiwan<br>*Low*	Urine<br>Reference (no dental fluorosis)/dental fluorosis	0.38 ± 0.25 mg/L (reference)<br>0.46 ± 0.32 mg/L (dental fluorosis)	429, 97.72 (15.09)<br>133, 99.22 (15.87)	429, 97.72 (15.09)<br>133, 99.22 (15.87)	−0.97 (−2.90, 0.97) per 1 ln mg/L urinary F	Raven's Colored Progressive Matrices-Parallel, Taiwan edition<br>Ages: 6−12<br>Age; area of school; BMI; sex; history of allergic rhinitis; parental education	Table 1, Table 4

© 2025 Taylor KW et al. *JAMA Pediatrics*.

Reference[a] Study Design Location *Risk-of-bias Determination*	Fluoride Exposure		Mean-effects Meta-analysis N, Mean (SD) [Reference] [Exposed]	Dose-response Mean-effects Meta-analysis N, Mean (SD) [Reference] [Exposed]	Regression Slopes Meta-analysis Slope (SE) or 95% CI per Unit Change Fluoride	Intelligence Assessment Method Age Range (Years) Confounders Considered	Source in Study
	Assessment (Metric, Exposure Groups)	Levels					
Xia et al. (2023)[106, me, w ,u, rs]<br><br>Cross-sectional<br>China<br>*Low*	Urine, drinking water<br><br>Reference/high fluoride area	Urine: 0.98 (0.33, 3.59) (reference)[g]<br>2.63 (0.57, 8.84) (high fluoride area)<br><br>Water: 0.73 ± 0.08 mg/L (reference)<br>1.89 ± 0.17 mg/L (high fluoride area)	358, 110 (12.3)<br>353, 105 (16.3)	358, 110 (12.3)<br>353, 105 (16.3)	1.42 (−0.47, 3.32) per 1 mg/L urinary F (reference)<br><br>−4.08 (−3.04, −1.32) per 1 mg/L urinary F (high F area)	Combined Raven's Test for Rural China<br>Ages: 8−12<br>Age; sex; BMI; parental education	Tables 1,2, 5

Notes:

Publications excluded from the *mean-effect meta-analyses* and/or used in sensitivity analyses can be found in **eTable 2**.

COMT = catechol-O-methyltransferase; GCI = General Cognitive Index; HOME = Home Observation for Measurement of the Environment; MDI = Mental Development Index; RoB = risk of bias; SD = standard deviation; SE = standard error; SES = socioeconomic status

[a]The superscript "me" indicates studies included in the *mean-effects meta-analysis*; the superscript "o" indicates studies with "other" exposure matrices besides water and dental fluorosis, including non-drinking water sources of fluoride such as coal burning; the superscript "w" indicates studies included in the *dose-response mean-effects meta-analysis* using fluoride in water; the superscript "u" indicates studies included in the *dose-response mean-effects meta-analysis* using fluoride in urine; the asterisk "*" indicates studies included in the *dose-response mean-effects meta-analysis* at levels < 1.5 mg/L; the superscript "rs" indicates studies included in the *regression slopes meta-analysis*; the superscript "sa" indicates studies only included in sensitivity analyses. Studies are presented in chronological order; effect estimates and 95% CIs used in meta-analyses are presented in **Figure 1**.

[b]Additional exposure regions including iodine levels were not included in the analysis.

[c]Additional exposure regions including arsenic levels were not included in the analysis.

[d]Median (q1−q3).

[e]Overlapping population with Green et al. (2019),[89] but smaller sample size; excluded from overall *regression slopes meta-analysis* but used in *regression slopes meta-analysis* by exposure matrix and in a sensitivity analysis.

[f]Overlapping population with Zhao et al. (2018),[88] but smaller sample size; excluded from overall *mean-effects meta-analysis* but used in mean-effects subgroup meta-analysis by age group.

[g]Median (min, max).

© 2025 Taylor KW et al. *JAMA Pediatrics*.

eTable 2. List of Excluded Studies from Mean-Effects Meta-Analysis

Reference, Country	Reason for Exclusion
Qin et al. (1990)[107] [translated in Qin et al. 2008], China	Missing mean or SD of outcome measure
Yang et al. (1994)[109] [translated in Yang et al. 2008], China	Overlapping population with Wang et al. (2001)[67]; Table 2 in Yang et al. (1994)[109] seemed incomplete
Wang et al. (2005b)[111] [translated in Wang et al. 2008a], China	Missing mean or SD of outcome measure
Rocha-Amador et al. (2007)[112], Mexico	Missing mean or SD of outcome measure
Liu et al. (2000)[113] [translated in Liu et al. 2008], China	Overlapping population with Lu et al. (2000)[63]
Sudhir et al. (2009)[115], India	Missing mean or SD of outcome measure
He and Zhang (2010)[116], China	Missing mean or SD of outcome measure
Xiang et al. (2011)[117], China	Overlapping population with Xiang et al. (2003a)[70]
Saxena et al. (2012)[118], India	Missing mean or SD of outcome measure
Wang et al. (2012)[119], China	Overlapping population with Xiang et al. (2003a)[70]
Nagarajappa et al. (2013)[120], India	Seguin Foam Board test; due to the test measuring eye-hand coordination and cognitive-perceptual abilities
Pratap et al.(2013)[121], India	Missing mean or SD of outcome measure
Asawa et al. (2014)[122], India	Seguin Foam Board test; due to the test measuring eye-hand coordination and cognitive-perceptual abilities
Wei et al. (2014)[123], China	Missing mean or SD of outcome measure
Choi et al. (2015)[124], China	Cognitive functions other than IQ
Kundu et al. (2015)[79], India	Unusual IQ scores based on Raven's Standardized Progressive Matrices Test; used only for sensitivity analysis for the *mean-effects meta-analysis*
Aravind et al. (2016)[82], India	Unusually low IQ scores Raven's Standardized Progressive Matrices Test; used only for sensitivity analysis for the *mean-effects meta-analysis*
Jin et al.(2016)[125], China	Cognitive functions other than IQ; potential overlap with Zhang et al. (2015c)[81]
Kumar et al. (2016)[126], India	Seguin Foam Board test; due to the test measuring eye-hand coordination and cognitive-perceptual abilities
Bashash et al. (2017)[84], Mexico	Overlap with Goodman et al. (2022a)[101]; used only for sensitivity analysis for the *mean-effects meta-analysis, dose-response meta-analysis* and *regression slopes meta-analysis*
Jin et al.(2017)[127], China	Overlap with Jin et al. (2016)[125]; unusual IQ scores reported as percentiles
Razdan et al. (2017)[85], India	Unusually low IQ scores based on Raven's Standardized Progressive Matrices Test; used only for sensitivity analysis for the *mean-effects meta-analysis*
Valdez Jiménez et al. (2017)[86], Mexico	Bayley tests; used only for sensitivity analysis for the *regression slopes meta-analysis*

© 2025 Taylor KW et al. *JAMA Pediatrics*.

Reference, Country	Reason for Exclusion
Wang et al. (2017)[128], China	Overlapping population with Xiang et al. (2003a)[70]
Cui et al. (2018)[87], China	Missing mean or SD of outcome measure; used in *regression slopes meta-analysis*
Luo et al. (2018)[129], China	Overlapping population with Lou et al. (2021)[35]
Naik et al. (2018)[130], India	Missing sample sizes by exposure groups. Missing mean and SD for IQ scores
Sharma et al.(2018)[131], India	Missing mean and SD for IQ scores
Soto-Barreras et al. (2019)[132], Mexico	Missing mean or SD of IQ grade based on Raven's Standardized Progressive Matrices Test
Zhao et al. (2019)[133], China	Overlapping population with Yu et al. (2018)[5], but smaller sample size
Zhou et al. (2019)[134], China	Overlapping population with Yu et al. (2018)[5], but smaller sample size
Till et al.(2020)[91], Canada	Missing mean or SD of outcome measure; overlapping population with Green et al. (2019),[89] but smaller sample size; excluded from overall *regression slopes meta-analysis* but used in regression slopes subgroup meta-analysis by exposure matrix and in a sensitivity analysis
Wang et al. (2020b)[4], China	Missing mean or SD of outcome measure; used in sensitivity analysis for the *regression slopes meta-analysis*
Xu et al. (2020)[135], China	Study retracted by journal. Overlapping population with Feng et al. (2022)[100]
Zhao et al. (2020)[136], China	Overlapping population with Yu et al. (2018)[5], but smaller sample size
Aggeborn and Öhman (2021)[137], Sweden	Cognitive functions other than IQ; cognitive test not specified
Cantoral et al. (2021)[92], Mexico	Bayley tests; used only for sensitivity analysis for the *regression slopes meta-analysis*
Farmus et al. (2021)[138], Canada	Same data as Till et al.(2020)[91]
Guo et al. (2021)[93], China	Overlapping population with Zhao et al. (2018)[88], but smaller sample size; excluded from overall *mean-effects meta-analysis* but used in mean-effects subgroup meta-analysis by age group
Ibarluzea et al. (2021)[94], Spain	Bayley and McCarthy tests; used only for sensitivity analysis for the *mean-effects meta-analysis*, *dose-response meta-analysis*, and *regression slopes meta-analysis*
Prabhakar et al. (2021)[95], India	Unusual IQ scores based on Raven's Standardized Progressive Matrices Test; used only for sensitivity analysis for the *mean-effects meta-analysis*
Wang et al. (2021b)[139], China	Overlapping population with Wang et al. (2021)[97]; cognitive functions other than IQ
Wang et al. (2021c)[140], China	Overlapping population with Yu et al. (2018)[5], but smaller sample size
Yani et al. (2021)[141], Indonesia	Missing mean and SD for IQ scores
Yu et al. (2021)[142], China	Overlapping population with Yu et al. (2018)[5], but smaller sample size

© 2025 Taylor KW et al. *JAMA Pediatrics*.

Reference, Country	Reason for Exclusion
Zhao et al. (2021)[98], China	Missing mean or SD of outcome measure; used in *regression slopes meta-analysis*
Zhou et al. (2021)[143], China	Overlapping population with Yu et al. (2018)[5], but smaller sample size
Goodman et al. (2022a)[101], Mexico	Missing mean or SD of outcome measure; used in *regression slopes meta-analysis*
Goodman et al. (2022b)[144], Canada	Overlapping population with Green et al. (2019)[89]
Kaur et al. (2022)[145], India	IQ based on Raven's Colored Progressive Matrices Test reported as percentiles rather than scores
Tamayo De la Cruz et al. (2022)[146], Dominican Republic	Missing mean and SD for IQ scores; age range 13–23 years
Grandjean et al. (2023)[104], Denmark	Missing mean or SD of outcome measure; used in *regression slopes meta-analysis*

© 2025 Taylor KW et al. *JAMA Pediatrics*.

eFigure 2. Results From Risk-of-Bias Evaluations for Studies Included in the Meta-Analyses and Sensitivity Analyses

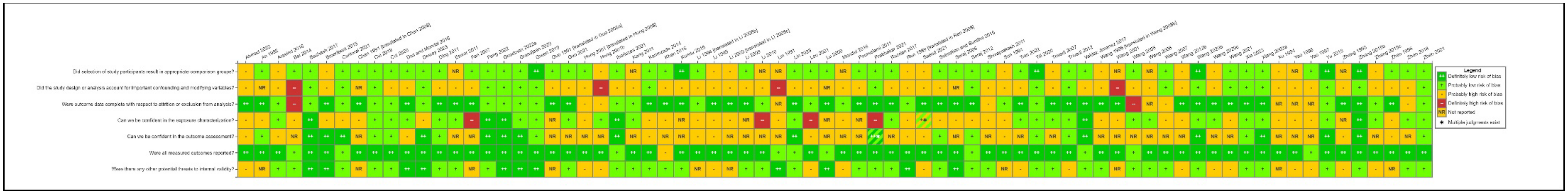

eFigure 2a. Results From Risk-of-Bias Evaluations for All Studies Included in the Meta-Analyses and Sensitivity Analyses[a]

[a]An interactive version of **eFigure 2a** is available here: https://hawcproject.org/summary/visual/assessment/405/eFigure-2-Meta-analysis-RoB/.

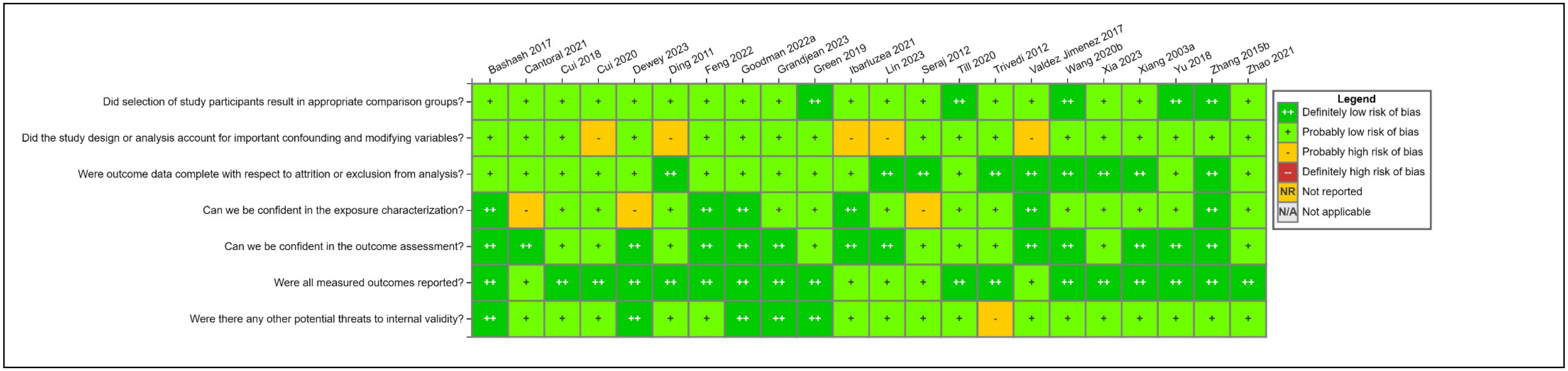

eFigure 2b. Results From Risk-of-bias Evaluations for Low Risk-of-Bias Studies Included in the Meta-Analyses and Sensitivity Analyses[a]

[a]An interactive version of **eFigure 2b** is available here: https://hawcproject.org/summary/visual/assessment/405/eFigure-2b-Meta-analysis-RoB-low-RoB-studies/.

The following low risk-of-bias studies are included in the *mean-effects meta-analysis* and *dose-response mean-effects meta-analysis*: Cui et al. (2020),[90] Ding et al. (2011),[76] Feng et al. (2022),[100] Goodman et al. (2022a),[101] Green et al. (2019),[89] Lin et al. (2023),[105] Seraj et al. (2012),[46] Trivedi et al. (2012),[56] Xia et al. (2023),[106] Xiang et al. (2003a),[70] Yu et al. (2018),[5] and Zhang et al.(2015b).[80] Dewey et al. (2023)[103] is also included in the *mean-effects meta-analysis* but not the *dose-response mean-effects meta-analysis*.

The following low risk-of-bias studies are included in the *regression slopes meta-analysis*: Cui et al. (2018),[87] Ding et al. (2011),[76] Feng et al. (2022),[100] Goodman et al. (2022a),[101] Grandjean et al. (2023),[104] Green et al. (2019),[89] Lin et al. (2023),[105] Xia et al. (2023),[106] Yu et al. (2018),[5] Zhang et al.(2015b),[80] and Zhao et al. (2021).[98]

Five low risk-of-bias studies are only included in sensitivity analyses. All five of these studies are included in sensitivity analyses for the *regression slopes meta-analysis* and include Bashash et al. (2017),[84] Cantoral et al. (2021),[92] Ibarluzea et al. (2021),[94] Valdez Jiménez et al. (2017),[86] and Wang et al. (2020b).[4] Bashash et al. (2017)[84] and Ibarluzea et al. (2021)[94] are also included in sensitivity analyses for the *mean-effects meta-analysis* and *dose-response mean-effects meta-analysis*. Till et al. (2020)[91] was excluded from the overall *regression slopes meta-analysis* but used in regression slopes subgroup meta-analysis by exposure matrix and in a regression slopes sensitivity analysis.

© 2025 Taylor KW et al. *JAMA Pediatrics*.

Mean-Effects Meta-Analysis

Fifty-two studies show inverse associations between fluoride exposure and IQ scores in children. The seven studies with non-negative associations reported SMD estimates of 0.01 (95% CI: −0.19, 0.21),[89] 0.01 (95% CI: −0.19, 0.22),[41] 0.43 (95% CI: 0.07, 0.80),[99] 0.09 (5% CI: −0.06, 0.24),[100] 0.00 (95% CI: −0.25, 0.25),[77] 0.01 (−0.22, 0.23),[103] and 0.10 (−0.10, 0.29).[105] Two of the seven studies with non-negative SMDs compared mean IQs for children living in fluoridated versus non-fluoridated areas in Canada[89] or New Zealand.[41] No other studies included in the main *mean-effects meta-analysis* made comparisons between fluoridated and non-fluoridated areas. In both studies, levels of fluoride in water were low, even in communities with fluoridated drinking water, likely limiting the power to detect an effect. Similarly, one study[103] examined whether maternal exposure to fluoride during pregnancy was associated with children's intelligence at 3–5 years of age in a community where fluoride stopped being added to the water supply at the recommended 0.7 mg/L, after which fluoride levels were not adjusted and varied between 0.1 and 0.4 mg/L. The very low levels of fluoride and lack of contrast across comparison groups potentially limited the ability to detect an effect for IQ, although associations were observed for poor inhibitory control and with poorer cognitive flexibility in girls.

We conducted several sensitivity analyses. In Bashash et al. (2017),[84] the SMD compares mean IQ scores in children with urinary fluoride levels <0.80 mg/L versus children with levels ≥0.80 mg/L in Mexico.[84] Unlike other studies in the *mean-effects meta-analysis* that compared mean IQ scores between fluoridated and non-fluoridated areas or between high fluoride and low fluoride areas (**eTable 1**), the Bashash et al. (2017)[84] study was not designed to measure fluoride by geographical area. However, since the mean IQ scores were provided in the study for children with urinary fluoride levels <0.80 mg/L and ≥0.80 mg/L, we included the study in the sensitivity analysis (**eTable 3)**.

We conducted several additional sensitivity analyses to evaluate the impact of: an outlier study[50]; a study with effect estimates that had to be derived from an original publication[61]; studies with unspecified IQ tests[28 [translated in Li et al. 2008b], 56]; studies with unusual IQ scores[79, 82, 85, 95]; a study reporting the cognitive subset of evaluations using Bayley and McCarthy tests,[94] or combining all exposed groups and comparing them to the reference group (**eTable 3)**.

We conducted subgroup analyses to evaluate potential sources of heterogeneity. Although we conducted subgroup analyses by sex, only 2 of the 16 studies that reported IQ scores separately for boys and girls analyzed fluoride exposure for each sex separately.[89, 103] This is essential for evaluating whether a differential change in IQ by sex may be related to higher susceptibility or higher exposure in that sex. With a couple of exceptions, the subgroup analyses in the *mean-effects meta-analysis* did not explain a large amount of the overall heterogeneity. However, the heterogeneity in the *regression slopes meta-analysis*, which included mostly low risk-of-bias studies, was explained by subgroup analyses. This suggests that the aggregate nature of the *mean-effects meta-analysis* might not be sufficiently sensitive to capture potential sources of heterogeneity as seen when using studies with individual-level data in the *regression slopes meta-analysis*. However, the large number of studies included in the *mean-effects meta-analysis* and the consistency in the direction of the association across the analysis make this less of a concern.

To further evaluate potential sources of heterogeneity, we conducted meta-regression analyses using mean age in years (from the age range reported in each study) and year of publication. The results of the meta-regression models of the mean-effects meta-analysis indicate that year of publication and mean age of study children did not explain a large degree of heterogeneity as neither were significant predictors of the relationship between fluoride exposure and children's intelligence, and the residual I^2 remained high (85% and 88%, respectively). Year of publication (SMD = 0.01, 95% CI: −0.004, 0.02) and mean age (SMD = −0.04, 95% CI: −0.13, 0.05) explained relatively little between-study variance (adjusted R^2 of

13% and 3%, respectively). When both year of publication and mean age were included in the model, there were minor improvements to the amount of between-study variance explained (adjusted R^2 = 12%) or percent residual variation due to heterogeneity (residual I^2 = 85%).

Excluding the outlier study[50] resulted in a slightly lower heterogeneity for the overall mean-effects meta-analysis effect estimate (I^2 = 87%) and for the India-specific effect estimate (I^2 = 69%). The meta-regression indicates that year and age were significant predictors of the effect (SMD = 0.01, 95% CI: 0.005, 0.02, p-value = 0.002 and SMD = −0.06, 95% CI: −0.11, −0.01, p-value = 0.026, respectively), explaining 24% and 11% of the between-study variance, respectively. When both year of publication and mean age were included in the model, there were notable improvements to the amount of between-study variance explained (adjusted R^2 = 29%) or percent residual variation due to heterogeneity (residual I^2 = 80%).

© 2025 Taylor KW et al. *JAMA Pediatrics*.

Mean-Effects Meta-Analysis Sensitivity Analyses

eTable 3. Sensitivity Analyses for Mean-Effects Meta-Analysis: Pooled SMDs and 95% CIs for Children's IQ Score and Exposures to Fluoride

Analysis	Number of Studies	SMD (95% CI)	Heterogeneity p-value	Heterogeneity I^2
**Overall analysis**				
IQ	59	−0.45 (−0.57, −0.33)	<0.001	94%
**Sensitivity analyses**				
All exposed groups vs. reference group				
	59	−0.41 (−0.52, −0.30)	<0.001	93%
Excluding Khan et al. (2015)[50]				
	58	−0.39 (−0.48, −0.31)	<0.001	87%
Excluding Lin et al. (1991)[61]				
	58	−0.45 (−0.58, −0.33)	<0.001	94%
Excluding Li et al. (1994)[28] [translated in Li et al. 2008b]				
	58	−0.44 (−0.57, −0.32)	<0.001	94%
Excluding Trivedi et al. (2012)[56]				
	58	−0.45 (−0.57, −0.33)	<0.001	94%
Low risk of bias studies, excluding Trivedi et al. (2012)[56]				
	11	−0.19 (−0.35, −0.03)	<0.001	88%
Including Ibarluzea et al. (2021),[94] Bayley MDI score				
	60	−0.44 (−0.56, −0.32)	<0.001	94%
Including Ibarluzea et al. (2021),[94] McCarthy GCI score				
	60	−0.44 (−0.56, −0.32)	<0.001	94%
Including Aravind et al. (2016),[82] Kundu et al. (2015),[79] Razdan et al. (2017),[85] Bashash et al. (2017),[84] Prabhakar et al. (2021)[95]				
	64	−0.51 (−0.68, −0.34)	<0.001	97%
Including Aravind et al. (2016),[82] Kundu et al. (2015),[79] Razdan et al. (2017),[85] Bashash et al. (2017),[84] Prabhakar et al. (2021),[95] Ibarluzea et al. (2021),[94] Bayley MDI score				
	65	−0.50 (−0.67, −0.33)	<0.001	97%
Including Aravind et al. (2016),[82] Kundu et al. (2015),[79] Razdan et al. (2017),[85] Bashash et al. (2017),[84] Prabhakar et al. (2021),[95] Ibarluzea et al. (2021),[94] McCarthy GCI score				
	65	−0.50 (−0.67, −0.33)	<0.001	97%

Notes:

CI = confidence interval; SMD = standardized weighted mean difference; MDI = Mental Development Index; GCI = General Cognitive Index.

© 2025 Taylor KW et al. *JAMA Pediatrics*.

Tests Assessing Potential Publication Bias in Studies With Group-Level Exposures

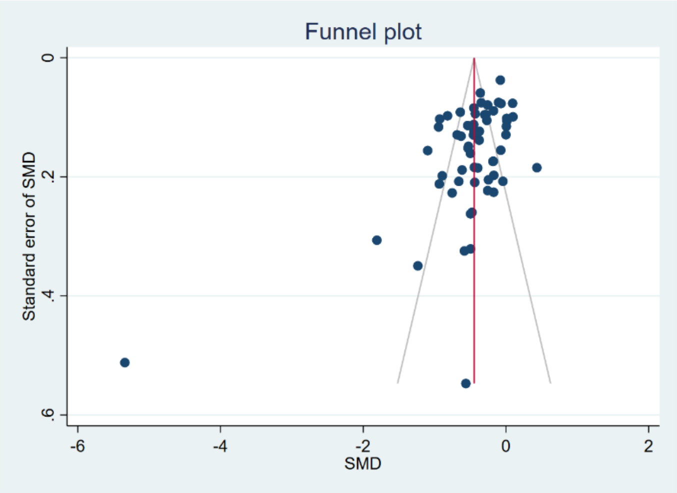

eFigure 3. Funnel Plot of Included Studies

This funnel plot shows individual studies included in the analysis according to random-effect SMD estimates (x-axis) and the SE of each study-specific SMD (y-axis). The solid vertical line indicates the pooled SMD estimate for all studies combined and the dashed lines indicate pseudo 95% confidence limits around the pooled SMD estimate.

```
Regression-based Egger test for small-study effects
Random-effects model
Method: REML

H0: beta1 = 0; no small-study effects
            beta1 =     -3.44
      SE of beta1 =     0.662
                z =     -5.20
       Prob > |z| =    0.0000

Begg's test for small-study effects

Kendall's score =   -331.00
    SE of score =   152.917
              z =     -2.17
     Prob > |z| =    0.0309
(68 missing values generated)
```

eFigure 4. Test for Publication Bias

© 2025 Taylor KW et al. *JAMA Pediatrics*.

```
Nonparametric trim-and-fill analysis of publication bias
Run estimator, imputing on the right

Iteration                        Number of studies =     61
  Model: Random-effects                   observed =     59
 Method: REML                             imputed =       2

Pooling
  Model: Random-effects
 Method: REML

---------------------------------------------------------------
             Studies |       Cohen's d   [95% conf. interval]
---------------------+-----------------------------------------
            Observed |          -0.447     -0.567     -0.326
  Observed + Imputed |          -0.392     -0.584     -0.201
---------------------------------------------------------------
```

```
Nonparametric trim-and-fill analysis of publication bias
Linear estimator, imputing on the left

Iteration                        Number of studies =     76
  Model: Random-effects                   observed =     59
 Method: REML                             imputed =      17

Pooling
  Model: Random-effects
 Method: REML

---------------------------------------------------------------
             Studies |       Cohen's d   [95% conf. interval]
---------------------+-----------------------------------------
            Observed |          -0.447     -0.567     -0.326
  Observed + Imputed |          -0.632     -0.759     -0.504
---------------------------------------------------------------
```

eFigure 5. Trim-and-Fill Analysis

Left panel shows the random-effects pooled SMD after filling in to the right using a run estimator (the linear estimator to the right showed no change in pooled SMD); right panel shows random-effects pooled SMD after filling in to the left using a linear estimator (the run estimator to the left showed no change in pooled SMD).

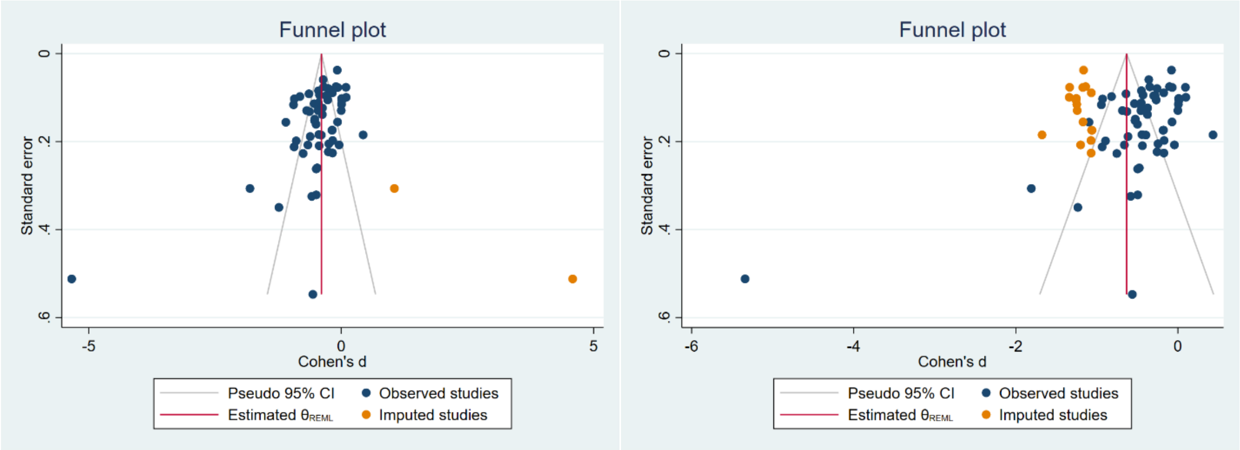

eFigure 6. Filled-In Funnel Plots to Eliminate Publication Bias

Left panel shows the funnel plot filled in to the right using a run estimator (the linear estimator to the right showed no change in pooled SMD); right panel shows the funnel plot filled in to the left using a linear estimator (the run estimator to the left showed no change in pooled SMD).

© 2025 Taylor KW et al. *JAMA Pediatrics*.

Forest plots for the Random Effects Meta-Analysis of Mean Effects: Subgroup Analyses

Risk-of-bias Subgroup Analysis

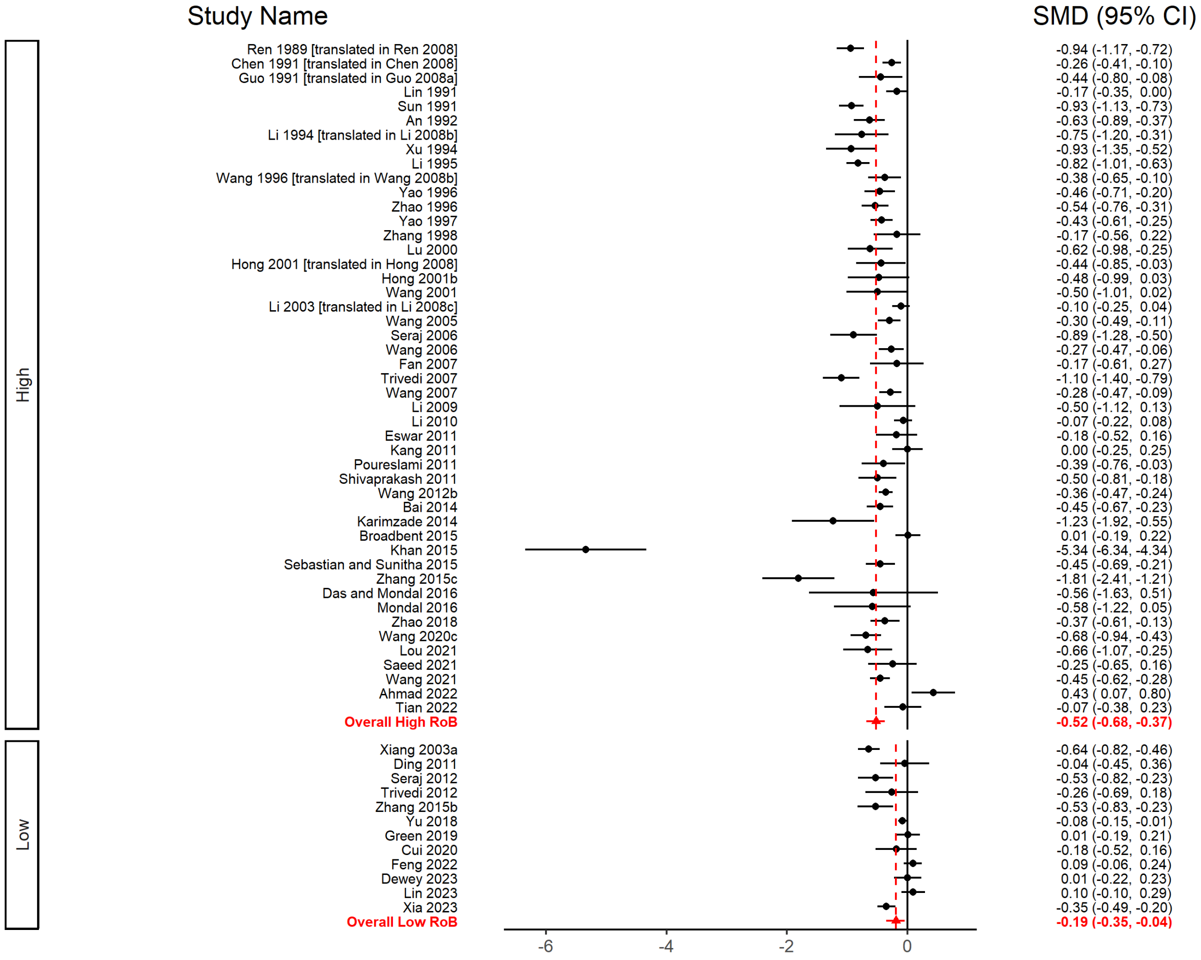

eFigure 7. Association Between Fluoride Exposure and IQ Scores in Children: Effect by Risk of Bias

© 2025 Taylor KW et al. *JAMA Pediatrics*.

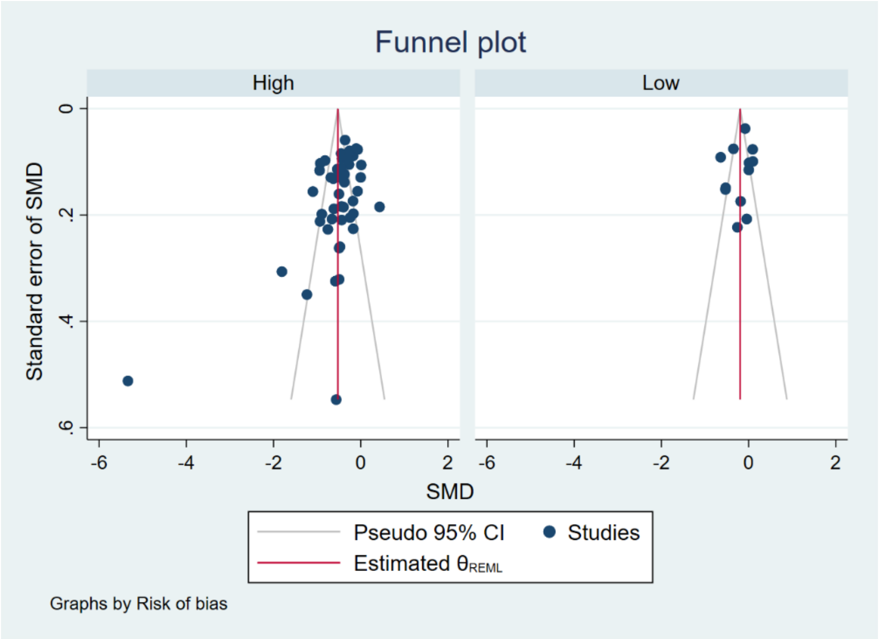

eFigure 8. Funnel Plot by Risk-of-Bias Evaluation

```
Regression-based Egger test for small-study effects
Random-effects model
Method: REML
                                               High RoB

H0: beta1 = 0; no small-study effects
            beta1 =      -3.62
      SE of beta1 =      0.757
                z =      -4.78
       Prob > |z| =     0.0000
                                               Low RoB

H0: beta1 = 0; no small-study effects
            beta1 =      -0.90
      SE of beta1 =      1.558
                z =      -0.58
       Prob > |z| =     0.5635
```

eFigure 9. Test for Publication Bias by Risk of Bias

© 2025 Taylor KW et al. *JAMA Pediatrics*.

```
Nonparametric trim-and-fill analysis of publication bias
Run estimator, imputing on the right

Iteration                          Number of studies =     48
  Model: Random-effects                     observed =     47
 Method: REML                               imputed  =      1

Pooling
  Model: Random-effects
 Method: REML
```

Studies	Cohen's d	[95% conf.	interval]
Observed	-0.524	-0.678	-0.371
Observed + Imputed	-0.474	-0.716	-0.232

```
Nonparametric trim-and-fill analysis of publication bias
Linear estimator, imputing on the left

Iteration                          Number of studies =     63
  Model: Random-effects                     observed =     47
 Method: REML                               imputed  =     16

Pooling
  Model: Random-effects
 Method: REML
```

Studies	Cohen's d	[95% conf.	interval]
Observed	-0.524	-0.678	-0.371
Observed + Imputed	-0.755	-0.912	-0.597

eFigure 10. Trim-and-Fill Analysis for High Risk-of-Bias Studies

Filling in to the right using a linear estimator or to the left using a run estimator showed no change in the pooled SMD.

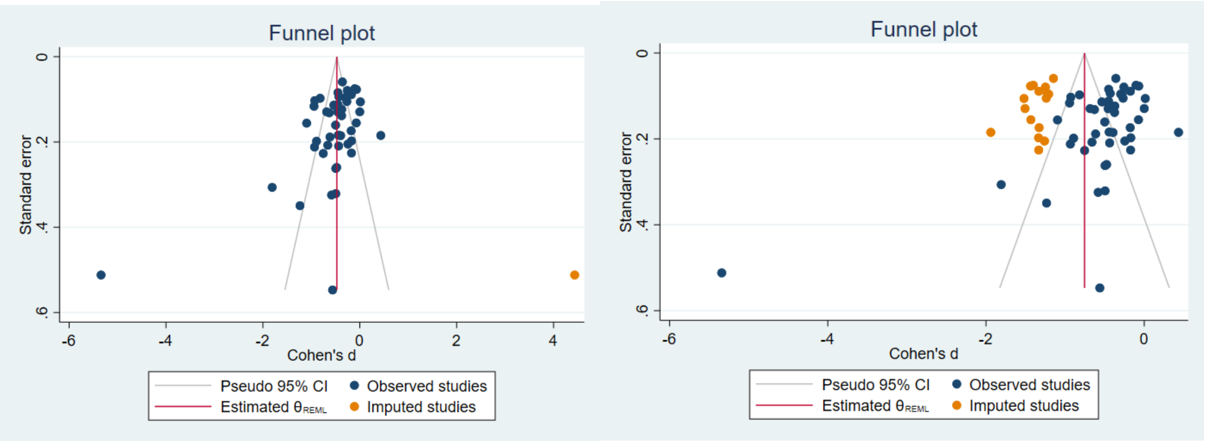

eFigure 11. Filled-In Funnel Plots for High Risk-of-Bias Studies

Left panel shows the random-effects pooled SMD after filling in to the right using a run estimator (the linear estimator to the right showed no change in the pooled SMD); right panel shows random-effects pooled SMD after filling in to the left using a linear estimator (the run estimator to the left showed no change in the pooled SMD).

© 2025 Taylor KW et al. *JAMA Pediatrics*.

Sex Subgroup Analysis

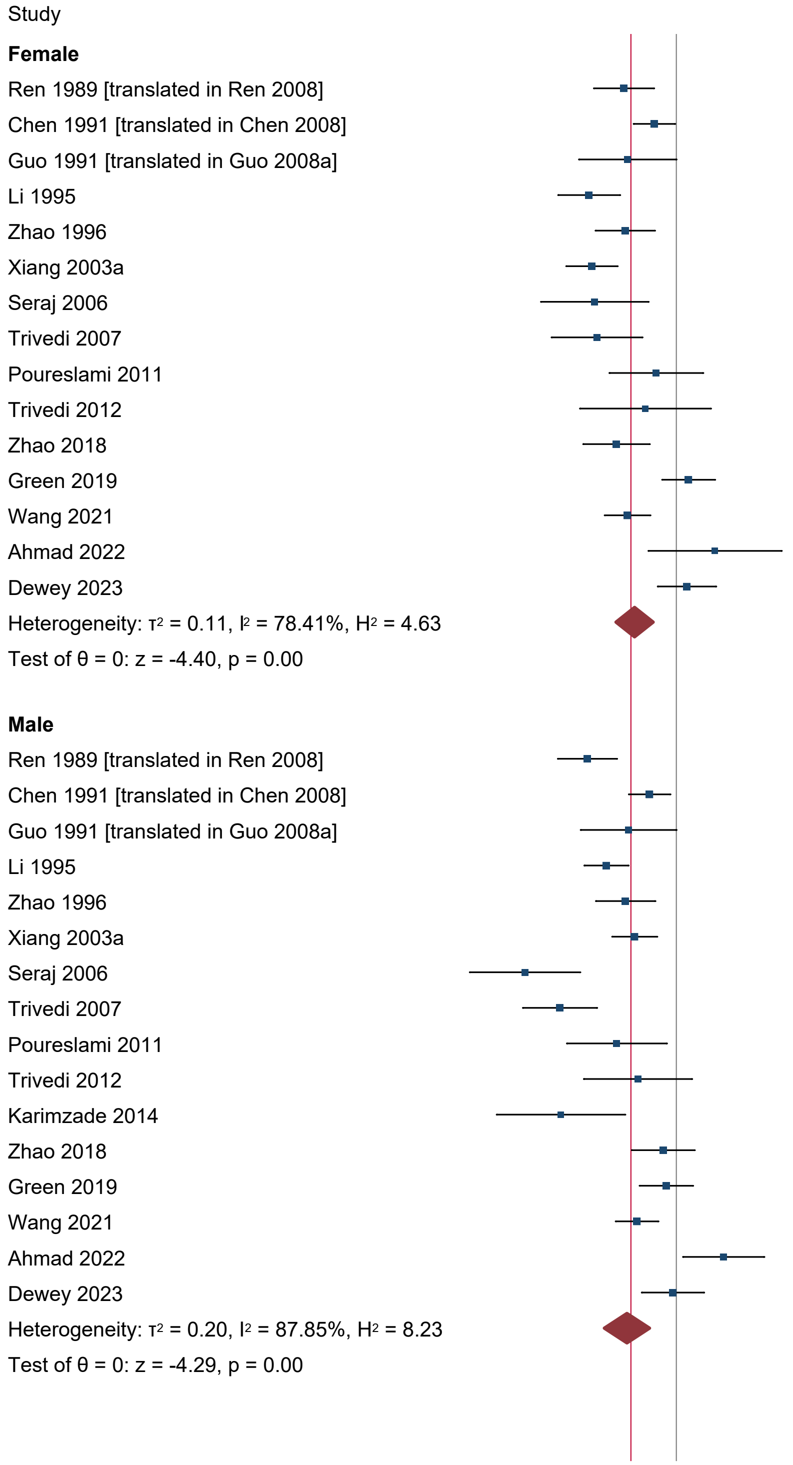

eFigure 12. Association Between Fluoride Exposure and IQ Scores in Children: Effect by Sex

© 2025 Taylor KW et al. *JAMA Pediatrics*.

Age Group Subgroup Analysis

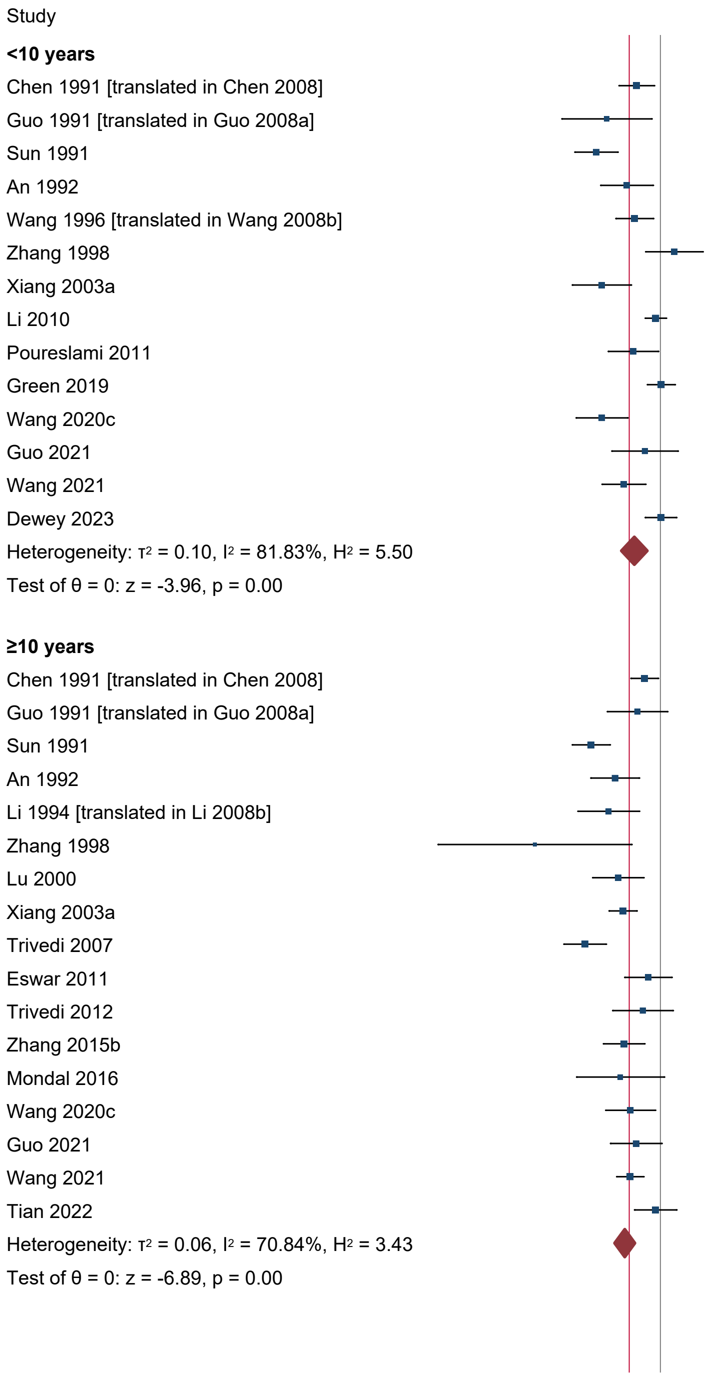

eFigure 13. Association Between Fluoride Exposure and IQ Scores in Children: Effect by Age Group

© 2025 Taylor KW et al. *JAMA Pediatrics*.

Country Subgroup Analysis

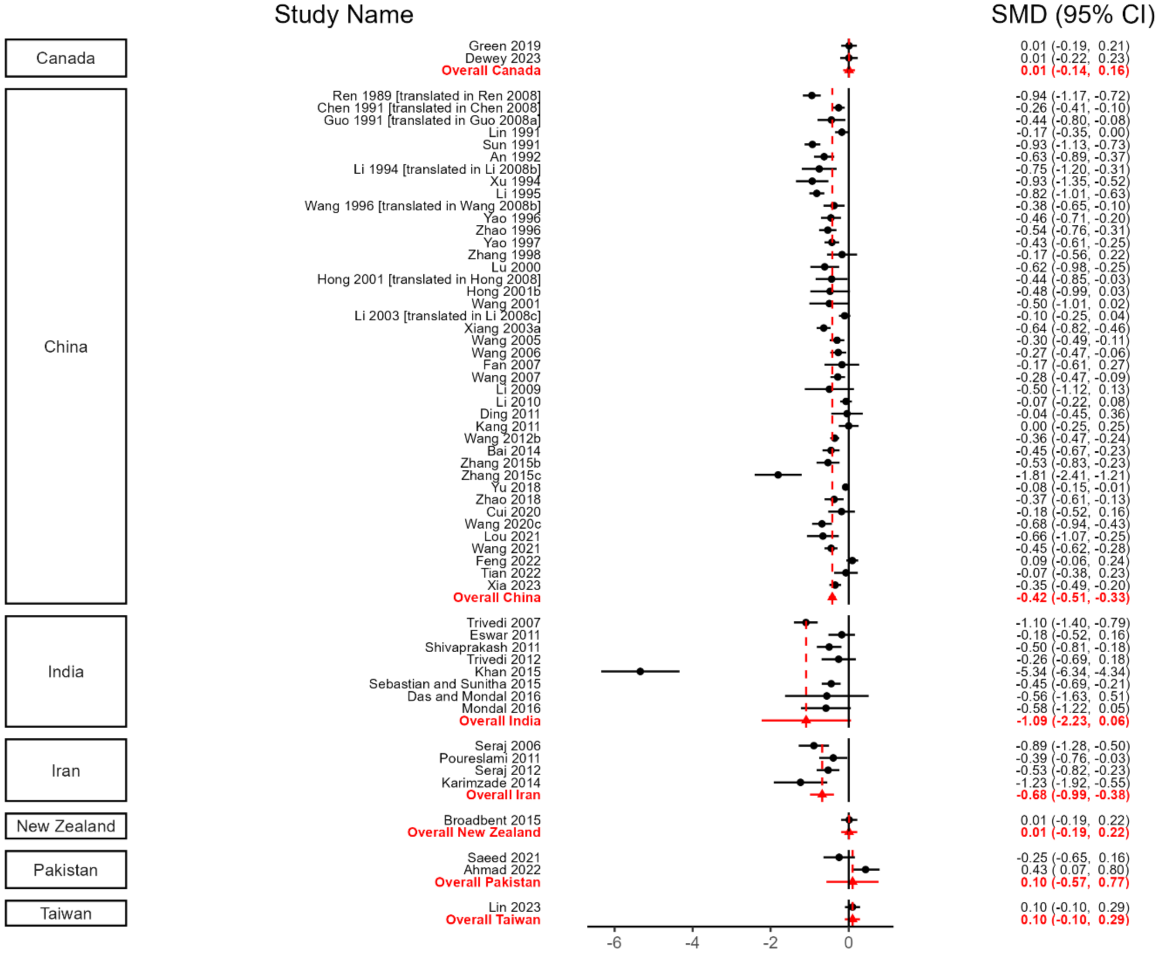

eFigure 14. Association Between Fluoride Exposure and IQ Scores in Children: Effect by Country

© 2025 Taylor KW et al. *JAMA Pediatrics*.

Outcome Assessment Type Subgroup Analysis

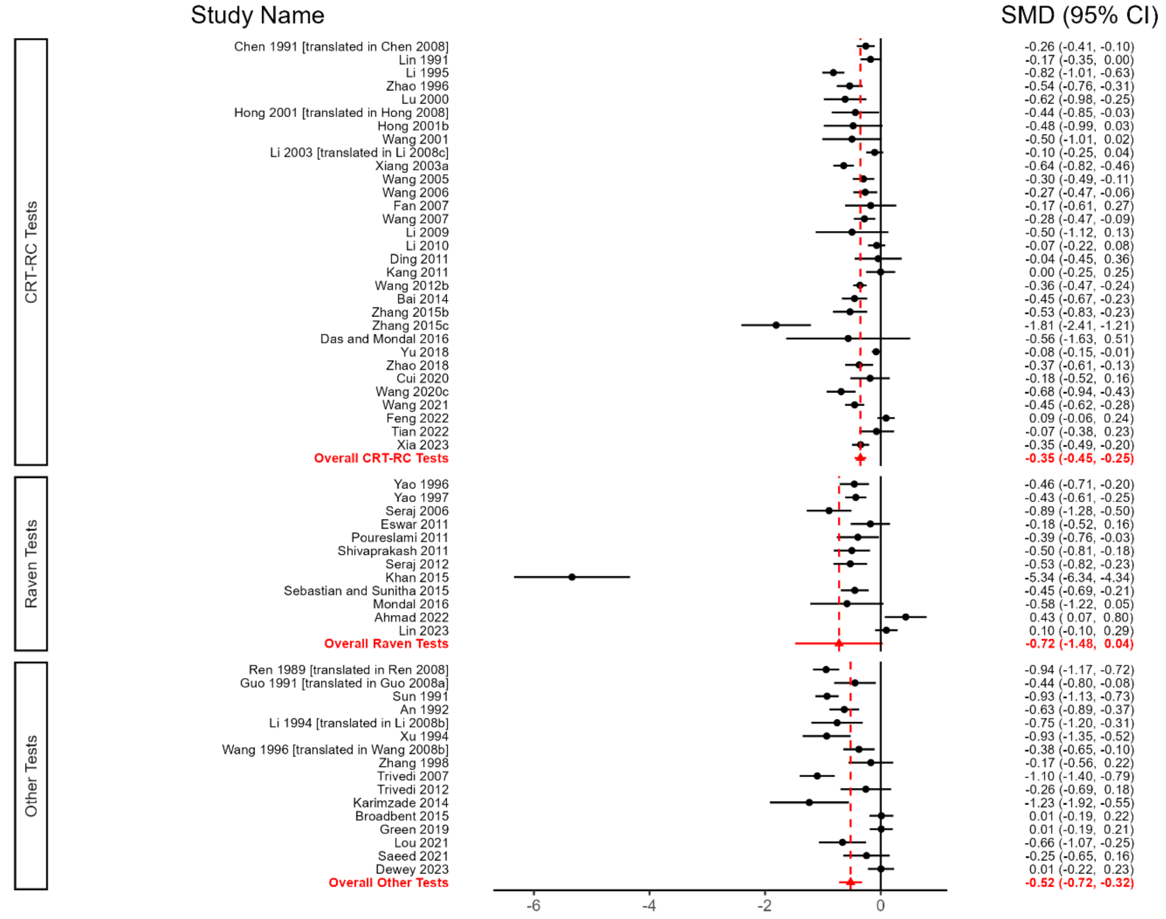

eFigure 15. Association Between Fluoride Exposure and IQ Scores in Children: Effect by Outcome Assessment Type

© 2025 Taylor KW et al. *JAMA Pediatrics*.

Exposure Matrix Subgroup Analysis

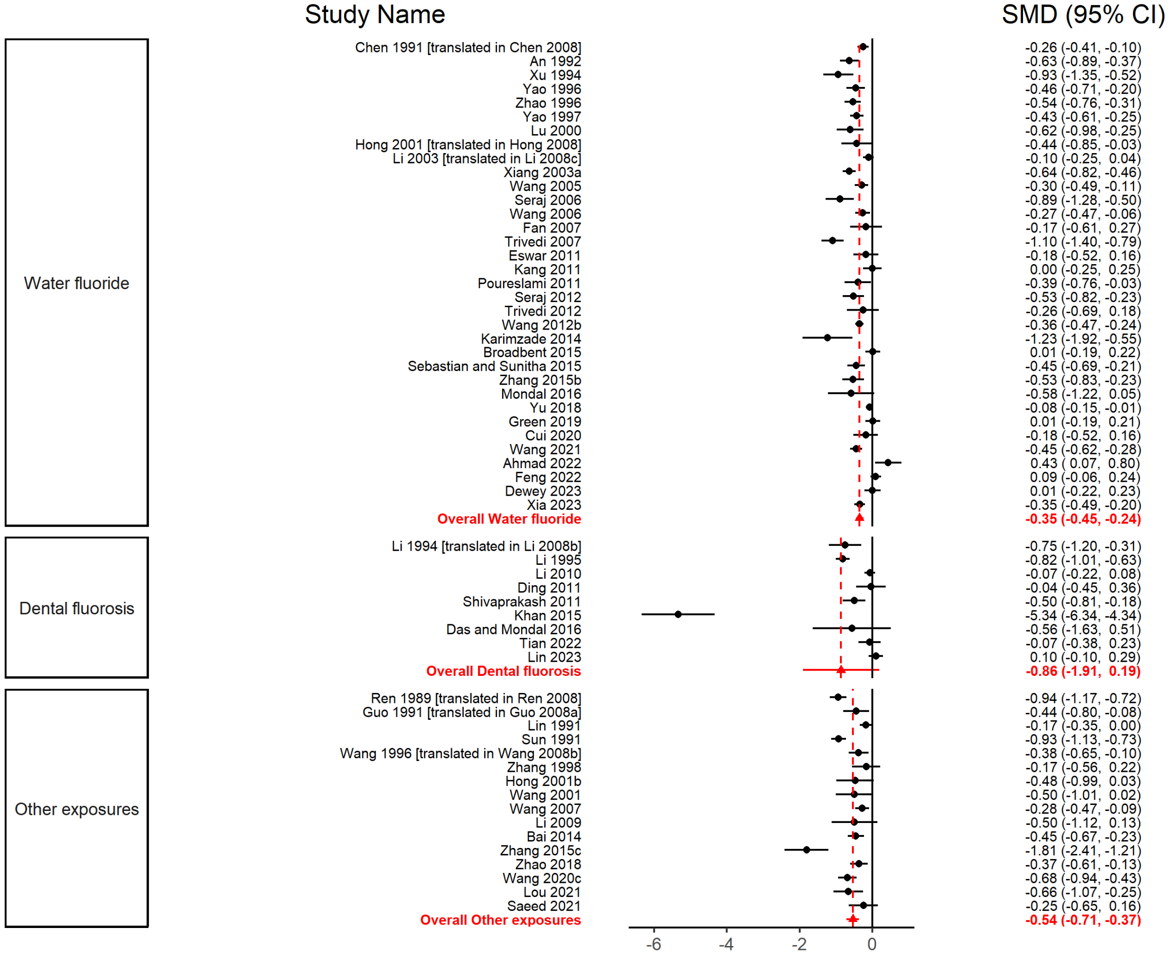

eFigure 16. Association Between Fluoride Exposure and IQ Scores in Children: Effect by Exposure Matrix

Exposure matrices include water, dental fluorosis, and other exposures (iodine, arsenic, aluminum, and fluoride from coal burning).

© 2025 Taylor KW et al. *JAMA Pediatrics*.

Dose-Response Meta-Analysis Using Mean Effect Estimates

To examine a potential nonlinear relationship between exposure to fluoride and children's IQ levels, quadratic terms and restricted cubic splines were created, and a potential departure from a linear trend was assessed by testing the coefficient of the quadratic term and a second spline equal to zero. Models were compared and the best model fit was determined based on the maximum likelihood Akaike information criterion (AIC).[13] The AIC is a goodness-of-fit measure that adjusts for the number of parameters in the model, and lower AIC values indicate better fitting models (**eTable 4**). However, when differences in AIC values between models are not large, thus suggesting that both models fit the data equally well, the parsimony principle favors the simpler model.

To examine the shape of the dose-response relationship at lower exposure levels, we also evaluated relationships including only exposures in the 0 to <4 mg/L range, the Environmental Protection Agency (EPA) current enforceable drinking water standard for fluoride; the 0 to <2 mg/L range, the EPA's non-enforceable secondary standard for fluoride in drinking water; and the 0 to <1.5 mg/L range, the WHO guideline for fluoride in drinking water. The studies included in the lower fluoride levels subgroups overlap between analyses (i.e., the <4-mg/L analysis included studies from the <2-mg/L analysis and the <2-mg/L analysis included studies from the <1.5-mg/L analysis). For example, the <2 mg/L analysis includes the studies that also have exposure levels <1.5 mg/L in addition to those with exposure levels between 1.5 and <2 mg/L.

When analyses were restricted to studies with groups exposed at <4 mg/L (i.e., 0 to <4 mg/L) fluoride in drinking water (n = 23 publications, 7 low and 16 high risk-of-bias studies), there was a statistically significant inverse association between fluoride exposure and children's IQ (SMD: −0.22; 95% CI: −0.27, −0.17; p-value < 0.001) (**eTable 4**). When restricted to <2 mg/L (i.e., 0 to <2 mg/L) fluoride in drinking water (n = 8 publications, 4 low and 4 high risk-of-bias studies), the magnitude of the effect estimate did not substantially change but the association was no longer statistically significant (SMD: −0.18; 95% CI: −0.40, 0.03; p-value = 0.096). However, when restricted to exposed groups with <1.5 mg/L (i.e., 0 to <1.5 mg/L) fluoride in drinking water (n = 7 publications, 3 low and 4 high risk-of-bias studies), there was no longer an inverse association between fluoride in drinking water and children's IQ (SMD: 0.05; 95% CI: −0.36, 0.45; p-value = 0.816). When analyses were further restricted to low risk-of-bias publications at <4 mg/L, <2 mg/L, and <1.5 mg/L fluoride in drinking water, the linear model was the best fit and the associations remained in the same direction and were larger in magnitude compared to when data from both low and high risk-of-bias studies were combined (in which case, only the SMD for <4 mg/L fluoride in drinking water was statistically significant) (**eTable 4**).

When analyses were restricted to studies with exposed groups with <4 mg/L urinary fluoride (n = 14 publications, 10 low and 4 high risk-of-bias studies), there was a statistically significant inverse association between children's urinary fluoride exposure and IQ (SMD: −0.20; 95% CI: −0.31, −0.08; p-value = 0.001) (**eTable 4**). When restricted to exposed groups with <2 mg/L urinary fluoride (n = 6 publications, 4 low and 2 high risk-of-bias studies), there was also a statistically significant inverse association (SMD: −0.08; 95% CI: −0.15, −0.005; p-value = 0.037). When restricted to exposed groups with <1.5 mg/L urinary fluoride (n = 5 publications, 4 low and 1 high risk-of-bias studies), the inverse association continued to be statistically significant (SMD: −0.08; 95% CI: −0.15, −0.003; p-value = 0.041). When analyses were further restricted to low risk-of-bias publications, the linear model was the best fit and the associations became smaller in magnitude at <4 mg/L urinary fluoride, but remained unchanged at <2 mg/L and <1.5 mg/L urinary fluoride; SMDs at all levels remained statistically significant (p-values < 0.05) (**eTable 4**).

© 2025 Taylor KW et al. *JAMA Pediatrics*.

eTable 4. Dose-Response Meta-Analysis Using Mean Effects—Restricted Maximum Likelihood Models[a]

Exposure Analysis	Parameters	Fluoride Exposure			
		All data	<4 mg/L	<2 mg/L	<1.5 mg/L
**Water Fluoride – All Studies**					
No. Studies/No. Observations[b]<br>Number of Children		31/41<br>12,487	23/29<br>9,554	8/10<br>3,682	7/7<br>2,832
Linear Model[c]	Beta (95% CI)<br>p-value<br>AIC	−0.15 (−0.20, −0.11)<br>p < 0.001<br>AIC = 59.8	−0.22 (−0.27, −0.17)<br>p < 0.001<br>AIC = 23.3	−0.18 (−0.40, 0.03)<br>p = 0.096<br>AIC = 11.8	0.05 (−0.36, 0.45)<br>p = 0.816<br>AIC = 8.2
Quadratic Model[d]	Beta (95% CI); p-value<br>Beta (95% CI); p-value<br>AIC	−0.27 (−0.33, −0.20); p < 0.001<br>0.02 (0.01, 0.03); p < 0.001<br>AIC = 55.3	−0.11 (−0.34, 0.11); p = 0.332<br>−0.04 (−0.10, 0.03); p = 0.265<br>AIC = 29.8	0.45 (−0.06, 0.97); p = 0.085<br>−0.36 (−0.64, −0.08); p = 0.012<br>AIC = 13.8	0.30 (−0.53, 1.14); p = 0.477<br>−0.23 (−1.01, 0.55); p = 0.561<br>AIC = 11.3
Restricted Cubic Splines Model[e]	Beta (95% CI); p-value<br>Beta (95% CI); p-value<br>AIC	−0.28 (−0.37, −0.19); p < 0.001<br>0.44 (0.15, 0.73); p = 0.003<br>AIC = 49.4	−0.14 (−0.33, 0.04), p = 0.128<br>−0.20 (−0.59, 0.19), p = 0.304<br>AIC = 26.4	0.50 (−0.14, 1.13); p = 0.124<br>−0.96 (−1.72, −0.19); p = 0.014<br>AIC = 11.5	0.49 (−0.50, 1.47) p = 0.334<br>−0.69 (−2.40, 1.02) p = 0.428<br>AIC = 10.2
**Water Fluoride – Low Risk-of-bias Studies**					
No. Studies/No. Observations<br>Number of Children		7/12<br>5,066	7/10<br>4,962	4/5<br>1,632	3/3<br>879
Linear model	Beta (95% CI)<br>p-value<br>AIC	−0.21 (−0.33, −0.09)<br>p = 0.001<br>AIC = 9.3	−0.23 (−0.34, −0.11)<br>p <0.001<br>AIC = 2.6	−0.33 (−0.53, −0.13)<br>p = 0.001<br>AIC = 3.9	−0.32 (−0.91, 0.26)<br>p = 0.276<br>AIC = 4.1
Quadratic Model[d]	Beta (95% CI); p-value<br>Beta (95% CI); p-value<br>AIC	−0.18 (−0.61, 0.25); p = 0.407<br>−0.03 (−0.16, 0.10); p = 0.638<br>AIC=16.3	−0.22 (−0.65, 0.22); p = 0.328<br>−0.01 (−0.13, 0.11); p = 0.878<br>AIC = 10.5	0.12 (−0.67, 0.91); p = 0.760<br>−0.26 (−0.68, 0.16); p = 0.222<br>AIC = 10.4	0.41 (−0.28, 1.11); p = 0.244<br>−0.54 (−0.99, −0.10); p = 0.016<br>AIC = 8.2
Restricted Cubic Splines Model[e]	Beta (95% CI); p-value<br>Beta (95% CI); p-value<br>AIC	−0.23 (−0.53, 0.06); p = 0.117<br>−0.16 (−1.04, 0.72); p = 0.720<br>AIC = 11.8	−0.23 (−0.62, 0.16); p = 0.247<br>−0.04 (−1.11, 1.03); p = 0.936<br>AIC = 5.9	−0.05 (−0.53, 0.44); p = 0.847<br>−0.78 (−1.95, 0.39); p = 0.191<br>AIC = 7.8	0.07 (−0.39, 0.52); p = 0.770<br>−0.40 (−0.73, −0.07); p = 0.016<br>AIC = 8.8

© 2025 Taylor KW et al. *JAMA Pediatrics*.

Exposure Analysis	Parameters	Fluoride Exposure			
		All data	<4 mg/L	<2 mg/L	<1.5 mg/L
**Urinary Fluoride – All Studies**					
No. Studies/No. Observations<br>Number of Children		20/32<br>9,756	14/25<br>8,019	6/10<br>4,692	5/8<br>4,219
Linear Model[c]	Beta (95% CI)<br>p-value<br>AIC	−0.15 (−0.23, −0.07)<br>p < 0.001<br>AIC = 69.3	−0.20 (−0.31, −0.08)<br>p = 0.001<br>AIC = 59.4	−0.08 (−0.15, −0.005)<br>p = 0.037<br>AIC = −6.64	−0.08 (−0.15, −0.003)<br>p = 0.041<br>AIC= −4.3
Quadratic Model[d]	Beta (95% CI); p-value<br>Beta (95% CI); p-value<br>AIC	−0.27 (−0.51, −0.04); p = 0.022<br>0.02 (−0.01, 0.06); p = 0.196<br>AIC = 78.6	0.07 (−0.25, 0.39); p = 0.668<br>−0.08 (−0.16, 0.003); p = 0.060<br>AIC = 66.7	−0.10 (−0.52, 0.31); p = 0.622<br>0.02 (−0.20, 0.23); p = 0.891<br>AIC = 2.1	0.07 (−1.22, 1.36); p = 0.915<br>−0.08 (−0.80, 0.64); p = 0.820<br>AIC = 1.8
Restricted Cubic Splines Model[e]	Beta (95% CI); p-value<br>Beta (95% CI); p-value<br>AIC	−0.23 (−0.40, −0.05); p = 0.013<br>0.14 (−0.19, 0.47); p = 0.410<br>AIC = 76.5	0.002 (−0.22, 0.23); p = 0.985<br>−0.30 (−0.59, −0.02); p = 0.038<br>AIC = 63.8	−0.09 (−0.31, 0.12); p = 0.403<br>0.02 (−0.21, 0.24); p = 0.874<br>AIC = 2.1	−0.03 (−0.58, 0.52); p = 0.918<br>−0.05 (−0.61, 0.51); p = 0.857<br>AIC = 2.3
**Urinary Fluoride – Sensitivity analysis including Bashash et al. (2017)[84]**					
No. Studies/No. Observations<br>Number of Children		21/33<br>9,945	15/26<br>8,208	7/11<br>4,881	5/8<br>4,219
Linear model	Beta (95% CI)<br>p-value<br>AIC	−0.14 (−0.22, −0.06)<br>p = 0.001<br>AIC = 70.1	−0.17 (−0.28, −0.06)<br>p = 0.002<br>AIC = 60.6	−0.05 (−0.14, 0.04)<br>p = 0.271<br>AIC =−6.1	−0.08 (−0.15, −0.003)<br>p = 0.041<br>AIC= −4.3
**Urinary Fluoride – Sensitivity analysis including Ibarluzea et al. (2021)[94] Bayley MDI scores**					
No. Studies/No. Observations<br>Number of Children		21/33<br>10,069	15/26<br>8,332	7/11<br>5,005	6/9<br>4,532
Linear model	Beta (95% CI)<br>p-value<br>AIC	−0.14 (−0.22, −0.06)<br>p = 0.001<br>AIC = 70.3	−0.18 (−0.29, −0.06)<br>p = 0.002<br>AIC = 60.7	−0.07 (−0.14, 0.002)<br>p = 0.055<br>AIC = −5.8	−0.04 (−0.19, 0.12)<br>p = 0.655<br>AIC = −3.7
**Urinary Fluoride – Sensitivity analysis including Ibarluzea et al. (2021)[94] McCarthy GCI scores**					
No. Studies/No. Observations<br>Number of Children		21/33<br>10,003	15/26<br>8,266	7/11<br>4,939	6/9<br>4,466
Linear model	Beta (95% CI)<br>p-value<br>AIC	−0.14 (−0.22, −0.06)<br>p < 0.001<br>AIC = 69.9	−0.18 (−0.30, −0.07)<br>p = 0.001<br>AIC = 60.2	−0.07 (−0.14, −0.0004)<br>p = 0.049<br>AIC = −6.2	−0.07 (−0.15, 0.002)<br>p = 0.056<br>AIC = −4.0

© 2025 Taylor KW et al. *JAMA Pediatrics*.

Exposure Analysis	Parameters	Fluoride Exposure			
		All data	<4 mg/L	<2 mg/L	<1.5 mg/L
**Urinary Fluoride – Low Risk-of-bias Studies**					
No. Studies/No. Observations<br>Number of Children		10/14<br>6,847	10/14<br>6,847	4/7<br>4,179	4/7<br>4,179
Linear model	Beta (95% CI)<br>p-value<br>AIC	−0.13 (−0.23, −0.03)<br>p = 0.010<br>AIC = −2.1	−0.13 (−0.23, −0.03)<br>p = 0.010<br>AIC = −2.1	−0.08 (−0.15, −0.002)<br>p = 0.044<br>AIC = −4.6	−0.08 (−0.15, −0.002)<br>p = 0.044<br>AIC = −4.6
Quadratic Model[d]	Beta (95% CI); p-value<br>Beta (95% CI); p-value<br>AIC	0.19 (−0.13, 0.51); p = 0.237<br>−0.10 (−0.18, −0.01); p = 0.021<br>AIC = 4.7	0.19 (−0.13, 0.51); p = 0.237<br>−0.10 (−0.18, −0.01); p = 0.021<br>AIC = 4.7	−0.003 (−1.30, 1.29); p = 0.997<br>−0.04 (−0.77, 0.69); p = 0.910<br>AIC = 1.5	−0.003 (−1.30, 1.29); p = 0.997<br>−0.04 (−0.77, 0.69); p = 0.910<br>AIC = 1.5
Restricted Cubic Splines Model[e]	Beta (95% CI); p-value<br>Beta (95% CI); p-value<br>AIC	0.15 (−0.13, 0.44); p = 0.295<br>−0.56 (−1.03, −0.10); p = 0.017<br>AIC = 1.7	0.16 (−0.13, 0.45); p = 0.287<br>−0.55 (−1.01, −0.10); p = 0.017<br>AIC = 1.8	−0.04 (−0.64, 0.56); p = 0.894<br>−0.05 (−0.91, 0.81); p = 0.905<br>AIC = 1.2	−0.03 (−0.63, 0.56); p = 0.910<br>−0.06 (−0.82, 0.71); p = 0.887<br>AIC = 1.4

Notes:

AIC = Akaike information criterion (lower values indicate a better fit model); SMD = standardized mean difference; p = p-value for effect estimate; MDI = Mental Development Index; GCI = General Cognitive Index

[a]Parameter estimates are changes in SMDs (beta [95% CI]) based on the restricted maximum likelihood models; model fit is represented by the maximum likelihood AIC.

[b]The observations represent the number of effect estimates from all the studies included in the analysis. Studies with more than two exposure levels provide more than one SMD for inclusion on the dose-response meta-analysis.

[c]The estimates represent change in SMD for the linear model and AIC, respectively.

[d]The estimates represent change in SMD for the linear term, change in SMD for quadratic term, AIC. Potential departure from a linear trend was assessed by testing the coefficient of the quadratic term equal to zero.

[e]The estimates represent change in SMD for the first spline term, change in SMD for the second spline term, AIC. Potential departure from a linear trend was assessed by testing the coefficient of the second spline equal to zero.

© 2025 Taylor KW et al. *JAMA Pediatrics*.

Regression Slopes Meta-Analysis

Studies with overlapping populations

Yu et al. (2018)[5] and Wang et al. (2020b)[4] used the same study cohort of children recruited in 2015 from the rural areas of Tianjin City, China. Since Wang et al. (2020b)[4] (n = 571) used a subset of the original study sample from Yu et al. (2018)[5] (n = 2,886), only results from Yu et al. (2018)[5] were included in the meta-analysis. A sensitivity analysis was performed to evaluate the impact of using the effect estimate from Wang et al. (2020b)[4] rather than the pooled effect estimate from Yu et al. (2018).[5]

Green et al. (2019)[89] and Till et al. (2020)[91] used the same Maternal-Infant Research on Environmental Chemicals (MIREC) cohort that reported drinking tap water in 10 Canadian cities, with the studies overlapping for 398 mother-child pairs. Both studies reported effect estimates for maternal urinary fluoride (MUF) and water fluoride concentrations. In the Green et al. (2019)[89] study, 512 mother-child pairs had MUF data compared to 398 pairs in Till et al. (2020).[91] Water fluoride levels were available for 420 pairs in Green et al. (2019)[89] compared to 398 pairs in Till et al. (2020).[91] Both studies reported effect estimates adjusted for maternal education, maternal race, child's sex, HOME total score, and secondhand smoke status in the child's home. In addition, Till et al. (2020)[91] adjusted for child's age at IQ testing (the age range for all children was 3–4 years old). Because of the larger sample size and because covariate adjustments were similar, results from Green et al. (2019)[89] were included in the main analysis. However, because of the more adjusted estimates from Till et al. (2020)[91] compared to Green et al. (2019),[89] a sensitivity analysis was performed using the water fluoride result for formula-fed children and the MUF result from Till et al. (2020).[91] For fluoride from intake, the estimates from both studies were used, Green et al. (2019)[89] for total fluoride intake and Till et al. (2020)[91] for infant fluoride intake from formula.

Bashash et al. (2017)[84] and Goodman et al. (2022a)[101] used the same Early Life Exposures in Mexico to Environmental Toxicants (ELEMENT) cohort. Both studies reported effect estimates for MUF concentrations. In Goodman et al. (2022a),[101] 348 mother-child pairs had data on McCarthy Scales of Children's Abilities and Wechsler Abbreviated Scale of Intelligence at a minimum of two time points (ages 4, 5, and/or 6–12 years). Bashash et al. (2017)[84] evaluated 199 mother-child pairs with data on MSCA and WASI at ages 4 and 6–12 years, but did not have data at age 5 years. Because of the larger sample size and because covariate adjustments were similar, results from Goodman et al. (2022a)[101] were included in the main analysis. However, Bashash et al. (2017)[84] provided unadjusted estimates and estimates adjusted for cohort as a random-effect model which were used in sensitivity analyses (**eTable 5**).

Three studies were excluded with reported slopes because the exposure was measured at the community level.[41,46,51] Only two studies[96;102] included in this meta-analysis were considered high risk of bias. For Goodman et al. (2022a),[101] Bashash et al. (2017),[84] Yu et al. (2018),[5] and Till et al. (2020),[91] units of exposure were transformed from 0.5 mg/L to 1 mg/L. Several studies (Cui et al. (2018),[87] Zhao et al. (2021),[98] Valdez Jimenez et al. (2017),[86] Lin et al. (2023),[105] and Grandjean et al. (2023)[104]) reported associations between IQ and log-transformed exposure, and units of exposure were transformed to 1 mg/L.[147] Yu et al. (2018)[5] reported estimates from piecewise linear regression models and provided three ranges for urinary fluoride exposure (low, 0.01–1.60 mg/L; medium, 1.60–2.50 mg/L; and high, 2.50–5.54 mg/L) and two ranges for water fluoride exposure (low, 0.20–3.40 mg/L and high, 3.40–3.90 mg/L). Since these piecewise effect estimates are likely correlated, the study-specific pooled effect estimates were used for urine and water fluoride exposures for the overall effect meta-analysis. Similarly, Xia et al. (2023)[106] reported estimates separately for children in the control group and children in the high fluoride group, and a study-specific polled effect estimate was used in the overall effect meta-analysis. A sensitivity analysis was performed to evaluate the impact of using pooled estimates rather than piecewise estimates from Yu et al. (2018)[5] and Xia et al. (2023)[106] (**eTable 5**).

© 2025 Taylor KW et al. *JAMA Pediatrics*.

For studies reporting multiple measures of fluoride exposure, the results associated with measured or estimated individual-level exposures, biomarker levels (such as urinary fluoride), or fluoride intake levels were prioritized over water fluoride concentrations (see protocol; https://ntp.niehs.nih.gov/go/785076); however, subgroup analyses by exposure metric (urinary fluoride, fluoride intake, and water fluoride) were also performed.

Regression slopes meta-analysis sensitivity analyses

Information about demographic variables was not always accessible, making it difficult to study the impact of potential confounders on effect estimates. Sensitivity analyses for the *regression slopes meta-analysis* explored the impact of using unadjusted estimates, and results were not significantly impacted (**eTable 5**). Also, most of the estimates used in the *mean-effects meta-analyses* come from studies that used fluoride concentrations at the community level to represent exposure. Therefore, unless community-level clustering is accounted for in the analysis, the standard errors of the difference in means between exposed and reference groups are likely to be biased. This is less of an issue in studies using individual-level exposures (e.g., the *regression slopes meta-analysis*). However, most studies lacked adjustment for clustering,[5, 80, 87] or for complex sampling strategies.[5, 80] Therefore, we performed sensitivity analyses to assess the impact of such issues, and there were minimal changes in the pooled slope (**eTable 5**). In the *regression slopes meta-analysis*, we used the estimates from the Green et al. (2019)[89] and Goodman et al. (2022a)[101] studies, which reported from the models using the clustering variable (city and cohort, respectively) as a fixed effect. However, the sensitivity analysis using the regression slopes from the corresponding models with random effects from the Green et al. (2019)[89] and Bashash et al. (2017)[84] studies showed that a 1-mg/L increase in urinary fluoride was associated with a statistically significant lower IQ score of 1.65 points (95% CI: −2.36, −0.94). This suggests that clustering is not a significant issue in the results of our *regression slopes meta-analysis*.

Limitations of Publication Bias Tests

There are also several limitations to the existing approaches for evaluating potential for publication bias. The funnel plot asymmetry is a subjective assessment and is recommended only when at least 10 studies are included in the meta-analysis.[148] Furthermore, the Egger regression test and Begg's rank tests[19-21] may suffer from inflated type I power and limited power in certain situations.[149] Additionally, trim-and-fill analyses typically rely on Egger's test results to determine whether data should be imputed on the left or right side of the funnel plot and do not account for reasons of funnel plot asymmetry other than publication bias. However, we used multiple estimator methods and specified directions for bias correction regardless of the Egger's test results in order to examine effects of different estimators and outlying studies.[150] The trim-and-fill analyses using various estimators evaluating the potential impact of publication bias consistently supported the inverse association between measures of fluoride exposure and children's IQ.

© 2025 Taylor KW et al. *JAMA Pediatrics*.

eTable 5. Regression Slopes and 95% CIs From Random Effects Meta-Analysis of the Association Between Individual-Level Measures of Urinary Fluoride and IQ Scores in Children: Sensitivity Analyses

Analysis	Number of Studies	Beta (95% CI)	Heterogeneity	
			p-value	I^2
**Overall Estimate**				
Full-scale IQ	13	−1.63 (−2.33, −0.93)	<0.001	60%
**Sensitivity Analyses**				
Using the piecewise estimates from Yu et al. (2018)[5] and Xia et al. (2023)[106]				
Full-scale IQ	16[a]	−1.64 (−2.52, −0.76)	<0.001	83%
Using effect estimates from Wang et al. (2020b)[4] rather than Yu et al. (2018)[5]				
Full-scale IQ	13	−1.57 (−2.22, −0.93)	<0.001	63%
Using Till et al. *(2020)[91] rather than Green et al. (2019)[89] estimates*				
Full-scale IQ	13	−1.66 (−2.34, −0.97)	<0.001	60%
Using estimates from random effect models for Green et al. (2019)[89] and Bashash et al. (2017)[84]				
Full-scale IQ	13	−1.65 (−2.36, −0.94)	<0.001	61%
Males	3	−1.30 (−4.11, 1.50)	0.069	70%
Females	3	−1.09 (−2.22, 0.04)	0.391	0%
Excluding Cui et al. (2018)[87]				
Full-scale IQ	12	−1.66 (−2.43, −0.90)	<0.001	63%
Excluding Yu et al. (2018)[5] Zhang et al. (2015b)[80], Feng et al. (2022)[100] and Tian et al. (2022)[102]				
Full-scale IQ	9	−1.64 (−2.50, −0.78)	<0.001	68%
Using unadjusted estimates from Bashash et al. (2017),[84] Cui et al. (2018),[87] Green et al. (2019)[89], Yu et al. (2018)[5]				
Full-scale IQ	13	−1.65 (−2.36, −0.95)	<0.001	60%
Using Verbal or Performance IQ scores from Green et al. (2019)[89] and *Goodman et al. (2022a)[101]*				
Verbal IQ	13	−1.58 (−2.25, −0.90)	0.001	58%
Performance IQ	13	−1.73 (−2.50, −0.96)	<0.001	68%
Using Goodman et al. (2022a)[101] McCarthy GCI scores, Valdez Jimenez et al. (2017)[86] (Bayley MDI scores), Cantoral et al. (2021)[92] (Bayley III cognitive scores), Ibarluzea et al. (2021)[94] (Bayley MDI scores).				
Urinary fluoride	15	−1.63 (−2.32, −0.94)	<0.001	58%
Intake	3	−3.28 (−5.87, −0.68)	0.799	0%
Water fluoride	2	−4.77 (−9.09, −0.45)	0.707	0%
Using Goodman et al. (2022a)[101] McCarthy GCI scores, Valdez Jimenez et al. (2017)[86] (Bayley MDI scores), Cantoral et al. (2021)[92] (Bayley III cognitive scores), Ibarluzea et al. (2021)[94] (McCarthy GCI scores).				
Urinary fluoride	15	−1.70 (−2.38, −1.02)	<0.001	57%
Intake	3	−3.28 (−5.87, −0.68)	0.799	0%
Water fluoride	2	−4.77 (−9.09, −0.45)	0.707	0%

Notes:

CI = confidence interval; GCI = General Cognitive Index; MDI = Mental Development Index.

[a]Number of effect estimates from 13 studies included in analysis.

© 2025 Taylor KW et al. *JAMA Pediatrics*.

Tests Assessing Potential Publication Bias in Studies With Individual-Level Exposures

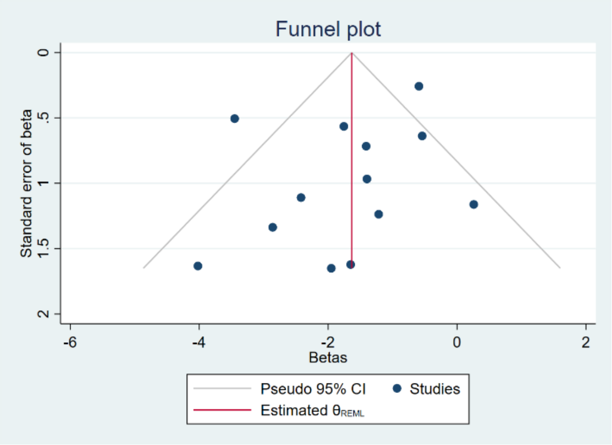

eFigure 17. Funnel Plot for Studies With Individual-Level Exposures

```
Regression-based Egger test for small-study effects
Random-effects model
Method: REML

H0: beta1 = 0; no small-study effects
            beta1 =     -0.63
      SE of beta1 =     0.850
                z =     -0.74
       Prob > |z| =    0.4581
```

eFigure 18. Test for Publication Bias for Studies With Individual-Level Exposures

© 2025 Taylor KW et al. *JAMA Pediatrics*.

Forest Plots for the Random Effects Meta-Analysis of Regression Slopes: Subgroup Analyses

Risk-of-bias Subgroup Analysis

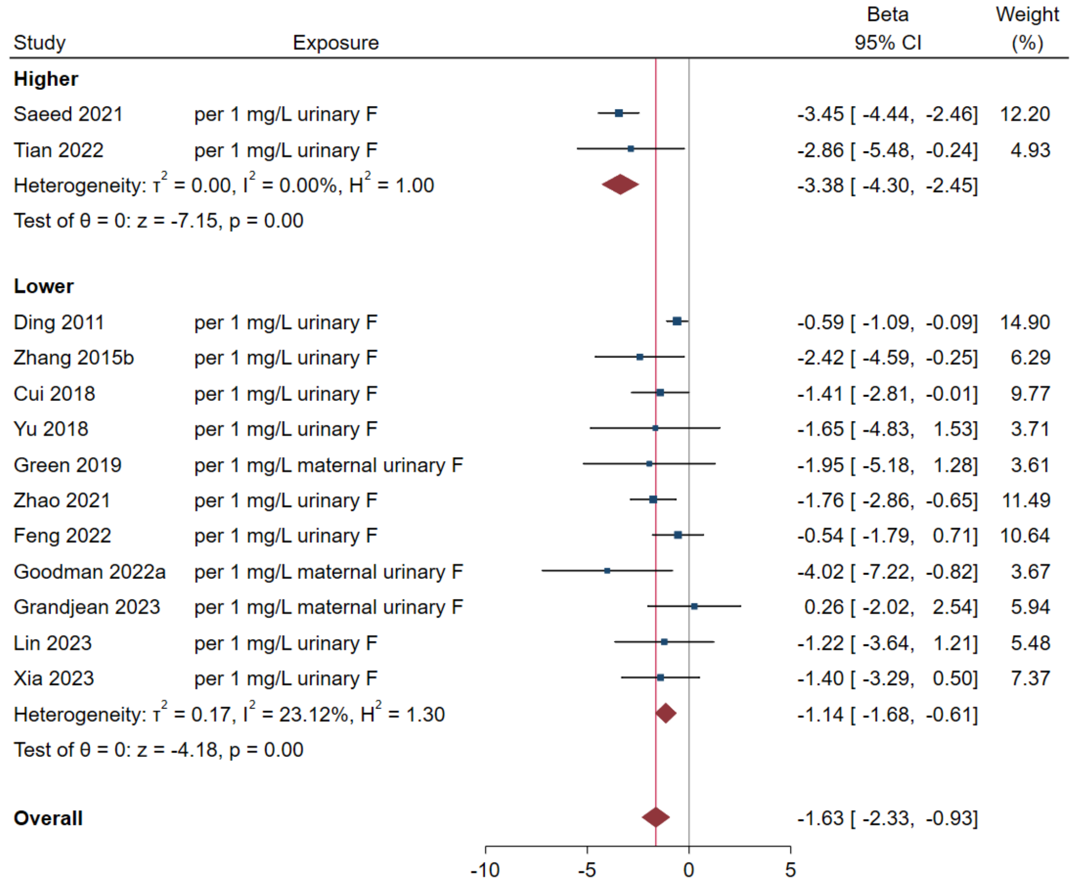

eFigure 19. Association Between Individual-Level Fluoride Exposure and IQ Scores in Children: Effect by Risk of Bias

```
Regression-based Egger test for small-study effects
Random-effects model
Method: REML

H0: beta1 = 0; no small-study effects
            beta1 =     -1.10
      SE of beta1 =     0.521
                z =     -2.11
       Prob > |z| =    0.0349
```

eFigure 20. Test for Publication Bias for Low Risk-of-Bias Studies With Individual-Level Exposures

© 2025 Taylor KW et al. *JAMA Pediatrics*.

```
Nonparametric trim-and-fill analysis of publication bias
Linear estimator, imputing on the right

Iteration                              Number of studies =     15
  Model: Random-effects                         observed =     11
 Method: REML                                   imputed =      4

Pooling
  Model: Random-effects
 Method: REML

----------------------------------------------------------------
            Studies |      Beta    [95% conf. interval]
--------------------+-------------------------------------------
           Observed |    -1.142      -1.677      -0.607
 Observed + Imputed |    -0.775      -1.334      -0.216
----------------------------------------------------------------
```

eFigure 21. Trim-and-Fill Analysis for Low Risk-of-Bias Studies

Filling in to the right using a linear estimator showed no significant change in the pooled SMD.

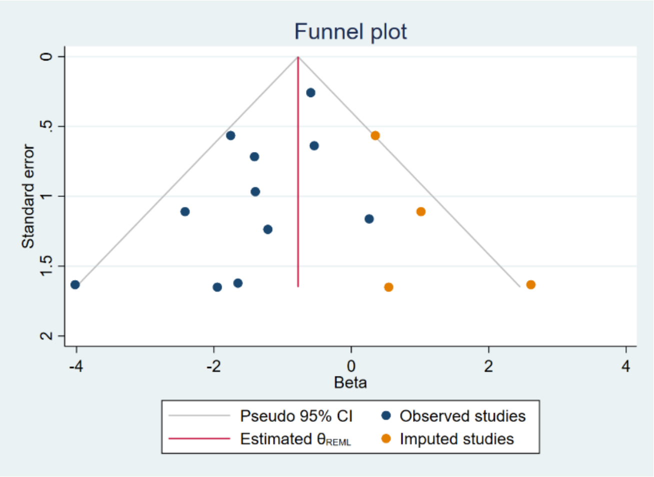

eFigure 22. Filled-In Funnel Plots for Low Risk-of-Bias Studies

Random-effects pooled estimate after filling in to the right using a linear estimator (the linear estimator to the left or the run estimator to the left or to the right showed no change in the pooled slope).

© 2025 Taylor KW et al. *JAMA Pediatrics*.

Exposure Matrix Subgroup Analysis

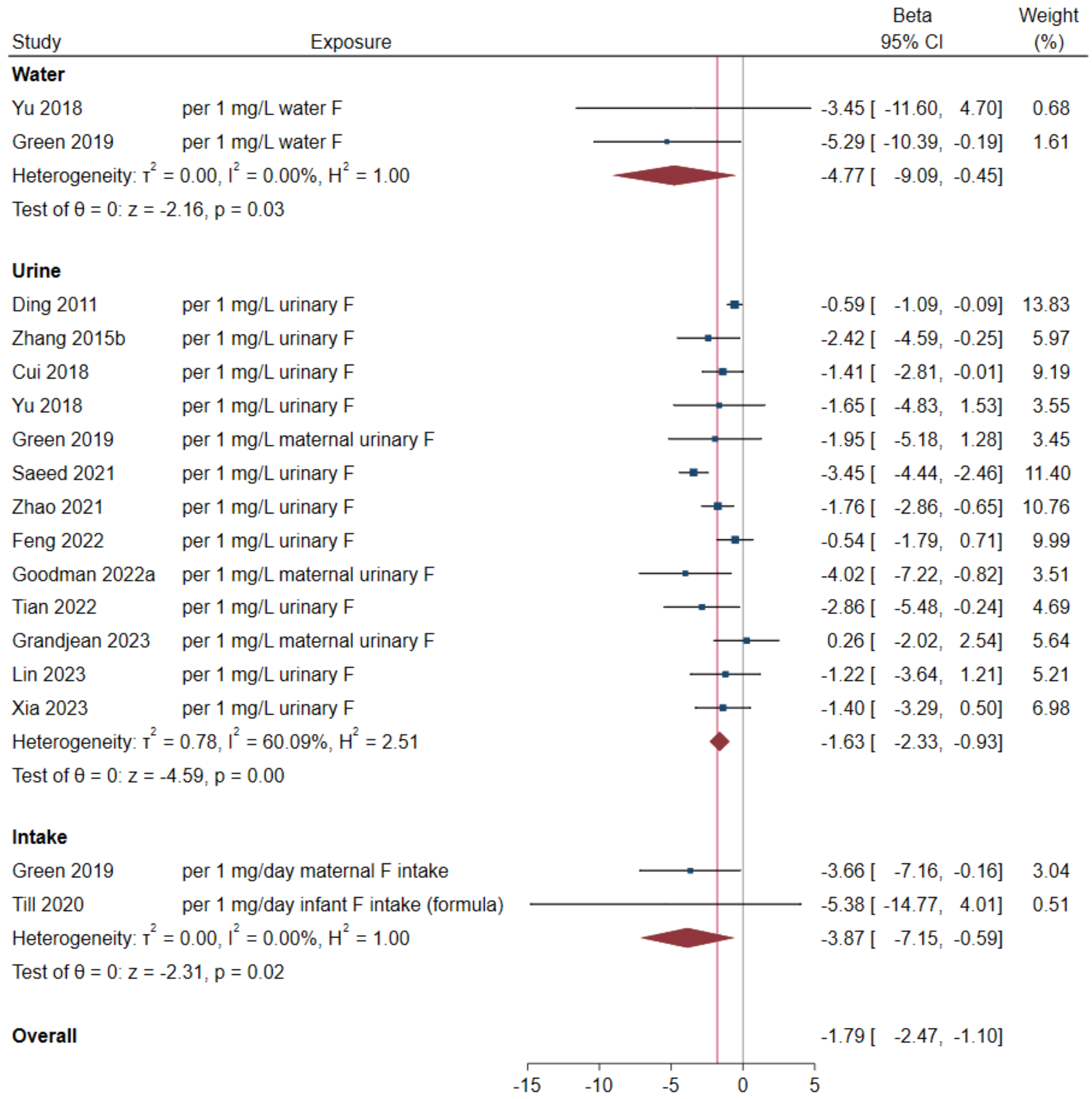

eFigure 23. Association Between Individual-Level Fluoride Exposure and IQ Scores in Children: Effect by Exposure Matrix

© 2025 Taylor KW et al. *JAMA Pediatrics*.

Country Subgroup Analysis

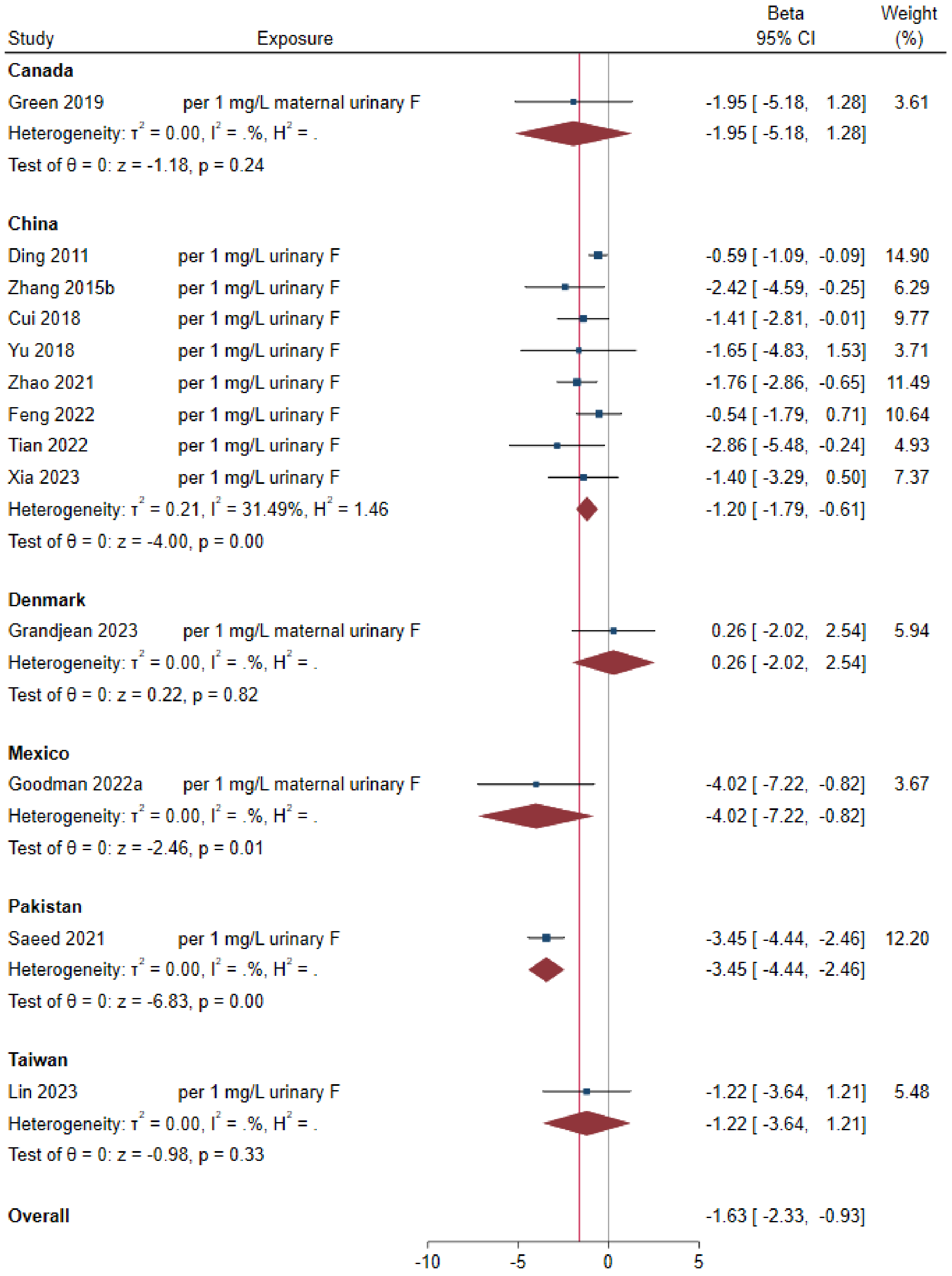

eFigure 24. Association Between Individual-Level Fluoride Exposure and IQ Scores in Children: Effect by Country

Note: The analyses for publication bias for studies by country rely on a very small number of studies each and are not shown.

© 2025 Taylor KW et al. *JAMA Pediatrics*.

Outcome Assessment Type Subgroup Analysis

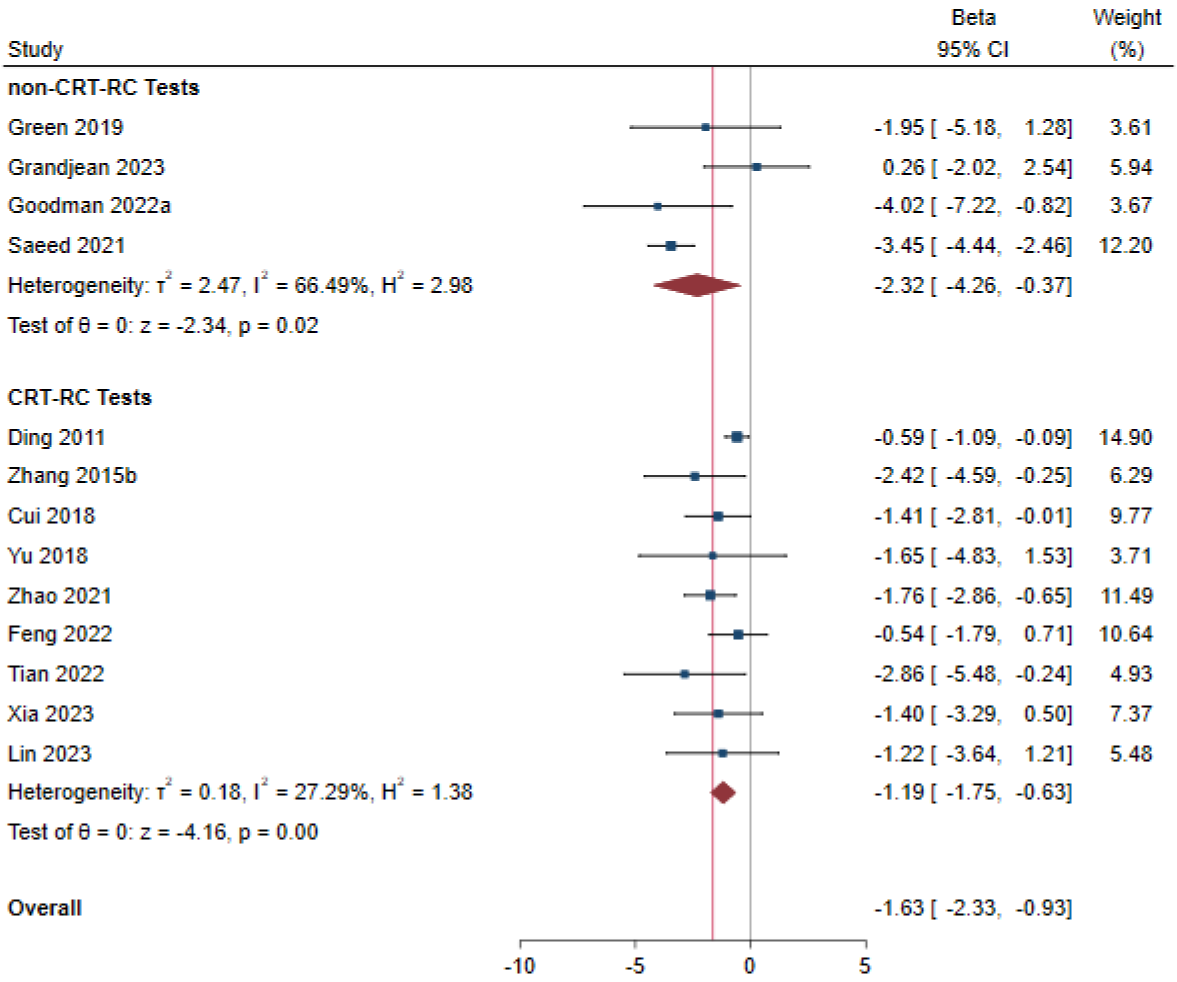

eFigure 25. Association Between Individual-Level Fluoride Exposure and IQ Scores in Children: Effect by Assessment Type

Note: The analyses for publication bias for CRT-RC studies and non-CRT-RC studies include only nine and four studies, respectively, and are not shown.

© 2025 Taylor KW et al. *JAMA Pediatrics*.

Sex Subgroup Analysis

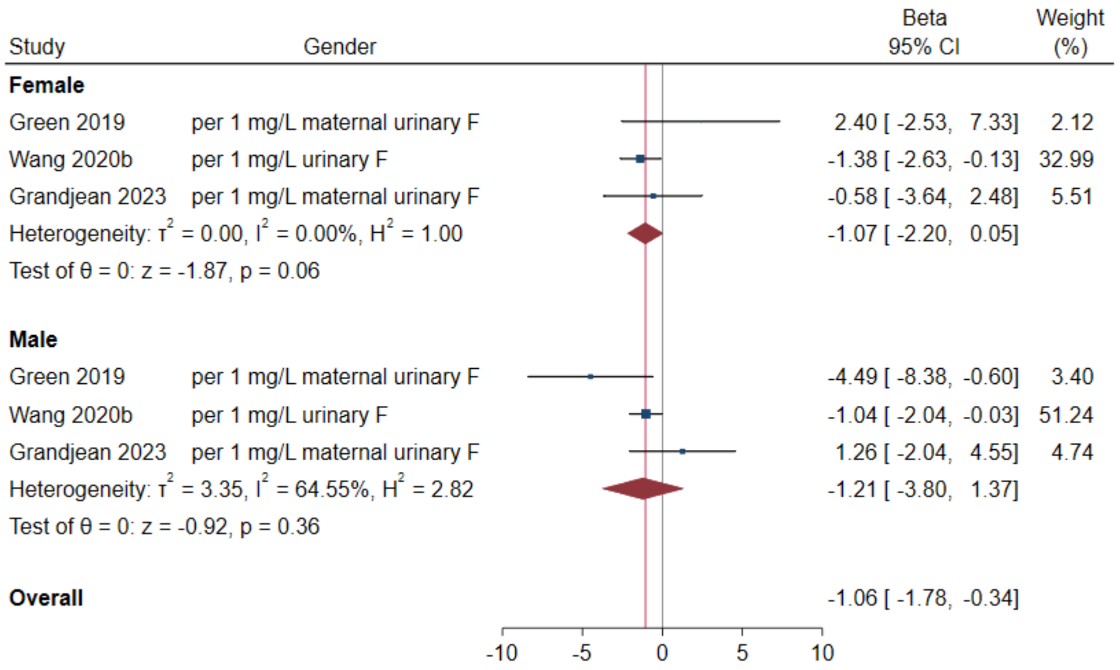

eFigure 26. Association Between Individual-Level Fluoride Exposure and IQ Scores in Children: Effect by Sex

Note: The analyses for publication bias by gender relies on three studies each and are not shown.

© 2025 Taylor KW et al. *JAMA Pediatrics*.

Prenatal vs Postnatal Exposure Subgroup Analysis

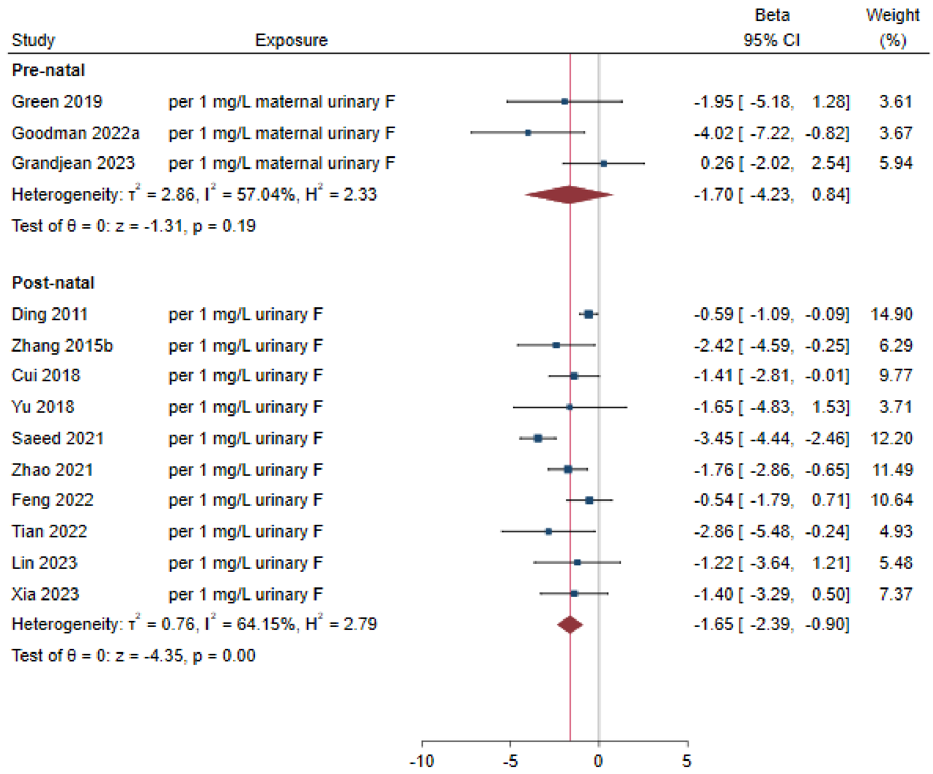

eFigure 27. Association Between Individual-Level Fluoride Exposure and IQ Scores in Children: Effect by Prenatal vs. Postnatal Exposure

© 2025 Taylor KW et al. *JAMA Pediatrics*.

Characteristics of Previous Meta-Analyses

Six previous meta-analyses found an inverse association between fluoride exposure and children's IQ (**eTable 6).** Three previous meta-analyses used the SMD as the effect measure,[151-153] two used a weighted mean difference,[154, 155] and one used an odds ratio.[156] Although the use of various effect measures makes comparison of the magnitude of the meta-analytic associations difficult across meta-analyses, all meta-analyses were consistent in their finding of an inverse association between fluoride exposure and children's IQ.

eTable 6. Previous Meta-Analyses on Exposures to Fluoride and Children's IQ

Analysis	Number of Studies	Pooled Effect Type, Estimate (95% CI)	Heterogeneity	
			p-value	I^2
**Current *mean-effects meta-analysis***				
**Current Meta-analysis**	59	SMD, −0.45 (−0.57, −0.33)	<0.001	94%
**Previous Meta-Analyses**				
Tang et al. (2008)[154]	16	WMD, −5.03 (−6.51, −3.55)	NR	NR
Choi et al. (2012)[151]	27	SMD, −0.45 (−0.56, −0.34)	<0.001	80%
Duan et al. (2018)[152]	26	SMD, −0.52 (−0.62, −0.42)	<0.001	69.1%
Miranda et al. (2021)[156]	10	OR, 3.88 (2.41, 6.23)	<0.0001	77%
Kumar et al. (2023)[153]	28	SMD, −0.33 (−0.44, −0.22)	<0.00001	83%
Veneri et al. (2023)[155]	30 (38 results)	WMD, −4.68 (−6.45, −2.92)	NR	98.75%

Notes:
CI = confidence interval; NR = not reported; SMD = standardized weighted mean difference; OR = odds ratio of low IQ in the high fluoride vs. low fluoride groups; WMD = weighted mean difference

Some of these meta-analyses lacked information on study quality evaluation, and most used group-level estimates of fluoride exposure. The meta-analyses used a range of methods and varied in the degree of transparency, objectivity (e.g., predefined protocol), and rigorousness of the analytic approaches applied, including procedures such as: study selection, quantitative evaluation of overall associations, and selection of effect measures. For the effect measure, three meta-analyses used a standardized mean difference (SMD),[151-153] two used weighted mean difference (WMD),[154, 155] and one used an odds ratio.[156]

Tang et al. (2008)[154] conducted a meta-analysis of 16 case-control studies with a pooled WMD calculated using the Mantel-Haenszel method. The study reported significant inverse associations that indicate "children who live in a fluorosis area have five times higher odds of developing low IQ than those who live in a non-fluorosis area or a slight fluorosis area." The authors' interpretation of the WMD as "odds of developing low IQ" is lacking because the WMD compares mean differences in IQ scores, not probabilities. Other limitations of this analysis included incorrect statements on study design assignment (all included studies are cross-sectional, not case-control as labeled in the analysis); lack of investigation of sources of heterogeneity (I^2 not reported or incorrectly listed as "not applicable"); unclear choice of sensitivity analyses; and no reporting of a predefined protocol or software used for the analyses.

Choi et al. (2012)[151] assessed 27 studies comparing mean IQ scores in high fluoride exposure groups to those in low fluoride exposure groups. Most of these study populations were from China and were exposed to fluoride through drinking water. Choi et al. (2012)[151] found an inverse association between fluoride exposure and IQ in children (**eTable 6**). In meta-regression analyses, the study found that year of publication (but not mean age of the study children) was a significant source of heterogeneity. When the analyses were restricted to the 16 studies that used the Combined Raven's Test–The Rural edition in China (CRT-RC), the mean age of the study children (but not year of publication) was a significant

© 2025 Taylor KW et al. *JAMA Pediatrics*.

predictor of the estimated SMD. While the study estimated a risk ratio for living in an endemic fluorosis area, authors excluded studies with individual-level measures of exposure and were not able to perform a formal dose-response analysis. Although software used for the meta-analysis was reported, the study lacked a predefined protocol.

A more recent meta-analysis[152] assessed 26 studies that evaluated intelligence levels in children exposed to high or low drinking water fluoride. Thirteen studies included in the Choi et al. (2012)[151] meta-analysis were not considered in the Duan et al. (2018)[152] evaluation for unclear reasons. The Duan et al. (2018)[152] study included four studies from Iran and four studies from India, with the remaining studies conducted in China. Duan et al. (2018)[152] found an inverse association between fluoride exposure and IQ in children (**eTable 6**). In meta-regression analyses, the mean age of study children significantly affected the relationship between high water fluoride levels and children's intelligence levels. Subgroup analyses included country, age (<10 or ≥10 years), water fluoride level, type of intelligence assessment, and sex. Duan et al. (2018)[152] also performed a dose-response meta-analysis that suggested a significant association between increased water fluoride exposure and lower intelligence levels; however, it is unclear which studies were included in this analysis. The study reported both linear and nonlinear inverse relationships between fluoride exposure and children's intelligence levels. Software used in the meta-analysis was reported; however, the study lacked a predefined protocol.

Miranda et al. (2021)[156] performed a meta-analysis of 10 studies and found an association between high fluoride exposure and decreased IQ as reflected by odds ratios (**eTable 6**). The meta-analysis has many serious methodological and reporting limitations. Regardless of how the original studies reported the data, Miranda et al. (2021)[156] chose to "classify the studies according to the WHO guidelines that consider optimal levels between 0.5–1.0 mg/L (low levels) and > 2 mg/L, as higher levels for water fluoridation" thus limiting the body of evidence to the few studies reporting the "optimal" low levels, and also excluding studies that reported exposure levels qualitatively (e.g., just described as high and low). In addition, only "low risk of bias" studies were included, and study selection raises serious concerns since many relevant studies were excluded. Other limitations included inappropriate risk-of-bias assessment methodology (i.e., best practices were not followed, and no rationale was presented to support ratings that were reported); improper conduct of the quantitative analysis; and use of an effect measure that is limited in its interpretation and usefulness for assessing fluoride-IQ associations.

Veneri et al. (2023)[155] performed a meta-analysis of 30 studies. They reported significant inverse associations between fluoride exposure and IQ in children (**eTable 6**) although, the meta-analysis has significant limitations. Veneri et al. (2023)[155],with WMDs used as effect measures and only results for spline models reported, observed inverse non-linear associations between children's IQ and drinking water and children's urinary fluoride. When the outcome assessment approaches are not identical across studies—such as when IQ is measured with various tests with different scales—a pooled WMD is not an appropriate effect measure (as compared to the pooled SMD which accounts for test heterogeneity). Also, several studies are counted multiple times for each exposure measure of fluoride provided in the study (that result in the same WMD) with no evidence that the correlation between study-specific effects was considered. This occurs for 6 of the 30 included studies.[41,57,80,99,140,142] Another limitation is lack of adequate justification for not considering or including several studies that would appear to meet their inclusion criteria.[101,112,138] In addition, although the study research question called for a separate assessment of prenatal exposure, and several studies had maternal urinary fluoride measures, there is no analysis of prenatal fluoride exposure. There is also no discussion of heterogeneity or evidence to indicate that sources of heterogeneity were investigated using stratified analyses or meta-regression. Lastly, for assessing risk of bias, Veneri et al. (2023)[155] employ the ROBINS-E tool. This tool uses an algorithm to determine an individual study's overall risk of bias, which is based on the domain with the greatest risk of bias. For example, if one domain receives a judgement of "high" risk of bias, the study is determined to be "high" risk of bias overall. This approach assumes, without supporting evidence, that each type of bias

© 2025 Taylor KW et al. *JAMA Pediatrics*.

has an equal influence which may distort true study quality measures. Approaches that give different types of biases equal weight or influence are discouraged by the National Academies of Science (http://bit.ly/2CbAd1A) and others[157-159] as they hinder a more thoughtful and informative critical examination of the evidence and do not acknowledge that some potential biases are more important and influential than others.[160-162] In contrast, the OHAT approach is consistent with Cochrane guidance[163] in which studies may be considered “lower” risk of bias (as compared to “higher” risk of bias) based on concern for bias on key domains (e.g., exposure assessment, outcome assessment, confounding) which are determined on a project-specific basis. Veneri et al. (2023)[155] cautioned that the observational design of the reviewed studies may have resulted in unmeasured or residual confounding, reducing confidence in the associations. Although we agree that unmeasured or residual confounding may be a concern, when assessing the impact of each covariate in our draft assessment,[1] we carefully considered this issue and found that no trends were discernable that would suggest bias due to confounding could explain the consistency of the inverse association across the body of evidence.

Using a subset of studies included in our meta-analysis, Kumar et al. (2023)[153] reported a statistically significant inverse association between fluoride exposure and children’s IQ in their overall mean-effects meta-analysis of 28 studies (31 results). The study reported significant inverse associations between fluoride exposure and children’s intelligence measures (IQ and cognition scores) (**eTable 6**). The analysis included double counting of three studies that contributed data to both endemic and nonendemic fluoride areas (i.e., per Kumar et al. (2023)[153] “> and < ~1.5 mg/L fluoride levels” respectively). Kumar et al. (2023)[153] discussed a number of reasons why the results from studies in “endemic” regions may not apply to areas with generally lower exposures to fluoride and speculate, among other reasons, the existence of a population threshold for IQ effects. However, they do not provide evidence to support such a threshold. When restricting analyses to eight studies from the nonendemic fluorosis area, the meta-analysis found a positive non-significant association. Authors also report no significant association in nonlinear modeling with restricted cubic splines. Meta-analyses of regression coefficients from studies with children’s and maternal urinary fluoride found a positive nonsignificant association. The publication lacks essential details supporting the authors’ conclusion. Missing information includes, but is not limited to, a predefined protocol with study eligibility criteria, documentation supporting risk-of-bias ratings, and important details about the analysis (e.g., assessment of sources of heterogeneity, interpretation and assessment of publication bias, and rationale for specific sensitivity analyses).

Additional Publications on Fluoride Exposure and Neurodevelopmental Outcomes

Two recent publications concluded that current estimated fluoride exposures, including those associated with community water fluoridation, are not associated with children’s IQ or other neurodevelopmental outcomes.[153,164,165] In a narrative-style review, Guth et al. (2020)[164] reported that 21 of 23 epidemiological studies reported an association between “high” fluoride exposure and lower measures of children’s intelligence, but then concluded that, even for individuals with relatively high fluoride intake, levels of exposure in Europe are “clearly below levels that lead to adverse effects in vitro or in animals.” Do et al. (2023)[165], in a population-based longitudinal study of Australian schoolchildren, concluded that exposure to fluoridated water during the first 5 years of life was not associated with measures of emotional and behavioral development and executive function. The neurodevelopmental tests used in Do et al. (2023)[165] (parental assessments of children using the Strengths and Difficulties Questionnaire and the Behavior Rating Inventory of Executive Function) were coupled with estimates of fluoride exposure based on residential history and drinking water fluoride levels associated with postal codes. Although the authors concluded that executive function in adolescents was “at least equivalent” to that of children who had no exposure to fluoridated water, children’s IQ was not measured in this study.

© 2025 Taylor KW et al. *JAMA Pediatrics*.

References

1. National Toxicology Program (NTP). 2024. NTP monograph on the state of the science concerning fluoride exposure and neurodevelopment and cognition: a systematic review. Research Triangle Park, NC: National Toxicology Program. NTP Monograph 08. https://doi.org/10.22427/NTP-MGRAPH-8

2. National Toxicology Program (NTP). *Protocol for systematic review of effects of fluoride exposure on neurodevelopment*. U.S. Department of Health and Human Services, Public Health Service, National Institutes of Health; 2020. https://ntp.niehs.nih.gov/ntp/ohat/fluoride/ntpprotocol_revised20200916_508.pdf

3. Higgins JPT, Thomas J, Chandler J, et al., eds. *Cochrane Handbook for Systematic Reviews of Interventions version 6.4 (updated August 2023)*. Cochrane; 2023.

4. Wang M, Liu L, Li H, et al. Thyroid function, intelligence, and low-moderate fluoride exposure among Chinese school-age children. *Environ Int*. 2020b;134:105229. doi:https://doi.org/10.1016/j.envint.2019.105229

5. Yu X, Chen J, Li Y, et al. Threshold effects of moderately excessive fluoride exposure on children's health: A potential association between dental fluorosis and loss of excellent intelligence. *Environ Int*. 2018;118:116-124. doi:https://doi.org/10.1016/j.envint.2018.05.042

6. National Toxicology Program (NTP). *OHAT risk of bias rating tool for human and animal studies*. U.S. Department of Health and Human Services, Public Health Service, National Institutes of Health; 2015.

7. Bonett DG. Confidence intervals for standardized linear contrasts of means. *Psychol Methods*. 2008;13(2):99-109. doi:https://doi.org/10.1037/1082-989x.13.2.99

8. Hedges LV, Olkin I. *Statistical methods for meta-analysis*. Academic Press; 1985.

9. Rosenthal R. Parametric measures of effect size. In: Cooper H, Hedges LV, eds. *The Handbook of Research Synthesis*. Russell Sage Foundation; 1994.

10. Crippa A, Thomas I, Orsini N. A pointwise approach to dose-response meta-analysis of aggregated data. *Int J Stat Med Res*. 2018;7(2):25-32.

11. Crippa A, Discacciati A, Bottai M, Spiegelman D, Orsini N. One-stage dose–response meta-analysis for aggregated data. *Stat Methods Med Res*. 2019;28(5):1579-1596. doi:https://doi.org/10.1177/0962280218773122

12. Orsini N. Weighted mixed-effects dose–response models for tables of correlated contrasts. *Stata J*. 2021;21(2):320-347. doi:https://doi.org/10.1177/1536867x211025798

13. Müller S, Scealy JL, Welsh AH. Model selection in linear mixed models. *Statist Sci*. 2013;28(2):135-167. doi:https://doi.org/10.1214/12-STS410

14. U.S. Environmental Protection Agency (US EPA). *New fluoride risk assessment and relative source contribution documents*. Office of Water; 2011. EPA-822-F-11-001. https://www.epa.gov/sites/default/files/2019-03/documents/fluoride-risk-assess-factsheet.pdf

15. World Health Organization (WHO). *Guidelines for drinking-water quality. Fourth edition*. 2011. https://apps.who.int/iris/bitstream/handle/10665/44584/9789241548151_eng.pdf?sequence=1&isAllowed=y&ua=1

16. DerSimonian R, Laird N. Meta-analysis in clinical trials. *Control Clin Trials*. 1986;7(3):177-88. doi:https://doi.org/10.1016/0197-2456(86)90046-2

© 2025 Taylor KW et al. *JAMA Pediatrics*.

17. Cochran WG. The combination of estimates from different experiments. *Biometrics*. 1954;10(1):101-129. doi:https://doi.org/10.2307/3001666

18. Higgins JPT, Thompson SG, Deeks JJ, Altman DG. Measuring inconsistency in meta-analyses. *BMJ*. 2003;327(7414):557-560. doi:https://doi.org/10.1136/bmj.327.7414.557

19. Begg CB, Mazumdar M. Operating characteristics of a rank correlation test for publication bias. *Biometrics*. 1994;50(4):1088-1101. doi:https://doi.org/10.2307/2533446

20. Egger M, Smith G, Schneider M, Minder C, eds. *Systematic reviews in health care: Meta-analysis in context*. BMJ Publishing Group; 2008.

21. Egger M, Smith GD, Schneider M, Minder C. Bias in meta-analysis detected by a simple, graphical test. *BMJ*. 1997;315(7109):629-634. doi:https://doi.org/10.1136/bmj.315.7109.629

22. Duval S, Tweedie R. A nonparametric "trim and fill" method of accounting for publication bias in meta-analysis. *J Am Stat Assoc*. 2000;95(449):89-98. doi:https://doi.org/10.1080/01621459.2000.10473905

23. Duval S, Tweedie R. Trim and fill: A simple funnel-plot–based method of testing and adjusting for publication bias in meta-analysis. *Biometrics*. 2000;56(2):455-463. doi:https://doi.org/10.1111/j.0006-341X.2000.00455.x

24. StataCorp. *Stata Statistical Software: Release 17*. StataCorp LP; 2021.

25. Palmer TM, Sterne JAC, eds. *Meta-analysis in Stata: An updated collection from the Stata Journal*. 2nd ed. Stata Press; 2016.

26. Guo XC, Wang RY, Cheng CF, et al. [A preliminary investigation of the IQs of 7-13 year-old children from an area with coal burning-related fluoride poisoning]. *Chin J Epidemiol*. 1991;10(2):98-100.

27. Guo XC, Wang RY, Cheng CF, et al. A preliminary investigation of the IQs of 7-13 year-old children from an area with coal burning-related fluoride poisoning. *Fluoride*. 2008a;41:125-128.

28. Li Y, Li X, Wei S. [Effects of high fluoride intake on child mental work capacity: Preliminary investigation into the mechanisms involved]. *J West Chin University Med Sci*. 1994;25(2):188-191.

29. Li Y, Li X, Wei S. Effects of high fluoride intake on child mental work capacity: Preliminary investigation into the mechanisms involved. *Fluoride*. 2008b;41:331-335.

30. Li XS, Zhi JL, Gao RO. Effect of fluoride exposure on the intelligence of children. *Fluoride*. 1995;28:189-192.

31. Li F, Chen X, Huang R, Xie Y. The impact of endemic fluorosis caused by the burning of coal on the development of intelligence in children. *J Environ Health*. 2009;26(4):838-840.

32. Bai A, Li Y, Fan Z, Li X, Li P. [Intelligence and growth development of children in coal-burning-borne arsenism and fluorosis areas: An investigation study]. *Chin J Endemiol*. 2014;33(2):160-163.

33. Zhang P, Cheng L. Effects of coal-burning endemic fluorosis on children's physical development and intelligence. *Chin J Endem Dis Control*. 2015:458-459.

34. Wang J, Yu M, Yang L, Yang X, Deng B. The effect of coal-burning fluoride exposure on children's intelligence and physical development. *J Environ Health*. 2020c;37(11):971-974.

© 2025 Taylor KW et al. *JAMA Pediatrics*.

35. Lou D, Luo Y, Liu J, et al. Refinement impairments of verbal-performance intelligent quotient in children exposed to fluoride produced by coal burning. *Biol Trace Elem Res*. Feb 2021;199(2):482-489. doi:https://dx.doi.org/10.1007/s12011-020-02174-z

36. Wang G, Yang D, Jia F, Wang H. [A study of the IQ levels of four- to seven-year-old children in high fluoride areas]. *Endem Dis Bull*. 1996;11(1):60-62.

37. Wang G, Yang D, Jia F, Wang H. A study of the IQ levels of four- to seven-year-old children in high fluoride areas. *Fluoride*. 2008b;41:340-343.

38. Ren D, Li K, Liu D. [A study of the intellectual ability of 8-14 year-old children in high fluoride, low iodine areas]. *Chin J Control Endem Dis*. 1989;4(4):251.

39. Ren D, Li K, Liu D. A study of the intellectual ability of 8-14 year-old children in high fluoride, low iodine areas. *Fluoride*. 2008;41:319-320.

40. An J, Mei S, Liu A, Fu Y, Wang C. [Effect of high level of fluoride on children's intelligence]. *Chin J Control Endem Dis*. 1992;7(2):93-94.

41. Broadbent JM, Thomson WM, Moffitt TE, Poulton R. Community water fluoridation and intelligence response. *Am J Public Health*. 2015;105:3-4.

42. Xu Y, Lu C, Zhang X. [The effect of fluorine on the level of intelligence in children]. *Endem Dis Bull*. 1994;9(2):83-84.

43. Yao Y. Comparable analysis on the physical and mental development of children in endemic fluorosis area with water improvement and without water improvement. *Lit Inf Prev Med*. 1997;3(1):42-43.

44. Yao L, Zhou J, Wang S, Cui K, Lin F. Analysis on TSH and intelligence level of children with dental Fluorosis in a high fluoride area. *Lit Inf Prev Med*. 1996;2(1):26-27.

45. Seraj B, Shahrabi M, Falahzade M, Falahzade F, Akhondi N. Effect of high fluoride concentration in drinking water on children's intelligence. *J Dent Med*. 2006;19(2):80-86.

46. Seraj B, Shahrabi M, Shadfar M, et al. Effect of high water fluoride concentration on the intellectual development of children in Makoo, Iran. *J Dent*. 2012;9:221-229.

47. Eswar P, Nagesh L, Devaraj CG. Intelligent quotients of 12-14 year old school children in a high and low fluoride village in India. *Fluoride*. 2011;44:168-172.

48. Poureslami HR, Horri A, Garrusi B. A comparative study of the IQ of children age 7-9 in a high and a low fluoride water city in Iran. *Fluoride*. 2011;44:163-167.

49. Shivaprakash PK, Ohri K, Noorani H. Relation between dental fluorosis and intelligence quotient in school children of Bagalkot District. *J Indian Soc Pedod Prev Dent*. 2011;29:117-120.

50. Khan SA, Singh RK, Navit S, et al. Relationship between dental fluorosis and intelligence quotient of school going children in and around Lucknow District: A cross-sectional study. *J Clin Diagn Res*. 2015;9(11):10-15. doi:https://doi.org/10.7860/JCDR/2015/15518.6726

51. Sebastian ST, Sunitha S. A cross-sectional study to assess the intelligence quotient (IQ) of school going children aged 10-12 years in villages of Mysore District, India with different fluoride levels. *J Indian Soc Pedod Prev Dent*. 2015;33:307-311.

52. Mondal D, Dutta G, Gupta S. Inferring the fluoride hydrogeochemistry and effect of consuming fluoride-contaminated drinking water on human health in some endemic areas of Birbhum District, West Bengal. *Environ Geochem Health*. 2016;38:557-576.

© 2025 Taylor KW et al. *JAMA Pediatrics*.

53. Karimzade S, Aghaei M, Mahvi AH. Investigation of intelligence quotient in 9-12 year-old children exposed to high- and low-drinking water fluoride in West Azerbaijan Province, Iran. *Fluoride*. 2014;47:9-14.

54. Sun M, Li S, Wang Y, Li F. [Measurement of intelligence by drawing test among the children in the endemic area of Al-F combined toxicosis]. *J Guiyang Med Coll*. 1991;16(3):204-206.

55. Zhang J, Yao H, Chen Y. [The effect of high levels of arsenic and fluoride on the development of children's intelligence]. *Chin J Public Health*. 1998;17(2):119.

56. Trivedi M, Sangai N, Patel R, Payak M, Vyas S. Assessment of groundwater quality with special reference to fluoride and its impact on IQ of schoolchildren in six villages of the Mundra Region, Kachchh, Gujurat, India. *Fluoride*. 2012;45(4):377-383.

57. Trivedi MH, Verma RJ, Chinoy NJ, Patel RS, Sathawara NG. Effect of high fluoride water on intelligence of school children in India. *Fluoride*. 2007;40:178-183.

58. Wang SY, Zhang HX, Fan W, et al. The effects of endemic fluoride poisoning caused by coal burning on the physical development and intelligence of children. *Fluoride*. 2008a;41:344-348.

59. Chen YX, Han FL, Zhoua ZL, et al. [Research on the intellectual development of children in high fluoride areas]. *Chin J Control Endem Dis*. 1991;6(Suppl):99-100.

60. Chen YX, Han FL, Zhoua ZL, et al. Research on the intellectual development of children in high fluoride areas. *Fluoride*. 2008;41:120-124.

61. Lin F, Ai H, Zhao H, Lin J, Jhiang J, Maimaiti. [High fluoride and low iodine environment and subclinical cretinism in Xinjiang]. *Endem Dis Bull*. 1991;6(2):62-67.

62. Zhao LB, Liang GH, Zhang DN, Wu XR. Effect of a high fluoride water supply on children's intelligence. *Fluoride*. 1996;29:190-192.

63. Lu Y, Sun ZR, Wu LN, Wang X, Lu W, Liu SS. Effect of high-fluoride water on intelligence in children. *Fluoride*. 2000;33:74-78.

64. Hong FG, Cao YX, Yang D, Wang H. [Research on the effects of fluoride on child intellectual development under different environmental conditions]. *Chin Prim Health Care*. 2001;15(3):56-57.

65. Hong FG, Cao YX, Yang D, Wang H. Research on the effects of fluoride on child intellectual development under different environmental conditions. *Fluoride*. 2008;41:156-160.

66. Hong F, Wang Hui Yang Dong Zhang Z. Investigation on the intelligence and metabolism of iodine and fluoride in children with high iodine and fluoride. *Chin J Endem Dis Control*. 2001b:12-14.

67. Wang X, Wang L, Hu P, Guo X, Luo X. [Effects of high iodine and high fluorine on children's intelligence and thyroid function]. *Chin J Endemiol*. 2001;20(4):288-290.

68. Li YP, Jing XY, Chen D, Lin L, Wang ZJ. [Effects of endemic fluoride poisoning on the intellectual development of children in Baotou]. *Chin J Public Health Manag*. 2003;19(4):337-338.

69. Li YP, Jing XY, Chen D, Lin L, Wang ZJ. Effects of endemic fluoride poisoning on the intellectual development of children in Baotou. *Fluoride*. 2008c;41:161-164.

70. Xiang Q, Liang Y, Chen L, et al. Effect of fluoride in drinking water on children's intelligence. *Fluoride*. 2003a;36:84-94.

© 2025 Taylor KW et al. *JAMA Pediatrics*.

71. Wang SX, Wang ZH, Cheng XT, et al. [Investigation and evaluation on intelligence and growth of children in endemic fluorosis and arsenism areas]. *Chin J Endemiol*. 2005;24:179-182.

72. Wang Z, Wang S, Zhang X, Li J, Zheng X, Hu C. Investigation of children's growth and development under long-term fluoride exposure. *Chin J Control Endem Dis*. 2006;21(4):239-241.

73. Fan Z, Dai H, Bai A, Li P, Li T, Li G. The effect of high fluoride exposure in children's intelligence. *J Environ Health*. 2007;24(10):802-803.

74. Wang SX, Wang ZH, Cheng XT, et al. Arsenic and fluoride exposure in drinking water: Children's IQ and growth in Shanyin County, Shanxi Province, China. *Environ Health Perspect*. 2007;115:643-647.

75. Li X, Hou G, Yu B, Yuan C, Liu Y, L Z. [Investigation and analysis of children's IQ and dental fluorosis in high fluoride area]. *Chin J Pest Control*. 2010;26(3):230-231.

76. Ding Y, Sun H, Han H, et al. The relationships between low levels of urine fluoride on children's intelligence, dental fluorosis in endemic fluorosis areas in Hulunbuir, Inner Mongolia, China. *J Hazard Mater*. 2011;186:1942-1946.

77. Kang J, Cheng Y, Wu K, Lin S, He G, Jin Y. [Effect of exposure to fluoride and arsenic in drinking water of Hangjinhouqi on children's intelligence]. *Chin School Health*. 2011:679-681.

78. Wang S, Zhu X. Investigation and analysis of intelligence level of children in high fluoride area. *Chin J Endem Dis Control*. 2012b;27(1):67-68.

79. Kundu H, Basavaraj P, Singla A, Gupta R, Singh K, Jain S. Effect of fluoride in drinking water on children's intelligence in high and low fluoride areas of Delhi. *J Indian Assoc Public Health Dent*. Apr 2015;13(2):116-121.

80. Zhang S, Zhang X, Liu H, et al. Modifying effect of COMT gene polymorphism and a predictive role for proteomics analysis in children's intelligence in endemic fluorosis area in Tianjin, China. *Toxicol Sci*. 2015b;144:238-245.

81. Zhang P, Cheng L. Effect of coal-burning endemic fluorosis on children's physical development and intellectual level. *Chin J Endem Dis Control*. 2015c;30(6):458-459.

82. Aravind A, Dhanya RS, Narayan A, Sam G, Adarsh VJ, Kiran M. Effect of fluoridated water on intelligence in 10-12-year-old school children. *J Int Soc Prev Community Dent*. Dec 2016;6(Suppl 3):S237-S242. doi:https://doi.org/10.4103/2231-0762.197204

83. Das K, Mondal NK. Dental fluorosis and urinary fluoride concentration as a reflection of fluoride exposure and its impact on IQ level and BMI of children of Laxmisagar, Simlapal Block of Bankura District, W.B., India. *Environ Monit Assess*. 2016;188:218.

84. Bashash M, Thomas D, Hu H, et al. Prenatal fluoride exposure and cognitive outcomes in children at 4 and 6-12 years of age in Mexico. *Environ Health Perspect*. 2017;125(9):1-12. doi:https://doi.org/10.1289/ehp655

85. Razdan P, Patthi B, Kumar JK, Agnihotri N, Chaudhari P, Prasad M. Effect of fluoride concentration in drinking water on intelligence quotient of 12-14-year-old children in Mathura District: A cross-sectional study. *J Int Soc Prev Community Dent*. 2017;7:252-258. doi:https://doi.org/10.4103/jispcd.JISPCD_201_17

86. Valdez Jimenez L, Lopez Guzman OD, Cervantes Flores M, et al. In utero exposure to fluoride and cognitive development delay in infants. *Neurotoxicology*. Mar 2017;59:65-70. doi:https://dx.doi.org/10.1016/j.neuro.2016.12.011

© 2025 Taylor KW et al. *JAMA Pediatrics*.

87. Cui Y, Zhang B, Ma J, et al. Dopamine receptor D2 gene polymorphism, urine fluoride, and intelligence impairment of children in China: A school-based cross-sectional study. *Ecotoxicol Environ Saf*. 2018;165:270-277. doi:https://doi.org/10.1016/j.ecoenv.2018.09.018

88. Zhao Y, Cui Y, Yu J, et al. Study on the relationship between water-borne high iodine and thyroid hormone and children's intelligence level. *J Environ Health*. 2018:6-9.

89. Green R, Lanphear B, Hornung R, et al. Association between maternal fluoride exposure during pregnancy and IQ scores in offspring in Canada. *JAMA Pediatr*. 2019:E1-E9.

90. Cui Y, Yu J, Zhang B, Guo B, Gao T, Liu H. The relationships between thyroid-stimulating hormone and/or dopamine levels in peripheral blood and IQ in children with different urinary iodine concentrations. *Neurosci Lett*. 2020;729:134981. doi:https://doi.org/10.1016/j.neulet.2020.134981

91. Till C, Green R, Flora D, et al. Fluoride exposure from infant formula and child IQ in a Canadian birth cohort. *Environ Int*. 2020;134:105315. doi:https://doi.org/10.1016/j.envint.2019.105315

92. Cantoral A, Tellez-Rojo MM, Malin AJ, et al. Dietary fluoride intake during pregnancy and neurodevelopment in toddlers: A prospective study in the progress cohort. *Neurotoxicology*. Dec 2021;87:86-93. doi:https://dx.doi.org/10.1016/j.neuro.2021.08.015

93. Guo B, Yu J, Cui Y, et al. DBH gene polymorphism, iodine and fluoride and their interactions and their interaction with children’s intelligence. *J Environ Hygiene*. 2021;11(02):134-140.

94. Ibarluzea J, Gallastegi M, Santa-Marina L, et al. Prenatal exposure to fluoride and neuropsychological development in early childhood: 1-to 4 years old children. *Environ Res*. Oct 8 2021:112181. doi:https://dx.doi.org/10.1016/j.envres.2021.112181

95. Prabhakar A, Abdulkhayarkutty K, Cheruvallil SV, Sudhakaran P. Effect of endemic fluorosis on cognitive function of school children in Alappuzha District, Kerala: A cross sectional study. *Ann Indian Acad Neurol*. 2021;24(5):715-720. doi:https://doi.org/10.4103/aian.AIAN_850_20

96. Saeed M, Rehman MYA, Farooqi A, Malik RN. Arsenic and fluoride co-exposure through drinking water and their impacts on intelligence and oxidative stress among rural school-aged children of Lahore and Kasur districts, Pakistan. *Environ Geochem Health*. Nov 9 2021;doi:https://dx.doi.org/10.1007/s10653-021-01141-4

97. Wang R, He N, Wang Y, Hou G, Zhang P-J. Investigation and analysis of children's dental fluorosis and IQ level in high fluoride district of Hengshui City. *Med Anim Control*. 2021;37(8):796-800.

98. Zhao L, Yu C, Lv J, et al. Fluoride exposure, dopamine relative gene polymorphism and intelligence: A cross-sectional study in China. *Ecotoxicol Environ Safety*. Feb 2021;209:111826. doi:https://dx.doi.org/10.1016/j.ecoenv.2020.111826

99. Ahmad MS, Sarker MNI, Ahmad MN, Ali M, Sadiq, Abbas M. Does high fluoride intake cause low IQ? A case of Islamic religious schools (Madrassas) in rural and urban areas of Sindh, Pakistan. *Fluoride*. 2022;55(1):49-62.

100. Feng Z, An N, Yu F, et al. Do methylenetetrahydrofolate dehydrogenase, cyclohydrolase, and formyltetrahydrofolate synthetase 1 polymorphisms modify changes in intelligence of school-age children in areas of endemic fluorosis? *Chin Med J*. 2022;135(15):1846-1854. doi:https://doi.org/10.1097/CM9.0000000000002062

101. Goodman CV, Bashash M, Green R, et al. Domain-specific effects of prenatal fluoride exposure on child IQ at 4, 5, and 6–12 years in the ELEMENT cohort. *Environ Res*. 2022a;211:112993. doi:https://doi.org/10.1016/j.envres.2022.112993

© 2025 Taylor KW et al. *JAMA Pediatrics*.

102. Tian W, Zhao A, Yu Y, et al. The relationship between IQ and urinary fluoride in children in endemic fluorosis endemic areas polluted by coal burning. *Chin J Endem Dis*. 2022;41(2):117-119. doi:https://doi.org/10.3760/cma.j.cn231583-20210125-00025

103. Dewey D, England-Mason G, Ntanda H, et al. Fluoride exposure during pregnancy from a community water supply is associated with executive function in preschool children: A prospective ecological cohort study. *Sci Total Environ*. 2023;891:164322. doi:https://doi.org/10.1016/j.scitotenv.2023.164322

104. Grandjean P, Meddis A, Nielsen F, et al. Dose dependence of prenatal fluoride exposure associations with cognitive performance at school age in three prospective studies. *Eur J Public Health*. 2023;34(1):143-419. doi:https://doi.org/10.1093/eurpub/ckad170

105. Lin YY, Hsu WY, Yen CE, Hu SW. Association of dental fluorosis and urinary fluoride with intelligence among schoolchildren. *Children*. 2023;10(6):987. doi:https://doi.org/10.3390/children10060987

106. Xia Y, Xu Y, Shi M, et al. Effects of high-water fluoride exposure on IQ levels in school-age children: A cross-sectional study in Jiangsu, China. *Exposure and Health*. 2023;doi:https://doi.org/10.1007/s12403-023-00597-2

107. Qin L, Huo S, Chen R, Chang Y, Zhao M. [Using the Raven's Standard Progressive Matrices to determine the effects of the level of fluoride in drinking water on the intellectual ability of school-age children]. *Chin J Endem Dis*. 1990;5(4):203-204.

108. Qin LS, Huo SY, Chen RL, Chang YZ, Zhao MY. Using the Raven's Standard Progressive Matrices to determine the effects of the level of fluoride in drinking water on the intellectual ability of school-age children. *Fluoride*. 2008;41:115-119.

109. Yang Y, Wang X, X. G, Hu P. [The effects of high levels of fluoride and iodine on child intellectual ability and the metabolism of fluoride and iodine]. *Chin J Epidemiol*. 1994;15(4):296-298.

110. Yang Y, Wang X, Guo X, Hu P. The effects of high levels of fluoride and iodine on child intellectual ability and the metabolism of fluoride and iodine. *Fluoride*. 2008;41:336-339.

111. Wang SY, Zhang HX, Fan W, et al. [The effects of endemic fluoride poisoning caused by coal burning on the physical development and intelligence of children]. *J Appl Clin Ped*. 2005b;20(9):897-899.

112. Rocha-Amador D, Navarro ME, Carrizales L, Morales R, Calderon J. Decreased intelligence in children and exposure to fluoride and arsenic in drinking water. *Cad Saude Publica*. 2007;23(Suppl 4):S579-587.

113. Liu SL, Lu Y, Sun ZR, et al. [Report on the intellectual ability of children living in high-fluoride water areas]. *Chin J Control Endem Dis*. 2000;15(4):231-232.

114. Liu SL, Lu Y, Sun ZR, et al. Report on the intellectual ability of children living in high-fluoride water areas. *Fluoride*. 2008;41:144-147.

115. Sudhir KM, Chandu GN, Prashant GM, Subba Reddy VV. Effect of fluoride exposure on intelligence quotient (IQ) among 13-15 year old school children of known endemic area of fluorosis, Nalgonda District, Andhra Pradesh. *J Indian Assoc Public Health Dent*. 2009;2009(13):88-94.

116. He MX, Zhang CN. [Investigation of children's intelligence quotient and dental fluorosis in drinking water-type of endemic fluorosis area in Pucheng County, Shaanxi Province before and after drinking water change]. *Chin J Endemiol*. 2010;29:547-548.

© 2025 Taylor KW et al. *JAMA Pediatrics*.

117. Xiang Q, Liang Y, Chen B, Chen L. Analysis of children's serum fluoride levels in relation to intelligence scores in a high and low fluoride water village in China. *Fluoride*. 2011;44:191-194.

118. Saxena S, Sahay A, Goel P. Effect of fluoride exposure on the intelligence of school children in Madhya Pradesh, India. *J Neurosci Rural Pract*. 2012;3:144-149.

119. Wang G, Gao M, Zhang M, Yang M, Xiang Q. Correlation between total fluoride intake and children's IQ. *J Southeast Univ Med Ed*. 2012:743-746.

120. Nagarajappa R, Pujara P, Sharda AJ, et al. Comparative assessment of intelligence quotient among children living in high and low fluoride areas of Kutch, India: A pilot study. *Iran J Public Health*. 2013;42:813-818.

121. Pratap SV, Singh CD, Sandeep T, et al. A correlation between serum vitamin, acetylcholinesterase activity and IQ in children with excessive endemic fluoride exposure in Rajasthan, India. *Int Res J Medical Sci*. 2013;1(3):12-16.

122. Asawa K, Pujara P, Thakkar JP, et al. Assessment of intelligence quotient among schoolchildren of fishermen community of Kutch, Gujarat, India. *Int Marit Health*. 2014;65(2):73-8. doi:https://doi.org/10.5603/imh.2014.0017

123. Wei N, Li Y, Deng J, Xu S, Guan Z. [The effects of comprehensive control measures on intelligence of school-age children in coal-burning-borne endemic fluorosis areas]. *Chin J Endemiol*. 2014;33(3):320-322.

124. Choi AL, Zhang Y, Sun G, et al. Association of lifetime exposure to fluoride and cognitive functions in Chinese children: A pilot study. *Neurotoxicol Teratol*. 2015;47:96-101.

125. Jin T, Han T, Wei Y, Wu Y, Wang Z, Zhang H. Investigation on working memory level of children aged 8-12 years in coal-burning fluorosis area. *J Environ Health*. 2016:409-411.

126. Kumar R, Gupta VK, Kumar A, Shetty R, Singh M, Pandey V. Evaluation of correlation of dental fluorosis and cognitive status. *Ann Int Med Dental Res*. 2016;2(4):155.

127. Jin T, Wang Z, Wei Y, Wu Y, Han T, Zhang H. Investigation on intelligence level of children aged 8-12 years old in coal-burning fluorosis area. *J Environ Health*. 2017:229-231.

128. Wang G, Zhang M, Wang Q, et al. Investigation on the relationship between serum fluoride content and IQ of children before and after reducing fluoride to water. *Capital Pub Health*. 2017:274-277.

129. Luo Y, Ma R, Liu Z, Guan Z, Lou D, Zheng D. Intelligence investigation and forensic significance of children in coal-burning fluorosis area. *Chin J Forensic Med*. 2018:590-593.

130. Naik SP, Bankur PK, Sathe S, Haris PMM, Kadar N, Satyanarayan A. Impact of fluoridated water on intelligence quotient levels of school children: A exploratory study. *Int J Oral Care Res*. 2018;6(1):63-66.

131. Sharma P, Bhardwaj AK, Singh M, Kumar D, Sharma A, Grover A. Does fluorosis affect the intelligence profile of children? A cross sectional analysis of school children of district Una, Himachal Pradesh, India. *Int J Community Med Public Health*. 2018;5(3):1047-53. doi:http://dx.doi.org/10.18203/2394-6040.ijcmph20180759

132. Soto-Barreras U, Escalante-Villalobos KY, Holguin-Loya B, et al. Effect of fluoride in drinking water on dental caries and IQ in children. *Fluoride*. 2019;52:474-482.

133. Zhao Q, Niu Q, Chen J, et al. Roles of mitochondrial fission inhibition in developmental fluoride neurotoxicity: Mechanisms of action in vitro and associations with cognition in rats and children. *Arch Toxicol*. Jan 18 2019;93(3):709-726. doi:https://doi.org/10.1007/s00204-019-02390-0

© 2025 Taylor KW et al. *JAMA Pediatrics*.

134. Zhou G, Tang S, Yang L, et al. Effects of long-term fluoride exposure on cognitive ability and the underlying mechanisms: Role of autophagy and its association with apoptosis. *Toxicol Appl Pharmacol*. 2019;378:114608. doi:https://doi.org/10.1016/j.taap.2019.114608

135. Xu K, An N, Huang H, et al. Fluoride exposure and intelligence in school-age children: evidence from different windows of exposure susceptibility. *BMC Public Health*. Nov 4 2020;20(1):1657. doi:https://dx.doi.org/10.1186/s12889-020-09765-4

136. Zhao Q, Tian Z, Zhou G, et al. SIRT1-dependent mitochondrial biogenesis supports therapeutic effects of resveratrol against neurodevelopment damage by fluoride. *Theranostics*. 2020;10:4822-4838. doi:https://doi.org/10.7150/thno.42387

137. Aggeborn L, Öhman M. The effects of fluoride in drinking water. *J Polit Econ*. 2020;129(2):465-491. doi:https://dx.doi.org/10.1086/711915

138. Farmus L, Till C, Green R, et al. Critical windows of fluoride neurotoxicity in Canadian children. *Environ Res*. Sep 2021;200:111315. doi:https://doi.org/10.1016/j.envres.2021.111315

139. Wang R, He N, Hou G, Zhang P, Jin W, Wang Y. Hengshui Survey and analysis of the mental health status of children aged 8-12 years in the high-fluoride area of Shanghai. *Med Anim Control*. 2021b;37(06):583-586.

140. Wang S, Zhao Q, Li G, et al. The cholinergic system, intelligence, and dental fluorosis in school-aged children with low-to-moderate fluoride exposure. *Ecotoxicol Environ Safety*. 2021/12/25/ 2021;228:112959. doi:https://doi.org/10.1016/j.ecoenv.2021.112959

141. Yani SI, Seweng A, Mallongi A, et al. The influence of fluoride in drinking water on the incidence of fluorosis and intelligence of elementary school students in Palu City. *Gac Sanit*. 2021/01/01/ 2021;35:S159-S163. doi:https://doi.org/10.1016/j.gaceta.2021.07.010

142. Yu X, Xia L, Zhang S, et al. Fluoride exposure and children's intelligence: Gene-environment interaction based on SNP-set, gene and pathway analysis, using a case-control design based on a cross-sectional study. *Environ Int*. 2021;155:106681. doi:https://doi.org/10.1016/j.envint.2021.106681

143. Zhou G, Zhao Q, Luo C, et al. Low-moderate fluoride exposure and intelligence among Chinese school-aged children: Role of circulating mtDNA content. *Sci Total Environ*. Sep 10 2021;786:147330. doi:https://dx.doi.org/10.1016/j.scitotenv.2021.147330

144. Goodman CV, Hall M, Green R, et al. Iodine status modifies the association between fluoride exposure in pregnancy and preschool boys' intelligence. *Nutrients*. 2022b;14(14):2920. doi:https://doi.org/10.3390/nu14142920

145. Kaur D, Kaur K, Sharma A, Goyal H, Pahuja A, Solanki D. Assessment of fluoride content in water and its impact on the intelligence quotient of school children aged 12-13 years. *Cureus*. 2022;14(10):e30157. doi:https://doi.org/10.7759/cureus.30157

146. Tamayo de la Cruz J, Pérez LDlS, Tejada FC. Relationship among dental fluorosis, intellectual quotient and academic performance. *Fluoride*. 2022;55(1):41-48.

147. Rodriguez-Barranco M, Tobias A, Redondo D, Molina-Portillo E, Sanchez M-J. Standardizing effect size from linear regression models with log-transformed variables for meta-analysis. *BMC Med Res Methodol*. 2017;17(44)doi:https://doi.org/10.1186/s12874-017-0322-8

148. Higgins JPT, Chandler TJ, Cumpston M, Li T, Page MJ, Welch VA, eds. *Cochrane Handbook for Systematic Reviews of Interventions version 6.2 (updated February 2021)*. Cochrane; 2021.

149. Lin L, Chu H. Quantifying publication bias in meta-analysis. *Biometrics*. 2018;74(3):785-794. doi:https://doi.org/10.1111/biom.12817

© 2025 Taylor KW et al. *JAMA Pediatrics*.

150. Shi L, Lin L. The trim-and-fill method for publication bias: Practical guidelines and recommendations based on a large database of meta-analyses. *Medicine*. 2019;98(23):e15987. doi:https://doi.org/10.1097/md.0000000000015987

151. Choi AL, Sun G, Zhang Y, Grandjean P. Developmental fluoride neurotoxicity: A systematic review and meta-analysis. *Environ Health Perspect*. 2012;120:1362-1368.

152. Duan Q, Jiao J, Chen X, Wang X. Association between water fluoride and the level of children's intelligence: A dose-response meta-analysis. *Public Health*. 2018;154:87-97. doi:https://doi.org/10.1016/j.puhe.2017.08.013

153. Kumar JV, Moss ME, Liu H, Fisher-Owens S. Association between low fluoride exposure and children's intelligence: A meta-analysis relevant to community water fluoridation. *Public Health*. Jun 2023;219:73-84. doi:https://doi.org/10.1016/j.puhe.2023.03.011

154. Tang QQ, Du J, Ma HH, Jiang SJ, Zhou XJ. Fluoride and children's intelligence: A meta-analysis. *Biol Trace Elem Res*. 2008;126(1-3):115-20. doi:https://doi.org/10.1007/s12011-008-8204-x

155. Veneri F, Vinceti M, Generali L, et al. Fluoride exposure and cognitive neurodevelopment: Systematic review and dose-response meta-analysis. *Environ Res*. 2023;221:115239. doi:https://doi.org/10.1016/j.envres.2023.115239

156. Miranda GHN, Alvarenga MOP, Ferreira MKM, et al. A systematic review and meta-analysis of the association between fluoride exposure and neurological disorders. *Sci Rep*. 2021;11(1):22659. doi:https://doi.org/10.1038/s41598-021-99688-w

157. Jüni P, Witschi A, Bloch R, Egger M. The hazards of scoring the quality of clinical trials for meta-analysis. *JAMA*. 1999;282(11):1054-60. doi:https://doi.org/10.1001/jama.282.11.1054

158. Singla VI, Sutton PM, Woodruff TJ. The Environmental Protection Agency Toxic Substances Control Act systematic review method may curtail science used to inform policies, with profound implications for public health. *Am J Public Health*. 2019;109(7):982-984. doi:https://doi.org/10.2105/ajph.2019.305068

159. Stang A. Critical evaluation of the Newcastle-Ottawa scale for the assessment of the quality of nonrandomized studies in meta-analyses. *Eur J Epidemiol*. 2010;25(9):603-5. doi:https://doi.org/10.1007/s10654-010-9491-z

160. Arroyave WD, Mehta SS, Guha N, et al. Challenges and recommendations on the conduct of systematic reviews of observational epidemiologic studies in environmental and occupational health. *J Expo Sci Environ Epidemiol*. 2021;31(1):21-30. doi:https://doi.org/10.1038/s41370-020-0228-0

161. Savitz DA, Wellenius GA, Trikalinos TA. The problem with mechanistic risk of bias assessments in evidence synthesis of observational studies and a practical alternative: Assessing the impact of specific sources of potential bias. *Am J Epidemiol*. Sep 1 2019;188(9):1581-1585. doi:https://doi.org/10.1093/aje/kwz131

162. Steenland K, Schubauer-Berigan MK, Vermeulen R, et al. Risk of bias assessments and evidence syntheses for observational epidemiologic studies of environmental and occupational exposures: Strengths and limitations. *Environ Health Perspect*. 2020;128(9):095002. doi:https://doi.org/10.1289/EHP6980

163. Sterne JAC, Hernán MA, McAleenan A, Reeves BC, Higgins JPT. Chapter 25: Assessing risk of bias in a non-randomized study. In: Higgins JPT, Thomas J, Chandler J, Cumpston M, Li T, Page MJ, Welch VA, editors. Cochrane Handbook for Systematic Reviews of Interventions version 6.4 (updated August 2023). Cochrane; 2023.

© 2025 Taylor KW et al. *JAMA Pediatrics*.

164. Guth S, Hüser S, Roth A, et al. Toxicity of fluoride: critical evaluation of evidence for human developmental neurotoxicity in epidemiological studies, animal experiments and in vitro analyses. *Arch Toxicol*. 2020;94(5):1375-1415. doi:https://doi.org/10.1007/s00204-020-02725-2

165. Do LG, Spencer AJ, Sawyer A, et al. Early childhood exposures to fluorides and child behavioral development and executive function: A population-based longitudinal study. *J Dent Res*. Jan 2023;102(1):28-36. doi:https://doi.org/10.1177/00220345221119431

© 2025 Taylor KW et al. *JAMA Pediatrics*.

eAppendix 2. Details for Low Risk-of-Bias Studies

Xiang et al. (2003)[1]

Study Details

Study design: Cross-sectional

Population: Children aged 8–13 years

Study area: Wamiao and Xinhuai villages located in Sihong County, Jiangsu Province, China

Sample size: 512 school children

Data relevant to the review: Comparison of IQ (mean and distribution) between Wamiao County (a severe endemic fluorosis area) and Xinhuai County (a non-endemic fluorosis area); additional breakdown of the Wamiao area into five water fluoride exposure groups. Correlations between water fluoride and IQ scores.

Reported association with fluoride exposure: Yes: Significantly lower IQ scores observed with water fluoride levels of 1.53 mg/L or higher. The percentage of subjects with IQ scores below 80 was significantly increased at water fluoride levels of 2.46 mg/L or higher. Significant inverse correlation between urinary fluoride and IQ scores (r = –0.164). Mean IQ scores for children in the non-endemic region (100.41 ± 13.21) were significantly higher than the endemic region (92.02 ± 13.00).

Risk of Bias

Author contacts:

Authors were not contacted for additional information because it was not necessary.

Population selection:

Rating: Probably low risk of bias (+)

Summary: Two villages, Wamiao and Xinhuai, located 64 km apart in Sihong County, Jiangsu Province, were selected for this study, which was conducted between September and December 2002. Wamiao is located in a severe fluorosis endemic area, and Xinhuai is located in a non-endemic fluorosis area. Neither village has fluoride pollution from burning coal or other industrial sources. All eligible children in each village were included; children who had been absent from either village for 2 years or longer or who had a history of brain disease or head injury were excluded. In Wamiao, 93% of the children (222 out of 238) were included in the study; in Xinhuai, 95% were included (290 out of 305). The children in Wamiao were divided into five subgroups according to the level of fluoride in their drinking water: <1.0 mg/L (group A), 1.0–1.9 mg/L (group B), 2.0–2.9 mg/L (group C), 3.0–3.9 mg/L (group D), and >3.9 mg/L (group E). Children in Xinhuai (0.18–0.76 mg/L in the drinking water) served as a control group (group F). Demographic characteristics are not presented, and statistical analyses are not adjusted, but mean IQ scores are stratified by age, sex, family income, and parental education.

Basis for rating: Probably low risk of bias based on indirect evidence that the exposure groups were similar and were recruited using the same methods within the same time frame, with direct evidence that there was no difference in participation/response rates.

Confounding:

Rating: Probably low risk of bias (+)

Summary: Although information was stated to be collected on personal characteristics, medical history, education levels of the children and parents, family SES, and lifestyle,

© 2025 Taylor KW et al. *JAMA Pediatrics*.

only sex, age, family income, and parental education were considered. Potential co-exposures, such as arsenic, were not addressed. A separate publication in 2003 [[2], letter to the editor] indicated that blood lead levels were not significantly different between the two areas. Although arsenic was not addressed specifically in this publication, Xiang et al. (2013)[3] measured both fluoride and arsenic in the Wamiao and Xinhuai areas. Xinhuai (the low-fluoride area) had significantly higher arsenic levels compared with Wamiao (the endemic fluorosis area). This is likely to bias the association toward the null; however, the study observed a significantly lower IQ score in the endemic fluorosis area. Iodine was tested in a subset of the children and found not to be significantly different between the two groups.

Potentially important study-specific covariates: Arsenic often occurs in the drinking water along with fluoride in some Chinese populations; however, based on information provided in Xiang et al. (2013)[3], arsenic concentrations were higher in the low-fluoride area compared with the high fluoride area. Because there were significant effects on IQ observed in the high fluoride areas, the impact of co-exposure to arsenic is less of a concern. The presence of arsenic in the control village may cause an underestimation of the effect of fluoride, but despite this potential impact, there was still a significant association between fluoride exposure and IQ.

Direction/magnitude of effect size: Presence of arsenic in this study population would potentially bias the association toward the null.

Basis for rating: Probably low risk of bias because there is indirect evidence that the key covariates were taken into account, methods used for collecting the information were valid and reliable, and co-exposures to arsenic and lead and iodine deficiency were not attributing to the effect observed in this area. The potential for bias toward the null, combined with the reported significant association increases confidence in the observed effect.

Attrition:

Rating: Definitely low risk of bias (++)

Summary: Data are complete. IQ results were reported for all 512 children included in the study (222 in the endemic area and 290 in the nonendemic area).

Basis for rating: Definitely low risk of bias based on direct evidence that there was no attrition.

Exposure:

Rating: Probably low risk of bias (+)

Summary: Exposure was based on drinking water and urinary levels of fluoride. The two study areas were selected to reflect a severe endemic area and a non-endemic area. Drinking water was collected from wells, and early-morning spot urine samples were collected from a randomly selected subsample of children. Both water and urine samples were measured using fluoride ion-selective electrode, but no quality control was discussed. Both absolute and creatinine-adjusted urine results were reported.

Direction/magnitude of effect size: There is potential for exposure misclassification because only current levels were assessed. Migration of subjects in or out of the area was not assessed, but the study authors noted that if the children had been absent from the village for 2 or more years, they were excluded. Misclassification would likely be non-differential, which could likely bias the association in either direction.

Basis for rating: Probably low risk of bias based on indirect evidence that exposure was consistently assessed using well-established methods that directly measured exposure.

Outcome:

Rating: Definitely low risk of bias (++)

Summary: The IQ of each child was measured with the Combined Raven's Test for Rural China (CRT-RC) (++ for methods). The test was stated to be administered to the children independently in a school classroom, in a double-blind manner, under the supervision of an examiner and two assistants, and in accordance with the directions of the CRT-RC manual regarding test administration conditions, instructions to be given, and test environment (++ for blinding). Overall rating = ++

Basis for rating: Definitely low risk of bias based on direct evidence that the outcome was assessed using instruments that were valid and reliable in the study population, and that the outcome assessor was blind to participants' fluoride exposure.

Selective Reporting:

Rating: Definitely low risk of bias (++)

Summary: All outcomes outlined in the abstract, introduction, and methods are reported in sufficient detail.

Basis for rating: Definitely low risk of bias based on direct evidence that all measured outcomes were reported.

Other potential threats:

Rating: Probably low risk of bias (+)

Summary:

Statistical analyses: There is no mention of the tests conducted, but data were stated to be analyzed using SAS, suggesting appropriate tests were applied. Results provided in the tables indicate that t-tests comparing IQ values between the villages (overall and by sex) were conducted, but it was not reported that heterogeneity of variance was assessed. In addition, correlations between IQ and age, family income, and parents' education level were tested with Pearson's correlation. There is no evidence that a test for trend was conducted to evaluate the stated "significant inverse concentration-response relationship between the fluoride level in drinking water and the IQ of children."

A potential concern raised by the NASEM [4] committee's review was the lack of accounting for relationships in exposure between persons from the same village. Given only two villages were included and the analyses consisted of village-level comparisons (no use of individual-level covariate data), it is likely that the standard error of the difference in mean IQ between fluoride in water exposure groups will be biased, making differences appear stronger than they actually are. Without controlling for village effects and given the large differences in fluoride concentrations and IQ levels between villages, the apparent dose-response relationship could be due to a village effect in addition to a fluoride effect. However, a dose-response relationship is apparent within the "exposed" village, diminishing the concern for a village-only effect and likely minimizing the impact on the effect estimates.

Other potential concerns: None identified.

Basis for rating: Probably low risk of bias based on indirect evidence that statistical analyses were appropriate and that there were no other threats of risk of bias.

Basis for classification as low risk-of-bias study overall: Definitely or probably low risk-of-bias ratings in confounding, exposure, and outcome. Study strengths include individual exposure

© 2025 Taylor KW et al. *JAMA Pediatrics*.

assessment measurements and blinding of outcome assessor to exposure but is limited by the cross-sectional study design and lack of accounting for urine dilution. All key covariates were considered in the study design or analysis, but there is potential for the presence of arsenic to bias the association toward the null.

Ding et al. (2011)[5]

Study Details

Study design: Cross-sectional

Population: Elementary school children aged 7–14 years old

Study area: Hulunbuir City, Inner Mongolia, China

Sample size: 331 school children

Data relevant to the review: IQ mean difference based on 10 categories of urine fluoride. Adjusted associations between urinary fluoride and IQ scores.

Reported association with fluoride exposure: Yes: Significant inverse association between urinary fluoride and IQ score (adjusted β = −0.59 per 1-mg/L increase; 95% CI: −1.09, −0.08).

Risk of Bias

Author contacts:

Authors were not contacted for additional information because it was not necessary.

Population selection:

Rating: Probably low risk of bias (+)

Summary: The study randomly selected 7–14-year-olds (n=340) from four nearby elementary schools in Hulunbuir. The four elementary schools appeared to be very similar in teaching quality. The study authors noted that they followed the principles of matching social and natural factors like economic situation, educational standards, and geological environments as much as possible; however, how this was done is unclear and no table of study subject characteristics by group was provided.

Basis for rating: Probably low risk of bias based on indirect evidence that the exposure groups were similar and were recruited within the same time frame using the same methods, with no evidence of differences in participation/response rates.

Confounding:

Rating: Probably high risk of bias (−)

Summary: It was noted that none of the four sites had other potential neurotoxins, including arsenic, in their drinking water. Details were not provided, except for a reference supporting the statement. In addition, iodine deficiency was noted as not being an issue in any of the four areas. Age was the only key covariate adjusted for in the regression model. Although dental fluorosis severity by % female was reported, not enough data were provided to determine whether sex should have been considered in the regression model. The study authors note that future studies will include covariates such as parents' educational attainment, mother's age at delivery, and household income.

Potentially important study-specific covariates: Sex

Direction/magnitude of effect size: There is not enough information to determine whether there was an effect from sex. There were some differences in dental fluorosis level by

© 2025 Taylor KW et al. *JAMA Pediatrics*.

sex, but it is unclear how this might impact the results or whether the distribution of sex differed by age.

Basis for rating: Probably high risk of bias based on indirect evidence that there were differences in sex that were not considered in the study design or analyses.

Attrition:

Rating: Definitely low risk of bias (++)

Summary: Data were relatively complete (i.e., <5% loss). Of the 340 subjects selected for inclusion, 5 were excluded because they lived in the area for less than a year with an additional 4 not consenting to participate.

Basis for rating: Definitely low risk of bias based on direct evidence that exclusion of subjects from analysis was adequately addressed, and reasons were documented when subjects were removed from the study or excluded from analyses.

Exposure:

Rating: Probably low risk of bias (+)

Summary: Spot urine samples were collected and measured using China CDC standards. All samples were analyzed twice using a fluoride ion-selective electrode. Recovery rates were specified as 95%–105% with an LOD of 0.05 mg/L. Water samples were collected from small-scale central water supply systems and tube wells with handy pumps and were processed using standard methods, similar to the urine samples. Quality assurance validation was reported. A blind professional examiner evaluated the children for dental fluorosis using Dean's Index. All urine and water samples were above the LOD. Urine levels were the primary exposure assessment measures used in the analysis. The study authors did not account for urinary dilution in the spot samples. The mean urine fluoride concentration was correlated with the dental fluorosis levels.

Direction/magnitude of effect size: Spot urine samples that did not account for dilution could have exposure misclassification. The misclassification is likely non-differential, and the potential direction of bias is unknown.

Basis for rating: Probably low risk of bias based on indirect evidence that exposure was consistently assessed using well-established methods that directly measure exposure.

Outcome:

Rating: Probably low risk of bias (+)

Summary: IQ was determined using the Combined Raven's Test–The Rural in China (CRT-RC3) (++ for methods). Although blinding was not reported, it is unlikely that the IQ assessors had knowledge of the children's urine levels or even of the water levels from the four sites, as these were sent to a separate lab for testing (+ for blinding). Overall rating for methods and blinding = +.

Basis for rating: Probably low risk of bias based on indirect evidence that the outcome was assessed using instruments that were valid and reliable in the study population, and that the outcome assessors were blind to participants' fluoride exposure.

Selective Reporting:

Rating: Definitely low risk of bias (++)

Summary: All outcomes outlined in the abstract, introduction, and methods are reported in sufficient detail.

Basis for rating: Definitely low risk of bias based on direct evidence that all measured outcomes were reported.

© 2025 Taylor KW et al. *JAMA Pediatrics*.

Other potential threats:

Rating: Probably low risk of bias (+)

Summary:

Statistical analyses: Statistical analyses were reasonable (ANOVA and multiple linear regression), but consideration of homogeneity of variance was not reported. The NASEM committee's review NASEM [6] pointed out a potential concern regarding the lack of accounting for clustering at the school level because children were selected from four elementary schools. However, as outlined in the ***Selection*** domain, the authors stated that they followed the principles of matching social and natural factors like economic situation, educational standards, and geological environments to the extent possible and that the four elementary schools appeared to be very similar in teaching quality. There is no evidence that the sampling strategy was otherwise accounted for (i.e., via sampling weights). The impact of these factors on the effect estimates is expected to be minimal given the use of individual-level data and adjustment for age as a key covariate.

Other potential concerns: None identified.

Basis for rating: Probably low risk of bias based on indirect evidence that the statistical analyses were appropriate and that there were no other potential threats of risk of bias.

Basis for classification as low risk-of-bias study overall: Probably low risk-of-bias ratings in exposure and outcome. Study strengths include individual exposure assessment measurements, but the study is limited by the cross-sectional study design, lack of accounting for urine dilution, and lack of consideration of sex as a key covariate.

Seraj et al. (2012)[7]

Study Details

Study design: Cross-sectional

Population: Children aged 6–11 years

Study area: five villages, Makoo, Iran

Sample size: 293 children

Data relevant to the review: IQ (mean and distribution) assessed by Raven's Colored Progressive Matrices and presented by fluoride area. Adjusted associations between water fluoride and IQ scores.

Reported association with fluoride exposure: Yes: Significant inverse association between water fluoride and IQ score (adjusted $\beta = -3.865$ per 1-mg/L increase; CIs not reported); significantly higher IQ score in normal area (97.77 ± 18.91) compared with medium (89.03 ± 12.99) and high (88.58 ± 16.01) fluoride areas.

Risk of Bias

Author contacts:

Authors were not contacted for additional information because it was not necessary.

Population selection:

Rating: Probably low risk of bias (+)

Summary: Subjects were selected from five villages in Makoo. The villages were stated to all be rural with similar general demographic and geographic characteristics and were comparable in terms of SES and parental occupations. Children were 6–11 years old.

© 2025 Taylor KW et al. *JAMA Pediatrics*.

Age, sex, and education were taken into account in the analysis. No other characteristics were provided or discussed. Participation rates were not reported. There is indirect evidence that the populations were similar, and some possible differences were addressed.

Basis for rating: Probably low risk of bias based on indirect evidence that subjects were similar and recruited using the same methods during the same time frame.

Confounding:

Rating: Probably low risk of bias (+)

Summary: Age, sex, dental fluorosis intensity, and educational levels (child's and parents') were evaluated as important covariates. Other covariates such as smoking were not discussed. Information was obtained from a detailed questionnaire. Lead was measured but found only in low levels in the drinking water throughout the study regions. Iodine in the water was also stated to be measured, and residents were receiving iodine-enriched salt. Arsenic was not addressed, but there is no evidence that arsenic levels would vary across villages in this area. Based on water quality maps, co-exposure to arsenic is likely not a major concern in this area.

Potentially important study-specific covariates: Arsenic.

Direction/magnitude of effect size: Conceptually, if there were differential amounts of arsenic in the different villages, co-exposure to arsenic could bias the association, with the direction of the bias dependent on where the arsenic was present; however, arsenic was not expected to be a major concern in this study area based on water quality maps.

Basis for rating: Probably low risk of bias based on indirect evidence that the methods used to collect the information were valid and that key covariates, including potential co-exposures, were addressed or were not likely to be an issue in the study area.

Attrition:

Rating: Definitely low risk of bias (++)

Summary: Attrition was low if it occurred. It was noted that 293 out of 314 children living in the villages were recruited. It is not clear whether 21 children were excluded based on exclusion criteria or whether they refused to participate; however, this accounts for less than 10% of the population, and results were available for all 293 subjects.

Basis for rating: Definitely low risk of bias based on direct evidence that exclusion of subjects from analyses was minimal, adequately addressed, and reasons were documented when subjects were removed from the study or excluded from analyses.

Exposure:

Rating: Probably high risk of bias (−)

Summary: Exposure was primarily based on area of residence. Fluoride in the groundwater was analyzed by the SPADNS (Sulfophenylazo dihydroxynaphthalene-disulfonate) method, utilizing the 4000 UV-Vis spectrophotometers in the environmental health engineering laboratory of the Public Health School of the Tehran University of Medical Sciences. Specific details were not provided on methods of collection or sample locations or whether these locations represented the primary sources of drinking water for the subjects. Villages were categorized into normal (0.5–1 ppm), moderate (3.1 ± 0.9 ppm), and high (5.2 ± 1.1 ppm) fluoride based on the mean fluoride content of all seasons presumably for the stated 12-year time period. Subjects were stated to be long-life residents of the village. Dental fluorosis was also measured and increased in severity with

© 2025 Taylor KW et al. *JAMA Pediatrics*.

fluoride levels; however, all areas had some degree of dental fluorosis. Although authors used an average fluoride level in varying seasons over presumably 12 years, they used a less-established method without reporting reliability or validity, and they did not provide data to indicate that the mean was truly representative of the fluoride levels over time and throughout the village. Although dental fluorosis severity increased with increasing fluoride levels, the data could also indicate potential exposure misclassification.

Direction/magnitude of effect size: The presence of dental fluorosis in all groups indicates that there may have been different exposures in some children at a younger age. Although there were only about 20 children in the "normal" fluoride group with very mild to mild dental fluorosis, this could bias the results toward the null because those children may have experienced a higher level of fluoride at some point. The other two fluoride groups were exposed to fluoride levels that likely exceeded those in the "normal" fluoride group.

Basis for rating: Probably high risk of bias based on indirect evidence that exposure was assessed using insensitive methods.

Outcome:

Rating: Probably low risk of bias (+)

Summary: Intelligence was evaluated using Raven's Color Progressive Matrices. This is a well-established method. Although the study authors did not provide data to indicate that the methods were valid in this study population, the test is designed to be culturally diverse (+ for methods). The study report stated that test administrators were blinded to subjects' exposure status (++ for blinding). Overall rating for methods and blinding = +.

Basis for rating: Probably low risk of bias based on indirect evidence that outcomes were assessed using instruments that were valid and reliable in the study population, and that the outcome assessors were blind to participants' fluoride exposure.

Selective Reporting:

Rating: Probably low risk of bias (+)

Summary: All outcomes outlined in the abstract, introduction, and methods were reported. However, because the study author did not report the method for obtaining the betas in Table 4 of the study, it is not clear whether these were adjusted or unadjusted regression coefficients.

Basis for rating: Probably low risk of bias based on direct evidence that all the study's measured outcomes were reported, but the results were not sufficiently reported.

Other potential threats:

Rating: Probably low risk of bias (+)

Summary:

Statistical analyses: Statistical methods for comparisons of IQ level by exposure groups were reasonable (ANOVA, post hoc test, and Kruskal-Wallis test), but consideration of heterogeneity of variance was not reported. Clustering at the village levels was not accounted for in multivariate analyses, which used area-level water fluoride levels. Because the exposure levels within a certain area are highly correlated (which might be expected), the results are likely to be biased. There was adjustment for some individual-level important covariates, and the children were from five rural areas with similar general demographic and geographic characteristics and were comparable in terms of SES and parental occupations. These factors are expected to

© 2025 Taylor KW et al. *JAMA Pediatrics*.

mitigate some of the impact of lack of accounting for clustering, and the overall impact on the effect estimates is expected to be minimal.

Other potential concerns: None identified.

Basis for rating: Probably low risk of bias based on indirect evidence that the statistical analyses were appropriate and there were no other potential threats of risk of bias identified.

Basis for classification as low risk-of-bias study overall: Probably low risk-of-bias ratings in confounding and outcome. Study strengths include addressing potential key covariates, but it was limited by the cross-sectional study design and the group-level exposure data.

Trivedi et al. (2012)[8]

Study Details

Study design: Cross-sectional

Population: Children aged 12–13 years

Study area: Kachchh, Gujarat, India

Sample size: 84 children

Data relevant to the review: Mean IQ scores and distribution by low and high fluoride villages.

Reported association with fluoride exposure: Yes: Significantly lower mean IQ score in the high fluoride villages (92.53 ± 3.13) compared with the low-fluoride villages (97.17 ± 2.54) in boys and girls combined (and by sex).

Risk of Bias

Author contacts:

Authors were contacted in September of 2017 to obtain additional information for risk-of-bias evaluation. Additional information provided by the authors informed the rating decision for the following risk-of-bias domains: Selection, Attrition, Detection (exposure assessment), Detection (outcome assessment).

Population selection:

Rating: Probably low risk of bias (+)

Summary: There is insufficient information provided on the sampling methods to determine whether the populations were similar. Although it was noted that samples were obtained for groundwater quality from March to May of 2011, there is no indication that the children were selected at the same time or during a similar time frame. Correspondence with the author indicates that children were selected within a week of the water collection based on random selection of a school in the village. Study participants were selected from six different villages of the Mundra region of Gujarat, India. Subjects were grouped into high and low villages based on the level of fluoride in the drinking water of those villages. The number of subjects per village was not reported, but it was noted that there were 50 children in the low-fluoride group and 34 children in the high fluoride group. It is not clear whether the differences in numbers were based on different participation rates or whether there were fewer children in the high fluoride villages. Recruitment methods, including any exclusion criteria and participation rates, were not provided. SES was stated to be low and equal based on questionnaire information, but the results were not provided. It should also be noted that only regular students (having attendance more than 80%) of standard 6th and 7th grades were selected, but it was not noted whether attendance varied by village. Correspondence with the study author indicated that there

was an average of 20 students per class with an average of 40 students per village. It appears that keeping the requirement of 80% attendance was a limiting factor that resulted in different numbers of children by area; however, this was applied similarly to both groups.

Basis for rating: Probably low risk of bias based on indirect evidence that subjects were similar and recruited using the same methods during the same time frame.

Confounding:

Rating: Probably low risk of bias (+)

Summary: Children were stated to be students of the 6th and 7th standard grades. Age was not addressed, but the children would all be of similar ages based on the grades included. Results were reported for males and females separately as well as combined. SES and iodine consumption were stated to be analyzed via a questionnaire and were standardized on the basis of the 2011 census of India. Although it was noted in the abstract that the SES was equal (no data provided), the study report did not mention the iodine results. Although arsenic and lead were not considered, the study authors provided physicochemical analyses for the water samples from the six different villages. While the authors did not specifically analyze lead or arsenic in the water samples, these physicochemical analyses suggest that differential lead or arsenic exposure was unlikely. Moreover, based on water quality maps, arsenic was not expected to be a major concern in this study area. According to the information from the water quality maps and the physiochemical analysis of the water provided, there is indirect evidence that neither arsenic nor lead were a concern in this study population.

Potentially important study-specific covariates: Key covariates age, sex, and measures of SES were similar between exposure groups; however, arsenic was not considered. Arsenic often occurs in the drinking water along with fluoride in some Indian populations; however, based on water quality maps, arsenic does not appear to be an issue in the study area.

Direction/magnitude of effect size: Conceptually, the presence of arsenic would potentially bias the association away from the null if present with fluoride, or toward the null if present in the reference group; however, for reasons noted above, arsenic is not considered a concern in this study population.

Basis for rating: Probably low risk of bias based on indirect evidence that the methods used to collect the information were valid and reliable, that potential co-exposures were not an issue, and that key covariates were addressed.

Attrition:

Rating: Definitely low risk of bias (++)

Summary: Results were provided for 84 children, but the methods do not indicate how many children were initially selected to participate, nor were any exclusion criteria provided. It was noted in the results that 84 children had their groundwater and urine tested, but it was not noted whether analyses were restricted to these children or whether exposures were assessed in all the children who had IQ measurements. Correspondence with the study author indicated that the main reason for exclusion was a <80% attendance rate, with fluoride and IQ measured on all 84 children who met the criteria.

Basis for rating: Definitely low risk of bias based on direct evidence of no attrition.

Exposure:

Rating: Probably low risk of bias (+)

Summary: Children in villages were grouped based on fluoride levels that were assessed in groundwater (low fluoride villages versus high fluoride villages). The average concentration of these levels was considered to be the levels in the drinking water with confirmation using urinary fluoride levels. The groundwater samples were selected to cover major parts of the taluka and represent overall groundwater quality. Ten samples were obtained from each village. Fluoride was measured in the groundwater using ion exchange chromatography. Although urine levels were also significantly higher in the high fluoride village, no information was provided on how or when the urinary samples were obtained or how they were measured. However, correspondence with the study author indicated that the groundwater and urine fluoride levels were available for all 84 children, indicating that the urine measures were available for the children that had IQ measures. The urine samples were stated to be collected at the same time the second water sample was collected.

Direction/magnitude of effect size: Fluoride levels were measured in both the drinking water and urine. Although there is some variability in the measurements, there is no overlap between the two groups, and the urine and drinking water levels in the children support each other. Any potential exposure misclassification would be non-differential, and the impact on the direction and magnitude of the effect size is unknown.

Basis for rating: Probably low risk of bias based on indirect evidence that exposure was consistently assessed using well-established methods that directly measured exposure.

Outcome:

Rating: Probably low risk of bias (+)

Summary: Outcome methods were only noted to be reported in Trivedi et al. (2007)[9], which was scored as follows: IQ was measured in the children of both areas using a questionnaire prepared by Professor JH Shah, copyrighted by Akash Manomapan Kendra, Ahmedabad, India, and standardized on the Gujarati population with a 97% reliability rate in relation to the Stanford-Binet Intelligence Scale (+ for methods). Blinding or other methods to reduce bias were not reported, but correspondence with the study author indicated that the teachers were blind to the status of fluoride. The teachers administered the tests in the presence of a research fellow. It is not completely clear who scored the tests, but it is assumed the teachers (+ for blinding). Overall rating for methods and blinding = +.

Basis for rating: Probably low risk of bias based on indirect evidence that the outcomes were assessed using instruments that were valid and reliable in the study population, and that the outcome assessors were blind to participants' fluoride exposure.

Selective Reporting:

Rating: Definitely low risk of bias (++)

Summary: All outcomes outlined in the abstract, introduction, and methods are reported in sufficient detail.

Basis for rating: Definitely low risk of bias based on direct evidence that all measured outcomes were reported.

Other potential threats:

Rating: Probably high risk of bias (−)

Summary:

Statistical analyses: Mean IQ scores in low and high fluoride villages were compared using a t-test. Consideration of heterogeneity of variances was not reported. Results are reported as means and standard errors of the means, with p-values for significant differences. Area-level exposures were used. There was no accounting for clustering of children within the villages, and comparative analyses did not account for covariates. Urinary fluoride was not considered in the comparative analyses. The lack of individual exposure levels and the lack of accounting for clustering are likely to bias the standard error of the difference in mean IQ levels between the high- and low-fluoride villages and make the differences appear stronger than they actually are.

Basis for rating: Probably high risk of bias based on indirect evidence that the statistical analyses did not account for clustering, and this lack of accounting could bias the association. There were no other potential threats of risk of bias identified.

Basis for classification as low risk-of-bias study overall: Probably low risk-of-bias ratings in confounding, exposure, and outcome. Study strengths include individual exposure assessment measurements and the addressing of potential key covariates, but the study was limited by the cross-sectional study design. Another limitation was the lack of accounting for clustering, which may bias the standard error of the differences, making the effect appear stronger than it actually is; however, this does not change the nearly 5-point difference in IQ scores between the two villages.

Zhang et al. (2015)[10]

Study Details

Study design: Cross-sectional

Population: Children aged 10–12 years

Study area: Tianjin City, China

Sample size: 180 children

Data relevant to the review: Mean IQ scores by control and high fluoride groups; correlations between water, serum, or urinary fluoride and IQ scores; adjusted associations between urinary fluoride and IQ scores (by genotypes).

Reported association with fluoride exposure: Yes: Significant correlation between children's serum fluoride (r = −0.47) and urinary fluoride (r = −0.45) and IQ scores; significant difference in mean IQ score for high-fluoride area (defined as >1 mg/L in drinking water; 102.33 ± 13.46) compared with control area (<1 mg/L; 109.42 ± 13.30).

Risk of Bias

Author contacts:

Authors were not contacted for additional information because it was not necessary.

Population selection:

Rating: Definitely low risk of bias (++)

Summary: Subjects were similar and recruited during the same time frame using the same methods. Authors recruited schoolchildren from a high fluoride area (1.40 mg/L) and a control area (0.63 mg/L) in Tianjin City, China. In accordance with the principles of matching social and natural factors such as educational standard, economic situation, and geological environments as much as possible, two areas with different fluoride concentrations in the groundwater were selected by a stratified cluster random sampling

© 2025 Taylor KW et al. *JAMA Pediatrics*.

of this region. A total of 180 5th grade children aged 10 to 12 years from two primary schools located 18 km apart in the Jinnan District were recruited—Gegu Second Primary School (from an endemic fluorosis area) and Shuanggang Experimental Primary School (from a non-endemic fluorosis area). The areas are not affected by other drinking water contaminants, such as arsenic or iodine. All subjects were unrelated ethnic Han Chinese and residents in Tianjin with similar physical and mental health status. The authors excluded subjects with known neurological conditions, including pervasive developmental disorders and epilepsy. Descriptive statistics of the study population are presented by exposure group in Table 1 of the study. A number of potential differences were considered in the statistical analyses.

Basis for rating: Definitely low risk of bias based on direct evidence that the exposure groups were similar and recruited using similar methods during the same time frame.

Confounding:

Rating: Probably low risk of bias (+)

Summary: Covariates included in the statistical models were age, sex, educational levels of parents, drinking water fluoride (mg/L), and levels of thyroid hormones (T3, T4, and TSH). Authors report that the study areas were not affected by other contaminants such as arsenic or iodine, and residents were of similar physical and mental health status. Other important covariates (maternal demographics, smoking, reproductive health) were not considered. Covariate data were obtained from a study questionnaire.

Potentially important study-specific covariates: All key covariates were considered in this study.

Direction/magnitude of effect size: Not applicable.

Basis for rating: Probably low risk of bias based on indirect evidence that the methods used to collect the information were valid and reliable and direct evidence that key covariates, including potential co-exposures, were considered.

Attrition:

Rating: Definitely low risk of bias (++)

Summary: Results are complete for the 180 children selected for the study.

Basis for rating: Definitely low risk of bias based on direct evidence that there was no attrition.

Exposure:

Rating: Definitely low risk of bias (++)

Summary: Drinking water samples (10 mL) were collected from the tube wells of each child’s household. Three fasting venous blood samples were also collected. Urine samples were collected in the early morning before breakfast. Fluoride content in drinking water (W-F), serum (S-F), and urine (U-F) was measured using an ion analyzer EA940 with a fluoride ion-selective electrode (Shanghai Constant Magnetic Electronic Technology Co, Ltd, China), according to the China standard GB 7484-87. All reference solutions for the fluoride determinations were double-deionized water. Parallel samples were set for determination, and averages were taken. The quantitation limits of this method for W-F, S-F, and U-F were 0.2, 0.012, and 0.5 mg/L, respectively. Recovery rates for this method were in the range of 94.3%–106.4%. The intra- and inter-assay coefficients of variation for fluoride were 2.7% and 6.7%, respectively. Dilution of the urinary fluoride was not addressed.

Direction/magnitude of effect size: Not applicable.

© 2025 Taylor KW et al. *JAMA Pediatrics*.

Basis for rating: Definitely low risk of bias based on direct evidence that the exposure was consistently assessed using well-established methods that directly measured exposure.

Outcome:

Rating: Definitely low risk of bias (++)

Summary: A Combined Raven's Test for Rural China (CRT-RC) was taken to evaluate the IQ of each child (++ for methods). The study report stated that all tests were administered at school by a trained examiner who was masked to participants' drinking water fluoride levels (++ for blinding). Overall rating for methods and blinding = ++.

Basis for rating: Definitely low risk of bias based on direct evidence that the outcome was assessed using instruments that were valid and reliable in the study population, and that the outcome assessor was blind to participants' fluoride exposure.

Selective Reporting:

Rating: Definitely low risk of bias (++)

Summary: All results outlined in the abstract, introduction, and methods sections were reported in sufficient detail.

Basis for rating: Definitely low risk of bias based on direct evidence that all measured outcomes were reported.

Other potential threats:

Rating: Probably low risk of bias (+)

Summary:

Statistical analyses: Associations between serum and urinary fluoride levels and IQ score were estimated using general linear models and multivariate linear regression by COMT polymorphism. Normality (Kolmogorov-Smirnov test) was evaluated for all continuous variables. There is no evidence that residual diagnostics were used to examine model assumptions or that the complex sampling design (stratified multistage random sampling) was accounted for in the analysis using sampling weights and adjustment for clustering. The impact of these factors on the regression effect estimates is expected to be minimal given the use of individual-level data and adjustment for numerous covariates.

Other potential concerns: None identified.

Basis for rating: Probably low risk of bias based on direct evidence that the statistical analyses were appropriate and there were no other potential threats of risk of bias identified.

Basis for classification as low risk-of-bias study overall: Definitely or probably low risk-of-bias ratings in confounding, exposure, and outcome. Study strengths include individual exposure assessment measurements, blinding of outcome assessor to participants' fluoride exposure, and consideration of key covariates including potential co-exposures.

Bashash et al. (2017)[11]

Study Details

Study design: Prospective cohort

Population: Early Life Exposures in Mexico to Environmental Toxicants (ELEMENT) participants (pregnant mothers and their children aged 4 or 6–12 years)

Study area: Mexico City, Mexico

Sample size: 299 mother-child pairs, of whom 211 had data for the IQ analyses and 287 had data for the general cognitive index (GCI).

Data relevant to the review: Adjusted and unadjusted associations between maternal or child's urinary fluoride concentrations and IQ scores.

Reported association with fluoride exposure: Yes: Significant inverse association between maternal urinary fluoride and IQ score (adjusted β = −2.50 per 0.5-mg/L increase; 95% CI: −4.12, −0.59). Significant inverse association between maternal urinary fluoride and GCI score (adjusted β = −3.15 per 0.5-mg/L increase; 95% CI: −5.42, −0.87). No significant associations with children's urinary fluoride.

Risk of Bias

Author contacts:

Authors were contacted for additional information on whether clustering was addressed. The authors provided results from additional models with cohort as a random effect, which informed the rating decision for the following risk-of-bias domains: Other.

Population selection:

Rating: Probably low risk of bias (+)

Summary: Study participants were selected from two different cohorts from three hospitals in Mexico City that serve low-to-moderate income populations. One cohort was from an observational study of prenatal lead exposure and neurodevelopment outcomes, and the other was from a randomized trial of the effect of calcium on maternal blood lead levels. The authors state that participants had no history of psychiatric disorders, high-risk pregnancies, gestational diabetes, illegal drug use, or continuous prescription drugs, but no information on smoking habits was considered. Study populations appear to be similar, but there may be some differences because subjects were selected from two different cohorts that were recruited from slightly different time periods.

Basis for rating: Probably low risk of bias based on indirect evidence that the exposure groups were similar despite the subjects coming from different original study populations wherein different methods were used for recruitment.

Confounding:

Rating: Probably low risk of bias (+)

Summary: Data were collected via questionnaire on maternal age, education, marital status at first prenatal visit, birth order, birth weight, gestational age at delivery, maternal smoking, maternal IQ, and HOME scores. All models were adjusted for gestational age at birth, sex, birth weight, birth order, age at testing, maternal marital status, smoking history, age at delivery, maternal IQ, education, and cohort, with additional testing for children's urinary fluoride, mercury, lead, and calcium. Sensitivity analyses were additionally adjusted for HOME score. Important covariates not considered included BMI, iodine deficiency, arsenic, and maternal mental health and nutrition. Arsenic is assumed not to be a potential co-exposure in this population because the study authors did not discuss it as an issue, although other co-exposures were considered. Arsenic is included in the water quality control program in Mexico City and thus is not considered a concern in this population.

Potentially important study-specific covariates: All key covariates were addressed.

Direction/magnitude of effect size: Not applicable.

Basis for rating: Probably low risk of bias based on direct evidence that key covariates, including other potential co-exposures, were addressed, indirect evidence that the

© 2025 Taylor KW et al. *JAMA Pediatrics*.

methods used to collect the information were valid and reliable and that arsenic is not likely to be an issue in this study population.

Attrition:

Rating: Probably low risk of bias (+)

Summary: Although there was a large amount of attrition, the study authors clearly describe all reasons for attrition and also provide characteristics to compare those participants included to those excluded. There were some slight differences between those included and those excluded, but there is no evidence to indicate that attrition would potentially bias the results.

Basis for rating: Probably low risk of bias based on indirect evidence that exclusion of subjects from analyses was adequately addressed, and reasons were documented when subjects were removed from the study or excluded from analyses.

Exposure:

Rating: Definitely low risk of bias (++)

Summary: Urinary fluoride concentrations were determined in spot urine samples (second morning void) collected from mothers (during at least one trimester) and children ages 6–12 years. Fluoride content was measured using ion-selective electrode-based assays. Quality control methods were described including between laboratory correlations. All samples were measured in duplicate. Extreme outliers were excluded. Urinary dilution was addressed by using creatinine-adjusted levels.

Direction/magnitude of effect size: Not applicable.

Basis for rating: Definitely low risk of bias based on direct evidence that exposure was consistently assessed using well-established methods that directly measured exposure.

Outcome:

Rating: Definitely low risk of bias (++)

Summary: Outcome was assessed using the McCarthy Scales of Children's Abilities (MSCA) in 4-year-old children (translated into Spanish) and the Wechsler Abbreviated Scale of Intelligence (WASI) in 6–12-year-olds. The WASI is a well-established test, and the validity of both tests is well documented by the authors. Inter-examiner reliability was evaluated and reported with a correlation of 0.99 (++ for methods). The study report stated that psychologists were blind to the children's fluoride exposure (++ for blinding). Overall rating for methods and blinding = ++.

Basis for rating: Definitely low risk of bias based on direct evidence that the outcome was assessed using instruments that were valid and reliable in the study population, and that the outcome assessor was blind to participants' fluoride exposure.

Selective Reporting:

Rating: Definitely low risk of bias (++)

Summary: All outcomes outlined in the abstract, introduction, and methods are reported in sufficient detail.

Basis for rating: Definitely low risk of bias based on direct evidence that all measured outcomes were reported.

Other potential threats:

Rating: Definitely low risk of bias (++)

© 2025 Taylor KW et al. *JAMA Pediatrics*.

Summary:

Statistical analyses: Statistical analyses used were appropriate for the study. Statistical tests of bivariate associations (using Chi-square tests for categorical variables and analysis of variance [ANOVA]) were used to compare the means of the outcomes or exposure within groups based on the distribution of each covariate. Generalized additive models (GAMs) were used to estimate the adjusted association between fluoride exposure and measures of children's intelligence. Residual diagnostics were used to examine model assumptions and identify any potentially influential observations. Results are reported as adjusted regression slopes and 95% CIs. In sensitivity analyses, regression models accounted for clustering at the cohort level by using cohort as a fixed effect in the models. Although using cohort as a random effect would be more appropriate, using individual-level exposure data and accounting for numerous important covariates in the models likely captured the cohort effect. Additional models with cohort as a random effect were also subsequently made available via personal communication with the study authors and showed similar results to the main model.

Other potential concerns: None identified.

Basis for rating: Definitely low risk if bias is based on direct evidence that the statistical analyses were appropriate and there were no other potential threats of risk of bias identified.

Basis for classification as low risk-of-bias study overall: Definitely or probably low risk-of-bias ratings in confounding, exposure, and outcome. Study strengths include individual exposure assessment measurements, blinding of outcome assessor to participants' fluoride exposure, and the prospective cohort study design.

Valdez Jimenez et al. (2017)[12]

Study Details

Study design: Prospective cohort

Population: Infants aged 3–15 months

Study area: Durango City and Lagos de Moreno, Jalisco, Mexico

Sample size: 65 infants

Data relevant to the review: The Bayley Scales of Infant Development II was used to assess Mental Development Index scale and the Psychomotor Development Index scale in children aged 3 to 15 months and evaluated for associations with first and second trimester maternal urine fluoride.

Reported association with fluoride exposure: Yes: Significant inverse association between maternal urinary fluoride and MDI score during first trimester (adjusted $\beta = -19.05$ per log10-mg/L increase; SE = 8.9) and second trimester (adjusted $\beta = -19.34$ per log10-mg/L increase; SE = 7.46). No association between maternal fluoride during any trimester and Psychomotor Developmental Index (PDI).

Risk of Bias

Author contacts:

Authors were not contacted for additional information because it was not necessary.

Population selection:

Rating: Probably low risk of bias (+)

Summary: Subjects were recruited from two endemic areas in Mexico. The study authors do not provide information on the similarities or differences between the two areas, nor do they indicate whether there were different participation rates. However, recruitment methods were the same. Women receiving prenatal care in health centers located in Durango City and Lagos de Moreno, Jalisco, Mexico were recruited in 2013–2014. Participation rates are not likely to be an issue as characteristics were similar between those who participated and those who did not. Although the authors did not provide characteristics by area, the characteristics provided do not indicate any differences that may be biased by the selection. Considering the age range for the non-participants, the mean age for non-participants appears to be incorrect (or the age range is incorrect); however, there does not appear to be a difference that would potentially indicate selection bias.

Basis for rating: Probably low risk of bias based on indirect evidence that the exposure groups were similar and were recruited with the same methods in the same time frame, with no evidence of differences or issues with participation/response rates.

Confounding:

Rating: Probably high risk of bias (−)

Summary: Questionnaires were used to obtain information about sociodemographic factors, prenatal history, mother's health status before pregnancy (e.g., use of drugs, vaccines, diseases), and the type of water for drinking and cooking. The marginalization index (MI) was obtained from the National Population Council (CONAPO). Two additional surveys were conducted during the second and third trimester of pregnancy to get information about the mother's health, pregnancy evolution, and sources of water consumption. A survey was also conducted to get information about childbirth (type of birth, week of birth, weight and length of the baby at birth, Apgar score and health conditions of the baby during the first month of life). This information was corroborated with the birth certificate. Linear regression models included gestational age, children's age, marginality index, and type of drinking water. Bivariate analyses were conducted on the other factors, including sex, prior to conducting multivariable regression models. Some important covariates were not considered, including parental mental health, IQ, smoking, and potential co-exposures. Water quality maps indicate a potential for arsenic to be present in the study area.

Potentially important study-specific covariates: Arsenic is a potential co-exposure in this area of Mexico.

Direction/magnitude of effect size: If arsenic were present as a co-exposure, it would likely bias the association away from the null.

Basis for rating: Probably high risk of bias based on indirect evidence that there is a potential for co-exposure with arsenic that was not addressed.

Attrition:

Rating: Definitely low risk of bias (++)

Summary: Out of the 90 women selected for inclusion in the study, 65 approved the participation of their infants. The authors provide a table of characteristics between women who consented to their children's cognitive evaluation and those who participated only in biological monitoring. There were no significant differences between the groups. There were fewer women who provided urine during the second and third trimesters. All specified children are included in the relevant analyses.

© 2025 Taylor KW et al. *JAMA Pediatrics*.

Basis for rating: Definitely low risk of bias based on direct evidence that exclusion of subjects from analyses was adequately addressed, and reasons were documented when subjects were removed from the study or excluded from analyses.

Exposure:

Rating: Definitely low risk of bias (++)

Summary: Fluoride exposure was assessed through morning urine samples and water fluoride levels collected from the children's homes. Sampling methodology was appropriately documented, and water levels were quantified through specific ion-sensitive electrode assays. QC was described, and accuracy was >90%. Urinary fluoride was corrected by specific gravity.

Direction/magnitude of effect size: Not applicable.

Basis for rating: Definitely low risk of bias based on direct evidence that exposure was consistently assessed using well-established methods that directly measured exposure.

Outcome:

Rating: Definitely low risk of bias (++)

Summary: Neurodevelopment was assessed with the Bayley Scales of Infant Development II (BSDI-II) that was noted to be reliable and valid for evaluating children from 3 months to 5 years of age. The average age of children assessed was 8 months, with a range of 3–15 months) (++ for methods). The study report stated that a trained psychologist who was blinded to the mother's fluoride exposure evaluated the infants at home (++ for blinding). Overall rating for methods and blinding = ++.

Basis for rating: Definitely low risk of bias based on direct evidence that the outcome was assessed using instruments that were valid and reliable in the study population, and that the outcome assessor was blind to participants' fluoride exposure.

Selective Reporting:

Rating: Probably low risk of bias (+)

Summary: All outcomes outlined in the abstract, introduction, and methods were reported. Table 4 of the study displays only data for trimesters 1 and 2. Although third trimester data were collected, they were not reported, likely because they were available for only 29 subjects. No discussion of this was provided.

Basis for rating: Probably low risk of bias because, although it appears some data were not reported, it is likely because there were insufficient data and not because the authors were selectively reporting the results.

Other potential threats:

Rating: Probably low risk of bias (+)

Summary:

Statistical analyses: Statistical analyses used log10-transformed exposure variables. Normality, homoscedasticity, and linearity assumptions were tested and satisfied for MDI and PDI scores. Bivariate analyses included correlations, t-tests, and ANOVA. Multiple linear regression models by the first and second trimester of pregnancy were used to evaluate the association between maternal fluoride exposure and MDI and PDI scores. The best-fit model was selected using a "stepwise method," and the best-fit line was evaluated using "the curve fitting method." It is not further specified or cited what these methods entailed. Best-fit or goodness-of-fit statistics are not reported. It is unclear how a best-fit model could be selected when the authors state

that all models adjusted for the same set of covariates regardless of significance, and these covariates also appear in the final model—presumably the best-fit model. It is unlikely that a stepwise method would retain all those covariates unless they were forced in the model. Residual analysis was conducted to assess model validity; however, there is no description of the results of the residual analysis. Nonetheless, the impact on effect estimates is expected to be minimal.

Other potential concerns: No other potential concerns were identified. In the peer-review report, NASEM [4] cited the following as potential concerns: "the large difference in numbers of males and females in the offspring (20 males, 45 females), and apparently incorrect probabilities were reported for age differences between participants and nonparticipants, high rates of cesarean deliveries and premature births among participants (degree of overlap not reported), and incorrect comparisons of observed prematurity rates with national expected rates." However, these concerns were taken into consideration in other domains (***Selection, Confounding***).

Basis for rating: Probably low risk of bias based on indirect evidence that the statistical analyses were appropriate and there were no other potential threats of risk of bias identified.

Basis for classification as low risk-of-bias study overall: Definitely low risk-of-bias ratings in exposure and outcome. Study strengths include individual exposure assessment measurements and blinding of outcome assessor to participants' fluoride exposure, but it is limited by the cross-sectional study design and lack of accounting for potential co-exposures to arsenic.

Cui et al. (2018)[13]

Study Details

Study design: Cross-sectional

Population: School children aged 7–12 years from four schools in two districts in China with different fluoride levels

Study area: Jinghai and Dagang in Tianjin City, China

Sample size: 323 school children

Data relevant to the review: Adjusted associations between urinary fluoride and IQ scores.

Reported association with fluoride exposure: Yes: Significant inverse association between urinary fluoride and IQ score (adjusted $\beta = -2.47$ per 1-log mg/L increase; 95% CI: −4.93, −0.01).

Risk of Bias

Author contacts:

Authors were contacted in June 2019 to obtain additional information for risk-of-bias evaluation. Additional information provided by the authors informed the rating decision for the following risk-of-bias domains: Detection (outcome assessment).

Population selection:

Rating: Probably low risk of bias (+)

Summary: Four schools were selected from the same district in China. The schools were selected based on levels of fluoride in the local drinking water and the degree of school cooperation. No details were provided on the number of schools in given areas or the difficulty in getting school cooperation. It was noted that the residents in the four areas

© 2025 Taylor KW et al. *JAMA Pediatrics*.

had similar living habits, economic situations, and educational standards. Although authors do not provide the specific data to support this, fluoride levels and IQ scores were provided by different subject characteristics. The areas were classified as historically endemic fluorosis and non-fluorosis. Cluster sampling was used to select the grades in each school according to previously set child ages, and classroom was randomly selected with all students within a selected classroom included. Reasons for exclusion do not appear to be related to exposure or outcome.

Basis for rating: Probably low risk of bias based on indirect evidence that the exposure groups were similar and recruited within the same time frame using the same methods, with no evidence of differences in participation/response rates.

Confounding:

Rating: Probably low risk of bias (+)

Summary: The measurements of all covariates were obtained by structured questionnaires that were completed by children with the help of their parents. Covariates that were assessed include: sex, age, child's ethnicity, child's BMI, birth (normal vs. abnormal), mother's age at delivery, mother's education, income per family member, mother's smoking/alcohol during pregnancy, family member smoking, environmental noise, iodine region (non-endemic vs. iodine-excess-endemic area), factory within 30 m of residence, iodine salt, diet supplements, seafood/pickled food/tea consumption, surface water consumption, physical activity, stress, anger, anxiety/depression, trauma, having a cold 5 times a year, thyroid disease in relatives, mental retardation in relatives, and cancer in relatives. Covariates not considered include parity, maternal and paternal IQ, and quantity and quality of caregiving environment (e.g., HOME score). The authors report that there were no other environmentally toxic substances that might have affected intelligence, such as high arsenic or iodine deficiency according to the Tianjin Centers for Disease Prevention and Control.

Potentially important study-specific covariates: All key covariates were considered in this study.

Direction/magnitude of effect size: Not applicable.

Basis for rating: Probably low risk of bias because there is indirect evidence that the key covariates were considered, methods for collecting the information were valid and reliable, and co-exposure to arsenic was likely not an issue in this area.

Attrition:

Rating: Probably low risk of bias (+)

Summary: Of the 400 children enrolled, 35 were excluded because they did not have informed consent signed by a guardian or they moved out of the area. Forty-two children were excluded because they did not have a DRD2 genotyping measurement. It is unclear whether these children were from the same schools or whether they were evenly distributed throughout the study area. It is also unclear whether the excluded subjects were similar to those included in the study. In the study, some analyses had fewer than 323 subjects, but this seems reasonable given the subgroups that were being evaluated.

Basis for rating: Probably low risk of bias based on indirect evidence that exclusion of subjects from analyses was adequately addressed, and reasons were documented when subjects were removed from the study or excluded from analyses.

Exposure:

Rating: Probably low risk of bias (+)

© 2025 Taylor KW et al. *JAMA Pediatrics*.

Summary: Although children were selected based on area fluoride levels, fluoride in the urine was used in the analysis. Urine was collected from each child during the morning of enrollment and analyzed within a week. Fluoride levels were measured using an ion-selective electrode according to the China standard. A brief description of the method was provided, but no QC methods were reported. The study authors did not account for urinary dilution in the spot samples.

Direction/magnitude of effect size: Not accounting for dilution could cause some exposure misclassification. The direction and magnitude would depend on where the differences occurred.

Basis for rating: Probably low risk of bias based on indirect evidence that exposure was consistently assessed using acceptable methods that provide individual levels of exposure.

Outcome:

Rating: Probably low risk of bias (+)

Summary: IQ was measured by professionals using the Combined Raven's Test–The Rural in China method, which is the appropriate test for the study population (++ for methods). Blinding or other methods to reduce bias were not reported. Although it was unlikely that the outcome assessor would have knowledge of the child's urine fluoride levels, there was potential that they would know whether the child was from an endemic or non-endemic area if the IQ tests were conducted at the child's school, and there was no information provided on how the IQ tests were administered. Correspondence with the study author noted the cross-sectional nature of the study with outcome and exposure assessed at the same time, making the outcome assessors blind to the exposure status of participants. However, there was still potential for knowledge of the area (+ for blinding).

Basis for rating: Probably low risk of bias based on indirect evidence that the outcome was assessed using instruments that were valid and reliable in the study population, and that the outcome assessors were blind to participants' fluoride exposure.

Selective Reporting:

Rating: Definitely low risk of bias (++)

Summary: All outcomes in the abstract, introduction, and methods are reported in sufficient detail.

Basis for rating: Definitely low risk of bias based on direct evidence that all measured outcomes were reported.

Other potential threats:

Rating: Probably low risk of bias (+)

Summary:

Statistical analyses: Statistical analyses were appropriate. Multiple linear regression models were applied to evaluate the relationship between urine fluoride levels and IQ scores, accounting for numerous important covariates. The urinary fluoride levels were log-transformed due to a skewed distribution. Residual diagnostics were used to examine model assumptions. Model robustness was tested through bootstrap, sensitivity analysis after excluding potential outliers, and cross-validation techniques. Results are reported as adjusted regression slopes and 95% CIs. The analysis did not account for clustering at the school level or at the grade level (students were from four schools in grades selected via a clustered sampling method). There is no evidence that the sampling strategy was otherwise accounted for via sampling

© 2025 Taylor KW et al. *JAMA Pediatrics*.

weights. The impact of these factors on the effect estimates is expected to be minimal given the use of individual-level data and adjustment for several important covariates.

Other potential concerns: None identified.

<u>Basis for rating</u>: Probably low risk of bias based on indirect evidence that the statistical analyses were appropriate, and there were no other potential threats of risk of bias identified.

Basis for classification as low risk-of-bias study overall: Probably low risk-of-bias ratings in confounding, exposure, and outcome. Study strengths include individual exposure assessment measurements but is limited by the cross-sectional study design and lack of accounting for urine dilution. All key covariates were considered in the study design or analysis.

Yu et al. (2018)[14]

Study Details

Study design: Cross-sectional

Population: Children aged 7–13 years

Study area: Tianjin City, China

Sample size: 2,886 school children

Data relevant to the review: IQ for normal (≤1 mg/L) versus high (>1 mg/L) water fluoride; adjusted associations between water and urinary fluoride and IQ scores and IQ groupings.

Reported association with fluoride exposure: Yes: Significant difference in mean IQ scores in high water fluoride areas (>1.0 mg/L; 106.4 ± 12.3 IQ) compared to the normal water fluoride areas (≤1.0 mg/L; 107.4 ± 13.0). Distribution of IQ scores was also significantly different ($p = 0.003$). Significant inverse association between water fluoride and IQ scores (adjusted $\beta = -4.29$ per 0.5-mg/L increase; 95% CI: −8.09, −0.48).

Risk of Bias

Author contacts:

Authors were contacted in September 2018 to obtain additional information for the risk-of-bias evaluation. Additional information provided by the authors informed the rating decision for the following risk-of-bias domains: Detection (outcome assessment).

Population selection:

<u>Rating</u>: Definitely low risk of bias (++)

<u>Summary</u>: School children (2,886), aged 7–13 years, were recruited from the rural areas of Tianjin City, China. After exclusion, 1,636 children were assigned to the "normal-fluoride" exposure group, and 1,250 were assigned to the "high-fluoride" exposure group based on a cut-off water fluoride level of 1.0 mg/L. A multistage random sampling technique, stratified by area, was performed to select representative samples among local children who were permanent residents since birth. Detailed characteristics of the study population were provided. Exclusion criteria included: 1) children who had congenital or acquired diseases affecting intelligence, 2) children with a history of cerebral trauma and neurological disorders, 3) children with a positive screening test history (like hepatitis B virus infection, Treponema palladium infection and Down's syndrome), and 4) children with adverse exposures (smoking and drinking) during maternal pregnancy. A table of characteristics was provided by fluoride level with differences adjusted in the analysis.

© 2025 Taylor KW et al. *JAMA Pediatrics*.

Basis for rating: Definitely low risk of bias based on direct evidence that the exposed groups were recruited using similar methods during the same time frame and that any differences between the exposed groups were considered in the statistical analyses.

Confounding:

Rating: Probably low risk of bias (+)

Summary: Demographic data were collected by trained investigators during a face-to-face interview with the recruited children and their parents. Questionnaires were not stated to be validated. The developmental status of the children was further assessed by calculation of BMI, and all measurements were conducted by nurses based on recommended standard methods. Variables that presented differential distribution between the normal-fluoride and high-fluoride exposure groups were adjusted in the linear regression analysis of IQ data and included age, sex, paternal and maternal education levels, and low birth weight. Children exposed to smoking in utero were excluded from the study. Sensitivity analyses were conducted by modifying covariates adjusted in multivariable models among demographics (age and sex); development (BMI); socioeconomics (maternal education, paternal education, and household income); history of maternal disease during pregnancy (gestational diabetes, malnutrition, and anemia); and delivery conditions (hypoxia, dystocia, premature birth, post-term birth, and low birth weight). None of the study sites selected were in areas endemic for iodine deficiency disorders, nor were other potential neurotoxins like lead, arsenic, and mercury present. Variables such as parental BMI and behavioral and mental health disorders were not addressed.

Potentially important study-specific covariates: All key covariates were considered in this study.

Direction/magnitude of effect size: Not applicable.

Basis for rating: Probably low risk of bias based on indirect evidence that methods of obtaining the information were valid and reliable and direct evidence that all key covariates and co-exposures were considered.

Attrition:

Rating: Probably low risk of bias (+)

Summary: There were 1,636 children assigned to the "normal-fluoride" exposure group based on water fluoride and 1,250 children assigned to the "high-fluoride" exposure group. Exclusion from the original group of 2,886 children was adequately described. A total of 2,380 children provided urine samples. There is no indication that the data presented excludes any additional children or urine samples, but results do not indicate a sample size for all results.

Basis for rating: Probably low risk of bias based on indirect evidence that exclusion of subjects from analyses was adequately addressed, and reasons were documented when subjects were removed from the study or excluded from analyses.

Exposure:

Rating: Probably low risk of bias (+)

Summary: According to the annual surveillance data from the CDC, the drinking water sources and water fluoride concentrations in each village had remained at stable levels over the past decade. During the investigation, water samples were collected randomly from the public water supplies in each village. Spot (early-morning) urine samples from every child and water samples from each village were collected in pre-cleaned, labeled polythene tubes and transported to the lab within 24 hours while frozen. Samples were stored at −80°C until analysis. Concentrations of fluoride ions (mg/L) were analyzed

using the national standardized ion-selective electrode method in China; the detection limit was 0.01 mg/L. Samples were diluted with an equal volume of total ionic strength adjusted buffer (TISAB) of pH 5–5.5 for optimal analysis. Double-distilled deionized water was used throughout the experiment. There is no reporting of any QC methods.

Direction/magnitude of effect size: Spot urine samples may lead to non-differential exposure misclassification. The large population size likely dilutes any potential effects of occasional misclassification. Because the drinking water sources of fluoride had been noted to be stable for the past decade and the children were 13 years or younger, there would only be exposure misclassification if there was a lot of migration between areas.

Basis for rating: Probably low risk of bias based on indirect evidence that exposure was consistently assessed using well-established methods that directly measured exposure.

Outcome:

Rating: Definitely low risk of bias (++)

Summary: IQ scores were measured using the second edition of the Combined Raven's Test–The Rural in China (CRT-RC2) for children aged 7–13 years (++ for methods). The test was completed by each participant within 40 minutes, according to the instruction manual. For each test, 40 children were randomly allocated to one classroom to take the test independently under the supervision of four trained professionals. There is no mention of whether the evaluators were blinded to the fluoride group of each child (normal vs. high fluoride) or whether there were steps taken to ensure consistency in scoring across the evaluators. It is also not clear whether the 40 children randomly assigned to the classroom were specific to the village or whether a local center was used. Correspondence with the study authors indicated that the four professionals worked together throughout the examination without knowledge of the child's fluoride exposure (++ for blinding).

Basis for rating: Definitely low risk of bias based on the direct evidence that the outcome was assessed using instruments that were valid and reliable, and that the outcome assessors were blind to participants' fluoride exposure.

Selective Reporting:

Rating: Definitely low risk of bias (++)

Summary: All outcomes outlined in the abstract, introduction, and methods are reported in sufficient detail.

Basis for rating: Definitely low risk of bias based on direct evidence that all measured outcomes were reported.

Other potential threats:

Rating: Probably low risk of bias (+)

Summary:

Statistical analyses: Statistical analyses used were appropriate for the study. Univariate and multivariable piecewise linear regression models were used to estimate the associations between water fluoride or urinary fluoride levels and IQ scores. Multiple logistic regression analysis was used to evaluate the association between water or urinary fluoride levels and IQ degree using the normal intelligence group as the control. Sensitivity analyses were conducted. There is no evidence that residual diagnostics were used to examine model assumptions or that the complex sampling design (stratified multistage random sampling) was accounted for in the analysis

© 2025 Taylor KW et al. *JAMA Pediatrics*.

using sampling weights and adjustment for clustering. The impact of these factors on the effect estimates is expected to be minimal given the use of individual-level data and adjustment for numerous important covariates.

Other potential concerns: None identified.

Basis for rating: Probably low risk of bias based on indirect evidence that the statistical analyses were appropriate and there were no other potential threats of risk of bias identified.

Basis for classification as low risk-of-bias study overall: Definitely or probably low risk-of-bias ratings in confounding, exposure, and outcome. Study strengths include individual exposure assessment measurements with blinding at outcome assessment but is limited by the cross-sectional study design and lack of accounting for urine dilution. All key covariates, including potential co-exposures, were considered in the study design or analysis.

Green et al. (2019)[15]

Study Details

Study design: Prospective cohort

Population: Maternal-Infant Research on Environmental Chemicals (MIREC) participants (pregnant mothers and their children aged 3–4 years)

Study area: 10 cities, Canada

Sample size: 512 mother-child pairs (238 from non-fluoridated areas, 162 from fluoridated areas; 264 females, 248 males)

Data relevant to the review: Adjusted associations between maternal urinary fluoride across all three trimesters or estimated maternal fluoride intake or drinking water fluoride and IQ.

Reported association with fluoride exposure: Yes: Significant inverse associations between maternal urinary fluoride and IQ in boys (adjusted $\beta = -4.49$ per 1-mg/L increase). No significant associations in girls only, or in both sexes combined. Significant inverse associations between maternal intake and IQ in both sexes combined (adjusted $\beta = -3.66$ per 1-mg/d increase; 95% CI: −7.16, −0.15); Significant inverse associations between drinking water fluoride in both sexes combined (adjusted $\beta = -5.29$ per 1-mg/L increase; 95% CI: −10.39, −0.19). No significant effect modification by sex for maternal fluoride intake and drinking water fluoride associations.

Risk of Bias

Author contacts:

Authors were contacted in June 2019 for additional information for the risk-of-bias evaluation. Additional information provided by the authors informed the rating decision for the following risk-of-bias domains: Other.

Population selection:

Rating: Definitely low risk of bias (++)

Summary: Pregnant women were recruited from the same population during the same time frame and using the same methods as the MIREC program. Methods were reported in detail.

Basis for rating: Definitely low risk of bias based on direct evidence that the exposed groups were similar and were recruited with the same methods during the same time frame.

Confounding:

Rating: Probably low risk of bias (+)

Summary: The study considered several possible covariates, including maternal age, pre-pregnancy BMI, marriage status, birth country, race, maternal education, employment, income, HOME score, smoking during pregnancy, secondhand smoke in the home, alcohol consumption during pregnancy, parity, sex, age at testing, gestational age, birth weight, time of void, and time since last void. The study also conducted secondary analyses to test for lead, mercury, arsenic, and PFOA. There is no indication of any other potential co-exposures in this study population. Iodine deficiency or excess could not be assessed but is not expected to differentially occur. The study was not able to assess parental IQ or mental health disorders. Methods used to obtain the information included questionnaires and laboratory tests.

Potentially important study-specific covariates: All key covariates were addressed.

Direction/magnitude of effect size: Not applicable.

Basis for rating: Probably low risk of bias based on indirect evidence that the methods used to collect the information were valid and reliable and direct evidence that key covariates, including potential co-exposures, were addressed.

Attrition:

Rating: Probably low risk of bias (+)

Summary: Of the 610 recruited children, 601 (98.5%) completed testing. Of the 601 mother-child pairs, 512 (85.2%) had all three maternal urine samples and complete covariate data, and 400 (66.6%) had data available to estimate fluoride intake.

Basis for rating: Probably low risk of bias based on indirect evidence that exclusion of subjects from analyses was adequately addressed, and reasons were documented when subjects were removed from the study or excluded from analyses.

Exposure:

Rating: Probably low risk of bias (+)

Summary: Spot urine samples from all three trimesters of pregnancy were evaluated using appropriate methods, and results were adjusted for creatinine and specific gravity. Fluoride intake was estimated based on fluoride water levels, and information on consumption of tap water and other water-based beverages (e.g., tea, coffee) was obtained via questionnaire.

Direction/magnitude of effect size: There is not any specific direction or magnitude of bias expected. Urinary fluoride levels are reflective of recent exposure. Having measurements from all three trimesters of pregnancy provides a better representation of actual exposure than a single measurement, although the potential for missed high exposure is possible. However, the possibility of the occurrence of missed high exposure would be similar in all females and would be non-differential. For the fluoride intake, exposure was based on the fluoride levels in the water at the residence. If women worked outside the home and the majority of intake occurred from areas outside the home (and were different from levels in the home), there is potential to bias toward the null.

Basis for rating: Probably low risk of bias based on indirect evidence that exposure was consistently assessed using well-established methods that directly measured exposure.

Outcome:

Rating: Probably low risk of bias (+)

© 2025 Taylor KW et al. *JAMA Pediatrics*.

Summary: The Wechsler Preschool and Primary Scale of Intelligence was normalized for ages 2.5–<4.0 and sex using the U.S population-based norms. Blinding was not reported, but it is unlikely that the outcome assessors had knowledge of the maternal fluoride level or were aware of whether the city had fluoridated water.

Basis for rating: Probably low risk of bias based on indirect evidence that the outcome was assessed using instruments that were valid and reliable in the study population, and that the outcome assessors were blind to participants' fluoride exposure.

Selective Reporting:

Rating: Definitely low risk of bias (++)

Summary: All outcomes were reported.

Basis for rating: Definitely low risk of bias based on direct evidence that all measured outcomes were reported.

Other potential threats:

Rating: Definitely low risk of bias (++)

Summary:

Statistical analyses: Multivariate linear regression analyses were used to evaluate the associations between maternal urinary fluoride and fluoride intake and children's IQ scores. Regression diagnostics were used to test assumptions for linearity, normality, and homogeneity. There were no potential influential observations (based on Cook's distance). Sensitivity analyses showed that the effects of maternal urinary fluoride (MUF), fluoride intake, and water fluoride were robust to the exclusion of two very low IQ scores in males (<70). City was accounted for as a covariate in the regression models published. Additional models with city as a random effect were also subsequently made publicly available and showed similar results to the main model.

Other potential concerns: None identified.

Basis for rating: Definitely low risk of bias based on direct evidence that the statistical analyses were appropriate and there were no other potential threats of risk of bias identified.

Basis for classification as low risk-of-bias study overall: Probably low risk-of-bias ratings in confounding, exposure, and outcome. Study strengths include individual exposure assessment measurements, prospective cohort design, and the consideration of key covariates.

Cui et al. (2020)[16]

Study Details

Study design: Cross-sectional

Population: School children aged 7–12 years

Study area: Tianjin City, China (one randomly selected school from each district based on iodine levels in the water), presumably was an expansion of the Cui et al. (2018)[13] study

Sample size: 498 school children

Data relevant to the review: Mean IQ scores by urine fluoride levels.

Reported association with fluoride exposure: Yes: A 2-point decrease in IQ was observed in the highest urinary fluoride group compared to the lowest urinary fluoride group (i.e., 110.00 in ≥2.5-mg/L group versus 112.16 in <1.6-mg/L group); however, the results did not achieve

© 2025 Taylor KW et al. *JAMA Pediatrics*.

statistical significance based on a one-way ANOVA comparing the three different urinary fluoride categories only.

Risk of Bias

Author contacts:

Authors were not contacted for the 2020 publication. Authors were contacted in June 2019 for additional information on the Cui et al. (2018)[13] publication. Additional information provided by the authors regarding Cui et al. (2018)[13] informed the rating decision for the following risk-of-bias domains: Detection (outcome assessment).

Population selection:

Rating: Probably low risk of bias (+)

Summary: Subjects were recruited from 2014 to 2018. One school was selected from each district where water concentrations of water iodine were <10, 10–100, 100–150, 150–300 and >300 µg/L. In each school, classes were randomly sampled for the appropriate age group of 7–12 years old. A table of subject characteristics was provided by IQ. A total of 620 children were recruited, and 122 children who did not have complete information or enough blood sample were excluded. Reasons for exclusion do not appear to be related to exposure or outcome. The characteristics of the 498 included children are presented.

Basis for rating: Probably low risk of bias based on indirect evidence that the exposure groups were similar and were recruited within the same time frame using the same methods, with no evidence of differences in participation/response rates.

Confounding:

Rating: Probably high risk of bias (−)

Summary: It was noted by the study authors that there were no other environmental poisons except water fluoride. Other studies also conducted in this area of China noted specifically that arsenic was not a concern. Iodine was addressed as that was one of the main points of the study. Twenty-one factors (provided in Table 1 of the study) were selected as covariates, and a homemade questionnaire of unspecified validity was used for obtaining the information. It was noted that child age, stress, and anger were significantly associated with IQ although it is unclear whether these varied by fluoride level. However, Cui et al. (2018)[13] indicate that fluoride was not significantly associated with stress and anger, and it was assumed that results would be similar for this study even though more children were included.

Potentially important study-specific covariates: Age (children 7–12 years old)

Direction/magnitude of effect size: Age is a key covariate for IQ, even in the narrow age range evaluated in this study. The direction of the association may depend on the number of children in each age group within the different urinary fluoride categories; however, these data were not provided. In general, there were fewer subjects ≤9 years of age (i.e., 111) compared to >9 years of age (i.e., 387) with a significantly higher IQ in the ≤9-year-old age group. Therefore, if exposure were higher in the older subjects, this could likely bias the association away from the null.

Basis for rating: Probably high risk of bias because there is indirect evidence that age was not addressed as a key covariate, and it may be related to both IQ and exposure.

Attrition:

Rating: Probably low risk of bias (+)

Summary: Of the 620 children recruited, 122 (20%) were excluded due to incomplete information or inadequate blood samples. No information was provided to indicate whether there were similarities or differences in the children included versus the children excluded, but exclusion is unlikely to be related to either outcome or exposure.

Basis for rating: Probably low risk of bias based on indirect evidence that exclusion of subjects from analyses was adequately addressed, and reasons were documented when subjects were removed from the study or excluded from analyses.

Exposure:

Rating: Probably low risk of bias (+)

Summary: Children's morning urine was collected with a clean polyethylene tube, and fluoride was measured using a fluoride ion-selective electrode following Chinese standard WS/T 89-2015. A brief description was provided, but no QC methods were reported. The study authors do not account for urinary dilution in the spot samples.

Direction/magnitude of effect size: Not accounting for dilution could cause some exposure misclassification. The direction and magnitude would depend on where the differences occurred.

Basis for rating: Probably low risk of bias based on indirect evidence that exposure was consistently assessed using acceptable methods that provide individual levels of exposure.

Outcome:

Rating: Probably low risk of bias (+)

Summary: IQ was measured using the Combined Raven's Test, which is an appropriate test for the study population (++ for methods). Blinding was not mentioned; however, the outcome assessors would not likely have had knowledge of the child's urinary fluoride. Subjects appear to have been recruited based on iodine levels; therefore, it is unlikely that there would have been any knowledge of potential fluoride exposure. Correspondence with the study authors for the Cui et al. (2018)[13] study also indicated that the outcome assessors would have been blind.

Basis for rating: Probably low risk of bias based on indirect evidence that the outcome was assessed using instruments that were valid and reliable in the study population, and that the outcome assessors were blind to participants' fluoride exposure.

Selective Reporting:

Rating: Definitely low risk of bias (++)

Summary: All outcomes in the abstract, introduction, and methods are reported in sufficient detail.

Basis for rating: Definitely low risk of bias based on direct evidence that all measured outcomes were reported.

Other potential threats:

Rating: Probably low risk of bias (+)

Summary:

Statistical analyses: One-way ANOVA was used to make comparisons between mean IQ by urinary fluoride levels. Consideration of heterogeneity of variances was not reported. There is no adjustment for covariates or for clustering of children at the school level. There is no evidence that the sampling strategy was otherwise accounted for (i.e., via sampling weights). The impact of these factors on the effect

estimates is expected to be minimal given the use of individual-level data. The primary focus of the study was to evaluate associations between IQ and thyroid hormone or dopamine levels (not between IQ and fluoride levels). It should also be noted that more advanced analyses used for thyroid hormone- and dopamine-IQ associations still lacked adjustment for school and accounting for clustering of children from the same school.

Other potential concerns: None identified.

Basis for rating: Probably low risk of bias based on indirect evidence that the statistical analyses were appropriate, and there were no other potential threats of risk of bias identified.

Basis for classification as low risk-of-bias study overall: Probably low risk-of-bias ratings in exposure and outcome. Study strengths include individual exposure measurements, but the study is limited by the cross-sectional study design, lack of accounting for urine dilution, and lack of addressing age as a key covariate.

Till et al. (2020)[17]

Study Details

Study design: Prospective cohort

Population: MIREC participants (pregnant mothers and their children aged 3–4 years)

Study area: 10 cities, Canada

Sample size: 398 mother-child pairs (247 from non-fluoridated areas, 151 from fluoridated areas; 200 breastfed as infants, 198 formula-fed as infants)

Data relevant to the review: Adjusted associations between water fluoride concentration (with or without adjusting for maternal urine) in formula-fed or breastfed infants or fluoride intake from formula and IQ scores.

Reported association with fluoride exposure: Yes: Significant inverse association between water fluoride and performance IQ by breastfeeding status (adjusted $\beta = -9.26$ formula-fed, -6.19 breastfed per 0.5-mg/L increase) and between fluoride intake from formula and performance IQ (adjusted $\beta = -8.76$ per 0.5-mg/d increase); significant inverse association between water fluoride and full-scale IQ in formula-fed children (adjusted $\beta = -4.40$ per 0.5-mg/L increase); no significant changes in full-scale IQ for water fluoride in breastfed children or fluoride intake from formula-fed children; no significant changes in verbal IQ scores with fluoride exposure.

Risk of Bias

Author contacts:

Authors were not contacted for the 2020 publication. Authors were contacted in June 2019 for additional information on the Green et al. (2019)[15] publication. Information obtained from that correspondence may have been used for additional information in the 2020 publication.

Population selection:

Rating: Definitely low risk of bias (++)

Summary: Pregnant women were recruited between 2008 and 2011 by the MIREC program from 10 cities across Canada. Inclusion and exclusion criteria were provided. Additional details were stated to be available in Arbuckle et al. (2013)[18]. A total of 610 children were recruited to participate in the developmental follow-up with 601 children

completing all testing. The demographic characteristics of women included in the current analyses (n = 398) were not substantially different from the original MIREC cohort (n = 1,945) or the subset without complete water fluoride and covariate data (n = 203). A table of characteristics of the study population was provided. Approximately half of the children lived in non-fluoridated cities and half lived in fluoridated cities.

Basis for rating: Definitely low risk of bias based on direct evidence that the exposed groups were similar and were recruited with the same methods during the same time frame.

Confounding:

Rating: Probably low risk of bias (+)

Summary: Covariates were selected a priori that have been associated with fluoride, breast feeding, and children's intellectual ability. Final covariates included sex and age at testing, maternal education, maternal race, secondhand smoke in the home, and HOME score. City was considered but excluded from the models. Covariates that were not assessed include parental mental health, iodine deficiency/excess, parental IQ, and co-exposure to arsenic and lead. Co-exposure to arsenic is less likely an issue in this Canadian population because it receives water mainly from municipal water supplies that monitor for lead and arsenic, and the lack of information is not considered to appreciably bias the results. In addition, a previous study on this population [15] conducted sensitivity analyses on co-exposures to lead and arsenic. Results from these sensitivity analyses support the conclusion that co-exposures to lead and arsenic are not likely a major concern in this study population.

Potentially important study-specific covariates: All key covariates were considered in this study.

Direction/magnitude of effect: Not applicable.

Basis for rating: Probably low risk of bias based on direct evidence that key covariates were considered and indirect evidence that the methods used to collect the information were valid and reliable and co-exposures were not an issue.

Attrition:

Rating: Probably low risk of bias (+)

Summary: Of 610 children, 601 (98.5%) in the MIREC developmental study who were ages 3–4 years completed the neurodevelopment testing. Of the 601 children who completed the neurodevelopmental testing, 591 (99%) completed the infant feeding questionnaire and 398 (67.3%) reported drinking tap water. It was noted that the demographic characteristics were not substantially different from the original MIREC cohort or the 203 subjects without complete water fluoride or covariate data.

Basis for rating: Probably low risk of bias based on indirect evidence that exclusion of subjects from analyses was adequately addressed, and reasons were documented when subjects were removed from the study or excluded from analyses.

Exposure:

Rating: Probably low risk of bias (+)

Summary: Information on breastfeeding was obtained via questionnaire at 30–48 months. Fluoride concentration in the drinking water was assessed by daily or monthly reports provided by water treatment plants. Water reports were first linked with mothers' postal codes, and the daily or weekly amounts were averaged over the first 6 months of each child's life. Additional details can be found in Till et al. (2018)[19]. Maternal urinary

© 2025 Taylor KW et al. *JAMA Pediatrics*.

exposure was used to assess fetal fluoride exposure. Procedures can be found in Green et al. (2019)[15].

Direction/magnitude of effect size: There is not any specific direction or magnitude of bias expected. Urinary fluoride levels are reflective of recent exposure. The possibility of exposure misclassification would be similar in all subjects and would be non-differential. For the fluoride intake from formula, exposure was based on the fluoride levels in the water at the residence and the proportion of time that the infant was not exclusively breastfed. This exposure misclassification would also be non-differential.

Basis for rating: Probably low risk of bias based on indirect evidence that exposure was consistently assessed using well-established methods that directly measured exposure.

Outcome:

Rating: Probably low risk of bias (+)

Summary: Intelligence was tested using the Wechsler Preschool and Primary Scale of Intelligence III, which is considered a gold standard test. It is appropriate for both the study population and age group. It was not reported whether the evaluators were blind to the child's fluoride exposure status during the assessment. Although it is unlikely that the assessors had knowledge of the specific drinking water levels or maternal urine levels, there is potential that the outcome assessors had knowledge of the city the child lived in and whether the city was fluoridated or non-fluoridated. Correspondence with the study authors on the outcome assessment for Green et al. (2019)[15] indicated that it was unlikely that the testers had knowledge of the city's fluoridation. The same is assumed here. Specific measurements included were identified.

Basis for rating: Probably low risk of bias based on indirect evidence that the outcome was assessed using instruments that were valid and reliable in the study population, and that the outcome assessors were blind to participants' fluoride exposure.

Selective Reporting:

Rating: Definitely low risk of bias (++)

Summary: All outcomes outlined in the abstract, introduction, and methods were reported in sufficient detail.

Basis for rating: Definitely low risk of bias based on direct evidence that all measured outcomes were reported.

Other potential threats:

Rating: Probably low risk of bias (+)

Summary:

Statistical analyses: Regression diagnostics were used to test assumptions for linearity, normality, and homogeneity. There were two potential influential observations (based on Cook's distance), and sensitivity analyses re-estimated the models without these two variables. Effect modification by breastfeeding status was evaluated. Interestingly, all regression coefficients were divided by 2 to represent change in IQ per 0.5-mg/L change in fluoride. One concern is posed by the lack of accounting for city in the regression models, ideally as a random effect. The authors explored including city as a covariate in the models; however, city was not included either because it was strongly multi-collinear with water fluoride concentration (VIF > 20) (model 1, with water fluoride concentration) or because fluoride intake from formula is a function of water fluoride concentration (assessed at the city level) and was

© 2025 Taylor KW et al. *JAMA Pediatrics*.

therefore deemed redundant (model 2). However, the models use city-level water fluoride concentrations—and, in sensitivity analyses, adjust for maternal urinary fluoride—which warrants exploration of city as a random effect rather than a fixed effect (as would be the case by having it included as a covariate). Even including individual-level maternal urinary fluoride might not fully account for lack of a city effect, given that the subjects were from six different cities, with half of them fully on fluoridated water. Hence, even individual-level exposures are likely to be correlated at the city level. Based on a previous analysis [15], it is unlikely that exclusion of city from models (as a fixed or random effect) would significantly impact the effect estimates.

Other potential concerns: None identified.

Basis for rating: Probably low risk of bias based on indirect evidence that the statistical analyses were appropriate and there were no other potential threats of risk of bias identified.

Basis for classification as low risk-of-bias study overall: Probably low risk-of-bias ratings in confounding, exposure, and outcome. Study strengths include individual exposure measurements, prospective cohort design, and consideration of key covariates.

Wang et al. (2020)[20]

Study Details

Study design: Cross-sectional

Population: School children aged 7–13 years

Study area: Tianjin City, China [possibly a subset of the children from Yu et al. (2018)[14]]

Sample size: 571 school children

Data relevant to the review: Adjusted associations between urine and water fluoride levels and IQ scores.

Reported association with fluoride exposure: Yes: Significant inverse associations between water fluoride and IQ scores (adjusted $\beta = -1.587$ per 1-mg/L increase; 95% CI: −2.607, −0.568) and between urinary fluoride and IQ scores (adjusted $\beta = -1.214$ per 1-mg/L increase; 95% CI: −1.987, −0.442) in boys and girls combined. No significant effect modification by sex.

Risk of Bias

Author contacts:

Authors were not contacted for additional information because it was not necessary.

Population selection:

Rating: Definitely low risk of bias (++)

Summary: Subjects were from a cross-sectional study conducted in 2015, but no citation was provided on this cohort [presumably the Yu et al. (2018)[14] cohort]. It was noted that the subjects in that cohort were from districts with historically high or normal fluoride levels. Subjects for this study were selected by using a stratified and multistage random sampling approach. A brief description was provided. The study area consisted of three historically high fluoride areas and four non-endemic areas. A flow diagram was provided for inclusion and exclusion, but this detail was given for all children and not by area. Therefore, it cannot be determined whether the participation differed by area. However, there was a 93% recruitment rate, and the 13 excluded due to missing data

were not likely excluded due to exposure. Detailed characteristics of the study population are provided. Exclusion criteria included: “children who had congenital or acquired diseases affecting intelligence, or a history of cerebral trauma and neurological disorders, or those with a positive screening test history (like hepatitis B virus infection, Treponema palladium infection and Down's syndrome) and adverse exposures (smoking and drinking) during maternal pregnancy, prior diagnosis of thyroid disease, and children who had had missing values of significant factors (2.2%) were also excluded.”

Basis for rating: Definitely low risk of bias based on direct evidence that the exposed groups were recruited using similar methods during the same time frame and that any differences between the exposed groups were accounted for in the statistical analyses.

Confounding:

Rating: Probably low risk of bias (+)

Summary: Study authors noted that the study areas were not exposed to other neurotoxins such as lead, arsenic, or mercury nor were they iodine deficient. Final models included age, sex, child’s BMI, maternal and paternal education, household income, and low birth weight. The other covariates that were considered are unclear as the authors only noted that the covariates were selected based on current literature. Reasons for exclusion included history of disease affecting intelligence, history of trauma or neurological disorders, positive screening test history, or exposures such as smoking or drinking during pregnancy. Information was obtained by questionnaire or measurements. Covariates such as parental BMI, behavioral and mental health disorders, IQ, and quantity and quality of the caregiving environment were not considered.

Potentially important study-specific covariates: All key covariates were considered in this study.

Direction/magnitude of effect size: Not applicable.

Basis for rating: Probably low risk of bias because there is direct evidence that the key covariates were considered and indirect evidence that the methods for collecting the information were valid and reliable and that co-exposure to arsenic was not an issue in this area.

Attrition:

Rating: Definitely low risk of bias (++)

Summary: A detailed chart of the recruitment process is presented. The study had a 93% recruitment rate, and only 2.2% of subjects with missing data for certain covariates were excluded.

Basis for rating: Definitely low risk of bias based on direct evidence that exclusion of subjects from analyses was adequately addressed, and reasons were documented when subjects were removed from the study or excluded from analyses.

Exposure:

Rating: Probably low risk of bias (+)

Summary: Children provided spot urine samples, presumably at the time of examination. Water samples were randomly collected from public water supplies in each village. Fluoride concentrations were analyzed using fluoride ion-selective electrode according to the national standardized method in China. There is no indication of whether the urine samples accounted for dilution.

© 2025 Taylor KW et al. *JAMA Pediatrics*.

Direction/magnitude of effect size: Not accounting for dilution could cause some exposure misclassification. The impact on the direction and magnitude of effect size would depend on where the differences occurred.

Basis for rating: Probably low risk of bias based on indirect evidence that exposure was consistently assessed using acceptable methods that provide individual levels of exposure.

Outcome:

Rating: Definitely low risk of bias (++)

Summary: Assessments of IQ scores were conducted by graduate students at the School of Public Health, Tongji Medical College, Huazhong University of Science and Technology. Each team member was assigned a single task, meaning that only one person would have conducted the IQ tests. A Combined Raven's Test for Rural China was used. Therefore, the test was appropriate for the study population (++ for method). It was noted that the examiner was trained and blind to exposure (++ for blinding). Overall = ++

Basis for rating: Definitely low risk of bias based on direct evidence that the outcome was assessed using instruments that were valid and reliable in the study population, and that the outcome assessor was blind to participants' fluoride exposure.

Selective Reporting:

Rating: Definitely low risk of bias (++)

Summary: All outcomes in the abstract, introduction, and methods are reported in sufficient detail.

Basis for rating: Definitely low risk of bias based on direct evidence that all measured outcomes were reported.

Other potential threats:

Rating: Probably low risk of bias (+)

Summary:

Statistical analyses: Logistic and multivariate regression models accounting for covariates were used. Results are presented as betas or ORs and 95% CIs. Regression diagnostics were conducted for all models, including examination of multicollinearity, heteroscedasticity, and influential observations. Mediation and interaction analyses were appropriate. There is no evidence that the stratified and multistage random sampling approach for subject selection was accounted for in the analyses by using sampling weights or accounting for clustering using random effect models; however, selected villages were similar in population and general demographic characteristics. Given the use of individual-level data and adjustment for important covariates, the impact on the regression coefficients is likely to be minimal.

Other potential concerns: None identified.

Basis for rating: Probably low risk of bias based on indirect evidence that the statistical analyses were appropriate and no other potential threats of risk of bias were identified.

Basis for classification as low risk-of-bias study overall: Definitely or probably low risk-of-bias ratings in confounding, exposure, and outcome. Study strengths include individual exposure assessment measurements but is limited by the cross-sectional study design and lack of accounting for urine dilution. All key covariates were considered in the study design or analysis.

© 2025 Taylor KW et al. *JAMA Pediatrics*.

Cantoral et al. (2021)[21]

Study Details

Study design: Prospective cohort

Population: Programming Research in Obesity, Growth, Environment and Social Stressors (PROGRESS) cohort (pregnant mothers and their children aged 12 or 24 months).

Study area: Mexico City, Mexico

Sample size: 103 mother-child pairs who had dietary fluoride intake data and Bayley-III (cognitive) analyses.

Data relevant to the review: Adjusted and unadjusted associations between maternal fluoride intake during pregnancy and toddler's cognitive scores at 12 and 24 months of age.

Reported association with fluoride exposure: Yes: Significant inverse association between overall maternal fluoride intake and cognitive scores in boys at 24 months (adjusted β = −3.50 per 0.5-mg/d increase; 95% CI: −6.58, −0.42). No significant associations in girls.

Risk of Bias

Author contacts:

Authors were contacted for additional information on quantitative data not reported in the manuscript, but no response was received.

Population selection:

Rating: Probably low risk of bias (+)

Summary: Subjects were participants of the Programming Research in Obesity, Growth, Environment, and Social Stressors (PROGRESS) cohort, which included 948 mother-child pairs from Mexico City. Inclusion and exclusion criteria for the cohort were provided. From the children available with neurodevelopment assessments, subjects were selected who had information of maternal dietary intake. This included 103 subjects. The study authors provided a flow chart of the included participants and compared characteristics of the included and the excluded subjects. There were no differences in the subject characteristics, but at 12 months there was a significant difference in the cognitive scores between those included and excluded. No difference in cognitive scores were observed at 24 months.

Basis for rating: Probably low risk of bias based on indirect evidence that subjects were similar and recruited using the same methods during the same time frame.

Confounding:

Rating: Probably low risk of bias (+)

Summary: Data were obtained from general questionnaire. SES was assessed using a validated questionnaire for categorizing SES in Mexican families. Calcium intake was assessed using the same validated food frequency questionnaire used for assessing fluoride intake. Analysis accounted for mother's age, education, calcium intake, and SES, birth weight, gestational age, child's sex, and breastfeeding practices. Arsenic was not addressed but it is included in the water quality control program in Mexico City and thus not considered a concern in this population.

Potentially important study-specific covariates: All key covariates were addressed.

Direction/magnitude of effect size: Not applicable.

Basis for rating: Probably low risk of bias based on direct evidence that key covariates were addressed, direct and indirect evidence that the methods used to collect the information were valid and reliable and that arsenic is not likely to be an issue in this study population.

Attrition:

Rating: Probably low risk of bias (+)

© 2025 Taylor KW et al. *JAMA Pediatrics*.

Summary: There was a clear indication of subjects from PROGRESS included and excluded from the current analysis with rationale as to why. The study authors included a table to compare characteristics of those included and excluded from the current study. There was a significant difference in the outcome of interest, but it is not possible to assess if this was related to exposure because the subjects were excluded due to lack of exposure information. However, since exposure was assessed through a validated food frequency questionnaire it is unlikely that exposure would be related to exclusion in a manner that would significantly bias the results.

Basis for rating: Probably low risk of bias based on indirect evidence that exclusion of subjects from analyses was adequately addressed, and reasons were documented when subjects were removed from the study or excluded from analyses.

Exposure:

Rating: Probably high risk of bias (−)

Summary: Fluoride intake was evaluated using a validated food frequency questionnaire (FFQ). This was administered by trained personnel to mothers in the second and third trimesters. Total fluoride intake per day (mg/d) was calculated using the Fluoride Content Tables in Foods and Beverages from Mexico City which are based on fluoride measured in most consumed foods and beverages reported in the Mexican National Health and Nutrition Survey (2016).22 For this national survey, the samples were analyzed for fluoride in duplicate using a modified hexamethyldisiloxane micro-diffusion method. It was noted that salt is fluoridated in Mexico at 250 ppm, and the FFQ asked about practices of adding salt via saltshaker to their meals; 27% of the subjects reported adding salt to their meals. It was noted that in women who completed both questionnaires there was a moderate correlation between dietary fluoride intake levels at trimester 2 and at trimester 3. Median and IQR dietary fluoride intake levels were reported in abstract, results and Table 2. There is some discrepancy in levels reported in results and Table 2, and there is no information provided to indicate why there is a difference in the numbers. Overall fluoride intake levels are reported correctly in abstract and Table 2. Therefore, there is uncertainty in what fluoride intake levels were used in the analysis.

Direction/magnitude of effect size: There is some concern on how salt intake was considered. Reliance on self-report on whether participants added salt to their meals (yes/no question) and lack of quantitative data on the amount of salt participants who answered yes used are likely to introduce exposure misclassification. However, there is no information to indicate that there would be differential salt intake or that it was handled differently across trimesters or participants. The potential misclassification is likely non-differential and not likely to bias in any specific direction.

Basis for rating: Probably high risk of bias based on indirect evidence that exposure was assessed using insensitive methods.

Outcome:

Rating: Definitely low risk of bias (++)

Summary: Neurodevelopment was assessed at 12 and 24 months using the Spanish version of the Bayley Scales of Infants and Toddler Development, Third Edition (Bayley-III) (++ for methods). Children were assessed by trained personnel with a supervising psychologist performing quality control checks by reviewing videotaped evaluations. Examiners were noted to be blinded to maternal fluoride intake levels (++ for blinding). Overall rating for methods and blinding = ++.

Basis for rating: Definitely low risk of bias based on direct evidence that the outcome was assessed using instruments that were valid and reliable in the study population, and that the outcome assessors were blind to participants' fluoride exposure.

Selective Reporting:

Rating: Probably low risk of bias (+)

© 2025 Taylor KW et al. *JAMA Pediatrics*.

Summary: The association between dietary fluoride intake in pregnancy and cognitive score (averaged across both time points s at 12 and 24 months) were not reported for both sexes combined. This is likely because the main analysis observed a sex interaction at 24 months, and not due to any specific selective reporting.

Basis for rating: Probably low risk of bias based on indirect evidence that all measured outcomes and analyses were reported.

Other potential threats:

Rating: Probably low risk of bias (+)

Summary:

Statistical analyses: Linear regression models were used to assess associations between maternal fluoride intake and cognitive scores at each time point. Interactions between fluoride intake and child's sex were also evaluated. Mixed effects linear regression models were used to evaluate associations between maternal fluoride intake and neurodevelopmental functioning across 12 and 24 months of age.

Other potential concerns: Although the number of boys and girls were reported for the total cohort (52 and 52, respectively), they were not reported for each time point. Therefore, it is likely that for the sex-stratified time-specific analyses, the study had small sample sizes (acknowledged by the study authors) and limited power and ability to detect an association. However, significant associations were observed for boys.

Basis for rating: Probably low risk if bias is based on indirect evidence that the statistical analyses were appropriate and there were no other potential threats of risk of bias identified.

Basis for classification as low risk-of-bias study overall: Definitely or probably low risk-of-bias ratings in confounding and outcome. Study strengths include individual exposure measurements, blinding of outcome assessor to participants' fluoride exposure, and the prospective cohort study design.

Ibarluzea et al. (2021)[23]

Study Details

Study design: Prospective cohort

Population: Infancia y Medio Ambiente (Childhood and Environment, INMA) participants (pregnant mothers and their children aged 1 and 4 years old).

Study area: Gipuzkoa, Spain

Sample size: 316 mother-child pairs with Bayley scores, 248 with McCarthy scores.

Data relevant to the review: Mean Bayley and McCarthy scores in nonfluoridated and fluoridated areas. Adjusted associations between maternal urinary fluoride and Bayley and McCarthy scores.

Reported association with fluoride exposure: Yes: Significant association between maternal urinary fluoride across the whole pregnancy and McCarthy scores in boys (adjusted β for verbal scores = 13.86 per 1-mg/g increase; 95% CI: 3.91, 23.82; adjusted β for performance scores = 5.86 per 1-mg/g increase; 95% CI: 0.32, 11.39). No significant associations with Bayley Mental Development Index scores. No significant associations in girls.

Risk of Bias

Author contacts:

Authors were contacted in December 2021 for additional quantitative information. Additional information was provided by the study authors to inform data extraction.

Population selection:

Rating: Probably low risk of bias (+)

© 2025 Taylor KW et al. *JAMA Pediatrics*.

Summary: Subjects were selected from a birth cohort established in Gipuzkoa, Spain. It was noted that within the different subcohorts in the INMA project, Gipuzkoa was the only active community fluoridated drinking water program that served more than 30,000 inhabitants. The study area covered two regions (Goierri and Urola) with 25 municipalities (15 of which included fluoridation). Mothers were recruited during the first antenatal visit to the gynecologist in the public referral hospital. Inclusion criteria were provided. A table of characteristics was provided for the different tests.

Basis for rating: Probably low risk of bias based on indirect evidence that subjects were similar and recruited using the same methods during the same time frame.

Confounding:

Rating: Probably high risk of bias (−)

Summary: Covariate data on maternal sociodemographic characteristics were obtained using questionnaires completed during the first and third trimesters. Questionnaires were administered face-to-face by trained interviewers. Covariates considered included maternal age, maternal social class, maternal education, country of birth, BMI, parity, smoking during pregnancy, alcohol consumption, diet, and duration of breastfeeding. When the children were about 15 months the mother's IQ was tested using the Similarities subtest of the WAIS-III. Child's characteristics considered included sex, birth order, whether they were premature, small for gestational age, and whether they attended kindergarten before the age of 2. Questionnaires were administered face-to-face by trained interviewers. Arsenic, iodine, and manganese were measured in the maternal urine samples. Mercury and lead were measured in cord blood. Sensitivity analyses including other neurotoxicants (As, Mn, Pb, Hg, As and Pb interactions) and iodine were conducted and showed that except for Hg results did not change significantly. There is concern that fish consumption was not accounted for in this population. Based on an evaluation of Hg and neurodevelopment in the INMA cohort by Llop et al. (2017), this study should have accounted for fish consumption (which was likely higher in the fluoridated area based on the higher Hg concentrations). Llop et al. (2017) indicated that a doubling of Hg was associated with an increase in IQ when accompanied by greater fish consumption. Therefore, not adjusting for fish consumption is likely to bias the results.

Potentially important study-specific covariates: All key covariates were addressed, but there is indirect evidence that the study population had high seafood consumption that should have been addressed in the analysis, and the lack of adjusting for this could significantly impact the results for this study population.

Direction/magnitude of effect size: Based on the results provided by Llop et al. (2017), the direction of the effect is likely away from the null in a positive direction. The magnitude of effect cannot be determined because the relationship between fluoride and seafood is likely different from that observed by Llop et al. (2017) between mercury and seafood.

Basis for rating: Probably high risk of bias based on indirect evidence that there was a key covariate for this study population (i.e., seafood consumption) that was not addressed.

Attrition:

Rating: Probably low risk of bias (+)

Summary: A table of characteristics comparing the children from the Gipuzkoa cohort that were included with those excluded from the study showed no significant differences. No information was provided on the children lost to follow-up from age 1 to age 4, but participants' characteristics are compared to those excluded at each time point.

Basis for rating: Probably low risk of bias based on indirect evidence that exclusion of subjects from analyses was adequately addressed, and reasons were documented when subjects were removed from the study or excluded from analyses.

© 2025 Taylor KW et al. *JAMA Pediatrics*.

Exposure:

Rating: Definitely low risk of bias (++)

Summary: Urinary fluoride concentrations were measured in spot maternal urine samples collected during the first and third trimesters. Fluoride content was measured using ion-selective electrode-based analysis. Quality control included using a reference urine sample and blanks. Urinary dilution was addressed by using creatinine-adjusted levels.

Direction/magnitude of effect size: Not applicable.

Basis for rating: Definitely low risk of bias based on direct evidence that exposure was consistently assessed using well-established methods that directly measured exposure.

Outcome:

Rating: Definitely low risk of bias (++)

Summary: Outcome was assessed using the Bayley Scales of Infant Development (BSDI) in 1-year old children and the McCarthy Scales of Children's Abilities (MSCA) adapted to the Spanish population in 4-year-old children and (++ for methods). The BSDI and MSCA are well-established tests, and the validity of both tests is well documented. All tests were carried out at the local health centers by specially trained neuropsychologists who were blind to the child's fluoride exposure status (++ for blinding). It was noted that to limit inter-observer variability, a strict protocol was applied including training sessions in which inter-observer differences were discussed.

Basis for rating: Definitely low risk of bias based on direct evidence that the outcome was assessed using instruments that were valid and reliable in the study population, and that the outcome assessor was blind to participants' fluoride exposure.

Selective Reporting:

Rating: Definitely low risk of bias (++)

Summary: All outcomes outlined in the abstract, introduction, and methods were reported in sufficient detail.

Basis for rating: Definitely low risk of bias based on direct evidence that all measured outcomes were reported.

Other potential threats:

Rating: Probably low risk of bias (+)

Summary:

Statistical analyses: Multiple linear regression models were used and included only covariates with a p-value below 0.2 in bivariate analyses. Model assumptions were tested using residual analysis and assumptions were met. Effect modification by sex was evaluated. Sensitivity analyses included adjustments for fluoridated or non-fluoridated area and for neurotoxicants (As, Mn, Pb) and iodine. Although the study area where the women were recruited from covered 15 municipalities with community fluoridated drinking water (CFDW) and 10 with community non-fluoridated drinking water systems (CNFDW), these are not formally accounted for in the analysis (ideally as random effects) beyond the adjustment for area as a fixed effect. However, the use of individual maternal urinary fluoride levels and numerous potential confounders in the models likely captured the community and area effect.

Other potential concerns: None identified.

Basis for rating: Probably low risk if bias is based on indirect evidence that the statistical analyses were appropriate and there were no other potential threats of risk of bias identified.

Basis for classification as low risk-of-bias study overall: Definitely or probably low risk-of-bias ratings in confounding, exposure, and outcome. Study strengths include individual exposure measurements, blinding of outcome assessor to participants' fluoride exposure, and the prospective cohort study design.

Zhao et al. (2021)[24]

Study Details

Study design: Cross-sectional
Population: School children aged 6–11
Sample size: 567 school children.
Data relevant to the review: Unadjusted and adjusted associations between urinary fluoride and IQ scores.
Reported association with fluoride exposure: Yes: Significant inverse association between child's urinary fluoride and IQ scores (adjusted β = −5.957 per 1-log mg/L increase; 95% CI: −9.712, −2.202).

Risk of Bias

Author contacts:

Authors were not contacted for additional information because it was not necessary.

Population selection:

Rating: Probably low risk of bias (+)

Summary: Study participants were selected during the same time frame using the same methods. Exclusion criteria were reported. Areas were divided into historically high fluoride and non-endemic fluorosis areas. A stratified and multistage random strategy was used for study participant sampling. The simple random sampling was used to select 5 towns (4 fluoride and one normal). Then one primary school was selected within each town using the same sampling method. Children aged 6-11 were selected from grades second to fifth in each school using a cluster sampling method. It was not reported how many children were selected from each grade for a total of 616 that were enrolled in the study. A table of participants' characteristics was provided, but not for the different areas. No information was provided on whether the characteristics were similar between the two areas. There is information to indicate that the study sites had concentrations of some potential neurotoxic heavy metals (e.g., lead and arsenic) in drinking water at the standard levels (not specified).

Basis for rating: Probably low risk of bias based on indirect evidence that subjects were similar and recruited using the same methods during the same time frame.

Confounding:

Rating: Probably low risk of bias (+)

Summary: Demographic information was obtained from a questionnaire filled by the parents. BMI was calculated based on height and weight obtained during physical examination. Potential confounders were selected based on current literature as covariates that could influence both urinary fluoride levels and IQ (citing Cui et al. (2018)[13] and Yu et al. (2018)[14]). Models included age, gender, BMI, paternal education, maternal education, household income, abnormal birth and maternal age at delivery as covariates. It was noted that none of the study areas were endemic areas of iodine deficiency, and that levels of arsenic and lead met the drinking water standards.

Potentially important study-specific covariates: All key covariates were addressed.

Direction/magnitude of effect size: Not applicable.

Basis for rating: Probably low risk of bias based on direct evidence that key covariates, including other potential co-exposures, were addressed and indirect evidence that the methods used to collect the information were valid and reliable.

Attrition:

Rating: Probably low risk of bias (+)

Summary: Reasons for exclusions and number of participants are reported. A total of 616 children were enrolled. 12 children were excluded because of negative long-term

© 2025 Taylor KW et al. *JAMA Pediatrics*.

residents, 3 children were excluded because there was immediate family member mentally retarded, 20 children were excluded because of missing IQ test, questionnaire or physical examination, and 14 children were excluded because no results of genotyping measurement, leaving 567 children for the study.

Basis for rating: Probably low risk of bias based on indirect evidence that exclusion of subjects from analyses was adequately addressed, and reasons were documented when subjects were removed from the study or excluded from analyses.

Exposure:

Rating: Probably low risk of bias (+)

Summary: Urinary fluoride concentrations were determined in spot urine samples (first morning void before breakfast) collected with an unspecified method and stored at -20 degrees C. Fluoride content was measured using ion analyzer EA940 with F-ion selective electrode. Samples were tested twice, and the mean of the two results was used. The quantification limit of the method was 0.05 mg/L with a recovery rate of 93.4%–108.3%. However, quality control methods were not reported and urinary dilution was not assessed.

Direction/magnitude of effect size: Not applicable.

Basis for rating: Spot urine samples that did not account for dilution could introduce exposure misclassification. The potential misclassification is likely non-differential and not likely to bias in any specific direction. Probably low risk of bias based on indirect evidence that exposure was consistently assessed using well-established methods that directly measured exposure.

Outcome:

Rating: Probably low risk of bias (+)

Summary: A Combined Raven's Test (CR) (modified in China) was used to evaluate the IQ of each child. For children 8 and older group tests were conducted, but for students younger than 8 the tests were conducted one on one by trained staff. Although the test is a well-established method, two different procedures were used based on age. The test results were inverted into IQ scores according to the norm for children in rural areas in China (CRT-RC2) (+ for methods). All tests were administered in a quiet environment by an examiner who was blind to the participants fluoride exposure (++ for blinding). Overall rating for methods and blinding = +.

Basis for rating: Probably low risk of bias based on indirect evidence that the outcome was assessed using instruments that were valid and reliable in the study population, and that the outcome assessor was blind to participants' fluoride exposure.

Selective Reporting:

Rating: Definitely low risk of bias (++)

Summary: All outcomes outlined in the abstract, introduction, and methods were reported in sufficient detail.

Basis for rating: Definitely low risk of bias based on direct evidence that all measured outcomes were reported.

Other potential threats:

Rating: Probably low risk of bias (+)

Summary:

Statistical analyses: Statistical analyses were appropriate for the study. Exposures were log-transformed because "urinary fluoride concentrations were not normally distributed." Log base was not specified. Multivariate linear regression was used to evaluate associations between fluoride in children's urine and IQ score. The analysis did not account for clustering at the school level. Information was not provided on the 5 schools to indicate that demographics were similar across the schools. Although the analysis used individual-level exposures rather than area-level exposures, if the exposure levels within a certain

© 2025 Taylor KW et al. *JAMA Pediatrics*.

area are highly correlated (which might be expected), then the results might be biased. However, the overall impact on the effect estimate is expected to be minimal given the use of individual data and adjustments for multiple potential confounders.

Other potential concerns: None identified.

Basis for rating: Probably low risk if bias is based on indirect evidence that the statistical analyses were appropriate and there were no other potential threats of risk of bias identified.

Basis for classification as low risk-of-bias study overall: Probably low risk-of-bias ratings in confounding, exposure, and outcome. Study strengths include individual exposure measurements, blinding of outcome assessor to participants' fluoride exposure, and the consideration of key covariates, but it was limited by the cross-sectional study design and lack of addressing dilution in the urine samples.

Feng et al. (2022)[25]

Study Details

Study design: Cross-sectional

Population: Primary school children aged 8–12 years.

Study area: Tongxu County, China

Sample size: 683 school children.

Data relevant to the review: IQ scores in control and high-fluoride groups. Adjusted associations between child's urinary fluoride and IQ scores.

Reported association with fluoride exposure: Yes: Significant inverse association between child's urinary fluoride and IQ score in the high-fluoride area (adjusted $\beta = -2.502$ per 1.0 mg/L increase; 95% CI: −4.411, −0.593). No significant associations in total population or in control area.

Risk of Bias

Author contacts:

Authors were not contacted for additional information because it was not necessary.

Population selection:

Rating: Probably low risk of bias (+)

Summary: The study population was described thoroughly in the Xu et al. (2020)[26] publication. Four primary schools were selected during the same time frame (April–May 2017). The schools were noted to be similar in terms of living conditions, living habits, and dietary structure. The same exclusion criteria were applied. Participants were all 8–12 years old from grades 2–6 and were recruited using a cluster sampling method. Characteristics were provided by exposure category; the analysis accounted for any characteristics that were significantly different by exposure category.

Basis for rating: Probably low risk of bias based on indirect evidence that subjects were similar and recruited using the same methods during the same time frame.

Confounding:

Rating: Probably low risk of bias (+)

Summary: Covariates considered in the analysis included child's age, gender, BMI, age at which pregnancy occurred, gestational weeks, birth modes, birth weight, and paternal and maternal education levels. It was noted that children were similar in terms of living conditions, living habits, and dietary structure, but details were not provided. Covariates were collected using a questionnaire, but reliability and validity was not provided. The previous study[26] noted that the population was not near any industrial sources of neurotoxic environmental pollutants such as arsenic, lead, or mercury and correspondence with the authors of Xu et al., 2020 indicated that arsenic exposure levels

© 2025 Taylor KW et al. *JAMA Pediatrics*.

in the study areas were all below China's national standards, based on monitoring data from the local Center for Disease Control and Prevention.

Potentially important study-specific covariates: All key covariates were addressed.

Direction/magnitude of effect size: Not applicable.

Basis for rating: Probably low risk of bias based on direct evidence that key covariates, including other potential co-exposure to arsenic, were addressed and indirect evidence that the methods used to collect the information were valid and reliable.

Attrition:

Rating: Probably low risk of bias (+)

Summary: The study reports that 694 children were recruited, but 11 children that had IQ scores below 90 were excluded, leaving 683 children in the study. There is no information on exposure characteristics for these children. However, given this very small number exclusions (11 out of 694), even if these children were from the same exposure category, it is unlikely that this would bias the effect estimates.

Basis for rating: Probably low risk of bias based on indirect evidence that exclusion of subjects from analyses was adequately addressed, and reasons were documented when subjects were removed from the study or excluded from analyses.

Exposure:

Rating: Definitely low risk of bias (++)

Summary: Based on the detailed description in Xu et al. (2020)[26], morning urine was collected into cleaned polyethylene tubes and stored at -80 degrees C. Urinary fluoride concentrations were measured using fluoride ion-selective electrode. Samples were measured twice. Recovery rates were noted to be 93.1%–109.93%. Samples were tested for creatinine and 15% were randomly selected to be repeated. The median creatinine adjusted fluoride level was then used to separate the children into the high fluoride group and control group.

Direction/magnitude of effect size: Not applicable.

Basis for rating: Definitely low risk of bias based on direct evidence that exposure was consistently assessed using well-established methods that directly measured exposure.

Outcome:

Rating: Definitely low risk of bias (++)

Summary: IQ was measured using the second edition of the Combined Raven's Test-The Rural in China (CRT-RC2) (++ for method). The test was conducted in the classrooms. Students completed the answer sheets independently under the supervision of trained investigators. It was not reported if the scores were evaluated blind to the exposure, but correspondence with the study authors for the Xu et al. (2020)[26] study indicated that all the outcome assessors and staff who conducted face-to-face interviews were not aware of the exposure (++ for blinding). Overall rating = ++.

Basis for rating: Definitely low risk of bias based on direct evidence that the outcome was assessed using instruments that were valid and reliable in the study population, and that the outcome assessor was blind to participants' fluoride exposure.

Selective Reporting:

Rating: Definitely low risk of bias (++)

Summary: All outcomes outlined in the abstract, introduction, and methods were reported in sufficient detail.

Basis for rating: Definitely low risk of bias based on direct evidence that all measured outcomes were reported.

Other potential threats:

Rating: Probably low risk of bias (+)

© 2025 Taylor KW et al. *JAMA Pediatrics*.

Summary:
Statistical analyses: Generalized linear models and multinomial logistic regression models were used to evaluate the association between urinary fluoride levels and IQ. Logistic regression models which assessed the odds of higher IQ categories (e.g., "Superior") with increasing exposure. The generalized linear model was used to analyze the association between children's urinary fluoride, polymorphisms, and IQ score. Exposure was modeled as tertiles or as a continuous variable. There is no evidence that residual diagnostics were used to examine model assumptions or that the complex sampling design (stratified multistage random sampling) was accounted for in the analysis using sampling weights and adjustment for clustering. The four selected schools were stated to be similar in population and general demographic characteristics. Although the analysis used individual-level exposures rather than area-level exposures, if the exposure levels with a certain area were highly correlated (which might be expected), then the results might still be biased. However, the overall impact on the effect estimates is expected to be minimal given the use of individual-level data and adjustment for multiple potential confounders.
Other potential concerns: None identified.
Basis for rating: Probably low risk if bias is based on indirect evidence that the statistical analyses were appropriate and there were no other potential threats of risk of bias identified.

Basis for classification as low risk-of-bias study overall: Definitely or probably low risk-of-bias ratings in confounding, exposure, and outcome. Study strengths include individual exposure measurements, blinding of outcome assessor to participants' fluoride exposure, the consideration of key covariates, and addressing dilution in the urine samples, but it was limited by the cross-sectional study design.

Goodman et al. (2022a)[27]

Study Details

Study design: Prospective cohort
Population: Early Life Exposures in Mexico to Environmental Toxicants (ELEMENT) participants (pregnant mothers and their children aged 4, 5 or 6–12 years).
Study area: Mexico City, Mexico
Sample size: 348 mother-child pairs.
Data relevant to the review: Adjusted associations between maternal urinary fluoride and IQ scores.
Reported association with fluoride exposure: Yes: Significant inverse association between maternal urinary fluoride and IQ scores in 6-12 year old children (adjusted β for full scale IQ = −2.01 per 0.5-mg/L increase; 95% CI: −3.66, −0.46; adjusted β for performance IQ = −1.80 per 0.5-mg/L increase; 95% CI: −3.39, −0.21; adjusted β for verbal IQ = −1.93 per 0.5-mg/L increase; 95% CI: −3.67, −0.18).

Risk of Bias

Author contacts:
Authors were not contacted for additional information because it was not necessary.
Population selection:
Rating: Probably low risk of bias (+)
Summary: Study participants were part of the ELEMENT project, which enrolled mother-child pairs form three hospitals in Mexico City serving low-to-moderate income families. Participants were recruited as part of four birth cohort studies. Two cohorts (2A and 3) had prenatal information and archived maternal urine samples collected during

pregnancy. Cohort 2A included 327 women recruited in 1997-1999. Cohort 3 included 670 women recruited in 2001-2003 for a randomized control trial of the effects of calcium supplementation on maternal blood lead levels. Inclusion criteria into the current study were provided and were consistent across cohorts. A detailed presentation of reasons for exclusions is provided in Figure 1. The final analysis sample was limited to mothers with pregnancy urine measurements and IQ scores for children at least at two timepoints ages 4, 5 or 6-12) (n=348). Study authors indicate that there were no demographic differences between those with IQ measurements and those without. Study populations appear to be similar, but there may be some differences because participants were selected from two different cohorts that were recruited from slightly different time periods.

Basis for rating: Probably low risk of bias based on indirect evidence that the exposure groups were similar despite the subjects coming from different original study populations for whom different methods were used for recruitment.

Confounding:

Rating: Probably low risk of bias (+)

Summary: Covariates included in the analysis were gestational age, weight at birth, sex, parity, age at outcome, time of testing, smoking history, marital status, maternal age at delivery, maternal education, and cohort/calcium treatment. Study authors noted that they used the same covariates used in Bashash et al. (2017)[11]. However, they noted that they didn't include maternal IQ because maternal education ensured a larger sample size due to missing data; sensitivity analyses with maternal IQ, HOME scores and maternal bone lead were also performed. Data were collected via questionnaire. Similar to Bashash et al. (2017)[11], arsenic is not considered a potential concern in this study population since arsenic is measured as part of the water quality control program in Mexico City.

Potentially important study-specific covariates: All key covariates were addressed.

Direction/magnitude of effect size: Not applicable.

Basis for rating: Probably low risk of bias based on direct evidence that key covariates, including other potential co-exposures, were addressed and indirect evidence that the methods used to collect the information were valid and reliable and that arsenic is not likely to be an issue in this study population.

Attrition:

Rating: Probably low risk of bias (+)

Summary: Although there was a large amount of attrition, the study authors clearly describe the reasons and provide a table of characteristics for the children included in each follow-up. There are only slight differences in the follow-up compared to the primary sample.

Basis for rating: Probably low risk of bias based on indirect evidence that exclusion of subjects from analyses was adequately addressed, and reasons were documented when subjects were removed from the study or excluded from analyses.

Exposure:

Rating: Definitely low risk of bias (++)

Summary: Urinary fluoride concentrations were determined in spot urine samples (second morning void) collected from mothers (during at least one trimester). Fluoride content was measured using ion-selective electrode-based assays. Although QC procedures were not described in this publication, they are described in Bashash et al. (2017)[11]. Extreme outliers (>3.5 standard deviations from the mean) were dropped. Urinary dilution was addressed by using creatinine-adjusted levels.

Direction/magnitude of effect size: Not applicable.

Basis for rating: Definitely low risk of bias based on direct evidence that exposure was consistently assessed using well-established methods that directly measured exposure.

© 2025 Taylor KW et al. *JAMA Pediatrics*.

Outcome:

Rating: Definitely low risk of bias (++)

Summary: Outcome was assessed using the McCarthy Scales of Children's Abilities (MSCA) in children at ages 4 and 5 years (translated into Spanish) and the Spanish version of Wechsler Abbreviated Scale of Intelligence (WASI) in 6–12-year-olds. (++ for methods). The WASI is a well-established test, and the validity of both tests is well documented by the authors. The intraclass correlation coefficient was above 0.9. Three psychologists unaware of the child's prenatal fluoride exposure were used to conduct the tests (++ for blinding). Overall rating for methods and blinding=++.

Basis for rating: Definitely low risk of bias based on direct evidence that the outcome was assessed using instruments that were valid and reliable in the study population, and that the outcome assessor was blind to participants' fluoride exposure.

Selective Reporting:

Rating: Definitely low risk of bias (++)

Summary: All outcomes outlined in the abstract, introduction, and methods were reported in sufficient detail.

Basis for rating: Definitely low risk of bias based on direct evidence that all measured outcomes were reported.

Other potential threats:

Rating: Definitely low risk of bias (++)

Summary:

Statistical analyses: Statistical analyses used were appropriate for the study. Statistical tests of bivariate associations (using Chi-square tests for categorical variables and t-tests for continuous variables) were used to compare the differences between included and excluded study samples. Generalized estimating equations (GEEs) population averaged models for panel data (ordered by time point) with an autoregressive correlation structure were used to assess the association between maternal urinary fluoride and children's IQ scores across time. Individuals were included in the model if they had data for at least two time points. Age-stratified multiple linear regression analyses were used to estimate the associations between maternal urinary fluoride and children's IQ scores at each time point (i.e., age 4, 5, and 6–12). Results are reported as adjusted regression slopes and 95% CIs. Regression models accounted for clustering at the cohort level by using cohort as a fixed effect in the models. In sensitivity analyses, regression diagnostics did not reveal any model specific issues and confirmed the absence of collinearity. Model residuals were approximately normally distributed.

Other potential concerns: None identified.

Basis for rating: Definitely low risk if bias is based on direct evidence that the statistical analyses were appropriate and there were no other potential threats of risk of bias identified.

Basis for classification as low risk-of-bias study overall: Definitely or probably low risk-of-bias ratings in confounding, exposure, and outcome. Study strengths include individual exposure measurements, blinding of outcome assessor to participants' fluoride exposure, and the prospective cohort study design.

Dewey et al. (2023)

Study Details

Study design: Prospective ecological cohort
Population: Calgary cohort of the Alberta Pregnancy Outcomes and Nutrition (APrON) study.
Study area: Calgary, Canada
Sample size: 616 mother-child pairs.

© 2025 Taylor KW et al. *JAMA Pediatrics*.

Data relevant to the review: Adjusted associations between exposure to fluoridated water during pregnancy and IQ scores.

Reported association with fluoride exposure: No: There was no significant association between the fluoride exposure groups and full-scale IQ scores in 3–5-year-old children (adjusted β for full-scale IQ = 0.06; 95% CI: −3.10, 3.23 for partially exposed group compared to non-exposed group; adjusted β for full-scale IQ = 0.36; 95% CI: −2.69, 3.41 for fully exposed group compared to non-exposed group).

Risk of Bias

Author contacts:

Authors were contacted in November and December 2023 for additional quantitative information not reported in the manuscript and for additional information for the risk-of-bias evaluation. Additional information provided by the authors informed data extraction and the rating decision for the following risk-of-bias domains: Confounding and Detection (outcome assessment).

Population selection:

Rating: Probably low risk of bias (+)

Summary: Study participants were selected from the Calgary cohort of the Alberta Pregnancy Outcomes and Nutrition (APrON) study between 2009 and 2012. Women were eligible if they could communicate in English, were <27 weeks gestational age, and were ≥16 years of age. Participation rates were not reported. A subset of 616 maternal-child pairs from Calgary whose children participated in cognitive and executive function assessments at 3–5 years old participated in this study. Demographic characteristics were presented by exposure group and analyses adjusted for potential differences. There is no evidence that recruitment methods were associated with exposure status.

Basis for rating: Probably low risk of bias based on indirect evidence that subjects were similar and recruited using the same methods during the same general timeframe although exposure timeframe was slightly different due to the nature of the exposure being around the cessation of fluoridated water.

Confounding:

Rating: Probably low risk of bias (+)

Summary: Maternal factors (age and education, marital status, income, birthplace, parity, pre-pregnancy BMI, pre-pregnancy smoking, depression), child sex, and infant gestational age at birth were evaluated as potential confounders. Child sex and age, whether or not mothers were born in Canada, and pre-pregnancy smoking were retained in the model evaluating the association between fluoride exposure group and full-scale IQ. It was noted that children's performance was not age standardized and that child age was included as a covariate. Data on covariates were obtained from questionnaires. Covariates that were not assessed included iodine deficiency/excess and co-exposure to arsenic, lead or mercury. Co-exposure to arsenic is not a concern in this Canadian population which receives water mainly from municipal water supplies that typically monitor for lead and arsenic. The lack of information on arsenic is not considered to appreciably bias the results. Correspondence with study authors about iodine mentioned that "Based on the prenatal care records of the women who participated in this study iodine deficiency was not observed in any participants."

Potentially important study-specific covariates: All key covariates were addressed.

Direction/magnitude of effect size: Not applicable.

Basis for rating: Probably low risk of bias based on direct evidence that key covariates were addressed and indirect evidence that the methods used to collect the information were valid and reliable and that arsenic or other co-exposures are not likely to be an issue in this study population.

Attrition:

Rating: Probably low risk of bias (+)

Summary: Of the original 1,969 eligible pregnant women, 616 participated in the cognitive and executive function test at 3–5 years of age. No information was provided to compare the 1,969 to the 616 that participated. The amount of missing data was small and is not a concern: Fluoride exposure group data were 100% complete; covariates were >98% complete; and child neurodevelopmental data were between 93% and 99% complete. Incomplete data were imputed by chained equations using predictive mean matching and classification and regression trees. Five data sets were imputed, and estimates were evaluated in all multiple imputation data sets and combined by pooling.

Basis for rating: Probably low risk of bias based on indirect evidence that exclusion of subjects from analyses was adequately addressed, and reasons were documented when subjects were removed from the study or excluded from analyses.

Exposure:

Rating: Probably high risk of bias (−)

Summary: Three exposure groups based on maternal exposure to fluoride in drinking water from the Calgary community water supply and duration of exposure (fully exposed throughout pregnancy, partially exposed, or not exposed) were assessed. The Calgary water supply stopped adding fluoride in May 2011; therefore, all women with children born before that date were considered fully exposed. Partially exposed were children born between May 19, 2011, and May 13, 2012, which included an additional 90 days (based on expert advice) to ensure fluoride in community supply will reach steady state after cessation of fluoridation). Not exposed were children born after May 13, 2012. Exposure status was not confirmed using measures of fluoride in drinking water or biomarkers of exposure.

Direction/magnitude of effect size: Direction of effect is likely to be toward the null because there may be some overlap in the populations as the timing of partial and no exposure was based on expert opinion. In addition, other sources of fluoride were not addressed. Magnitude of effect is unknown based on uncertainty in actual total fluoride exposure in the different exposure groups.

Basis for rating: Probably high risk of bias based on indirect evidence that exposure was assessed using insensitive methods.

Outcome:

Rating: Definitely low risk of bias (++)

Summary: Intelligence was assessed at ages 3–5 years using the Wechsler Preschool and Primary Scale of Intelligence Fourth Edition: Canadian (WPPSI-IVCND) which is considered a gold standard test (++ for methods). It is appropriate for both the study population and age group. Children's performance on the WPPSI-IV Verbal Comprehension Index (VCI) and Visual Spatial Index (VSI) were also assessed. Correspondence with study authors provided more detail on administration of tests: "The IQ assessment was administered individually to each child by a trained psychometrist who was supervised by a registered PHD clinical psychologist. None of the assessors had any information on the child's exposure group. All scores were age standardized using Canadian norms, which differ from US norms." (++ for blinding). Overall rating: ++ for methods and blinding.

Basis for rating: Definitely low risk of bias based on direct evidence that the outcome was assessed using instruments that were valid and reliable for the study population and that the outcome assessor was blind to participants' fluoride exposure.

Selective Reporting:

Rating: Definitely low risk of bias (++)

© 2025 Taylor KW et al. *JAMA Pediatrics*.

Summary: All outcomes outlined in the abstract, introduction, and methods were reported in sufficient detail.

Basis for rating: Definitely low risk of bias based on direct evidence that all measured outcomes were reported.

Other potential threats:

Rating: Definitely low risk of bias (++)

Summary:

Statistical analyses: Analysis of variance and Chi-square tests were used to examine differences in sample characteristics between fluoride exposure groups. Multiple imputations and robust multivariate regression were used since children's intellectual functioning scores were skewed and violated the assumptions of a normal distribution. Sensitivity analyses removing potential outlier values of the full-scale IQ and removing children with low birthweights assessed the robustness of results for full-scale IQ.

Other potential concerns: None identified.

Basis for rating: Definitely low risk if bias is based on direct evidence that the statistical analyses were appropriate and there were no other potential threats of risk of bias identified.

Basis for classification as low risk-of-bias study overall: Definitely or probably low risk-of-bias ratings in confounding and outcome. Study strengths include blinding of outcome assessor to participants' fluoride exposure and the prospective cohort study design.

Grandjean et al. (2023)

Study Details

Study design: Prospective cohort

Population: Odense Child Cohort (OCC).

Study area: Odense, Denmark

Sample size: 837 mother-child pairs.

Data relevant to the review: Adjusted associations between maternal urinary fluoride and IQ scores.

Reported association with fluoride exposure: Yes: Inverse association between maternal urinary fluoride and full scale IQ scores in 7-year-old children (adjusted β for full scale IQ = −0.72 per doubling of maternal urinary fluoride; 95% CI: −3.24, 1.80) and in girls (adjusted β for full scale IQ = −0.40 per doubling of maternal urinary fluoride; 95% CI: −2.52, 1.71). However, the results were not statistically significant.

Risk of Bias

Author contacts:

Authors were contacted in November 2023 for additional information for the risk-of-bias evaluation. Additional information provided by the authors informed the rating decision for the following risk-of-bias domains: Confounding.

Population selection:

Rating: Probably low risk of bias (+)

Summary: Study participants were similar and recruited during the same timeframe using the same methods. Study participants were pregnant women from the Odense Child Cohort (OCC) recruited between 2010 and 2012. The present study population included 837 singleton mother–child pairs with results on child IQ, a maternal urine sample analyzed for fluoride, and information about parental education, child sex and preterm birth. Population characteristics for the study cohort sample were presented and compared with those of the total cohort. Important characteristics were accounted for in the analyses.

© 2025 Taylor KW et al. *JAMA Pediatrics*.

Details on the OCC recruitment were not provided but are available in a separate publication.

Basis for rating: Probably low risk of bias based on indirect evidence that subjects were similar and recruited using the same methods during the same timeframe.

Confounding:

Rating: Probably low risk of bias (+)

Summary: Child's sex and SES were accounted for in the model and all children were 7 years of age. Additional covariates considered included preterm birth, maternal smoking and alcohol intake during pregnancy, duration of breastfeeding, school type, school grade, psychologist examiner, and parents' highest education. There is no indication of any potential co-exposures to arsenic, lead or mercury. Arsenic is not considered a concern in this study population. Correspondence with the study authors confirmed that arsenic is removed at the drinking water treatment plant, and it rarely exceeds exposure limits in the water supply. Iodine deficiency or excess could not be assessed but correspondence with the study authors indicated that deficiencies and excess iodine are rare in Denmark.

Potentially important study-specific covariates: All key covariates were addressed.

Direction/magnitude of effect size: Not applicable.

Basis for rating: Probably low risk of bias based on direct evidence that key covariates, including other potential co-exposures, were addressed and indirect evidence that the methods used to collect the information were valid and reliable.

Attrition:

Rating: Probably low risk of bias (+)

Summary: 2,874 of the 4,017 women contacted agreed to be enrolled in the OCC; 374 women dropped out before and after giving birth; and 837 singleton mother-child pairs with results on child IQ, a maternal urine sample analyzed for fluoride, and information about parental education, child sex, and preterm birth were included in the current study. Population characteristics for the study cohort sample were presented and compared with those of the total cohort. Important characteristics were accounted for in the analyses.

Basis for rating: Probably low risk of bias based on indirect evidence that exclusion of subjects from analyses was adequately addressed, reasons were documented when subjects were removed from the study or excluded from analyses, and information was provided to indicate potential differences from those included and the original cohort.

Exposure:

Rating: Probably low risk of bias (+)

Summary: Fluoride concentrations in maternal urine samples obtained during late pregnancy (28 weeks gestation) were measured using OrionTM Ion Selective Electrode (ISE 9609 BNWP) (Thermo Fisher Scientific, Waltham, MA, USA) coupled to a Model 15 pH meter from Denver Instruments. All measured urine samples from the OCC cohort had fluoride concentrations higher than the limit of determination (0.02 mg/L), i.e., the lowest point on the calibration curve (CV% < 3%), and quality control methods were noted in the supplemental data. The average imprecision was <5.1%. Urinary dilution was addressed by using creatinine-adjusted levels; however, 384 women collected a 24-hour sample and 453 collected a spot urine sample in the morning. The study authors noted that the level of urinary fluoride did not differ between sampling conditions and cite supplemental table S1 although the table does not provide actual urinary levels but results by the different urine collections.

Direction/magnitude of effect size: Not applicable.

Basis for rating: Probably low risk of bias based on direct evidence that exposure was assessed using well-established methods that directly measured exposure and indirect evidence that the inconsistent methods of collecting the urine samples did not affect the exposure assessment.

© 2025 Taylor KW et al. *JAMA Pediatrics*.

Outcome:

Rating: Definitely low risk of bias (++)

Summary: Intelligence was assessed at age 7 years using an abbreviated version of the Wechsler Intelligence Scales for Children, considered a gold standard. Children attending schools outside the Municipality of Odense were either invited to complete the test in OCC facilities or offered a home visit. A Danish version of the Wechsler Intelligence Scale for Children, 5th edition (WISC-V) was administered by trained psychologists (primarily by one psychologist [tester 1] assisted by three psychologists [testers 2–4] throughout the period). An experienced psychologist acted as supervisor for the four psychologists. All discrepancies in scoring were discussed and rated in consensus to secure high interrater reliability (++ for methods). Examiners were blinded to fluoride exposure status (++ for binding). Overall rating for methods and blinding = ++.

Basis for rating: Definitely low risk of bias based on direct evidence that the outcome was assessed using instruments that were valid and reliable for the study population, and that the outcome assessor was blind to participants' fluoride exposure.

Selective Reporting:

Rating: Definitely low risk of bias (++)

Summary: All outcomes outlined in the abstract, introduction, and methods were reported in sufficient detail.

Basis for rating: Definitely low risk of bias based on direct evidence that all measured outcomes were reported.

Other potential threats:

Rating: Definitely low risk of bias (++)

Summary:

Statistical analyses: Multivariate linear regression was used to model the associations between maternal urinary fluoride exposure (log2 transformed because of skewness) and child full-scale IQ scores. Sex was introduced in models as an interaction term with maternal fluoride. The creatinine-adjusted urinary fluoride was also stratified for the type of urine sample available (i.e. 24-hr and spot), and a joint analysis was conducted with a fixed effect for the type of urine sample. A cubic spline model was also used for descriptive purposes.

Other potential concerns: None identified.

Basis for rating: Definitely low risk of bias is based on direct evidence that the statistical analyses were appropriate and there were no other potential threats of risk of bias identified.

Basis for classification as low risk-of-bias study overall: Definitely or probably low risk-of-bias ratings in confounding, exposure, and outcome. Study strengths include individual exposure measurements, blinding of outcome assessor to participants' fluoride exposure, and the prospective cohort study design.

Lin et al. (2023)

Study Details

Study design: Cross-sectional

Population: School children aged 6–12 years

Study area: Taichung, Taiwan

Sample size: 562 school children

Data relevant to the review: Adjusted associations between child's urinary fluoride and IQ scores.

Reported association with fluoride exposure: Yes: Inverse association between creatinine-adjusted natural log-transformed child's urinary fluoride and IQ scores in 6–12-year-old

© 2025 Taylor KW et al. *JAMA Pediatrics*.

children (adjusted β for full scale IQ = −2.21 per ln-mg/g increase; 95% CI: −4.99, 0.57); however, results were not statistically significant ($p = 0.119$). The change in mean IQ score for a 10% increase in urine fluoride was −0.21 (95% CI: −0.48, 0.05).

Risk of Bias

Author contacts:

Authors were contacted in November 2023 for additional quantitative information not reported in the manuscript and for additional information for the risk of bias evaluation. Information provided by the authors informed data extraction and the rating decision for the following risk-of-bias domains: Confounding and Detection (outcome assessment).

Population selection:

Rating: Probably low risk of bias (+)

Summary: Study participants were school children in Taichung, Taiwan, recruited from five elementary schools, which were randomly sampled from districts with different urbanization levels. Two schools were randomly sampled from each group with the probability proportional to size. Within each school, two classes from each of the six grades were invited to participate in the study. Invitation letters and consent forms were sent to 1,380 students from 60 classes, and 562 children who completed the measurement of urinary fluoride, the evaluation of dental fluorosis, and the assessment of intelligence were included in the data analysis. Reasons for exclusion do not appear to be related to exposure or outcome. Participant characteristics are presented by dental fluorosis group.

Basis for rating: Probably low risk of bias based on indirect evidence that subjects were similar and recruited using the same methods during the same timeframe.

Confounding:

Rating: Probably high risk of bias (−)

Summary: Children's age, gender, BMI, history of physician-diagnosed diseases, food intake habits (e.g., the frequencies for intake of foods with high fluoride), usage of systemic and topical fluoride products, and parents' educational levels were evaluated as potential confounders. Covariates not considered included parity, quantity and quality of caregiving environment, co-exposures (such as arsenic, lead, mercury), and iodine deficiency. Questionnaires were used to collect demographic data. The child's body weight and height were measured by one trained researcher. Correspondence with study authors confirmed that exposures to arsenic, lead, and mercury were not controlled for in the study and that iodine deficiency was not evaluated in the study population. However, drinking water concentrations of arsenic, lead, and mercury in the water treatment plants that serve the study area were very low or not detected. Iodine deficiency was also unlikely since the author correspondence revealed that "77% of 7 to 12 year old children in Taiwan use iodized salt." There is concern that seafood consumption was not accounted for in this population. The frequency of seafood consumption was significantly different between children with and without dental fluorosis in the study. Given that seafood consumption is typically associated with increased IQ, not adjusting for seafood consumption is likely to bias the results.

Potentially important study-specific covariates: All key covariates were addressed, but indirect evidence that the study population had high seafood consumption that should have been addressed in the analysis and the lack of adjusting could significantly impact the results for this study population.

Direction/magnitude of effect size: Direction and magnitude of effect is unknown. Llop et al. (2017) provide evidence that greater fish consumption will increase IQ even when accompanied by an increase in mercury exposure; therefore, it is likely that the same would apply with fluoride exposure. Because this study population had a high fish

© 2025 Taylor KW et al. *JAMA Pediatrics*.

consumption (i.e., >50% consumed fish at least once a week), not accounting for fish consumption could bias the results.

Basis for rating: Probably high risk of bias because there is indirect evidence that the study population had high seafood consumption that should have been addressed in the analysis and the lack of adjusting could significantly impact the results for this study population.

Attrition:

Rating: Definitely low risk of bias (++)

Summary: Date are complete; 562 children who completed the measurement of urinary fluoride, the evaluation of dental fluorosis, and the assessment of intelligence were included in the data analysis.

Basis for rating: Definitely low risk of bias based on direct evidence that there was no attrition.

Exposure:

Rating: Probably low risk of bias (+)

Summary: Fluoride in spot urine samples was assessed using ion-selective electrode technique. Handling and storage are briefly described. LODs are not reported nor were any methods of QC. Urinary dilution was addressed by using creatinine-adjusted levels. Dental fluorosis was assessed according to the criteria of Dean’s Index by two trained and calibrated dentists.

Direction/magnitude of effect size: Not applicable.

Basis for rating: Probably low risk of bias based on indirect evidence that exposure was consistently assessed using well-established methods that directly measured exposure.

Outcome:

Rating: Definitely low risk of bias (++)

Summary: Intelligence was measured using the Taiwan edition of Raven’s Colored Progressive Matrices-Parallel, Standard Progressive Matrices-Parallel) (++ for methods). All tests were administered in the children’s classrooms by the same qualified psychologist following the guidance of the test manual. The original scores from the tests were converted into standardized IQ scores. There is no mention of whether the evaluator was blinded to the fluoride exposure levels of each child. Correspondence with study authors confirmed that "The psychologist and research assistants were blinded to the children’s dental fluorosis status, urinary fluoride levels, and usages of fluoride products" (++ for blinding). Overall rating = ++ for methods and blinding.

Basis for rating: Definitely low risk of bias based on direct evidence that the outcome was assessed using instruments that were valid and reliable in the study population, and that the outcome assessor was blind to participants’ fluoride exposure.

Selective Reporting:

Rating: Probably low risk of bias (+)

Summary: All outcomes outlined in the abstract, introduction, and methods were reported in sufficient detail. Author communication did not provide requested results for exposures expressed as creatinine adjusted in mg/L units. The authors claimed not to understand the formula that other researchers used to convert from mg/g cr to mg/L and present Bashash et al. (2017) formula and rationale, but no additional details about why this formula does not apply here.

Basis for rating: Probably low risk of bias based on indirect evidence that some data are not available.

Other potential threats:

Rating: Probably low risk of bias (+)

Summary:

Statistical analyses: Box plots and Kolmogorov–Smirnov test were used to evaluate the normality of distributions for continuous variables. The Student's t-test, analysis of variance (ANOVA) followed by Tukey's post hoc test, Pearson correlation coefficients, and Chi-square tests were used to compare variables. Multiple regression analyses were performed to assess the association between ln-transformed urinary fluoride and IQ scores. Clustering at the school level was not accounted for in multivariate analyses, but the study accounted for area of school. The impact of clustering on the effect estimates is expected to be minimal given the use of individual-level data and adjustment for several important covariates. Results are reported as slopes per 1 ln-mg/L increase in urinarily fluoride and as change in mean IQ score for a 10% increase in urinary fluoride. Details on how the latter were calculated were obtained from communication with the study authors.

Other potential concerns: None identified.

Basis for rating: Probably low risk of bias is based on indirect evidence that the statistical analyses were appropriate and there were no other potential threats of risk of bias identified.

Basis for classification as low risk-of-bias study overall: Definitely or probably low risk-of-bias ratings in exposure and outcome. Study strengths include individual exposure measurements, blinding of outcome assessor to participants' fluoride exposure, the consideration of key covariates, and addressing dilution in the urine samples, but the study is limited by its cross-sectional study design.

Xia et al. (2023)

Study Details

Study design: Cross-sectional

Population: Primary school children aged 8–12 years.

Study area: Jiangsu, China

Sample size: 721 school children

Data relevant to the review: Adjusted associations between child's urinary fluoride and IQ scores in control and high fluoride areas.

Reported association with fluoride exposure: Yes: Significant inverse association between child's urinary fluoride and IQ scores in 8–12-year-old children (adjusted β for IQ in high fluoride group = −4.08 per 1-mg/L increase; 95% CI: −3.04, −1.32; adjusted β for IQ in control fluoride group = 1.42 per 1-mg/L increase; 95% CI: −0.47, 3.32).

Risk of Bias

Author contacts:

Authors were contacted in November 2023 for additional quantitative data not reported in the manuscript. No response was received to the email request for information.

Population selection:

Rating: Probably low risk of bias (+)

Summary: Study participants were children from rural Jiangsu, China from three communities affected by drinking-water fluorosis and three communities with normal fluoride water levels from 2018 to 2019. Children aged 8–12 years who lived locally for more than 5 years were recruited from one primary school in each village via cluster random sampling to select one class from grades 3 to 6. Thirty students were randomly selected from that class or from neighboring classes. Reasons for exclusion do not appear to be related to exposure and outcome. Participant characteristics are presented by exposure group.

© 2025 Taylor KW et al. *JAMA Pediatrics*.

Basis for rating: Probably low risk of bias based on indirect evidence that subjects were similar and recruited using the same methods during the same timeframe.

Confounding:

Rating: Probably low risk of bias (+)

Summary: Sex, age, BMI, and family education level were evaluated as potential confounders. Covariates not considered included parity, quantity and quality of caregiving environment, and co-exposures (such as arsenic, lead, mercury) or iodine deficiency. Based on water quality maps and other studies conducted in this area, arsenic is not expected to be a major concern in this study population. Questionnaires were used to collect demographic data (date of birth, gender, level of parental education, etc.), but few details were provided including who answered the questions. Doctors from the Xuzhou CDC took the physical measurements in accordance with accepted procedures.

Potentially important study-specific covariates: All key covariates were addressed.

Direction/magnitude of effect size: Not applicable.

Basis for rating: Probably low risk of bias based on direct evidence that key covariates were addressed and indirect evidence that the methods used to collect the information were valid and reliable and that arsenic is not likely to be an issue in this study population.

Attrition:

Rating: Definitely low risk of bias (++)

Summary: All attrition was clearly reported and there were only 10 of the initial 721 participants that were not included. Rationales for exclusion are clearly stated: four children were not native to the area or had been residents for less than five years; two children did not complete the IQ test; and three children had no urine samples collected.

Basis for rating: Definitely low risk of bias based on direct evidence that exclusion of subjects from analyses was adequately addressed, and reasons were documented when subjects were removed from the study or excluded from analyses.

Exposure:

Rating: Probably low risk of bias (+)

Summary: Study participants were initially selected based on fluoride levels in the village. Children residing in the village for less than 5 years were excluded. Urine and water samples were collected by CDC practitioners. Water samples were collected from two randomly chosen homes as water came from a centralized delivery system. Fluoride concentrations were measured using a fluoride ion-selective electrode method. Recovery rates were noted to be between 90.3% and 105.9%. Urinary fluoride was corrected for specific gravity. Water concentrations were higher in the high fluoride areas compared to the control area. It was also noted that water improvements were completed at the time the study was conducted which may have impacted the exposure measurements.

Direction/magnitude of effect size: Not applicable.

Basis for rating: Probably low risk of bias based on indirect evidence that exposure was consistently assessed using well-established methods that directly measured exposure.

Outcome:

Rating: Probably low risk of bias (+)

Summary: IQ was measured using a computerized version of the Raven Intelligence Test, version 2. CRT-RC2 is a well-established method (++ for method). Although it was not stated if the assessors were blind, the test was stated to be computerized and it is assumed that the computer scored the test, although it was only noted that "IQ scores were calculated accordingly" (+ for blinding). Overall rating = + for methods and blinding.

Basis for rating: Probably low risk of bias based on direct evidence that the outcome was assessed using instruments that were valid and reliable in the study population, and indirect evidence that the outcome assessor was blind to participants' fluoride exposure.

© 2025 Taylor KW et al. *JAMA Pediatrics*.

Selective Reporting:

Rating: Definitely low risk of bias (++)

Summary: All outcomes outlined in the abstract, introduction, and methods were reported in sufficient detail.

Basis for rating: Definitely low risk of bias based on direct evidence that all measured outcomes were reported.

Other potential threats:

Rating: Probably low risk of bias (+)

Summary:

Statistical analyses: Non-parametric tests or analysis of variance was used to detect significant differences between the exposure groups. Generalized linear models were used to assess the association between urinary fluoride and IQ. Models were tested for normality of residuals, multicollinearity, extreme values, and outliers. Two outliers were removed, but this had little impact on the study's overall conclusions, so were retained. Clustering at the village level was not accounted for in multivariate analyses. The impact of clustering on the effect estimates is expected to be minimal given the use of individual-level data and adjustment for several important covariates.

Other potential concerns: None identified.

Basis for rating: Probably low risk if bias is based on indirect evidence that the statistical analyses were appropriate and there were no other potential threats of risk of bias identified.

Basis for classification as low risk-of-bias study overall: Definitely or probably low risk-of-bias ratings in confounding, exposure, and outcome. Study strengths include individual exposure measurements, blinding of outcome assessor to participants' fluoride exposure, consideration of key covariates, and the addressing of dilution in the urine samples, but is limited by the cross-sectional study design.

References

1. Xiang Q, Liang Y, Chen L, et al. Effect of fluoride in drinking water on children's intelligence. *Fluoride*. 2003;36:84-94.

2. Xiang QY, Liang YX, Zhou MS, Zang HB. Blood lead of children in Wamiao-Xinhuai intelligence study. *Fluoride*. 2003;36:198-199.

3. Xiang Q, Wang Y, Yang M, Zhang M, Xu Y. Level of fluoride and arsenic in household shallow well water in Wamiao and Xinhuai villages in Jiangsu province, China. *Fluoride*. 2013;46:192-197.

4. National Academies of Sciences Engineering and Medicine (NASEM). *Review of the draft NTP monograph: Systematic review of fluoride exposure and neurodevelopmental and cognitive health effects*. National Academies of Sciences, Engineering and Medicine; 2020. https://doi.org/10.17226/25715.

5. Ding Y, Sun H, Han H, et al. The relationships between low levels of urine fluoride on children's intelligence, dental fluorosis in endemic fluorosis areas in Hulunbuir, Inner Mongolia, China. *J Hazard Mater*. 2011;186:1942-1946.

6. National Academies of Sciences Engineering and Medicine (NASEM). *Review of the revised NTP Monograph on the systematic review of fluoride exposure and neurodevelopmental and cognitive health effects: A letter report*. National Academies of Sciences, Engineering and Medicine; 2021. https://doi.org/10.17226/26030

7. Seraj B, Shahrabi M, Shadfar M, et al. Effect of high water fluoride concentration on the intellectual development of children in Makoo, Iran. *J Dent*. 2012;9:221-229.

8. Trivedi M, Sangai N, Patel R, Payak M, Vyas S. Assessment of groundwater quality with special reference to fluoride and its impact on IQ of schoolchildren in six villages of the Mundra Region, Kachchh, Gujurat, India. *Fluoride*. 2012;45(4):377-383.

9. Trivedi MH, Verma RJ, Chinoy NJ, Patel RS, Sathawara NG. Effect of high fluoride water on intelligence of school children in India. *Fluoride*. 2007;40:178-183.

10. Zhang S, Zhang X, Liu H, et al. Modifying effect of COMT gene polymorphism and a predictive role for proteomics analysis in children's intelligence in endemic fluorosis area in Tianjin, China. *Toxicol Sci*. 2015;144:238-245.

11. Bashash M, Thomas D, Hu H, et al. Prenatal fluoride exposure and cognitive outcomes in children at 4 and 6-12 years of age in Mexico. *Environ Health Perspect*. 2017;125(9):1-12. doi:https://doi.org/10.1289/ehp655

12. Valdez Jimenez L, Lopez Guzman OD, Cervantes Flores M, et al. In utero exposure to fluoride and cognitive development delay in infants. *Neurotoxicology*. Mar 2017;59:65-70. doi:https://dx.doi.org/10.1016/j.neuro.2016.12.011

13. Cui Y, Zhang B, Ma J, et al. Dopamine receptor D2 gene polymorphism, urine fluoride, and intelligence impairment of children in China: A school-based cross-sectional study. *Ecotoxicol Environ Saf*. 2018;165:270-277. doi:https://doi.org/10.1016/j.ecoenv.2018.09.018

14. Yu X, Chen J, Li Y, et al. Threshold effects of moderately excessive fluoride exposure on children's health: A potential association between dental fluorosis and loss of excellent intelligence. *Environ Int*. 2018;118:116-124. doi:https://doi.org/10.1016/j.envint.2018.05.042

15. Green R, Lanphear B, Hornung R, et al. Association between maternal fluoride exposure during pregnancy and IQ scores in offspring in Canada. *JAMA Pediatr*. 2019:E1-E9.

16. Cui Y, Yu J, Zhang B, Guo B, Gao T, Liu H. The relationships between thyroid-stimulating hormone and/or dopamine levels in peripheral blood and IQ in children with different urinary iodine concentrations. *Neurosci Lett*. 2020;729:134981. doi:https://doi.org/10.1016/j.neulet.2020.134981

17. Till C, Green R, Flora D, et al. Fluoride exposure from infant formula and child IQ in a Canadian birth cohort. *Environ Int*. 2020;134:105315. doi:https://doi.org/10.1016/j.envint.2019.105315

18. Arbuckle TE, Fraser WD, Fisher M, et al. Cohort profile: The maternal-infant research on environmental chemicals research platform. *Paediatr Perinat Epidemiol*. Jul 2013;27(4):415-25. doi:https://doi.org/10.1111/ppe.12061

19. Till C, Green R, Grundy JG, et al. Community water fluoridation and urinary fluoride concentrations in a national sample of pregnant women in Canada. *Environ Health Perspect*. Oct 2018;126(10):107001. doi:https://doi.org/10.1289/ehp3546

20. Wang M, Liu L, Li H, et al. Thyroid function, intelligence, and low-moderate fluoride exposure among Chinese school-age children. *Environ Int*. 2020;134:105229. doi:https://doi.org/10.1016/j.envint.2019.105229

21. Cantoral A, Tellez-Rojo MM, Malin AJ, et al. Dietary fluoride intake during pregnancy and neurodevelopment in toddlers: A prospective study in the progress cohort. *Neurotoxicology*. Dec 2021;87:86-93. doi:https://dx.doi.org/10.1016/j.neuro.2021.08.015

22. Romero-Martínez M, Shamah-Levy T, Cuevas-Nasu L, et al. [Methodological design of the National Health and Nutrition Survey 2016]. *Salud Publica Mex*. 2017;59(3):299-305. doi:https://doi.org/10.21149/8593

23. Ibarluzea J, Gallastegi M, Santa-Marina L, et al. Prenatal exposure to fluoride and neuropsychological development in early childhood: 1-to 4 years old children. *Environ Res*. Oct 8 2021:112181. doi:https://dx.doi.org/10.1016/j.envres.2021.112181

24. Zhao L, Yu C, Lv J, et al. Fluoride exposure, dopamine relative gene polymorphism and intelligence: A cross-sectional study in China. *Ecotoxicol Environ Safety*. Feb 2021;209:111826. doi:https://dx.doi.org/10.1016/j.ecoenv.2020.111826

25. Feng Z, An N, Yu F, et al. Do methylenetetrahydrofolate dehydrogenase, cyclohydrolase, and formyltetrahydrofolate synthetase 1 polymorphisms modify changes in intelligence of school-age children in areas of endemic fluorosis? *Chin Med J*. 2022;135(15):1846-1854. doi:https://doi.org/10.1097/CM9.0000000000002062

26. Xu K, An N, Huang H, et al. Fluoride exposure and intelligence in school-age children: evidence from different windows of exposure susceptibility. *BMC Public Health*. Nov 4 2020;20(1):1657. doi:https://dx.doi.org/10.1186/s12889-020-09765-4

27. Goodman CV, Bashash M, Green R, et al. Domain-specific effects of prenatal fluoride exposure on child IQ at 4, 5, and 6–12 years in the ELEMENT cohort. *Environ Res*. 2022a;211:112993. doi:https://doi.org/10.1016/j.envres.2022.112993

Appendix 3. Contributors to Fluoride Exposure and Children's IQ Scores: A Systematic Review and Meta-Analysis

Oversight of the Systematic Review and Meta-analysis

Note: oversight by the NIEHS/DTT principle authors included directing and oversight of all steps including problem formulation, protocol development, literature search, screening, data extraction, risk of bias, visualization, evidence synthesis, meta-analysis, quality control (QA/QC), writing, and review.

Division of Translational Toxicology, National Institute of Environmental Health Sciences, Research Triangle Park, North Carolina, USA

Kyla W. Taylor, Ph.D.; over 10 years of experience
Andrew A. Rooney[1], Ph.D; over 20 years of experience
John R. Bucher[1], Ph.D.; over 35 years of experience

Systematic Review

Literature Search

ICF, Reston, Virginia, USA

Michelle Cawley, M.A., M.L.S.; over 10 years of experience
Jeremy S. Frye, M.S.L.S.; over 5 years of experience
Nicole Vetter, M.S.L.S.; over 10 years of experience

Screening

ICF, Reston, Virginia, USA

Carlye A. Austin, Ph.D.; over 5 years of experience
Robyn B. Blain, Ph.D.; over 20 years of experience
Ryan Cronk, Ph.D.; over 1 year of experience
Katie Duke, Ph.D.; over 1 year of experience
Sorina E. Eftim, Ph.D.; over 15 years of experience
Anna Engstrom, Ph.D.; over 1 year of experience
Lauren Fitzharris, M.P.H.; over 1 year of experience
Susan Goldhaber, M.P.H.; over 5 years of experience
Samantha Goodman, M.S.; over 1 year of experience
Pamela A. Hartman, M.E.M.; over 20 years of experience
Angela Hensel, B.A.; over 1 year of experience
Melinda Hoang, M.P.H.; over 1 year of experience
Allison Killius, B.S.; over 1 year of experience
Alex Kliminsky, M.S.; over 1 year of experience
Kristen Magnuson, M.E.S.M.; over 5 years of experience
Shannon McGinnis, Ph.D.; over 1 year of experience
Devon Morgan, M.S.; over 1 year of experience
Revathi Muralidharan, B.S.; over 1 year of experience

---

[1] Authors and developers of the OHAT approach to systematic review, one of the first methods for systematic review for assessing evidence from environmental exposures. Rooney AA, Boyles AL, Wolfe MS, Bucher JR, Thayer KA. Systematic review and evidence integration for literature-based environmental health science assessments. *Environ Health Perspect.* 2014 Jul;122(7):711-8. doi: 10.1289/ehp.1307972

© 2025 Taylor KW et al. *JAMA Pediatrics*.

Joei Robertson, M.Sc.; over 1 year of experience
Johanna Rochester, Ph.D.; over 5 years of experience
Courtney Rosenthal, M.S.; over 1 year of experience
Amanda Ross, M.Ed.; over 10 years of experience
Pam Ross, M.S.P.H.; over 5 years of experience
Jennifer Seed, Ph.D.; over 20 years of experience
Karen E. Setty, Ph.D.; over 1 year of experience
Codi Sharp, M.P.H.; over 1 year of experience
Kelly Shipkowski, Ph.D.; over 1 year of experience
Christopher A. Sibrizzi, M.P.H.; over 10 years of experience
Raquel A. Silva, Ph.D.; over 5 years of experience
Parnian Soleymani, M.S.; over 1 year of experience

Data extraction

ICF, Reston, Virginia, USA

Robyn B. Blain, Ph.D.; over 20 years of experience
Katie Duke, Ph.D.; over 1 year of experience
Sorina E. Eftim, Ph.D.; over 15 years of experience
Susan Goldhaber, M.P.H.; over 5 years of experience
Pamela A. Hartman, M.E.M.; over 20 years of experience
Tao Hong, Ph.D.; over 1 year of experience
Alex Lindahl, M.P.H.; over 5 years of experience
Kristen Magnuson, M.E.S.M.; over 5 years of experience
Revathi Muralidharan, B.S.; over 1 year of experience
Johanna Rochester, Ph.D.; over 5 years of experience
Jennifer Seed, Ph.D.; over 20 years of experience
Robert Shin, M.H.S.; over 1 year of experience
Christopher A. Sibrizzi, M.P.H.; over 10 years of experience
Samantha J. Snow, Ph.D., DABT; over 5 years of experience
Codi Sharp, M.P.H.; over 1 year of experience

Risk-of-bias assessment

ICF, Reston, Virginia, USA

Louise Assem, Ph.D.; over 1 year of experience
Robyn B. Blain, Ph.D.; over 20 years of experience
Kristin Bornstein, Ph.D.; over 1 year of experience
Sorina E. Eftim, Ph.D.; over 15 years of experience
Anna Engstrom, Ph.D.; over 1 year of experience
Alexandra Goldstone, M.P.H.; over 5 years of experience
Pamela A. Hartman, M.E.M.; over 20 years of experience
Tao Hong, Ph.D.; over 1 year of experience
Jennifer Hsieh, M.S.P.H.; over 1 year of experience
Alex Lindahl, M.P.H.; over 5 years of experience
Yi Lu, Ph.D.; over 1 year of experience
Kristen Magnuson, M.E.S.M.; over 5 years of experience
Johanna Rochester, Ph.D.; over 5 years of experience
Pam Ross, M.S.P.H.; over 5 years of experience
Christopher A. Sibrizzi, M.P.H.; over 10 years of experience

© 2025 Taylor KW et al. *JAMA Pediatrics*.

Samantha J. Snow, Ph.D., DABT; over 5 years of experience

Evidence Synthesis

ICF, Reston, Virginia, USA

Robyn B. Blain, Ph.D.; over 20 years of experience
Sorina E. Eftim, Ph.D.; over 15 years of experience
Pamela A. Hartman, M.E.M.; over 20 years of experience
Kristen Magnuson, M.E.S.M.; over 5 years of experience
Christopher A. Sibrizzi, M.P.H.; over 10 years of experience

QA/QC of search, screening, data extraction, risk of bias, visualizations, text, and figures

ICF, Reston, Virginia, USA

Robyn B. Blain, Ph.D.; over 20 years of experience
Sorina E. Eftim, Ph.D.; over 15 years of experience
Pamela A. Hartman, M.E.M.; over 20 years of experience
Kristen Magnuson, M.E.S.M.; over 5 years of experience
William Mendez, Ph.D.; over 40 years of experience
Christopher A. Sibrizzi, M.P.H.; over 10 years of experience

Meta-analysis

Statistical analysis

ICF, Reston, Virginia, USA

Sorina E. Eftim, Ph.D.; over 15 years of experience

Independent replication of statistical analysis

ICF, Reston, Virginia, USA

Jonathan Cohen, Ph.D.; over 20 years of experience

Manuscript development

ICF, Reston, Virginia, USA

Robyn B. Blain, Ph.D.; over 20 years of experience
Sorina E. Eftim, Ph.D.; over 15 years of experience
Pamela A. Hartman, M.E.M.; over 20 years of experience
Kristen Magnuson, M.E.S.M.; over 5 years of experience
Christopher A. Sibrizzi, M.P.H.; over 10 years of experience

Visualizations

ICF, Reston, Virginia, USA

Grace Cooney, B.A.; over 1 year of experience
Rebecca Gray, M.P.H.; over 1 year of experience
Pamela A. Hartman, M.E.M.; over 20 years of experience
Delaney Reilly, M.E.M.; over 5 years of experience

QA/QC of statistical analysis, additional QA/QC of data and data extraction, figures, and text

ICF, Reston, Virginia, USA

Robyn B. Blain, Ph.D.; over 20 years of experience
Alexandra Goldstone, M.P.H.; over 5 years of experience
Hannah Eglinton, B.A.; over 1 year of experience
Pamela A. Hartman, M.E.M.; over 20 years of experience

© 2025 Taylor KW et al. *JAMA Pediatrics*.

Cynthia J. Lin, Ph.D.; over 5 years of experience
Nathan Lothrop, Ph.D.; over 1 year of experience
Kristen Magnuson, M.E.S.M.; over 5 years of experience
Michelle Mendez, Ph.D.; over 1 year of experience
Christopher A. Sibrizzi, M.P.H.; over 10 years of experience

© 2025 Taylor KW et al. *JAMA Pediatrics*.
