# Supplementary material for: Fluoride Exposure and Children’s IQ Scores: A Systematic Review and Meta-Analysis
Source: JAMA Pediatr. 2025 Jan 6;179(3):282–92. doi: 10.1001/jamapediatrics.2024.5542 (PMC11877182; doi:10.1001/jamapediatrics.2024.5542)
Supplement: Supplement 2. — Data Sharing Statement [file jamapediatr-e245542-s002.pdf]

Data Sharing Statement

Taylor. Fluoride Exposure and Children's IQ Scores. *JAMA Pediatr*. Published January 06, 2025. doi:10.1001/jamapediatrics.2024.5542

Data
Data available: Yes
Data types: Other (please specify)
Additional Information: Data extracted from individual studies used in the meta-analysis, risk of bias assessment
How to access data: https://hawcproject.org/assessment/405/
When available: With publication

Supporting Documents
Document types: Statistical/analytic code
How to access documents: kyla.taylor@nih.gov
When available: With publication

Additional Information
Who can access the data: anyone requesting the data
Types of analyses: for any purpose
Mechanisms of data availability: directly downloadable from free and open web application
